# PlaScope: a targeted approach to assess the plasmidome from genome assemblies at the species level

**DOI:** 10.1099/mgen.0.000211

**Published:** 2018-09-28

**Authors:** G. Royer, J. W. Decousser, C. Branger, M. Dubois, C. Médigue, E. Denamur, D. Vallenet

**Affiliations:** ^1^​ Département de Microbiologie, Assistance Publique-Hôpitaux de Paris, Hôpital Henri Mondor, Université Paris Est Créteil, F-94000 Créteil, France; ^2^​ Université Paris Diderot, INSERM, IAME, UMR 1137, Sorbonne Paris Cité, F-75018 Paris, France; ^3^​ LABGeM, Génomique Métabolique, Genoscope, Institut François Jacob, CEA, CNRS, Univ Evry, Université Paris-Saclay, 91057 Evry, France; ^4^​ Assistance Publique-Hôpitaux de Paris, Hôpital Bichat, Laboratoire de Génétique Moléculaire, F-75018 Paris, France

**Keywords:** plasmid detection, bioinformatic method, antimicrobial resistance, *Escherichia coli*

## Abstract

Plasmid prediction may be of great interest when studying bacteria of medical importance such as *Enterobacteriaceae* as well as *Staphylococcus aureus* or *Enterococcus*. Indeed, many resistance and virulence genes are located on such replicons with major impact in terms of pathogenicity and spreading capacities. Beyond strain outbreak, plasmid outbreaks have been reported in particular for some extended-spectrum beta-lactamase- or carbapenemase-producing *Enterobacteriaceae*. Several tools are now available to explore the ‘plasmidome’ from whole-genome sequences with various approaches, but none of them are able to combine high sensitivity and specificity. With this in mind, we developed PlaScope, a targeted approach to recover plasmidic sequences in genome assemblies at the species or genus level. Based on Centrifuge, a metagenomic classifier, and a custom database containing complete sequences of chromosomes and plasmids from various curated databases, PlaScope classifies contigs from an assembly according to their predicted location. Compared to other plasmid classifiers, PlasFlow and cBar, it achieves better recall (0.87), specificity (0.99), precision (0.96) and accuracy (0.98) on a dataset of 70 genomes of *Escherichia coli* containing plasmids. In a second part, we identified 20 of the 21 chromosomal integrations of the extended-spectrum beta-lactamase coding gene in a clinical dataset of *E. coli* strains. In addition, we predicted virulence gene and operon locations in agreement with the literature. We also built a database for *Klebsiella* and correctly assigned the location for the majority of resistance genes from a collection of 12 *Klebsiella pneumoniae* strains. Similar approaches could also be developed for other well-characterized bacteria.

## Data Summary

1. We did not sequence new strains for this study. All the genomes were downloaded from the National Center for Biotechnology Information Sequence Read Archive and Genome database (Tables S1 and S2, available in the online version of this article).

2. The source code of PlaScope is available on Github (https://github.com/GuilhemRoyer/PlaScope).

**Impact Statement**

Plasmid exploration could be of great interest because these replicons are pivotal in the adaptation of bacteria to their environment. They are involved in the exchange of many genes within and between species, with a significant impact on antibiotic resistance and virulence in particular. However, plasmid characterization has been a laborious task for many years, requiring complex conjugation or electroporation manipulations, for example. With the advent of whole genome sequencing techniques, access to these sequences is now potentially easier provided that appropriate tools are available. Many software tools have been developed to explore the plasmidome of a large variety of bacteria, but they rarely offer the best compromise in terms of specificity and sensitivity. Here, we focus on single species or genus, and we use the many data available to overcome this problem. With our tool, PlaScope, we achieve high performance compared with two other classifiers, PlasFlow and cBar, and we demonstrate the utility of such an approach to determine the location of virulence or resistance genes. We consider that PlaScope could be very useful in the analysis of specific and well-known bacteria.

## Introduction

Recently, several studies have evaluated the effectiveness of *in silico* plasmid prediction tools [1, 2]. In fact, many bioinformatics methods are now available to detect such mobile elements, with different approaches such as read coverage analysis (e.g. PlasmidSPAdes), k-mer-based classification (e.g. cBAR, PlasFlow) and replicon detection (e.g. PlasmidFinder); some of these are fully automated [3–7], others not [8]. Some of them achieve high sensitivity: for example, PlasmidSPAdes and cBar enable plasmid recall of 0.82 and 0.76 on a dataset of 42 genomes, respectively [1]. On the other side, some tools display very high precision, for example PlasmidFinder which reaches 100 % [1]. Unfortunately, none succeeds in finding a good trade-off between sensitivity and specificity, and thus users need to combine different methods to get correct predictions.

Concomitantly, more and more sequences are becoming available in public databases, with various levels of completeness from large sets of contigs to fully circularized genomes and plasmids. Some researchers have made an effort to curate these databases and proposed high-quality datasets. Carattoli *et al.* and Orlek *et al.*, for example, have published interesting and exhaustive plasmid datasets for *Enterobacteriaceae* [4, 9].

With this in mind, we propose here a workflow, called PlaScope, to assess the plasmidome of genome assemblies. We took advantage of available genomic data to create custom databases of plasmids and chromosomes. These are used as input of the Centrifuge software, a tool originally developed as a metagenomics classifier and that is able to assign sequences based on exact matches against the database [10]. We compared it with other plasmid classifiers, cBar and PlasFlow, and showed that with our specific knowledge-based approach we were able to recover nearly all plasmids of various *Escherichia coli* strains without compromising on specificity. Finally, the usefulness of our approach is illustrated on two datasets: (i) one composed of whole genomes of *E. coli* for which we have sought to identify the location of specific genes involved in virulence or antibiotic resistance, and (ii) the other made up of whole genomes of *Klebsiella pneumoniae* for which we focused on resistance genes and highlighted putative plasmid transmission between strains.

## Theory and implementation

### Workflow description

**Fig. 1.**
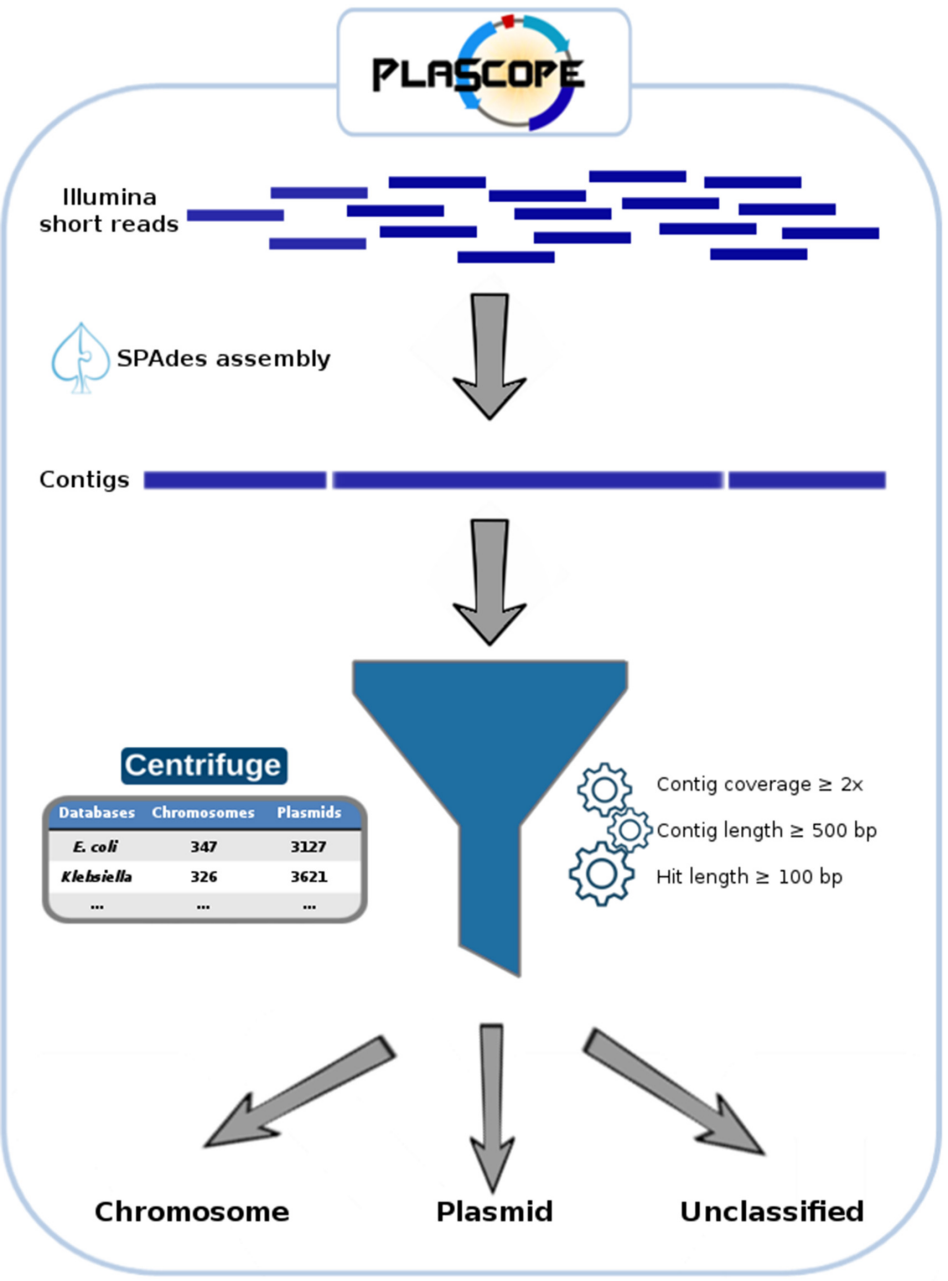
The PlaScope workflow. After read assembly using SPAdes, contigs are classified into three categories using Centrifuge (i.e. chromosome, plasmid, unclassified) with a custom database containing chromosome and plasmid sequences.

The PlaScope workflow is illustrated in Fig. 1. First, users have to provide paired end fastq files. Assembly is then run using SPAdes 3.10.1 [11] with the ‘careful’ option and automatic k-mer size selection to obtain contigs. Subsequently, Centrifuge [10] predicts the location of these contigs thanks to a custom database and sorts them into three classes: plasmid, chromosome and unclassified. The last includes (i) contigs shared by both categories (i.e. matching with plasmid and chromosome sequences from the database) and which are therefore indistinguishable, (ii) contigs without any hit against the database and (iii) contigs with length, hit length or coverage below the defined thresholds. Finally results are sorted based on those three classes and extracted using awk. The complete workflow is available through a unique bash script called PlaScope.sh on github (https://github.com/GuilhemRoyer/PlaScope) or can be installed through BioConda with all dependencies (conda install plascope).

### Centrifuge custom database construction

We gathered all the complete genome sequences (chromosomes and plasmids) of *E. coli* from the National Center for Biotechnology Information (NCBI) web site on 10 January 2018. We also added the plasmid sequences that were used to create the PlasmidFinder database [4] and those proposed by Orlek *et al.* [9]. Finally, we added a specific dataset containing *E. coli* plasmids involved in antibiotic resistance [12]. Altogether the database includes 347 chromosome and 3127 plasmid sequences (Table S1 – database available at https://doi.org/10.5281/zenodo.1311641).

We then pooled separately plasmid and chromosome sequences to create a custom database for Centrifuge 1.0.3 [10] with an artificial taxonomy containing only three nodes: ‘chromosome’, ‘plasmid’ and ‘unclassified’ (see README on https://github.com/GuilhemRoyer/PlaScope).

In the same way, we built a *Klebsiella* database. All complete genomes (326 chromosomes and 985 plasmids) of *Klebsiella* species were downloaded from the NCBI web site on 4 July 2018. In addition, the three plasmid databases (PlasmidFinder, Orlek *et al.* and Branger *et al.* datasets) were included (Table S1 – database is available at https://doi.org/10.5281/zenodo.1311647).

### Centrifuge classification method

Centrifuge has been developed as a classifier for metagenomic reads. It identifies exact matches between the input sequences and a database originally composed of sequences from several species. It then assigns a score to each of the species that match with the reads and go through a taxonomic tree of these species to output a classification. PlaScope uses this software with a custom database (centrifuge -f - -threads 2 -x custom_database -U example.fasta -k 1 - -report-file summary.txt -S extendedresult.txt) to classify contigs as ‘chromosome’, ‘plasmid’ or ‘unclassified’, with the option ‘-k’ set to 1 in order to obtain only one taxonomic assignment. Only contigs longer than 500 bp, with a Centrifuge hit longer than 100 bp and with a SPAdes contig coverage higher than 2 are classified as plasmid- or chromosome-related. These parameters were chosen empirically to exclude low-quality contigs and short hits that may not be specific.

### Reference dataset for method evaluation

To evaluate our tool, we searched for completely finished genomes of *E. coli* with Illumina reads available on the NCBI database. All corresponding chromosome and plasmid sequences and Illumina short reads were downloaded from the NCBI on 10 January 2018, and converted into fastq files with fastq-dump from the sra-toolkit (fastq-dump - -split-files). For evaluation purposes, these genomes were not included in the centrifuge custom database.

The short reads were assembled with SPAdes 3.10.1 [11] with standard parameters and ‘careful’ option (spades.py - -careful -t 8 -1 read_1.fastq.gz -2 read_2.fastq.gz -o output_directory). After assembly, rapid identification of 16S rRNA sequences was performed on fasta files using ident-16s [13]. Twelve assemblies which did not contain *Escherichia* 16S sequences or with multiple 16S sequences from various organisms were excluded from the subsequent analyses. Finally, we kept 70 genomes containing 183 plasmids and seven genomes with no plasmid according to the NCBI database (Table S2).

We filtered the assemblies based on contig length (≥500 bp) and SPAdes coverage (≥2). Each assembly was then mapped against the corresponding complete chromosome and plasmid sequences from the NCBI database using Quast 4.6 with standard parameters [14]. Contigs that did not align on any sequence (chromosome and plasmid) or aligned on less than 50 % of their length were not considered, as well as contigs that aligned on both sequences.

### PlaScope, PlasFlow and cBar benchmark

The PlaScope, PlasFlow [5] and cBar [7] programs with default parameters were run on the reference dataset of 70 *E. coli* genomes containing plasmids. These three methods use different databases and classification approaches to sort contigs as plasmidic or chromosomal. Moreover, PlaScope and PlasFlow may assign contigs as unclassified for ambiguous results.

**Fig. 2.**
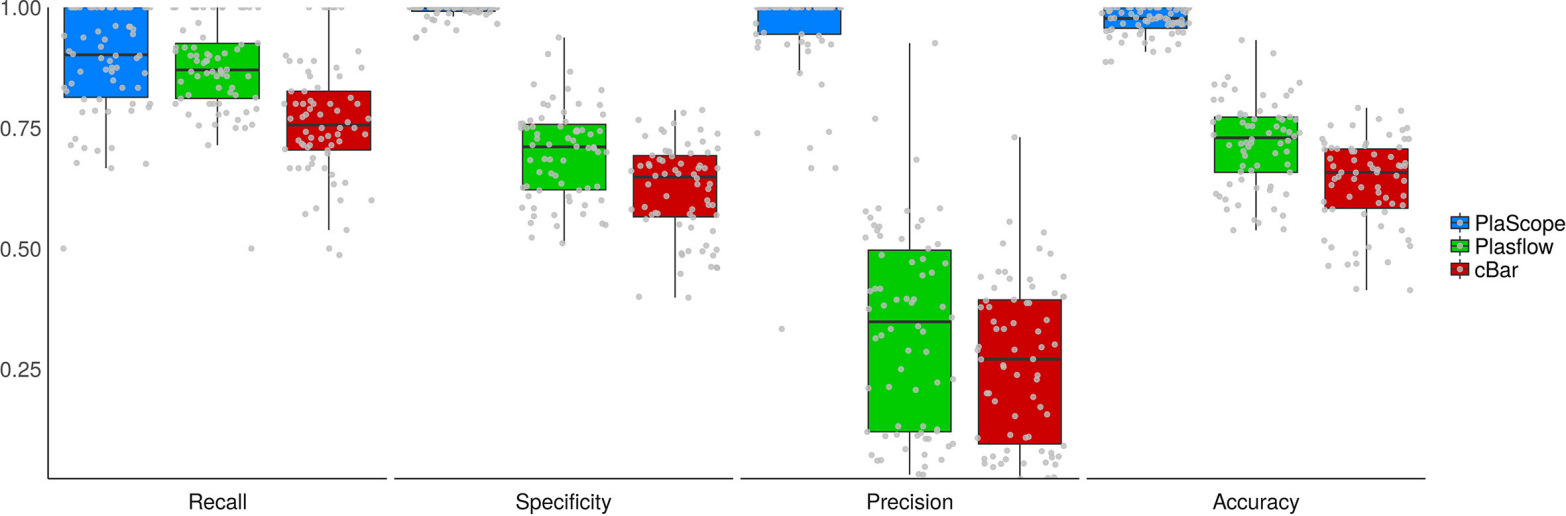
PlaScope, PlasFlow and cBar performance for each genome taken individually. Recall, specificity, precision and accuracy obtained for each of the 70 genomes containing plasmids are plotted according to the method in blue, green and red for PlaScope, PlasFlow and cBar, respectively. Grey points on box plots represent values for each of these genomes.

For each tool, the prediction for each contig was considered as (i) true positive (TP) (plasmid assignment of a plasmidic contig), (ii) true negative (TN) (chromosome or unclassified assignment of a non-plasmidic contig), (iii) false positive (FP) (plasmid assignment of a non-plasmidic contig) or (iv) false negative (FN) (chromosome or unclassified assignment of a plasmidic contig). Detailed counts of these metrics for each assignment type are provided in Table S3. We then calculated recall [TP/(TP+FN)], precision [TP/(TP+FP)], specificity [TN/(FP+TN)], accuracy [(TP+TN)/(TP+TN+FP+FN)] and F1 score [2×(recall×precision)/(recall+precision)]. The results are presented for genomes taken as a whole in Table 1 and individually in Fig. 2.

**Table 1.** PlaScope, PlasFlow and cBar benchmark results on contigs from 70 *E. coli* genomes

	**PlaScope**	**PlasFlow**	**cBar**
True positive	1123	1106	954
True negative	9162	6231	5570
False positive	52	2983	3644
False negative	173	190	342
			
Recall	0.87	0.85	0.74
Precision	0.96	0.27	0.21
Specificity	0.99	0.68	0.6
Accuracy	0.98	0.7	0.62
F1 score	0.91	0.41	0.32

PlaScope achieves the highest recall on the dataset (0.87) and is closely followed by PlasFlow (0.85), cBar having the lowest value (0.74) (Table 1). At the strain level (Fig. 2), recall values range from 0.50 to 1.00 for the three methods with the lowest median being observed with cBar (0.76) and the highest value with PlaScope (0.90). However, important differences were found for the other assessment criteria. Indeed, with PlaScope we obtained very high precision (0.96), specificity (0.99) and accuracy (0.98) compared to PlasFlow (0.27, 0.68 and 0.70, respectively) and cBar (0.21, 0.60 and 0.62, respectively). Moreover at the strain level, the dispersion of these metrics is high for PlasFlow and cBar compared to PlaScope, in particular for precision (Fig. 2). Clearly, these results are easily explained by the contents of our database, which was built specifically for *E. coli*. PlasFlow and cBar performed well in terms of recall, and their strength relies on their capacity to class many diverse taxonomic groups. Such methods can be particularly useful when working on metagenomes or on single genomes without any prior knowledge of the species, but when focusing on a particular species a targeted approach such as PlaScope drastically limits classification errors. However, 377 contigs from the 10 510 that were analysed remain unclassified with PlaScope. Among them, 248 share hits on both chromosome and plasmid, 117 have no hit and 12 have hits shorter than 100 bp.

In addition, PlaScope was run on the seven *E. coli* genomes with no plasmids. As expected, no plasmid was predicted for six genomes but, surprisingly, PlaScope predicted two plasmid contigs for *E. coli* KLY (GCA_000725305.1). To assess this result, we aligned these contigs against the NCBI database by blast n and obtained perfect alignments with the plasmid F sequence of *E. coli* K-12 C3026 (GenBank accession: CP014273.1). This result suggests that the original assembly of *E. coli* KLY is missing this plasmid.

### Application to resistance, virulence gene and operon locations in *E. coli*

In a second step, we evaluated our method on extended-spectrum beta-lactamase (ESBL)-carrying *E. coli* strains sequenced by Illumina MiSeq by Falgenhauer *et al.* [15]. These authors characterized *in silico* the genetic environment and the location of *bla*CTX-M-15 and they found an unusually high rate of chromosomal integration. Indeed, among the 27 isolates of sequence type (ST) 410, 21 carried a *bla*CTX-M-15 gene on their chromosome. We downloaded short reads of these isolates and ran PlaScope to classify the assembled contigs. In parallel, we determined the presence of CTX-M coding genes on the contigs using ResFinder (with a minimal identity of 95 % and a minimal alignment coverage of 90 %) [16].

**Fig. 3.**
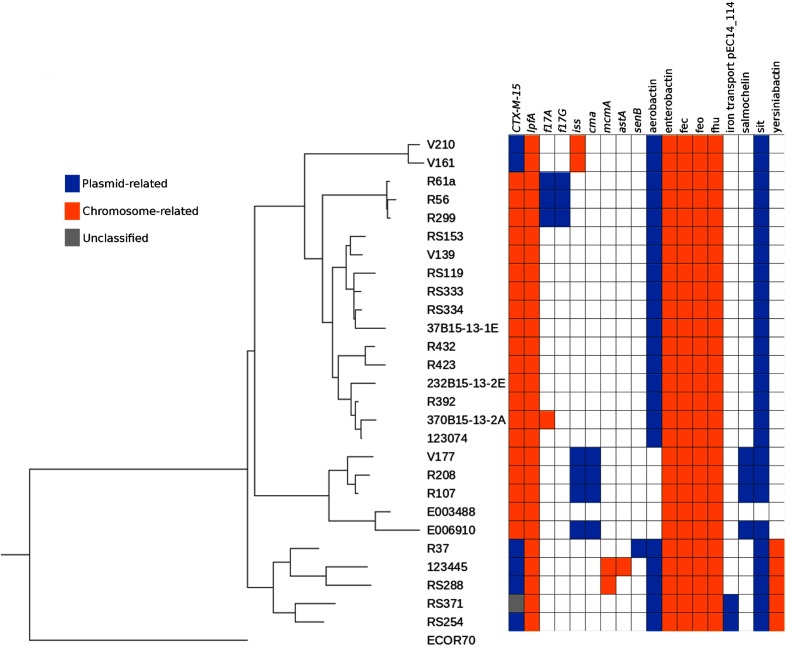
Genetic distance-based tree with PlaScope-predicted location of *bla*CTX-M-15, virulence genes and operons in the ST410 *E. coli* strains from Falgenhauer *et al.* [15]. Locations of the genes are displayed with coloured squares (blue: plasmid prediction, orange: chromosome prediction, grey: unclassified).

Using this approach, we accurately identified 20 chromosomally integrated and five plasmid-related CTX-M genes compared to the publication results. In Fig. 3, strains are classified in a neighbour-joining tree (module Phylo from biopython 1.68 [17]) rooted on strain ECOR70 [18] based on genomic similarity distances computed with Mash 2.0 (default parameters) [19]. The tree has been annotated via the Interactive Tree Of Life [20]. We only had a discrepancy with the two isolates of Clade E (strains RS254 and RS371). Indeed, we found a plasmid location of the CTX-M coding gene in strain RS254 whereas it was described as chromosome-related, probably because of an uncommon structure formed by the gene and its adjacent sequences. For the second strain, RS371, the location of the CTX-M coding gene is predicted as unclassified (i.e. hits on both chromosome and plasmid reference sequences) whereas it was stated as plasmid-located. Indeed, blast n alignment of the contig carrying this resistance gene against the GenBank database gave perfect hits on plasmid (e.g. CP029575.1) and chromosome (e.g. CP024855.1) sequences which do not allow Centrifuge to differentiate between plasmid or chromosome origin.

In the same publication, the authors also searched for virulence genes and iron metabolism operons. To go further, we used PlaScope results to determine the location of these genes (Fig. 3). Some of them are exclusively carried by chromosomes (*lpfA*, *mcmA*, *astA*) or plasmids (*f17G*, *cma*, *senB*). Interestingly, *iss* can be found on either type of replicon. For example, *iss* is on a chromosome in Clade A (strains V161 and V210) isolates whereas it is located on plasmids in four out of the five Clade C (strains E006910, R107, R208 and V177) isolates. This illustrates the different genetic background even between closely related strains. In the same way, the gene *f17A* has different locations: on plasmids in three strains (R299, R56, R61a) and on a chromosome in only one (370B15-13-2A, not described in the original publication). These two possible locations of *iss* and *f17A* were previously observed [21, 22]. Regarding the operons, five of them (i.e. enterobactin, fec, feo, fhu and yersiniabactin) were predicted as chromosome-related whereas the others (i.e. aerobactin, salmochellin, sit and the iron transporter pEC14_114) were predicted as plasmidic. These results are in agreement with the literature. Indeed, the first five are known to be chromosome-encoded [23–27] whereas iron transport pEC14_114 is plasmidic [28]. Aerobactin, salmochelin and sit have been found on both types of replicons [29].

### Application to resistance gene locations in *Klebsiella pneumoniae*

We applied PlaScope with the *Klebsiella* custom database on a dataset of 12 *K. pneumoniae* strains recovered from a patient and his hospital room environment [30]. PlasmidFinder and ResFinder were then used to identify replicon sequences and resistance genes, respectively. Among the 12 strains, the authors originally described (i) four related strains with one plasmid and no associated resistance genes, (ii) seven extensively drug-resistant (XDR) strains with many plasmids bearing resistance genes and particularly *bla*OXA-181 (a carbapenemase-coding gene) and (iii) a strain close to the four non-XDR strains but with the plasmid carrying *bla*OXA-181. Using PlaScope, we were able to find the correct location of several genes on chromosomes (*bla*SHV-11, *oqxA*, *oqxB*, *fosA*) and plasmids (APH(3″)Ib, APH(6)Id, *bla*OXA-181, *bla*TEM-1B, *catA2*) (Table S4). Furthermore, replicon sequences were detected by PlasmidFinder in 57 contigs predicted as plasmid-related by PlaScope and in only four contigs assigned as unclassified. In addition, some genes were always on unclassified contigs (*dfr*A14, QnrB1, *mph*(A), *arr*-2) as they only contain transposase and resistance genes. These cases cannot be solved by PlaScope due to assemblies being too fragmented and may only be addressed by obtaining finished genomes using long reads as performed by the authors [30]. Nonetheless, we were able to identify the plasmid location of the carbapenemase *bla*OXA-181 in the seven XDR strains and also in the strain that acquired the plasmid during patient hospitalization.

### Conclusion

Here, we propose a workflow, called PlaScope, for plasmid and chromosome classification from genome assemblies at the species level. It is based on the assembler SPAdes [11], and Centrifuge [10], a fast metagenomic classifier that uses exact matches between input sequences and a small-sized database to sort these sequences. PlaScope offers high specificity by selecting a unique assignment of contigs to plasmid, chromosome or unclassified. Indeed, we took advantage of the ever growing number of sequences from databases to build a custom database, which combines many high-quality sequences of *Enterobacteriaceae* plasmids and chromosome sequences of *E. coli*. We compared the performance of our tool with cBar and PlasFlow, as these bioinformatic software packages also enable the segregation of plasmid and chromosome contigs. These latter two programs rely on genomic signatures and have been developed to predict plasmid sequences in metagenomic samples.

Compared to PlaScope (recall=0.87), PlasFlow achieves roughly the same recall value on our dataset (recall=0.85), whereas cBar performed less well (recall=0.74). However, regarding other criteria such as precision, specificity and accuracy, PlaScope outperformed the others due to its highly specific database. cBar and PlasFlow are able to identify mobile elements in many bacterial species owing to their very diverse taxonomic database. However, when focusing on a species, the targeted approach of PlaScope gave indisputably better results in terms of both recall and precision as indicated by F1 score (PlaScope: 0.91; PlasFlow: 0.41; cBar: 0.32).

Using PlaScope, we were able to recover almost all plasmids from the analysed strains, with very high precision, specificity and accuracy. Furthermore, in one of the seven strains described as non-bearing plasmid strains in the NCBI database we were able to identify a mobile element: a typical plasmid F in *E. coli* K-12.

In a second analysis, we challenged our approach on more concrete data by looking at specific genes. Analysing clinical or environmental strains, it could be of great interest to detect specific clones with particular genetic backgrounds. Indeed, the plasmid location of a resistance or virulence gene does not have the same impact from an epidemiological point of view and from the capacity of transmission of the strain in a particular environment. For example, plasmid outbreaks can occur when a gene that confers resistance against a wide-spectrum antibiotic is carried by such a mobile element. Conversely, if the same gene integrates in the chromosome of an already highly virulent strain, it can lead to the emergence of a well-adapted and dangerous clone. To highlight this, we chose a genome dataset of *E. coli* wherein many strains exhibited a chromosomal integration of the *bla*CTX-M-15 coding gene, one of the main enzymes responsible for resistance to wide-spectrum antibiotics such as cephalosporins in *E. coli* [15]. Using PlaScope we accurately identified 20/21 of these chromosomal insertions. In addition, we predicted the location of virulence genes and iron metabolism operons in agreement with the literature. This demonstrates that PlaScope may be particularly useful to locate operons such as aerobactin or salmochellin, which can be plasmidic as well as chromosomal and have, like other iron-metabolism-related systems, major impact on virulence and/or fitness [27, 31].

We also built a *Klebsiella* database and assessed our workflow on *K. pneumoniae* clinical strains [30]. With PlaScope we were able to identify the location of the majority of the resistance genes, notably acquisition of the *bla*OXA-181 gene by a strain through plasmid transmission. However, few contigs carrying resistance genes remain unclassified as they only contain transposase and resistance genes. This is a limitation of our method that requires contigs of sufficient length with specific plasmid or chromosomal regions to make an assignment.

We consider that our approach will be useful when focusing on a well-described species as it makes it possible to decipher the plasmid content of the genomes without overpredicting plasmid sequences. It can highlight integration events or plasmid transmission between isolates. Nonetheless, as it is based on previous knowledge of plasmids found in a specific taxon (e.g. *Enterobacteriaceae*), it will require the database to be enriched to keep it up to date. Finally, it would also be interesting to create other databases for well-known bacteria with many complete genomes available, such as *Staphylococcus aureus*, *Enterococcus* or *Bacillus* species.

## Data bibliography

1. Falgenhauer L, Imirzalioglu C, Ghosh H, Gwozdzinski K, Schmiedel J *et al*. Bioproject PRJEB9568 (2016).
2. Simner PJ, Antar AAR, Hao S, Gurtowski J, Tamma PD *et al*. Bioproject PRJNA392824 (2017).

## Supplementary Data

Supplementary File 1
