# Supplementary material for: PlaScope: a targeted approach to assess the plasmidome from genome assemblies at the species level
Source: Microb Genom. 2018 Sep 28;4(9):e000211. doi: 10.1099/mgen.0.000211 (PMC6202455; doi:10.1099/mgen.0.000211)
Supplement: Supplementary File 1 [file mgen-4-211-s001.pdf]

Supplementary table S1. Sequences of the custom databases for PlaScope

Database	Sequence_accession	Molecule_type	Source
E. coli	CP006698.1	Chromosome	NCBI_complete_genomes
E. coli	CP011322.1	Chromosome	NCBI_complete_genomes
E. coli	CP011323.1	Chromosome	NCBI_complete_genomes
E. coli	CP012378.1	Chromosome	NCBI_complete_genomes
E. coli	CP013185.1	Chromosome	NCBI_complete_genomes
E. coli	CP013837.1	Chromosome	NCBI_complete_genomes
E. coli	CP014752.1	Chromosome	NCBI_complete_genomes
E. coli	CP015085.1	Chromosome	NCBI_complete_genomes
E. coli	CP024127.1	Chromosome	NCBI_complete_genomes
E. coli	CP024131.1	Chromosome	NCBI_complete_genomes
E. coli	CP024134.1	Chromosome	NCBI_complete_genomes
E. coli	CP024138.1	Chromosome	NCBI_complete_genomes
E. coli	CP024141.1	Chromosome	NCBI_complete_genomes
E. coli	CP024147.1	Chromosome	NCBI_complete_genomes
E. coli	CP024155.1	Chromosome	NCBI_complete_genomes
E. coli	CP024252.1	Chromosome	NCBI_complete_genomes
E. coli	CP024266.1	Chromosome	NCBI_complete_genomes
E. coli	CP024650.1	Chromosome	NCBI_complete_genomes
E. coli	CP025520.1	Chromosome	NCBI_complete_genomes
E. coli	NC_000913.3	Chromosome	NCBI_complete_genomes
E. coli	NC_002695.1	Chromosome	NCBI_complete_genomes
E. coli	NC_004431.1	Chromosome	NCBI_complete_genomes
E. coli	NC_007779.1	Chromosome	NCBI_complete_genomes
E. coli	NC_007946.1	Chromosome	NCBI_complete_genomes
E. coli	NC_008253.1	Chromosome	NCBI_complete_genomes
E. coli	NC_008563.1	Chromosome	NCBI_complete_genomes
E. coli	NC_009800.1	Chromosome	NCBI_complete_genomes
E. coli	NC_009801.1	Chromosome	NCBI_complete_genomes
E. coli	NC_010468.1	Chromosome	NCBI_complete_genomes
E. coli	NC_010473.1	Chromosome	NCBI_complete_genomes
E. coli	NC_010498.1	Chromosome	NCBI_complete_genomes
E. coli	NC_011353.1	Chromosome	NCBI_complete_genomes
E. coli	NC_011415.1	Chromosome	NCBI_complete_genomes
E. coli	NC_011601.1	Chromosome	NCBI_complete_genomes
E. coli	NC_011741.1	Chromosome	NCBI_complete_genomes
E. coli	NC_011742.1	Chromosome	NCBI_complete_genomes
E. coli	NC_011748.1	Chromosome	NCBI_complete_genomes
E. coli	NC_011750.1	Chromosome	NCBI_complete_genomes
E. coli	NC_011993.1	Chromosome	NCBI_complete_genomes
E. coli	NC_012759.1	Chromosome	NCBI_complete_genomes
E. coli	NC_012892.2	Chromosome	NCBI_complete_genomes
E. coli	NC_012947.1	Chromosome	NCBI_complete_genomes
E. coli	NC_012967.1	Chromosome	NCBI_complete_genomes
E. coli	NC_012971.2	Chromosome	NCBI_complete_genomes
E. coli	NC_013008.1	Chromosome	NCBI_complete_genomes
E. coli	NC_013353.1	Chromosome	NCBI_complete_genomes
E. coli	NC_013361.1	Chromosome	NCBI_complete_genomes
E. coli	NC_013364.1	Chromosome	NCBI_complete_genomes
E. coli	NC_013654.1	Chromosome	NCBI_complete_genomes
E. coli	NC_013941.1	Chromosome	NCBI_complete_genomes
E. coli	NC_017625.1	Chromosome	NCBI_complete_genomes
E. coli	NC_017626.1	Chromosome	NCBI_complete_genomes
E. coli	NC_017628.1	Chromosome	NCBI_complete_genomes
E. coli	NC_017631.1	Chromosome	NCBI_complete_genomes
E. coli	NC_017632.1	Chromosome	NCBI_complete_genomes
E. coli	NC_017633.1	Chromosome	NCBI_complete_genomes
E. coli	NC_017634.1	Chromosome	NCBI_complete_genomes
E. coli	NC_017635.1	Chromosome	NCBI_complete_genomes
E. coli	NC_017638.1	Chromosome	NCBI_complete_genomes
E. coli	NC_017641.1	Chromosome	NCBI_complete_genomes
E. coli	NC_017646.1	Chromosome	NCBI_complete_genomes
E. coli	NC_017651.1	Chromosome	NCBI_complete_genomes
E. coli	NC_017652.1	Chromosome	NCBI_complete_genomes
E. coli	NC_017656.1	Chromosome	NCBI_complete_genomes
E. coli	NC_017660.1	Chromosome	NCBI_complete_genomes
E. coli	NC_017663.1	Chromosome	NCBI_complete_genomes
E. coli	NC_017664.1	Chromosome	NCBI_complete_genomes
E. coli	NC_017906.1	Chromosome	NCBI_complete_genomes
E. coli	NC_020163.1	Chromosome	NCBI_complete_genomes
E. coli	NC_020518.1	Chromosome	NCBI_complete_genomes

E. coli	NC_022364.1	Chromosome	NCBI_complete_genomes
E. coli	NZ_CP005930.1	Chromosome	NCBI_complete_genomes
E. coli	NZ_CP005998.1	Chromosome	NCBI_complete_genomes
E. coli	NZ_CP006027.1	Chromosome	NCBI_complete_genomes
E. coli	NZ_CP006262.1	Chromosome	NCBI_complete_genomes
E. coli	NZ_CP006632.1	Chromosome	NCBI_complete_genomes
E. coli	NZ_CP006636.1	Chromosome	NCBI_complete_genomes
E. coli	NZ_CP006830.1	Chromosome	NCBI_complete_genomes
E. coli	NZ_CP006834.1	Chromosome	NCBI_complete_genomes
E. coli	NZ_CP007133.1	Chromosome	NCBI_complete_genomes
E. coli	NZ_CP007136.1	Chromosome	NCBI_complete_genomes
E. coli	NZ_CP007149.1	Chromosome	NCBI_complete_genomes
E. coli	NZ_CP007265.1	Chromosome	NCBI_complete_genomes
E. coli	NZ_CP007275.1	Chromosome	NCBI_complete_genomes
E. coli	NZ_CP007390.1	Chromosome	NCBI_complete_genomes
E. coli	NZ_CP007391.1	Chromosome	NCBI_complete_genomes
E. coli	NZ_CP007392.1	Chromosome	NCBI_complete_genomes
E. coli	NZ_CP007393.1	Chromosome	NCBI_complete_genomes
E. coli	NZ_CP007394.1	Chromosome	NCBI_complete_genomes
E. coli	NZ_CP007442.1	Chromosome	NCBI_complete_genomes
E. coli	NZ_CP007491.1	Chromosome	NCBI_complete_genomes
E. coli	NZ_CP007592.1	Chromosome	NCBI_complete_genomes
E. coli	NZ_CP007594.1	Chromosome	NCBI_complete_genomes
E. coli	NZ_CP007799.1	Chromosome	NCBI_complete_genomes
E. coli	NZ_CP008697.1	Chromosome	NCBI_complete_genomes
E. coli	NZ_CP008805.1	Chromosome	NCBI_complete_genomes
E. coli	NZ_CP009104.1	Chromosome	NCBI_complete_genomes
E. coli	NZ_CP009106.2	Chromosome	NCBI_complete_genomes
E. coli	NZ_CP009166.1	Chromosome	NCBI_complete_genomes
E. coli	NZ_CP009273.1	Chromosome	NCBI_complete_genomes
E. coli	NZ_CP009644.1	Chromosome	NCBI_complete_genomes
E. coli	NZ_CP009685.1	Chromosome	NCBI_complete_genomes
E. coli	NZ_CP009789.1	Chromosome	NCBI_complete_genomes
E. coli	NZ_CP009859.1	Chromosome	NCBI_complete_genomes
E. coli	NZ_CP010116.1	Chromosome	NCBI_complete_genomes
E. coli	NZ_CP010117.1	Chromosome	NCBI_complete_genomes
E. coli	NZ_CP010119.1	Chromosome	NCBI_complete_genomes
E. coli	NZ_CP010121.1	Chromosome	NCBI_complete_genomes
E. coli	NZ_CP010122.1	Chromosome	NCBI_complete_genomes
E. coli	NZ_CP010125.1	Chromosome	NCBI_complete_genomes
E. coli	NZ_CP010129.1	Chromosome	NCBI_complete_genomes
E. coli	NZ_CP010132.1	Chromosome	NCBI_complete_genomes
E. coli	NZ_CP010133.1	Chromosome	NCBI_complete_genomes
E. coli	NZ_CP010134.1	Chromosome	NCBI_complete_genomes
E. coli	NZ_CP010137.1	Chromosome	NCBI_complete_genomes
E. coli	NZ_CP010140.1	Chromosome	NCBI_complete_genomes
E. coli	NZ_CP010143.1	Chromosome	NCBI_complete_genomes
E. coli	NZ_CP010145.1	Chromosome	NCBI_complete_genomes
E. coli	NZ_CP010148.1	Chromosome	NCBI_complete_genomes
E. coli	NZ_CP010150.1	Chromosome	NCBI_complete_genomes
E. coli	NZ_CP010151.1	Chromosome	NCBI_complete_genomes
E. coli	NZ_CP010152.1	Chromosome	NCBI_complete_genomes
E. coli	NZ_CP010157.1	Chromosome	NCBI_complete_genomes
E. coli	NZ_CP010160.1	Chromosome	NCBI_complete_genomes
E. coli	NZ_CP010163.1	Chromosome	NCBI_complete_genomes
E. coli	NZ_CP010167.1	Chromosome	NCBI_complete_genomes
E. coli	NZ_CP010169.1	Chromosome	NCBI_complete_genomes
E. coli	NZ_CP010170.1	Chromosome	NCBI_complete_genomes
E. coli	NZ_CP010171.1	Chromosome	NCBI_complete_genomes
E. coli	NZ_CP010172.1	Chromosome	NCBI_complete_genomes
E. coli	NZ_CP010176.1	Chromosome	NCBI_complete_genomes
E. coli	NZ_CP010177.1	Chromosome	NCBI_complete_genomes
E. coli	NZ_CP010178.1	Chromosome	NCBI_complete_genomes
E. coli	NZ_CP010180.1	Chromosome	NCBI_complete_genomes
E. coli	NZ_CP010183.1	Chromosome	NCBI_complete_genomes
E. coli	NZ_CP010186.1	Chromosome	NCBI_complete_genomes
E. coli	NZ_CP010191.1	Chromosome	NCBI_complete_genomes
E. coli	NZ_CP010196.1	Chromosome	NCBI_complete_genomes
E. coli	NZ_CP010200.1	Chromosome	NCBI_complete_genomes
E. coli	NZ_CP010206.1	Chromosome	NCBI_complete_genomes
E. coli	NZ_CP010213.1	Chromosome	NCBI_complete_genomes
E. coli	NZ_CP010219.1	Chromosome	NCBI_complete_genomes

E. coli	NZ_CP010221.1	Chromosome	NCBI_complete_genomes
E. coli	NZ_CP010226.1	Chromosome	NCBI_complete_genomes
E. coli	NZ_CP010228.1	Chromosome	NCBI_complete_genomes
E. coli	NZ_CP010229.1	Chromosome	NCBI_complete_genomes
E. coli	NZ_CP010230.1	Chromosome	NCBI_complete_genomes
E. coli	NZ_CP010231.1	Chromosome	NCBI_complete_genomes
E. coli	NZ_CP010235.1	Chromosome	NCBI_complete_genomes
E. coli	NZ_CP010236.1	Chromosome	NCBI_complete_genomes
E. coli	NZ_CP010237.1	Chromosome	NCBI_complete_genomes
E. coli	NZ_CP010238.1	Chromosome	NCBI_complete_genomes
E. coli	NZ_CP010240.1	Chromosome	NCBI_complete_genomes
E. coli	NZ_CP010242.1	Chromosome	NCBI_complete_genomes
E. coli	NZ_CP010304.1	Chromosome	NCBI_complete_genomes
E. coli	NZ_CP010315.1	Chromosome	NCBI_complete_genomes
E. coli	NZ_CP010371.1	Chromosome	NCBI_complete_genomes
E. coli	NZ_CP010438.1	Chromosome	NCBI_complete_genomes
E. coli	NZ_CP010439.1	Chromosome	NCBI_complete_genomes
E. coli	NZ_CP010440.1	Chromosome	NCBI_complete_genomes
E. coli	NZ_CP010441.1	Chromosome	NCBI_complete_genomes
E. coli	NZ_CP010442.1	Chromosome	NCBI_complete_genomes
E. coli	NZ_CP010443.1	Chromosome	NCBI_complete_genomes
E. coli	NZ_CP010444.1	Chromosome	NCBI_complete_genomes
E. coli	NZ_CP010445.1	Chromosome	NCBI_complete_genomes
E. coli	NZ_CP010585.1	Chromosome	NCBI_complete_genomes
E. coli	NZ_CP010876.1	Chromosome	NCBI_complete_genomes
E. coli	NZ_CP011018.1	Chromosome	NCBI_complete_genomes
E. coli	NZ_CP011061.1	Chromosome	NCBI_complete_genomes
E. coli	NZ_CP011124.1	Chromosome	NCBI_complete_genomes
E. coli	NZ_CP011134.1	Chromosome	NCBI_complete_genomes
E. coli	NZ_CP011320.1	Chromosome	NCBI_complete_genomes
E. coli	NZ_CP011321.1	Chromosome	NCBI_complete_genomes
E. coli	NZ_CP011324.1	Chromosome	NCBI_complete_genomes
E. coli	NZ_CP011331.1	Chromosome	NCBI_complete_genomes
E. coli	NZ_CP011416.1	Chromosome	NCBI_complete_genomes
E. coli	NZ_CP011495.1	Chromosome	NCBI_complete_genomes
E. coli	NZ_CP011915.1	Chromosome	NCBI_complete_genomes
E. coli	NZ_CP012112.1	Chromosome	NCBI_complete_genomes
E. coli	NZ_CP012125.1	Chromosome	NCBI_complete_genomes
E. coli	NZ_CP012126.1	Chromosome	NCBI_complete_genomes
E. coli	NZ_CP012127.1	Chromosome	NCBI_complete_genomes
E. coli	NZ_CP012379.1	Chromosome	NCBI_complete_genomes
E. coli	NZ_CP012380.1	Chromosome	NCBI_complete_genomes
E. coli	NZ_CP012625.1	Chromosome	NCBI_complete_genomes
E. coli	NZ_CP012631.1	Chromosome	NCBI_complete_genomes
E. coli	NZ_CP012633.1	Chromosome	NCBI_complete_genomes
E. coli	NZ_CP012635.1	Chromosome	NCBI_complete_genomes
E. coli	NZ_CP012693.1	Chromosome	NCBI_complete_genomes
E. coli	NZ_CP012802.1	Chromosome	NCBI_complete_genomes
E. coli	NZ_CP012868.1	Chromosome	NCBI_complete_genomes
E. coli	NZ_CP012869.1	Chromosome	NCBI_complete_genomes
E. coli	NZ_CP012870.1	Chromosome	NCBI_complete_genomes
E. coli	NZ_CP013029.1	Chromosome	NCBI_complete_genomes
E. coli	NZ_CP013031.1	Chromosome	NCBI_complete_genomes
E. coli	NZ_CP013112.1	Chromosome	NCBI_complete_genomes
E. coli	NZ_CP013190.1	Chromosome	NCBI_complete_genomes
E. coli	NZ_CP013253.1	Chromosome	NCBI_complete_genomes
E. coli	NZ_CP013483.1	Chromosome	NCBI_complete_genomes
E. coli	NZ_CP013662.1	Chromosome	NCBI_complete_genomes
E. coli	NZ_CP013663.1	Chromosome	NCBI_complete_genomes
E. coli	NZ_CP013831.1	Chromosome	NCBI_complete_genomes
E. coli	NZ_CP013835.1	Chromosome	NCBI_complete_genomes
E. coli	NZ_CP013952.1	Chromosome	NCBI_complete_genomes
E. coli	NZ_CP014197.1	Chromosome	NCBI_complete_genomes
E. coli	NZ_CP014225.1	Chromosome	NCBI_complete_genomes
E. coli	NZ_CP014268.2	Chromosome	NCBI_complete_genomes
E. coli	NZ_CP014269.1	Chromosome	NCBI_complete_genomes
E. coli	NZ_CP014270.1	Chromosome	NCBI_complete_genomes
E. coli	NZ_CP014272.1	Chromosome	NCBI_complete_genomes
E. coli	NZ_CP014314.1	Chromosome	NCBI_complete_genomes
E. coli	NZ_CP014348.1	Chromosome	NCBI_complete_genomes
E. coli	NZ_CP014488.1	Chromosome	NCBI_complete_genomes
E. coli	NZ_CP014492.1	Chromosome	NCBI_complete_genomes

E. coli	NZ_CP014495.1	Chromosome	NCBI_complete_genomes
E. coli	NZ_CP014497.1	Chromosome	NCBI_complete_genomes
E. coli	NZ_CP014522.1	Chromosome	NCBI_complete_genomes
E. coli	NZ_CP014583.1	Chromosome	NCBI_complete_genomes
E. coli	NZ_CP014641.1	Chromosome	NCBI_complete_genomes
E. coli	NZ_CP014642.1	Chromosome	NCBI_complete_genomes
E. coli	NZ_CP014670.1	Chromosome	NCBI_complete_genomes
E. coli	NZ_CP015020.1	Chromosome	NCBI_complete_genomes
E. coli	NZ_CP015023.1	Chromosome	NCBI_complete_genomes
E. coli	NZ_CP015069.1	Chromosome	NCBI_complete_genomes
E. coli	NZ_CP015074.2	Chromosome	NCBI_complete_genomes
E. coli	NZ_CP015076.1	Chromosome	NCBI_complete_genomes
E. coli	NZ_CP015138.1	Chromosome	NCBI_complete_genomes
E. coli	NZ_CP015228.1	Chromosome	NCBI_complete_genomes
E. coli	NZ_CP015229.1	Chromosome	NCBI_complete_genomes
E. coli	NZ_CP015240.1	Chromosome	NCBI_complete_genomes
E. coli	NZ_CP015241.1	Chromosome	NCBI_complete_genomes
E. coli	NZ_CP015244.1	Chromosome	NCBI_complete_genomes
E. coli	NZ_CP015831.1	Chromosome	NCBI_complete_genomes
E. coli	NZ_CP015832.1	Chromosome	NCBI_complete_genomes
E. coli	NZ_CP015834.1	Chromosome	NCBI_complete_genomes
E. coli	NZ_CP015843.2	Chromosome	NCBI_complete_genomes
E. coli	NZ_CP015912.1	Chromosome	NCBI_complete_genomes
E. coli	NZ_CP015995.1	Chromosome	NCBI_complete_genomes
E. coli	NZ_CP016018.1	Chromosome	NCBI_complete_genomes
E. coli	NZ_CP016182.2	Chromosome	NCBI_complete_genomes
E. coli	NZ_CP016358.1	Chromosome	NCBI_complete_genomes
E. coli	NZ_CP016404.1	Chromosome	NCBI_complete_genomes
E. coli	NZ_CP016497.1	Chromosome	NCBI_complete_genomes
E. coli	NZ_CP016546.1	Chromosome	NCBI_complete_genomes
E. coli	NZ_CP016628.1	Chromosome	NCBI_complete_genomes
E. coli	NZ_CP016755.1	Chromosome	NCBI_complete_genomes
E. coli	NZ_CP016828.1	Chromosome	NCBI_complete_genomes
E. coli	NZ_CP017100.1	Chromosome	NCBI_complete_genomes
E. coli	NZ_CP017249.1	Chromosome	NCBI_complete_genomes
E. coli	NZ_CP017251.1	Chromosome	NCBI_complete_genomes
E. coli	NZ_CP017631.1	Chromosome	NCBI_complete_genomes
E. coli	NZ_CP017669.1	Chromosome	NCBI_complete_genomes
E. coli	NZ_CP017844.1	Chromosome	NCBI_complete_genomes
E. coli	NZ_CP018103.1	Chromosome	NCBI_complete_genomes
E. coli	NZ_CP018109.1	Chromosome	NCBI_complete_genomes
E. coli	NZ_CP018115.1	Chromosome	NCBI_complete_genomes
E. coli	NZ_CP018121.1	Chromosome	NCBI_complete_genomes
E. coli	NZ_CP018206.1	Chromosome	NCBI_complete_genomes
E. coli	NZ_CP018237.1	Chromosome	NCBI_complete_genomes
E. coli	NZ_CP018239.1	Chromosome	NCBI_complete_genomes
E. coli	NZ_CP018241.1	Chromosome	NCBI_complete_genomes
E. coli	NZ_CP018243.1	Chromosome	NCBI_complete_genomes
E. coli	NZ_CP018245.1	Chromosome	NCBI_complete_genomes
E. coli	NZ_CP018247.1	Chromosome	NCBI_complete_genomes
E. coli	NZ_CP018250.1	Chromosome	NCBI_complete_genomes
E. coli	NZ_CP018252.1	Chromosome	NCBI_complete_genomes
E. coli	NZ_CP018323.1	Chromosome	NCBI_complete_genomes
E. coli	NZ_CP018801.1	Chromosome	NCBI_complete_genomes
E. coli	NZ_CP018840.1	Chromosome	NCBI_complete_genomes
E. coli	NZ_CP019213.1	Chromosome	NCBI_complete_genomes
E. coli	NZ_CP019558.1	Chromosome	NCBI_complete_genomes
E. coli	NZ_CP019560.1	Chromosome	NCBI_complete_genomes
E. coli	NZ_CP019777.1	Chromosome	NCBI_complete_genomes
E. coli	NZ_CP019778.1	Chromosome	NCBI_complete_genomes
E. coli	NZ_CP019903.1	Chromosome	NCBI_complete_genomes
E. coli	NZ_CP019944.1	Chromosome	NCBI_complete_genomes
E. coli	NZ_CP020092.1	Chromosome	NCBI_complete_genomes
E. coli	NZ_CP020106.1	Chromosome	NCBI_complete_genomes
E. coli	NZ_CP020107.1	Chromosome	NCBI_complete_genomes
E. coli	NZ_CP020368.1	Chromosome	NCBI_complete_genomes
E. coli	NZ_CP020543.1	Chromosome	NCBI_complete_genomes
E. coli	NZ_CP020933.1	Chromosome	NCBI_complete_genomes
E. coli	NZ_CP021175.1	Chromosome	NCBI_complete_genomes
E. coli	NZ_CP021202.1	Chromosome	NCBI_complete_genomes
E. coli	NZ_CP021207.1	Chromosome	NCBI_complete_genomes
E. coli	NZ_CP021288.1	Chromosome	NCBI_complete_genomes

E. coli	NZ_CP021335.1	Chromosome	NCBI_complete_genomes
E. coli	NZ_CP021339.1	Chromosome	NCBI_complete_genomes
E. coli	NZ_CP021454.1	Chromosome	NCBI_complete_genomes
E. coli	NZ_CP021840.1	Chromosome	NCBI_complete_genomes
E. coli	NZ_CP021844.1	Chromosome	NCBI_complete_genomes
E. coli	NZ_CP022154.1	Chromosome	NCBI_complete_genomes
E. coli	NZ_CP022164.1	Chromosome	NCBI_complete_genomes
E. coli	NZ_CP022393.1	Chromosome	NCBI_complete_genomes
E. coli	NZ_CP022407.1	Chromosome	NCBI_complete_genomes
E. coli	NZ_CP022912.1	Chromosome	NCBI_complete_genomes
E. coli	NZ_CP023346.1	Chromosome	NCBI_complete_genomes
E. coli	NZ_CP023349.1	Chromosome	NCBI_complete_genomes
E. coli	NZ_CP023353.1	Chromosome	NCBI_complete_genomes
E. coli	NZ_CP023357.1	Chromosome	NCBI_complete_genomes
E. coli	NZ_CP023359.1	Chromosome	NCBI_complete_genomes
E. coli	NZ_CP023364.1	Chromosome	NCBI_complete_genomes
E. coli	NZ_CP023366.1	Chromosome	NCBI_complete_genomes
E. coli	NZ_CP023371.1	Chromosome	NCBI_complete_genomes
E. coli	NZ_CP023377.1	Chromosome	NCBI_complete_genomes
E. coli	NZ_CP023383.1	Chromosome	NCBI_complete_genomes
E. coli	NZ_CP023386.1	Chromosome	NCBI_complete_genomes
E. coli	NZ_CP023388.1	Chromosome	NCBI_complete_genomes
E. coli	NZ_CP023815.1	Chromosome	NCBI_complete_genomes
E. coli	NZ_CP024090.1	Chromosome	NCBI_complete_genomes
E. coli	NZ_CP024223.1	Chromosome	NCBI_complete_genomes
E. coli	NZ_CP024232.1	Chromosome	NCBI_complete_genomes
E. coli	NZ_CP024239.1	Chromosome	NCBI_complete_genomes
E. coli	NZ_CP024240.1	Chromosome	NCBI_complete_genomes
E. coli	NZ_CP024243.1	Chromosome	NCBI_complete_genomes
E. coli	NZ_CP024245.1	Chromosome	NCBI_complete_genomes
E. coli	NZ_CP024256.1	Chromosome	NCBI_complete_genomes
E. coli	NZ_CP024257.1	Chromosome	NCBI_complete_genomes
E. coli	NZ_CP024263.1	Chromosome	NCBI_complete_genomes
E. coli	NZ_CP024275.1	Chromosome	NCBI_complete_genomes
E. coli	NZ_CP024278.1	Chromosome	NCBI_complete_genomes
E. coli	NZ_CP024289.1	Chromosome	NCBI_complete_genomes
E. coli	NZ_CP024717.1	Chromosome	NCBI_complete_genomes
E. coli	NZ_CP024720.1	Chromosome	NCBI_complete_genomes
E. coli	NZ_CP024801.1	Chromosome	NCBI_complete_genomes
E. coli	NZ_CP024815.1	Chromosome	NCBI_complete_genomes
E. coli	NZ_CP024821.1	Chromosome	NCBI_complete_genomes
E. coli	NZ_CP024826.1	Chromosome	NCBI_complete_genomes
E. coli	NZ_CP024830.1	Chromosome	NCBI_complete_genomes
E. coli	NZ_CP024997.1	Chromosome	NCBI_complete_genomes
E. coli	NZ_CP025036.1	Chromosome	NCBI_complete_genomes
E. coli	NZ_CP025048.1	Chromosome	NCBI_complete_genomes
E. coli	NZ_CP025268.1	Chromosome	NCBI_complete_genomes
E. coli	NZ_CP025328.1	Chromosome	NCBI_complete_genomes
E. coli	NZ_CP025401.1	Chromosome	NCBI_complete_genomes
E. coli	NZ_HF572917.1	Chromosome	NCBI_complete_genomes
E. coli	NZ_HG738867.1	Chromosome	NCBI_complete_genomes
E. coli	NZ_HG941718.1	Chromosome	NCBI_complete_genomes
E. coli	NZ_LM993812.1	Chromosome	NCBI_complete_genomes
E. coli	NZ_LM995446.1	Chromosome	NCBI_complete_genomes
E. coli	NZ_LT601384.1	Chromosome	NCBI_complete_genomes
E. coli	NZ_LT615377.1	Chromosome	NCBI_complete_genomes
E. coli	NZ_LT615378.1	Chromosome	NCBI_complete_genomes
E. coli	NZ_LT838196.1	Chromosome	NCBI_complete_genomes
E. coli	NZ_LT883142.1	Chromosome	NCBI_complete_genomes
E. coli	NZ_LT903847.1	Chromosome	NCBI_complete_genomes
E. coli	NZ_LT906474.1	Chromosome	NCBI_complete_genomes
E. coli	CP013186.1	Plasmid	NCBI_complete_genomes
E. coli	CP013836.1	Plasmid	NCBI_complete_genomes
E. coli	CP014753.1	Plasmid	NCBI_complete_genomes
E. coli	CP015086.1	Plasmid	NCBI_complete_genomes
E. coli	CP015087.1	Plasmid	NCBI_complete_genomes
E. coli	CP015088.1	Plasmid	NCBI_complete_genomes
E. coli	CP024128.1	Plasmid	NCBI_complete_genomes
E. coli	CP024129.1	Plasmid	NCBI_complete_genomes
E. coli	CP024130.1	Plasmid	NCBI_complete_genomes
E. coli	CP024132.1	Plasmid	NCBI_complete_genomes
E. coli	CP024133.1	Plasmid	NCBI_complete_genomes

E. coli	CP024135.1	Plasmid	NCBI_complete_genomes
E. coli	CP024136.1	Plasmid	NCBI_complete_genomes
E. coli	CP024137.1	Plasmid	NCBI_complete_genomes
E. coli	CP024139.1	Plasmid	NCBI_complete_genomes
E. coli	CP024140.1	Plasmid	NCBI_complete_genomes
E. coli	CP024142.1	Plasmid	NCBI_complete_genomes
E. coli	CP024143.1	Plasmid	NCBI_complete_genomes
E. coli	CP024144.1	Plasmid	NCBI_complete_genomes
E. coli	CP024145.1	Plasmid	NCBI_complete_genomes
E. coli	CP024146.1	Plasmid	NCBI_complete_genomes
E. coli	CP024148.1	Plasmid	NCBI_complete_genomes
E. coli	CP024149.1	Plasmid	NCBI_complete_genomes
E. coli	CP024150.1	Plasmid	NCBI_complete_genomes
E. coli	CP024151.1	Plasmid	NCBI_complete_genomes
E. coli	CP024152.1	Plasmid	NCBI_complete_genomes
E. coli	CP024153.1	Plasmid	NCBI_complete_genomes
E. coli	CP024154.1	Plasmid	NCBI_complete_genomes
E. coli	CP024156.1	Plasmid	NCBI_complete_genomes
E. coli	CP024157.1	Plasmid	NCBI_complete_genomes
E. coli	CP024158.1	Plasmid	NCBI_complete_genomes
E. coli	CP024249.1	Plasmid	NCBI_complete_genomes
E. coli	CP024250.1	Plasmid	NCBI_complete_genomes
E. coli	CP024251.1	Plasmid	NCBI_complete_genomes
E. coli	CP024253.1	Plasmid	NCBI_complete_genomes
E. coli	CP024267.1	Plasmid	NCBI_complete_genomes
E. coli	CP024268.1	Plasmid	NCBI_complete_genomes
E. coli	CP024651.1	Plasmid	NCBI_complete_genomes
E. coli	CP024652.2	Plasmid	NCBI_complete_genomes
E. coli	NC_002127.1	Plasmid	NCBI_complete_genomes
E. coli	NC_002128.1	Plasmid	NCBI_complete_genomes
E. coli	NC_007941.1	Plasmid	NCBI_complete_genomes
E. coli	NC_009786.1	Plasmid	NCBI_complete_genomes
E. coli	NC_009787.1	Plasmid	NCBI_complete_genomes
E. coli	NC_009788.1	Plasmid	NCBI_complete_genomes
E. coli	NC_009789.1	Plasmid	NCBI_complete_genomes
E. coli	NC_009790.1	Plasmid	NCBI_complete_genomes
E. coli	NC_009791.1	Plasmid	NCBI_complete_genomes
E. coli	NC_009837.1	Plasmid	NCBI_complete_genomes
E. coli	NC_009838.1	Plasmid	NCBI_complete_genomes
E. coli	NC_010485.1	Plasmid	NCBI_complete_genomes
E. coli	NC_010486.1	Plasmid	NCBI_complete_genomes
E. coli	NC_010487.1	Plasmid	NCBI_complete_genomes
E. coli	NC_010488.1	Plasmid	NCBI_complete_genomes
E. coli	NC_011350.1	Plasmid	NCBI_complete_genomes
E. coli	NC_011351.1	Plasmid	NCBI_complete_genomes
E. coli	NC_011407.1	Plasmid	NCBI_complete_genomes
E. coli	NC_011408.1	Plasmid	NCBI_complete_genomes
E. coli	NC_011411.1	Plasmid	NCBI_complete_genomes
E. coli	NC_011413.1	Plasmid	NCBI_complete_genomes
E. coli	NC_011416.1	Plasmid	NCBI_complete_genomes
E. coli	NC_011419.1	Plasmid	NCBI_complete_genomes
E. coli	NC_011602.1	Plasmid	NCBI_complete_genomes
E. coli	NC_011603.1	Plasmid	NCBI_complete_genomes
E. coli	NC_013010.1	Plasmid	NCBI_complete_genomes
E. coli	NC_013354.1	Plasmid	NCBI_complete_genomes
E. coli	NC_013362.1	Plasmid	NCBI_complete_genomes
E. coli	NC_013363.1	Plasmid	NCBI_complete_genomes
E. coli	NC_013365.1	Plasmid	NCBI_complete_genomes
E. coli	NC_013366.1	Plasmid	NCBI_complete_genomes
E. coli	NC_013367.1	Plasmid	NCBI_complete_genomes
E. coli	NC_013368.1	Plasmid	NCBI_complete_genomes
E. coli	NC_013369.1	Plasmid	NCBI_complete_genomes
E. coli	NC_013370.1	Plasmid	NCBI_complete_genomes
E. coli	NC_013942.1	Plasmid	NCBI_complete_genomes
E. coli	NC_014543.1	Plasmid	NCBI_complete_genomes
E. coli	NC_017627.1	Plasmid	NCBI_complete_genomes
E. coli	NC_017629.1	Plasmid	NCBI_complete_genomes
E. coli	NC_017630.1	Plasmid	NCBI_complete_genomes
E. coli	NC_017636.1	Plasmid	NCBI_complete_genomes
E. coli	NC_017637.1	Plasmid	NCBI_complete_genomes
E. coli	NC_017639.1	Plasmid	NCBI_complete_genomes
E. coli	NC_017640.1	Plasmid	NCBI_complete_genomes

E. coli	NC_017642.1	Plasmid	NCBI_complete_genomes
E. coli	NC_017643.1	Plasmid	NCBI_complete_genomes
E. coli	NC_017645.1	Plasmid	NCBI_complete_genomes
E. coli	NC_017647.1	Plasmid	NCBI_complete_genomes
E. coli	NC_017648.1	Plasmid	NCBI_complete_genomes
E. coli	NC_017649.1	Plasmid	NCBI_complete_genomes
E. coli	NC_017650.1	Plasmid	NCBI_complete_genomes
E. coli	NC_017653.1	Plasmid	NCBI_complete_genomes
E. coli	NC_017654.1	Plasmid	NCBI_complete_genomes
E. coli	NC_017655.1	Plasmid	NCBI_complete_genomes
E. coli	NC_017657.1	Plasmid	NCBI_complete_genomes
E. coli	NC_017658.1	Plasmid	NCBI_complete_genomes
E. coli	NC_017659.1	Plasmid	NCBI_complete_genomes
E. coli	NC_017661.1	Plasmid	NCBI_complete_genomes
E. coli	NC_017662.1	Plasmid	NCBI_complete_genomes
E. coli	NC_017665.1	Plasmid	NCBI_complete_genomes
E. coli	NC_017721.1	Plasmid	NCBI_complete_genomes
E. coli	NC_017722.1	Plasmid	NCBI_complete_genomes
E. coli	NC_017723.1	Plasmid	NCBI_complete_genomes
E. coli	NC_017724.1	Plasmid	NCBI_complete_genomes
E. coli	NC_017903.1	Plasmid	NCBI_complete_genomes
E. coli	NC_017907.1	Plasmid	NCBI_complete_genomes
E. coli	NC_022741.1	Plasmid	NCBI_complete_genomes
E. coli	NC_022742.1	Plasmid	NCBI_complete_genomes
E. coli	NC_022743.1	Plasmid	NCBI_complete_genomes
E. coli	NZ_CP005931.1	Plasmid	NCBI_complete_genomes
E. coli	NZ_CP005932.1	Plasmid	NCBI_complete_genomes
E. coli	NZ_CP005999.1	Plasmid	NCBI_complete_genomes
E. coli	NZ_CP006000.1	Plasmid	NCBI_complete_genomes
E. coli	NZ_CP006001.1	Plasmid	NCBI_complete_genomes
E. coli	NZ_CP006002.1	Plasmid	NCBI_complete_genomes
E. coli	NZ_CP006028.1	Plasmid	NCBI_complete_genomes
E. coli	NZ_CP006029.1	Plasmid	NCBI_complete_genomes
E. coli	NZ_CP006263.1	Plasmid	NCBI_complete_genomes
E. coli	NZ_CP006264.1	Plasmid	NCBI_complete_genomes
E. coli	NZ_CP006633.1	Plasmid	NCBI_complete_genomes
E. coli	NZ_CP006634.1	Plasmid	NCBI_complete_genomes
E. coli	NZ_CP006635.1	Plasmid	NCBI_complete_genomes
E. coli	NZ_CP006637.1	Plasmid	NCBI_complete_genomes
E. coli	NZ_CP006638.1	Plasmid	NCBI_complete_genomes
E. coli	NZ_CP006639.1	Plasmid	NCBI_complete_genomes
E. coli	NZ_CP006640.1	Plasmid	NCBI_complete_genomes
E. coli	NZ_CP006641.1	Plasmid	NCBI_complete_genomes
E. coli	NZ_CP006642.1	Plasmid	NCBI_complete_genomes
E. coli	NZ_CP007134.1	Plasmid	NCBI_complete_genomes
E. coli	NZ_CP007135.1	Plasmid	NCBI_complete_genomes
E. coli	NZ_CP007137.1	Plasmid	NCBI_complete_genomes
E. coli	NZ_CP007138.1	Plasmid	NCBI_complete_genomes
E. coli	NZ_CP007150.1	Plasmid	NCBI_complete_genomes
E. coli	NZ_CP008715.1	Plasmid	NCBI_complete_genomes
E. coli	NZ_CP008716.1	Plasmid	NCBI_complete_genomes
E. coli	NZ_CP008717.1	Plasmid	NCBI_complete_genomes
E. coli	NZ_CP008718.1	Plasmid	NCBI_complete_genomes
E. coli	NZ_CP008719.1	Plasmid	NCBI_complete_genomes
E. coli	NZ_CP008720.1	Plasmid	NCBI_complete_genomes
E. coli	NZ_CP008721.1	Plasmid	NCBI_complete_genomes
E. coli	NZ_CP008806.1	Plasmid	NCBI_complete_genomes
E. coli	NZ_CP008807.1	Plasmid	NCBI_complete_genomes
E. coli	NZ_CP009105.1	Plasmid	NCBI_complete_genomes
E. coli	NZ_CP009107.1	Plasmid	NCBI_complete_genomes
E. coli	NZ_CP009167.1	Plasmid	NCBI_complete_genomes
E. coli	NZ_CP009168.1	Plasmid	NCBI_complete_genomes
E. coli	NZ_CP009169.1	Plasmid	NCBI_complete_genomes
E. coli	NZ_CP009860.1	Plasmid	NCBI_complete_genomes
E. coli	NZ_CP009861.1	Plasmid	NCBI_complete_genomes
E. coli	NZ_CP009862.1	Plasmid	NCBI_complete_genomes
E. coli	NZ_CP010118.1	Plasmid	NCBI_complete_genomes
E. coli	NZ_CP010120.1	Plasmid	NCBI_complete_genomes
E. coli	NZ_CP010181.1	Plasmid	NCBI_complete_genomes
E. coli	NZ_CP010182.1	Plasmid	NCBI_complete_genomes
E. coli	NZ_CP010184.1	Plasmid	NCBI_complete_genomes
E. coli	NZ_CP010185.1	Plasmid	NCBI_complete_genomes

E. coli	NZ_CP010201.1	Plasmid	NCBI_complete_genomes
E. coli	NZ_CP010202.1	Plasmid	NCBI_complete_genomes
E. coli	NZ_CP010203.1	Plasmid	NCBI_complete_genomes
E. coli	NZ_CP010204.1	Plasmid	NCBI_complete_genomes
E. coli	NZ_CP010205.1	Plasmid	NCBI_complete_genomes
E. coli	NZ_CP010207.1	Plasmid	NCBI_complete_genomes
E. coli	NZ_CP010208.1	Plasmid	NCBI_complete_genomes
E. coli	NZ_CP010209.1	Plasmid	NCBI_complete_genomes
E. coli	NZ_CP010210.1	Plasmid	NCBI_complete_genomes
E. coli	NZ_CP010211.1	Plasmid	NCBI_complete_genomes
E. coli	NZ_CP010212.1	Plasmid	NCBI_complete_genomes
E. coli	NZ_CP010214.1	Plasmid	NCBI_complete_genomes
E. coli	NZ_CP010215.1	Plasmid	NCBI_complete_genomes
E. coli	NZ_CP010216.1	Plasmid	NCBI_complete_genomes
E. coli	NZ_CP010217.1	Plasmid	NCBI_complete_genomes
E. coli	NZ_CP010218.1	Plasmid	NCBI_complete_genomes
E. coli	NZ_CP010220.1	Plasmid	NCBI_complete_genomes
E. coli	NZ_CP010222.1	Plasmid	NCBI_complete_genomes
E. coli	NZ_CP010223.1	Plasmid	NCBI_complete_genomes
E. coli	NZ_CP010224.1	Plasmid	NCBI_complete_genomes
E. coli	NZ_CP010225.1	Plasmid	NCBI_complete_genomes
E. coli	NZ_CP010227.1	Plasmid	NCBI_complete_genomes
E. coli	NZ_CP010232.1	Plasmid	NCBI_complete_genomes
E. coli	NZ_CP010233.1	Plasmid	NCBI_complete_genomes
E. coli	NZ_CP010234.1	Plasmid	NCBI_complete_genomes
E. coli	NZ_CP010239.1	Plasmid	NCBI_complete_genomes
E. coli	NZ_CP010241.1	Plasmid	NCBI_complete_genomes
E. coli	NZ_CP010243.1	Plasmid	NCBI_complete_genomes
E. coli	NZ_CP010244.1	Plasmid	NCBI_complete_genomes
E. coli	NZ_CP010245.1	Plasmid	NCBI_complete_genomes
E. coli	NZ_CP010305.1	Plasmid	NCBI_complete_genomes
E. coli	NZ_CP010316.1	Plasmid	NCBI_complete_genomes
E. coli	NZ_CP010317.1	Plasmid	NCBI_complete_genomes
E. coli	NZ_CP010318.1	Plasmid	NCBI_complete_genomes
E. coli	NZ_CP010372.1	Plasmid	NCBI_complete_genomes
E. coli	NZ_CP010373.2	Plasmid	NCBI_complete_genomes
E. coli	NZ_CP010877.1	Plasmid	NCBI_complete_genomes
E. coli	NZ_CP010878.1	Plasmid	NCBI_complete_genomes
E. coli	NZ_CP010879.1	Plasmid	NCBI_complete_genomes
E. coli	NZ_CP010880.1	Plasmid	NCBI_complete_genomes
E. coli	NZ_CP010881.1	Plasmid	NCBI_complete_genomes
E. coli	NZ_CP010882.1	Plasmid	NCBI_complete_genomes
E. coli	NZ_CP011019.1	Plasmid	NCBI_complete_genomes
E. coli	NZ_CP011062.1	Plasmid	NCBI_complete_genomes
E. coli	NZ_CP011063.1	Plasmid	NCBI_complete_genomes
E. coli	NZ_CP011064.1	Plasmid	NCBI_complete_genomes
E. coli	NZ_CP011065.1	Plasmid	NCBI_complete_genomes
E. coli	NZ_CP011066.1	Plasmid	NCBI_complete_genomes
E. coli	NZ_CP011067.1	Plasmid	NCBI_complete_genomes
E. coli	NZ_CP011135.1	Plasmid	NCBI_complete_genomes
E. coli	NZ_CP011136.1	Plasmid	NCBI_complete_genomes
E. coli	NZ_CP011137.1	Plasmid	NCBI_complete_genomes
E. coli	NZ_CP011138.1	Plasmid	NCBI_complete_genomes
E. coli	NZ_CP011139.1	Plasmid	NCBI_complete_genomes
E. coli	NZ_CP011140.1	Plasmid	NCBI_complete_genomes
E. coli	NZ_CP011141.1	Plasmid	NCBI_complete_genomes
E. coli	NZ_CP011142.1	Plasmid	NCBI_complete_genomes
E. coli	NZ_CP011143.1	Plasmid	NCBI_complete_genomes
E. coli	NZ_CP011332.1	Plasmid	NCBI_complete_genomes
E. coli	NZ_CP011333.1	Plasmid	NCBI_complete_genomes
E. coli	NZ_CP011334.1	Plasmid	NCBI_complete_genomes
E. coli	NZ_CP011335.1	Plasmid	NCBI_complete_genomes
E. coli	NZ_CP011336.1	Plasmid	NCBI_complete_genomes
E. coli	NZ_CP011337.1	Plasmid	NCBI_complete_genomes
E. coli	NZ_CP011338.1	Plasmid	NCBI_complete_genomes
E. coli	NZ_CP011417.1	Plasmid	NCBI_complete_genomes
E. coli	NZ_CP011418.1	Plasmid	NCBI_complete_genomes
E. coli	NZ_CP011496.1	Plasmid	NCBI_complete_genomes
E. coli	NZ_CP011916.1	Plasmid	NCBI_complete_genomes
E. coli	NZ_CP012113.1	Plasmid	NCBI_complete_genomes
E. coli	NZ_CP012114.1	Plasmid	NCBI_complete_genomes
E. coli	NZ_CP012491.1	Plasmid	NCBI_complete_genomes

E. coli	NZ_CP012492.1	Plasmid	NCBI_complete_genomes
E. coli	NZ_CP012493.1	Plasmid	NCBI_complete_genomes
E. coli	NZ_CP012494.1	Plasmid	NCBI_complete_genomes
E. coli	NZ_CP012495.1	Plasmid	NCBI_complete_genomes
E. coli	NZ_CP012496.1	Plasmid	NCBI_complete_genomes
E. coli	NZ_CP012498.1	Plasmid	NCBI_complete_genomes
E. coli	NZ_CP012499.1	Plasmid	NCBI_complete_genomes
E. coli	NZ_CP012500.1	Plasmid	NCBI_complete_genomes
E. coli	NZ_CP012501.1	Plasmid	NCBI_complete_genomes
E. coli	NZ_CP012626.1	Plasmid	NCBI_complete_genomes
E. coli	NZ_CP012627.1	Plasmid	NCBI_complete_genomes
E. coli	NZ_CP012628.1	Plasmid	NCBI_complete_genomes
E. coli	NZ_CP012629.1	Plasmid	NCBI_complete_genomes
E. coli	NZ_CP012630.1	Plasmid	NCBI_complete_genomes
E. coli	NZ_CP012632.1	Plasmid	NCBI_complete_genomes
E. coli	NZ_CP012634.1	Plasmid	NCBI_complete_genomes
E. coli	NZ_CP012636.1	Plasmid	NCBI_complete_genomes
E. coli	NZ_CP012637.1	Plasmid	NCBI_complete_genomes
E. coli	NZ_CP012638.1	Plasmid	NCBI_complete_genomes
E. coli	NZ_CP012803.1	Plasmid	NCBI_complete_genomes
E. coli	NZ_CP012804.1	Plasmid	NCBI_complete_genomes
E. coli	NZ_CP013028.1	Plasmid	NCBI_complete_genomes
E. coli	NZ_CP013030.1	Plasmid	NCBI_complete_genomes
E. coli	NZ_CP013191.1	Plasmid	NCBI_complete_genomes
E. coli	NZ_CP013192.1	Plasmid	NCBI_complete_genomes
E. coli	NZ_CP013193.1	Plasmid	NCBI_complete_genomes
E. coli	NZ_CP013194.1	Plasmid	NCBI_complete_genomes
E. coli	NZ_CP013832.1	Plasmid	NCBI_complete_genomes
E. coli	NZ_CP013833.1	Plasmid	NCBI_complete_genomes
E. coli	NZ_CP013834.1	Plasmid	NCBI_complete_genomes
E. coli	NZ_CP014198.2	Plasmid	NCBI_complete_genomes
E. coli	NZ_CP014199.1	Plasmid	NCBI_complete_genomes
E. coli	NZ_CP014200.1	Plasmid	NCBI_complete_genomes
E. coli	NZ_CP014271.1	Plasmid	NCBI_complete_genomes
E. coli	NZ_CP014273.1	Plasmid	NCBI_complete_genomes
E. coli	NZ_CP014489.1	Plasmid	NCBI_complete_genomes
E. coli	NZ_CP014490.1	Plasmid	NCBI_complete_genomes
E. coli	NZ_CP014491.1	Plasmid	NCBI_complete_genomes
E. coli	NZ_CP014493.1	Plasmid	NCBI_complete_genomes
E. coli	NZ_CP014494.1	Plasmid	NCBI_complete_genomes
E. coli	NZ_CP014496.1	Plasmid	NCBI_complete_genomes
E. coli	NZ_CP014498.1	Plasmid	NCBI_complete_genomes
E. coli	NZ_CP014523.1	Plasmid	NCBI_complete_genomes
E. coli	NZ_CP014524.1	Plasmid	NCBI_complete_genomes
E. coli	NZ_CP015021.1	Plasmid	NCBI_complete_genomes
E. coli	NZ_CP015022.1	Plasmid	NCBI_complete_genomes
E. coli	NZ_CP015070.1	Plasmid	NCBI_complete_genomes
E. coli	NZ_CP015071.1	Plasmid	NCBI_complete_genomes
E. coli	NZ_CP015072.1	Plasmid	NCBI_complete_genomes
E. coli	NZ_CP015073.1	Plasmid	NCBI_complete_genomes
E. coli	NZ_CP015075.2	Plasmid	NCBI_complete_genomes
E. coli	NZ_CP015077.1	Plasmid	NCBI_complete_genomes
E. coli	NZ_CP015078.1	Plasmid	NCBI_complete_genomes
E. coli	NZ_CP015139.1	Plasmid	NCBI_complete_genomes
E. coli	NZ_CP015140.1	Plasmid	NCBI_complete_genomes
E. coli	NZ_CP015141.1	Plasmid	NCBI_complete_genomes
E. coli	NZ_CP015142.1	Plasmid	NCBI_complete_genomes
E. coli	NZ_CP015143.1	Plasmid	NCBI_complete_genomes
E. coli	NZ_CP015144.1	Plasmid	NCBI_complete_genomes
E. coli	NZ_CP015239.1	Plasmid	NCBI_complete_genomes
E. coli	NZ_CP015242.1	Plasmid	NCBI_complete_genomes
E. coli	NZ_CP015245.1	Plasmid	NCBI_complete_genomes
E. coli	NZ_CP015246.1	Plasmid	NCBI_complete_genomes
E. coli	NZ_CP015816.1	Plasmid	NCBI_complete_genomes
E. coli	NZ_CP015833.1	Plasmid	NCBI_complete_genomes
E. coli	NZ_CP015835.1	Plasmid	NCBI_complete_genomes
E. coli	NZ_CP015836.1	Plasmid	NCBI_complete_genomes
E. coli	NZ_CP015837.1	Plasmid	NCBI_complete_genomes
E. coli	NZ_CP015838.1	Plasmid	NCBI_complete_genomes
E. coli	NZ_CP015844.2	Plasmid	NCBI_complete_genomes
E. coli	NZ_CP015845.2	Plasmid	NCBI_complete_genomes
E. coli	NZ_CP015913.1	Plasmid	NCBI_complete_genomes

E. coli	NZ_CP015914.1	Plasmid	NCBI_complete_genomes
E. coli	NZ_CP015915.1	Plasmid	NCBI_complete_genomes
E. coli	NZ_CP015916.1	Plasmid	NCBI_complete_genomes
E. coli	NZ_CP015917.1	Plasmid	NCBI_complete_genomes
E. coli	NZ_CP015996.1	Plasmid	NCBI_complete_genomes
E. coli	NZ_CP015997.1	Plasmid	NCBI_complete_genomes
E. coli	NZ_CP016405.1	Plasmid	NCBI_complete_genomes
E. coli	NZ_CP016498.1	Plasmid	NCBI_complete_genomes
E. coli	NZ_CP016547.1	Plasmid	NCBI_complete_genomes
E. coli	NZ_CP016548.1	Plasmid	NCBI_complete_genomes
E. coli	NZ_CP016549.1	Plasmid	NCBI_complete_genomes
E. coli	NZ_CP016550.1	Plasmid	NCBI_complete_genomes
E. coli	NZ_CP016551.1	Plasmid	NCBI_complete_genomes
E. coli	NZ_CP017250.1	Plasmid	NCBI_complete_genomes
E. coli	NZ_CP017252.1	Plasmid	NCBI_complete_genomes
E. coli	NZ_CP017632.1	Plasmid	NCBI_complete_genomes
E. coli	NZ_CP017633.1	Plasmid	NCBI_complete_genomes
E. coli	NZ_CP017670.1	Plasmid	NCBI_complete_genomes
E. coli	NZ_CP017845.1	Plasmid	NCBI_complete_genomes
E. coli	NZ_CP017846.1	Plasmid	NCBI_complete_genomes
E. coli	NZ_CP017847.1	Plasmid	NCBI_complete_genomes
E. coli	NZ_CP017848.1	Plasmid	NCBI_complete_genomes
E. coli	NZ_CP018104.1	Plasmid	NCBI_complete_genomes
E. coli	NZ_CP018105.1	Plasmid	NCBI_complete_genomes
E. coli	NZ_CP018106.1	Plasmid	NCBI_complete_genomes
E. coli	NZ_CP018107.1	Plasmid	NCBI_complete_genomes
E. coli	NZ_CP018108.1	Plasmid	NCBI_complete_genomes
E. coli	NZ_CP018110.1	Plasmid	NCBI_complete_genomes
E. coli	NZ_CP018111.1	Plasmid	NCBI_complete_genomes
E. coli	NZ_CP018112.1	Plasmid	NCBI_complete_genomes
E. coli	NZ_CP018113.1	Plasmid	NCBI_complete_genomes
E. coli	NZ_CP018114.1	Plasmid	NCBI_complete_genomes
E. coli	NZ_CP018116.1	Plasmid	NCBI_complete_genomes
E. coli	NZ_CP018117.1	Plasmid	NCBI_complete_genomes
E. coli	NZ_CP018118.1	Plasmid	NCBI_complete_genomes
E. coli	NZ_CP018119.1	Plasmid	NCBI_complete_genomes
E. coli	NZ_CP018120.1	Plasmid	NCBI_complete_genomes
E. coli	NZ_CP018122.1	Plasmid	NCBI_complete_genomes
E. coli	NZ_CP018123.1	Plasmid	NCBI_complete_genomes
E. coli	NZ_CP018124.1	Plasmid	NCBI_complete_genomes
E. coli	NZ_CP018125.1	Plasmid	NCBI_complete_genomes
E. coli	NZ_CP018126.1	Plasmid	NCBI_complete_genomes
E. coli	NZ_CP018207.1	Plasmid	NCBI_complete_genomes
E. coli	NZ_CP018208.1	Plasmid	NCBI_complete_genomes
E. coli	NZ_CP018238.1	Plasmid	NCBI_complete_genomes
E. coli	NZ_CP018240.1	Plasmid	NCBI_complete_genomes
E. coli	NZ_CP018242.1	Plasmid	NCBI_complete_genomes
E. coli	NZ_CP018244.1	Plasmid	NCBI_complete_genomes
E. coli	NZ_CP018246.1	Plasmid	NCBI_complete_genomes
E. coli	NZ_CP018248.1	Plasmid	NCBI_complete_genomes
E. coli	NZ_CP018251.1	Plasmid	NCBI_complete_genomes
E. coli	NZ_CP018253.1	Plasmid	NCBI_complete_genomes
E. coli	NZ_CP018625.1	Plasmid	NCBI_complete_genomes
E. coli	NZ_CP018626.1	Plasmid	NCBI_complete_genomes
E. coli	NZ_CP019214.1	Plasmid	NCBI_complete_genomes
E. coli	NZ_CP019215.1	Plasmid	NCBI_complete_genomes
E. coli	NZ_CP019216.1	Plasmid	NCBI_complete_genomes
E. coli	NZ_CP019559.1	Plasmid	NCBI_complete_genomes
E. coli	NZ_CP019561.1	Plasmid	NCBI_complete_genomes
E. coli	NZ_CP019904.1	Plasmid	NCBI_complete_genomes
E. coli	NZ_CP019905.1	Plasmid	NCBI_complete_genomes
E. coli	NZ_CP019906.1	Plasmid	NCBI_complete_genomes
E. coli	NZ_CP019907.1	Plasmid	NCBI_complete_genomes
E. coli	NZ_CP019908.1	Plasmid	NCBI_complete_genomes
E. coli	NZ_CP019909.1	Plasmid	NCBI_complete_genomes
E. coli	NZ_CP019910.1	Plasmid	NCBI_complete_genomes
E. coli	NZ_CP020934.1	Plasmid	NCBI_complete_genomes
E. coli	NZ_CP020935.1	Plasmid	NCBI_complete_genomes
E. coli	NZ_CP020936.1	Plasmid	NCBI_complete_genomes
E. coli	NZ_CP021176.1	Plasmid	NCBI_complete_genomes
E. coli	NZ_CP021177.1	Plasmid	NCBI_complete_genomes
E. coli	NZ_CP021203.1	Plasmid	NCBI_complete_genomes

E. coli	NZ_CP021204.1	Plasmid	NCBI_complete_genomes
E. coli	NZ_CP021205.1	Plasmid	NCBI_complete_genomes
E. coli	NZ_CP021206.1	Plasmid	NCBI_complete_genomes
E. coli	NZ_CP021208.1	Plasmid	NCBI_complete_genomes
E. coli	NZ_CP021209.1	Plasmid	NCBI_complete_genomes
E. coli	NZ_CP021210.1	Plasmid	NCBI_complete_genomes
E. coli	NZ_CP021289.1	Plasmid	NCBI_complete_genomes
E. coli	NZ_CP021336.1	Plasmid	NCBI_complete_genomes
E. coli	NZ_CP021337.1	Plasmid	NCBI_complete_genomes
E. coli	NZ_CP021340.1	Plasmid	NCBI_complete_genomes
E. coli	NZ_CP021341.1	Plasmid	NCBI_complete_genomes
E. coli	NZ_CP021841.1	Plasmid	NCBI_complete_genomes
E. coli	NZ_CP021842.1	Plasmid	NCBI_complete_genomes
E. coli	NZ_CP021843.1	Plasmid	NCBI_complete_genomes
E. coli	NZ_CP021845.1	Plasmid	NCBI_complete_genomes
E. coli	NZ_CP021846.1	Plasmid	NCBI_complete_genomes
E. coli	NZ_CP021847.1	Plasmid	NCBI_complete_genomes
E. coli	NZ_CP021871.1	Plasmid	NCBI_complete_genomes
E. coli	NZ_CP022155.1	Plasmid	NCBI_complete_genomes
E. coli	NZ_CP022156.1	Plasmid	NCBI_complete_genomes
E. coli	NZ_CP022157.1	Plasmid	NCBI_complete_genomes
E. coli	NZ_CP022158.1	Plasmid	NCBI_complete_genomes
E. coli	NZ_CP022159.1	Plasmid	NCBI_complete_genomes
E. coli	NZ_CP022165.1	Plasmid	NCBI_complete_genomes
E. coli	NZ_CP022166.1	Plasmid	NCBI_complete_genomes
E. coli	NZ_CP022167.1	Plasmid	NCBI_complete_genomes
E. coli	NZ_CP022408.1	Plasmid	NCBI_complete_genomes
E. coli	NZ_CP022913.1	Plasmid	NCBI_complete_genomes
E. coli	NZ_CP022914.1	Plasmid	NCBI_complete_genomes
E. coli	NZ_CP023347.1	Plasmid	NCBI_complete_genomes
E. coli	NZ_CP023348.1	Plasmid	NCBI_complete_genomes
E. coli	NZ_CP023350.1	Plasmid	NCBI_complete_genomes
E. coli	NZ_CP023351.1	Plasmid	NCBI_complete_genomes
E. coli	NZ_CP023352.1	Plasmid	NCBI_complete_genomes
E. coli	NZ_CP023354.1	Plasmid	NCBI_complete_genomes
E. coli	NZ_CP023355.1	Plasmid	NCBI_complete_genomes
E. coli	NZ_CP023356.1	Plasmid	NCBI_complete_genomes
E. coli	NZ_CP023358.1	Plasmid	NCBI_complete_genomes
E. coli	NZ_CP023360.1	Plasmid	NCBI_complete_genomes
E. coli	NZ_CP023361.1	Plasmid	NCBI_complete_genomes
E. coli	NZ_CP023362.1	Plasmid	NCBI_complete_genomes
E. coli	NZ_CP023363.1	Plasmid	NCBI_complete_genomes
E. coli	NZ_CP023365.1	Plasmid	NCBI_complete_genomes
E. coli	NZ_CP023367.1	Plasmid	NCBI_complete_genomes
E. coli	NZ_CP023368.1	Plasmid	NCBI_complete_genomes
E. coli	NZ_CP023369.1	Plasmid	NCBI_complete_genomes
E. coli	NZ_CP023370.1	Plasmid	NCBI_complete_genomes
E. coli	NZ_CP023372.1	Plasmid	NCBI_complete_genomes
E. coli	NZ_CP023373.1	Plasmid	NCBI_complete_genomes
E. coli	NZ_CP023374.1	Plasmid	NCBI_complete_genomes
E. coli	NZ_CP023375.1	Plasmid	NCBI_complete_genomes
E. coli	NZ_CP023376.1	Plasmid	NCBI_complete_genomes
E. coli	NZ_CP023378.1	Plasmid	NCBI_complete_genomes
E. coli	NZ_CP023379.1	Plasmid	NCBI_complete_genomes
E. coli	NZ_CP023380.1	Plasmid	NCBI_complete_genomes
E. coli	NZ_CP023381.1	Plasmid	NCBI_complete_genomes
E. coli	NZ_CP023382.1	Plasmid	NCBI_complete_genomes
E. coli	NZ_CP023384.1	Plasmid	NCBI_complete_genomes
E. coli	NZ_CP023385.1	Plasmid	NCBI_complete_genomes
E. coli	NZ_CP023387.1	Plasmid	NCBI_complete_genomes
E. coli	NZ_CP023389.1	Plasmid	NCBI_complete_genomes
E. coli	NZ_CP023552.1	Plasmid	NCBI_complete_genomes
E. coli	NZ_CP023816.1	Plasmid	NCBI_complete_genomes
E. coli	NZ_CP024224.1	Plasmid	NCBI_complete_genomes
E. coli	NZ_CP024225.1	Plasmid	NCBI_complete_genomes
E. coli	NZ_CP024226.1	Plasmid	NCBI_complete_genomes
E. coli	NZ_CP024227.1	Plasmid	NCBI_complete_genomes
E. coli	NZ_CP024233.1	Plasmid	NCBI_complete_genomes
E. coli	NZ_CP024234.1	Plasmid	NCBI_complete_genomes
E. coli	NZ_CP024235.1	Plasmid	NCBI_complete_genomes
E. coli	NZ_CP024236.1	Plasmid	NCBI_complete_genomes
E. coli	NZ_CP024237.1	Plasmid	NCBI_complete_genomes

E. coli	NZ_CP024238.1	Plasmid	NCBI_complete_genomes
E. coli	NZ_CP024241.1	Plasmid	NCBI_complete_genomes
E. coli	NZ_CP024242.1	Plasmid	NCBI_complete_genomes
E. coli	NZ_CP024244.1	Plasmid	NCBI_complete_genomes
E. coli	NZ_CP024246.1	Plasmid	NCBI_complete_genomes
E. coli	NZ_CP024247.1	Plasmid	NCBI_complete_genomes
E. coli	NZ_CP024248.1	Plasmid	NCBI_complete_genomes
E. coli	NZ_CP024254.1	Plasmid	NCBI_complete_genomes
E. coli	NZ_CP024255.1	Plasmid	NCBI_complete_genomes
E. coli	NZ_CP024258.1	Plasmid	NCBI_complete_genomes
E. coli	NZ_CP024259.1	Plasmid	NCBI_complete_genomes
E. coli	NZ_CP024264.1	Plasmid	NCBI_complete_genomes
E. coli	NZ_CP024265.1	Plasmid	NCBI_complete_genomes
E. coli	NZ_CP024276.1	Plasmid	NCBI_complete_genomes
E. coli	NZ_CP024277.1	Plasmid	NCBI_complete_genomes
E. coli	NZ_CP024279.1	Plasmid	NCBI_complete_genomes
E. coli	NZ_CP024280.1	Plasmid	NCBI_complete_genomes
E. coli	NZ_CP024281.1	Plasmid	NCBI_complete_genomes
E. coli	NZ_CP024290.1	Plasmid	NCBI_complete_genomes
E. coli	NZ_CP024291.1	Plasmid	NCBI_complete_genomes
E. coli	NZ_CP024292.1	Plasmid	NCBI_complete_genomes
E. coli	NZ_CP024718.1	Plasmid	NCBI_complete_genomes
E. coli	NZ_CP024719.1	Plasmid	NCBI_complete_genomes
E. coli	NZ_CP024721.1	Plasmid	NCBI_complete_genomes
E. coli	NZ_CP024722.1	Plasmid	NCBI_complete_genomes
E. coli	NZ_CP024802.1	Plasmid	NCBI_complete_genomes
E. coli	NZ_CP024803.1	Plasmid	NCBI_complete_genomes
E. coli	NZ_CP024804.1	Plasmid	NCBI_complete_genomes
E. coli	NZ_CP024805.1	Plasmid	NCBI_complete_genomes
E. coli	NZ_CP024806.1	Plasmid	NCBI_complete_genomes
E. coli	NZ_CP024816.1	Plasmid	NCBI_complete_genomes
E. coli	NZ_CP024817.1	Plasmid	NCBI_complete_genomes
E. coli	NZ_CP024818.1	Plasmid	NCBI_complete_genomes
E. coli	NZ_CP024822.1	Plasmid	NCBI_complete_genomes
E. coli	NZ_CP024823.1	Plasmid	NCBI_complete_genomes
E. coli	NZ_CP024824.1	Plasmid	NCBI_complete_genomes
E. coli	NZ_CP024825.1	Plasmid	NCBI_complete_genomes
E. coli	NZ_CP024827.1	Plasmid	NCBI_complete_genomes
E. coli	NZ_CP024828.1	Plasmid	NCBI_complete_genomes
E. coli	NZ_CP024829.1	Plasmid	NCBI_complete_genomes
E. coli	NZ_CP024831.1	Plasmid	NCBI_complete_genomes
E. coli	NZ_CP024832.1	Plasmid	NCBI_complete_genomes
E. coli	NZ_CP024833.1	Plasmid	NCBI_complete_genomes
E. coli	NZ_CP025329.1	Plasmid	NCBI_complete_genomes
E. coli	NZ_CP025402.1	Plasmid	NCBI_complete_genomes
E. coli	NZ_CP025403.1	Plasmid	NCBI_complete_genomes
E. coli	NZ_HG941719.1	Plasmid	NCBI_complete_genomes
E. coli	NZ_HG941720.1	Plasmid	NCBI_complete_genomes
E. coli	AB616660.2	Plasmid	Orlek_et_al.
E. coli	AB715422.2	Plasmid	Orlek_et_al.
E. coli	AE006471.2	Plasmid	Orlek_et_al.
E. coli	AJGY01000060.1	Plasmid	Orlek_et_al.
E. coli	AJGY01000090.1	Plasmid	Orlek_et_al.
E. coli	AP014611.1	Plasmid	Orlek_et_al.
E. coli	AP014654.2	Plasmid	Orlek_et_al.
E. coli	AP014804.1	Plasmid	Orlek_et_al.
E. coli	AP014805.1	Plasmid	Orlek_et_al.
E. coli	AP014806.1	Plasmid	Orlek_et_al.
E. coli	AP014807.1	Plasmid	Orlek_et_al.
E. coli	AP014939.1	Plasmid	Orlek_et_al.
E. coli	CM004293.1	Plasmid	Orlek_et_al.
E. coli	CP002127.1	Plasmid	Orlek_et_al.
E. coli	CP002128.1	Plasmid	Orlek_et_al.
E. coli	CP006737.1	Plasmid	Orlek_et_al.
E. coli	CP007449.1	Plasmid	Orlek_et_al.
E. coli	CP007450.1	Plasmid	Orlek_et_al.
E. coli	CP008701.1	Plasmid	Orlek_et_al.
E. coli	CP009231.1	Plasmid	Orlek_et_al.
E. coli	CP009232.1	Plasmid	Orlek_et_al.
E. coli	CP009233.1	Plasmid	Orlek_et_al.
E. coli	CP009410.2	Plasmid	Orlek_et_al.
E. coli	CP009411.2	Plasmid	Orlek_et_al.

E. coli	CP009412.2	Plasmid	Orlek_et_al.
E. coli	CP009413.2	Plasmid	Orlek_et_al.
E. coli	CP009414.2	Plasmid	Orlek_et_al.
E. coli	CP010513.1	Plasmid	Orlek_et_al.
E. coli	CP011658.1	Plasmid	Orlek_et_al.
E. coli	CP011999.1	Plasmid	Orlek_et_al.
E. coli	CP012000.1	Plasmid	Orlek_et_al.
E. coli	CP012197.1	Plasmid	Orlek_et_al.
E. coli	CP012198.1	Plasmid	Orlek_et_al.
E. coli	CP012902.1	Plasmid	Orlek_et_al.
E. coli	CP012903.1	Plasmid	Orlek_et_al.
E. coli	CP012904.1	Plasmid	Orlek_et_al.
E. coli	CP013215.1	Plasmid	Orlek_et_al.
E. coli	CP013836.1	Plasmid	Orlek_et_al.
E. coli	CP014776.1	Plasmid	Orlek_et_al.
E. coli	CP014777.1	Plasmid	Orlek_et_al.
E. coli	CP014778.1	Plasmid	Orlek_et_al.
E. coli	CP014779.1	Plasmid	Orlek_et_al.
E. coli	CP014962.1	Plasmid	Orlek_et_al.
E. coli	CP015086.1	Plasmid	Orlek_et_al.
E. coli	CP015977.1	Plasmid	Orlek_et_al.
E. coli	CP016815.1	Plasmid	Orlek_et_al.
E. coli	CP016864.1	Plasmid	Orlek_et_al.
E. coli	CP016865.1	Plasmid	Orlek_et_al.
E. coli	CP016866.1	Plasmid	Orlek_et_al.
E. coli	CP016867.1	Plasmid	Orlek_et_al.
E. coli	CP016890.1	Plasmid	Orlek_et_al.
E. coli	CP016891.1	Plasmid	Orlek_et_al.
E. coli	CP016892.1	Plasmid	Orlek_et_al.
E. coli	CP016919.1	Plasmid	Orlek_et_al.
E. coli	CP016920.1	Plasmid	Orlek_et_al.
E. coli	CP016921.1	Plasmid	Orlek_et_al.
E. coli	CP016922.1	Plasmid	Orlek_et_al.
E. coli	CP016924.1	Plasmid	Orlek_et_al.
E. coli	CP016925.1	Plasmid	Orlek_et_al.
E. coli	CP016927.1	Plasmid	Orlek_et_al.
E. coli	CP016932.1	Plasmid	Orlek_et_al.
E. coli	CP016933.1	Plasmid	Orlek_et_al.
E. coli	CP016934.1	Plasmid	Orlek_et_al.
E. coli	CP016936.1	Plasmid	Orlek_et_al.
E. coli	CP016938.1	Plasmid	Orlek_et_al.
E. coli	CP016939.1	Plasmid	Orlek_et_al.
E. coli	CP016941.1	Plasmid	Orlek_et_al.
E. coli	CP016942.1	Plasmid	Orlek_et_al.
E. coli	CP016943.1	Plasmid	Orlek_et_al.
E. coli	CP016946.1	Plasmid	Orlek_et_al.
E. coli	CP016947.1	Plasmid	Orlek_et_al.
E. coli	CU928146.1	Plasmid	Orlek_et_al.
E. coli	EF219134.3	Plasmid	Orlek_et_al.
E. coli	EU418923.1	Plasmid	Orlek_et_al.
E. coli	EU418926.1	Plasmid	Orlek_et_al.
E. coli	EU418931.1	Plasmid	Orlek_et_al.
E. coli	FN691998.1	Plasmid	Orlek_et_al.
E. coli	HF560649.1	Plasmid	Orlek_et_al.
E. coli	HF922624.1	Plasmid	Orlek_et_al.
E. coli	HF969016.1	Plasmid	Orlek_et_al.
E. coli	HG813238.1	Plasmid	Orlek_et_al.
E. coli	HG813239.1	Plasmid	Orlek_et_al.
E. coli	HG918041.1	Plasmid	Orlek_et_al.
E. coli	HG969995.1	Plasmid	Orlek_et_al.
E. coli	HG969996.1	Plasmid	Orlek_et_al.
E. coli	HG969997.1	Plasmid	Orlek_et_al.
E. coli	HG969998.1	Plasmid	Orlek_et_al.
E. coli	HG969999.1	Plasmid	Orlek_et_al.
E. coli	HG970000.1	Plasmid	Orlek_et_al.
E. coli	HG970001.1	Plasmid	Orlek_et_al.
E. coli	HM138194.1	Plasmid	Orlek_et_al.
E. coli	JMMY01000002.1	Plasmid	Orlek_et_al.
E. coli	JMSW01000002.1	Plasmid	Orlek_et_al.
E. coli	JMSW01000004.1	Plasmid	Orlek_et_al.
E. coli	JMSX01000002.1	Plasmid	Orlek_et_al.
E. coli	JMSX01000003.1	Plasmid	Orlek_et_al.

E. coli	JMSX01000004.1	Plasmid	Orlek_et_al.
E. coli	JMSX01000005.1	Plasmid	Orlek_et_al.
E. coli	JN194214.1	Plasmid	Orlek_et_al.
E. coli	JN233704.1	Plasmid	Orlek_et_al.
E. coli	JNBM01000002.1	Plasmid	Orlek_et_al.
E. coli	JNBM01000003.1	Plasmid	Orlek_et_al.
E. coli	JNBM01000005.1	Plasmid	Orlek_et_al.
E. coli	JX065631.1	Plasmid	Orlek_et_al.
E. coli	KC887916.2	Plasmid	Orlek_et_al.
E. coli	KC887917.2	Plasmid	Orlek_et_al.
E. coli	KC999035.4	Plasmid	Orlek_et_al.
E. coli	KF601686.2	Plasmid	Orlek_et_al.
E. coli	KF743817.1	Plasmid	Orlek_et_al.
E. coli	KF992018.2	Plasmid	Orlek_et_al.
E. coli	KJ020575.1	Plasmid	Orlek_et_al.
E. coli	KJ020576.1	Plasmid	Orlek_et_al.
E. coli	KJ158441.1	Plasmid	Orlek_et_al.
E. coli	KJ187750.1	Plasmid	Orlek_et_al.
E. coli	KJ187751.1	Plasmid	Orlek_et_al.
E. coli	KJ187752.1	Plasmid	Orlek_et_al.
E. coli	KJ190020.1	Plasmid	Orlek_et_al.
E. coli	KJ201886.1	Plasmid	Orlek_et_al.
E. coli	KJ201887.1	Plasmid	Orlek_et_al.
E. coli	KJ406378.1	Plasmid	Orlek_et_al.
E. coli	KJ440075.1	Plasmid	Orlek_et_al.
E. coli	KJ440076.1	Plasmid	Orlek_et_al.
E. coli	KJ460501.1	Plasmid	Orlek_et_al.
E. coli	KJ541068.1	Plasmid	Orlek_et_al.
E. coli	KJ541069.1	Plasmid	Orlek_et_al.
E. coli	KJ541070.1	Plasmid	Orlek_et_al.
E. coli	KJ541071.1	Plasmid	Orlek_et_al.
E. coli	KJ563250.1	Plasmid	Orlek_et_al.
E. coli	KJ577613.1	Plasmid	Orlek_et_al.
E. coli	KJ588779.1	Plasmid	Orlek_et_al.
E. coli	KJ653815.1	Plasmid	Orlek_et_al.
E. coli	KJ721789.1	Plasmid	Orlek_et_al.
E. coli	KJ721790.1	Plasmid	Orlek_et_al.
E. coli	KJ721805.1	Plasmid	Orlek_et_al.
E. coli	KJ802404.1	Plasmid	Orlek_et_al.
E. coli	KJ802405.1	Plasmid	Orlek_et_al.
E. coli	KJ812998.1	Plasmid	Orlek_et_al.
E. coli	KJ866866.1	Plasmid	Orlek_et_al.
E. coli	KJ958926.1	Plasmid	Orlek_et_al.
E. coli	KJ958927.1	Plasmid	Orlek_et_al.
E. coli	KM023153.1	Plasmid	Orlek_et_al.
E. coli	KM052220.1	Plasmid	Orlek_et_al.
E. coli	KM085449.1	Plasmid	Orlek_et_al.
E. coli	KM085450.1	Plasmid	Orlek_et_al.
E. coli	KM085451.1	Plasmid	Orlek_et_al.
E. coli	KM085452.1	Plasmid	Orlek_et_al.
E. coli	KM085453.1	Plasmid	Orlek_et_al.
E. coli	KM107838.1	Plasmid	Orlek_et_al.
E. coli	KM107839.1	Plasmid	Orlek_et_al.
E. coli	KM107840.1	Plasmid	Orlek_et_al.
E. coli	KM107841.1	Plasmid	Orlek_et_al.
E. coli	KM107842.1	Plasmid	Orlek_et_al.
E. coli	KM107843.1	Plasmid	Orlek_et_al.
E. coli	KM107844.1	Plasmid	Orlek_et_al.
E. coli	KM107845.1	Plasmid	Orlek_et_al.
E. coli	KM107846.1	Plasmid	Orlek_et_al.
E. coli	KM107847.1	Plasmid	Orlek_et_al.
E. coli	KM107848.1	Plasmid	Orlek_et_al.
E. coli	KM112087.1	Plasmid	Orlek_et_al.
E. coli	KM198330.1	Plasmid	Orlek_et_al.
E. coli	KM207012.2	Plasmid	Orlek_et_al.
E. coli	KM212169.1	Plasmid	Orlek_et_al.
E. coli	KM287568.1	Plasmid	Orlek_et_al.
E. coli	KM373703.1	Plasmid	Orlek_et_al.
E. coli	KM377238.1	Plasmid	Orlek_et_al.
E. coli	KM377239.1	Plasmid	Orlek_et_al.
E. coli	KM377240.1	Plasmid	Orlek_et_al.
E. coli	KM396298.1	Plasmid	Orlek_et_al.

E. coli	KM396299.1	Plasmid	Orlek_et_al.
E. coli	KM396300.1	Plasmid	Orlek_et_al.
E. coli	KM400601.1	Plasmid	Orlek_et_al.
E. coli	KM406488.1	Plasmid	Orlek_et_al.
E. coli	KM406489.1	Plasmid	Orlek_et_al.
E. coli	KM406490.1	Plasmid	Orlek_et_al.
E. coli	KM406491.1	Plasmid	Orlek_et_al.
E. coli	KM409652.1	Plasmid	Orlek_et_al.
E. coli	KM577619.1	Plasmid	Orlek_et_al.
E. coli	KM580532.1	Plasmid	Orlek_et_al.
E. coli	KM580533.1	Plasmid	Orlek_et_al.
E. coli	KM660724.1	Plasmid	Orlek_et_al.
E. coli	KM670336.1	Plasmid	Orlek_et_al.
E. coli	KM877269.1	Plasmid	Orlek_et_al.
E. coli	KM877517.1	Plasmid	Orlek_et_al.
E. coli	KM977631.1	Plasmid	Orlek_et_al.
E. coli	KP008371.1	Plasmid	Orlek_et_al.
E. coli	KP017243.1	Plasmid	Orlek_et_al.
E. coli	KP025948.1	Plasmid	Orlek_et_al.
E. coli	KP056256.1	Plasmid	Orlek_et_al.
E. coli	KP061858.1	Plasmid	Orlek_et_al.
E. coli	KP091735.1	Plasmid	Orlek_et_al.
E. coli	KP125892.1	Plasmid	Orlek_et_al.
E. coli	KP125893.1	Plasmid	Orlek_et_al.
E. coli	KP143090.1	Plasmid	Orlek_et_al.
E. coli	KP198615.1	Plasmid	Orlek_et_al.
E. coli	KP198616.1	Plasmid	Orlek_et_al.
E. coli	KP205272.1	Plasmid	Orlek_et_al.
E. coli	KP276584.1	Plasmid	Orlek_et_al.
E. coli	KP294351.1	Plasmid	Orlek_et_al.
E. coli	KP313759.1	Plasmid	Orlek_et_al.
E. coli	KP324830.1	Plasmid	Orlek_et_al.
E. coli	KP330456.1	Plasmid	Orlek_et_al.
E. coli	KP345882.1	Plasmid	Orlek_et_al.
E. coli	KP347127.1	Plasmid	Orlek_et_al.
E. coli	KP398867.1	Plasmid	Orlek_et_al.
E. coli	KP400525.1	Plasmid	Orlek_et_al.
E. coli	KP453775.1	Plasmid	Orlek_et_al.
E. coli	KP659188.1	Plasmid	Orlek_et_al.
E. coli	KP689347.1	Plasmid	Orlek_et_al.
E. coli	KP726894.1	Plasmid	Orlek_et_al.
E. coli	KP742988.1	Plasmid	Orlek_et_al.
E. coli	KP763470.1	Plasmid	Orlek_et_al.
E. coli	KP765744.1	Plasmid	Orlek_et_al.
E. coli	KP776609.1	Plasmid	Orlek_et_al.
E. coli	KP789019.1	Plasmid	Orlek_et_al.
E. coli	KP789020.1	Plasmid	Orlek_et_al.
E. coli	KP792123.1	Plasmid	Orlek_et_al.
E. coli	KP868646.1	Plasmid	Orlek_et_al.
E. coli	KP868647.1	Plasmid	Orlek_et_al.
E. coli	KP893385.1	Plasmid	Orlek_et_al.
E. coli	KP899803.1	Plasmid	Orlek_et_al.
E. coli	KP899804.1	Plasmid	Orlek_et_al.
E. coli	KP899805.1	Plasmid	Orlek_et_al.
E. coli	KP899806.1	Plasmid	Orlek_et_al.
E. coli	KP900015.1	Plasmid	Orlek_et_al.
E. coli	KP900016.1	Plasmid	Orlek_et_al.
E. coli	KP942676.1	Plasmid	Orlek_et_al.
E. coli	KP970685.1	Plasmid	Orlek_et_al.
E. coli	KP975074.1	Plasmid	Orlek_et_al.
E. coli	KP975075.1	Plasmid	Orlek_et_al.
E. coli	KP975077.1	Plasmid	Orlek_et_al.
E. coli	KP979588.1	Plasmid	Orlek_et_al.
E. coli	KP979589.1	Plasmid	Orlek_et_al.
E. coli	KP987215.1	Plasmid	Orlek_et_al.
E. coli	KP987216.1	Plasmid	Orlek_et_al.
E. coli	KP987217.1	Plasmid	Orlek_et_al.
E. coli	KP987218.1	Plasmid	Orlek_et_al.
E. coli	KR059864.1	Plasmid	Orlek_et_al.
E. coli	KR078259.1	Plasmid	Orlek_et_al.
E. coli	KR091911.1	Plasmid	Orlek_et_al.
E. coli	KR091915.1	Plasmid	Orlek_et_al.

E. coli	KR259130.1	Plasmid	Orlek_et_al.
E. coli	KR259131.1	Plasmid	Orlek_et_al.
E. coli	KR259132.1	Plasmid	Orlek_et_al.
E. coli	KR259133.1	Plasmid	Orlek_et_al.
E. coli	KR259134.1	Plasmid	Orlek_et_al.
E. coli	KR351290.1	Plasmid	Orlek_et_al.
E. coli	KR559888.1	Plasmid	Orlek_et_al.
E. coli	KR559889.1	Plasmid	Orlek_et_al.
E. coli	KR559890.1	Plasmid	Orlek_et_al.
E. coli	KR653209.1	Plasmid	Orlek_et_al.
E. coli	KR779901.1	Plasmid	Orlek_et_al.
E. coli	KR822246.1	Plasmid	Orlek_et_al.
E. coli	KR822247.1	Plasmid	Orlek_et_al.
E. coli	KR827684.1	Plasmid	Orlek_et_al.
E. coli	KR869777.1	Plasmid	Orlek_et_al.
E. coli	KR905384.1	Plasmid	Orlek_et_al.
E. coli	KR905385.1	Plasmid	Orlek_et_al.
E. coli	KR905386.1	Plasmid	Orlek_et_al.
E. coli	KR905387.1	Plasmid	Orlek_et_al.
E. coli	KR905388.1	Plasmid	Orlek_et_al.
E. coli	KR905389.1	Plasmid	Orlek_et_al.
E. coli	KR905390.1	Plasmid	Orlek_et_al.
E. coli	KT002541.1	Plasmid	Orlek_et_al.
E. coli	KT005457.1	Plasmid	Orlek_et_al.
E. coli	KT020860.1	Plasmid	Orlek_et_al.
E. coli	KT070138.1	Plasmid	Orlek_et_al.
E. coli	KT074362.1	Plasmid	Orlek_et_al.
E. coli	KT148595.1	Plasmid	Orlek_et_al.
E. coli	KT185451.1	Plasmid	Orlek_et_al.
E. coli	KT203286.1	Plasmid	Orlek_et_al.
E. coli	KT207463.1	Plasmid	Orlek_et_al.
E. coli	KT225462.1	Plasmid	Orlek_et_al.
E. coli	KT225520.1	Plasmid	Orlek_et_al.
E. coli	KT282968.1	Plasmid	Orlek_et_al.
E. coli	KT307967.1	Plasmid	Orlek_et_al.
E. coli	KT317611.1	Plasmid	Orlek_et_al.
E. coli	KT317612.1	Plasmid	Orlek_et_al.
E. coli	KT317613.1	Plasmid	Orlek_et_al.
E. coli	KT317614.1	Plasmid	Orlek_et_al.
E. coli	KT334335.1	Plasmid	Orlek_et_al.
E. coli	KT345945.1	Plasmid	Orlek_et_al.
E. coli	KT345946.1	Plasmid	Orlek_et_al.
E. coli	KT345947.1	Plasmid	Orlek_et_al.
E. coli	KT347600.1	Plasmid	Orlek_et_al.
E. coli	KT351734.1	Plasmid	Orlek_et_al.
E. coli	KT351735.1	Plasmid	Orlek_et_al.
E. coli	KT351736.1	Plasmid	Orlek_et_al.
E. coli	KT351737.1	Plasmid	Orlek_et_al.
E. coli	KT362706.1	Plasmid	Orlek_et_al.
E. coli	KT693143.1	Plasmid	Orlek_et_al.
E. coli	KT693144.1	Plasmid	Orlek_et_al.
E. coli	KT693145.1	Plasmid	Orlek_et_al.
E. coli	KT725788.1	Plasmid	Orlek_et_al.
E. coli	KT725789.1	Plasmid	Orlek_et_al.
E. coli	KT754160.1	Plasmid	Orlek_et_al.
E. coli	KT754161.1	Plasmid	Orlek_et_al.
E. coli	KT754162.1	Plasmid	Orlek_et_al.
E. coli	KT754163.1	Plasmid	Orlek_et_al.
E. coli	KT754164.1	Plasmid	Orlek_et_al.
E. coli	KT754165.1	Plasmid	Orlek_et_al.
E. coli	KT754166.1	Plasmid	Orlek_et_al.
E. coli	KT754167.1	Plasmid	Orlek_et_al.
E. coli	KT779550.1	Plasmid	Orlek_et_al.
E. coli	KT818627.1	Plasmid	Orlek_et_al.
E. coli	KT824791.1	Plasmid	Orlek_et_al.
E. coli	KT868530.1	Plasmid	Orlek_et_al.
E. coli	KT879914.1	Plasmid	Orlek_et_al.
E. coli	KT896499.1	Plasmid	Orlek_et_al.
E. coli	KT896500.1	Plasmid	Orlek_et_al.
E. coli	KT896501.1	Plasmid	Orlek_et_al.
E. coli	KT896502.1	Plasmid	Orlek_et_al.
E. coli	KT896503.1	Plasmid	Orlek_et_al.

E. coli	KT935445.1	Plasmid	Orlek_et_al.
E. coli	KT935446.1	Plasmid	Orlek_et_al.
E. coli	KT937280.1	Plasmid	Orlek_et_al.
E. coli	KT937281.1	Plasmid	Orlek_et_al.
E. coli	KT937282.1	Plasmid	Orlek_et_al.
E. coli	KT950740.1	Plasmid	Orlek_et_al.
E. coli	KT950741.1	Plasmid	Orlek_et_al.
E. coli	KT982613.1	Plasmid	Orlek_et_al.
E. coli	KT982614.1	Plasmid	Orlek_et_al.
E. coli	KT982616.1	Plasmid	Orlek_et_al.
E. coli	KT982617.1	Plasmid	Orlek_et_al.
E. coli	KT982618.1	Plasmid	Orlek_et_al.
E. coli	KT988018.1	Plasmid	Orlek_et_al.
E. coli	KT988019.1	Plasmid	Orlek_et_al.
E. coli	KT988020.1	Plasmid	Orlek_et_al.
E. coli	KT988306.1	Plasmid	Orlek_et_al.
E. coli	KT989598.1	Plasmid	Orlek_et_al.
E. coli	KT989599.1	Plasmid	Orlek_et_al.
E. coli	KT990220.1	Plasmid	Orlek_et_al.
E. coli	KU051707.1	Plasmid	Orlek_et_al.
E. coli	KU051708.1	Plasmid	Orlek_et_al.
E. coli	KU051709.1	Plasmid	Orlek_et_al.
E. coli	KU051710.1	Plasmid	Orlek_et_al.
E. coli	KU130396.1	Plasmid	Orlek_et_al.
E. coli	KU159085.1	Plasmid	Orlek_et_al.
E. coli	KU159086.1	Plasmid	Orlek_et_al.
E. coli	KU160530.1	Plasmid	Orlek_et_al.
E. coli	KU166868.1	Plasmid	Orlek_et_al.
E. coli	KU176944.1	Plasmid	Orlek_et_al.
E. coli	KU254578.1	Plasmid	Orlek_et_al.
E. coli	KU254579.1	Plasmid	Orlek_et_al.
E. coli	KU254580.1	Plasmid	Orlek_et_al.
E. coli	KU254581.1	Plasmid	Orlek_et_al.
E. coli	KU288634.1	Plasmid	Orlek_et_al.
E. coli	KU295131.1	Plasmid	Orlek_et_al.
E. coli	KU295132.1	Plasmid	Orlek_et_al.
E. coli	KU295133.1	Plasmid	Orlek_et_al.
E. coli	KU295134.1	Plasmid	Orlek_et_al.
E. coli	KU295135.1	Plasmid	Orlek_et_al.
E. coli	KU295136.1	Plasmid	Orlek_et_al.
E. coli	KU302800.1	Plasmid	Orlek_et_al.
E. coli	KU302801.1	Plasmid	Orlek_et_al.
E. coli	KU302802.1	Plasmid	Orlek_et_al.
E. coli	KU302803.1	Plasmid	Orlek_et_al.
E. coli	KU302804.1	Plasmid	Orlek_et_al.
E. coli	KU302805.1	Plasmid	Orlek_et_al.
E. coli	KU302806.1	Plasmid	Orlek_et_al.
E. coli	KU302807.1	Plasmid	Orlek_et_al.
E. coli	KU302808.1	Plasmid	Orlek_et_al.
E. coli	KU302809.1	Plasmid	Orlek_et_al.
E. coli	KU312044.1	Plasmid	Orlek_et_al.
E. coli	KU314941.1	Plasmid	Orlek_et_al.
E. coli	KU315015.1	Plasmid	Orlek_et_al.
E. coli	KU318419.1	Plasmid	Orlek_et_al.
E. coli	KU318420.1	Plasmid	Orlek_et_al.
E. coli	KU318421.1	Plasmid	Orlek_et_al.
E. coli	KU341381.1	Plasmid	Orlek_et_al.
E. coli	KU353730.1	Plasmid	Orlek_et_al.
E. coli	KU355873.1	Plasmid	Orlek_et_al.
E. coli	KU355874.1	Plasmid	Orlek_et_al.
E. coli	KU665641.1	Plasmid	Orlek_et_al.
E. coli	KU665642.1	Plasmid	Orlek_et_al.
E. coli	KU674895.1	Plasmid	Orlek_et_al.
E. coli	KU726092.1	Plasmid	Orlek_et_al.
E. coli	KU726616.1	Plasmid	Orlek_et_al.
E. coli	KU743383.1	Plasmid	Orlek_et_al.
E. coli	KU743384.1	Plasmid	Orlek_et_al.
E. coli	KU761326.1	Plasmid	Orlek_et_al.
E. coli	KU761327.1	Plasmid	Orlek_et_al.
E. coli	KU761328.1	Plasmid	Orlek_et_al.
E. coli	KU870627.1	Plasmid	Orlek_et_al.
E. coli	KU886034.1	Plasmid	Orlek_et_al.

E. coli	KU922754.1	Plasmid	Orlek_et_al.
E. coli	KU932021.1	Plasmid	Orlek_et_al.
E. coli	KU932022.1	Plasmid	Orlek_et_al.
E. coli	KU932023.1	Plasmid	Orlek_et_al.
E. coli	KU932024.1	Plasmid	Orlek_et_al.
E. coli	KU932025.1	Plasmid	Orlek_et_al.
E. coli	KU932026.1	Plasmid	Orlek_et_al.
E. coli	KU932027.1	Plasmid	Orlek_et_al.
E. coli	KU932028.1	Plasmid	Orlek_et_al.
E. coli	KU932029.1	Plasmid	Orlek_et_al.
E. coli	KU932030.1	Plasmid	Orlek_et_al.
E. coli	KU932031.1	Plasmid	Orlek_et_al.
E. coli	KU932032.1	Plasmid	Orlek_et_al.
E. coli	KU932033.1	Plasmid	Orlek_et_al.
E. coli	KU932034.1	Plasmid	Orlek_et_al.
E. coli	KU934011.1	Plasmid	Orlek_et_al.
E. coli	KU934209.1	Plasmid	Orlek_et_al.
E. coli	KU963389.1	Plasmid	Orlek_et_al.
E. coli	KU963390.1	Plasmid	Orlek_et_al.
E. coli	KU980950.1	Plasmid	Orlek_et_al.
E. coli	KU987452.1	Plasmid	Orlek_et_al.
E. coli	KU987453.1	Plasmid	Orlek_et_al.
E. coli	KU994859.1	Plasmid	Orlek_et_al.
E. coli	KU997026.1	Plasmid	Orlek_et_al.
E. coli	KX009507.1	Plasmid	Orlek_et_al.
E. coli	KX013538.1	Plasmid	Orlek_et_al.
E. coli	KX013539.1	Plasmid	Orlek_et_al.
E. coli	KX013540.1	Plasmid	Orlek_et_al.
E. coli	KX015668.1	Plasmid	Orlek_et_al.
E. coli	KX032519.1	Plasmid	Orlek_et_al.
E. coli	KX032520.1	Plasmid	Orlek_et_al.
E. coli	KX034083.1	Plasmid	Orlek_et_al.
E. coli	KX084392.1	Plasmid	Orlek_et_al.
E. coli	KX084393.1	Plasmid	Orlek_et_al.
E. coli	KX084394.1	Plasmid	Orlek_et_al.
E. coli	KX118608.1	Plasmid	Orlek_et_al.
E. coli	KX129782.1	Plasmid	Orlek_et_al.
E. coli	KX129783.1	Plasmid	Orlek_et_al.
E. coli	KX129784.1	Plasmid	Orlek_et_al.
E. coli	KX156772.1	Plasmid	Orlek_et_al.
E. coli	KX156773.1	Plasmid	Orlek_et_al.
E. coli	KX230795.1	Plasmid	Orlek_et_al.
E. coli	KX236178.1	Plasmid	Orlek_et_al.
E. coli	KX236309.1	Plasmid	Orlek_et_al.
E. coli	KX244760.1	Plasmid	Orlek_et_al.
E. coli	KX276657.1	Plasmid	Orlek_et_al.
E. coli	KX443694.1	Plasmid	Orlek_et_al.
E. coli	KX447767.1	Plasmid	Orlek_et_al.
E. coli	KX447768.1	Plasmid	Orlek_et_al.
E. coli	KX528699.1	Plasmid	Orlek_et_al.
E. coli	LC019731.1	Plasmid	Orlek_et_al.
E. coli	LC055503.1	Plasmid	Orlek_et_al.
E. coli	LC155908.1	Plasmid	Orlek_et_al.
E. coli	LC155909.1	Plasmid	Orlek_et_al.
E. coli	LK391770.1	Plasmid	Orlek_et_al.
E. coli	LN610760.1	Plasmid	Orlek_et_al.
E. coli	LN623683.2	Plasmid	Orlek_et_al.
E. coli	LN624486.1	Plasmid	Orlek_et_al.
E. coli	LN735558.1	Plasmid	Orlek_et_al.
E. coli	LN735559.1	Plasmid	Orlek_et_al.
E. coli	LN735560.1	Plasmid	Orlek_et_al.
E. coli	LN735561.1	Plasmid	Orlek_et_al.
E. coli	LN794247.1	Plasmid	Orlek_et_al.
E. coli	LN794248.1	Plasmid	Orlek_et_al.
E. coli	LN831046.1	Plasmid	Orlek_et_al.
E. coli	LN854558.1	Plasmid	Orlek_et_al.
E. coli	LN854559.1	Plasmid	Orlek_et_al.
E. coli	LN854560.1	Plasmid	Orlek_et_al.
E. coli	LN864819.1	Plasmid	Orlek_et_al.
E. coli	LN864820.1	Plasmid	Orlek_et_al.
E. coli	LN864821.1	Plasmid	Orlek_et_al.
E. coli	LN890286.1	Plasmid	Orlek_et_al.

E. coli	LN897474.2	Plasmid	Orlek_et_al.
E. coli	LN897475.2	Plasmid	Orlek_et_al.
E. coli	LO017736.1	Plasmid	Orlek_et_al.
E. coli	LO017737.1	Plasmid	Orlek_et_al.
E. coli	LO017738.1	Plasmid	Orlek_et_al.
E. coli	LT009688.1	Plasmid	Orlek_et_al.
E. coli	LT009689.1	Plasmid	Orlek_et_al.
E. coli	LT174531.1	Plasmid	Orlek_et_al.
E. coli	LT575491.1	Plasmid	Orlek_et_al.
E. coli	LT575492.1	Plasmid	Orlek_et_al.
E. coli	NC_001371.1	Plasmid	Orlek_et_al.
E. coli	NC_001373.1	Plasmid	Orlek_et_al.
E. coli	NC_001378.1	Plasmid	Orlek_et_al.
E. coli	NC_001537.1	Plasmid	Orlek_et_al.
E. coli	NC_001735.4	Plasmid	Orlek_et_al.
E. coli	NC_001740.1	Plasmid	Orlek_et_al.
E. coli	NC_001848.1	Plasmid	Orlek_et_al.
E. coli	NC_001898.1	Plasmid	Orlek_et_al.
E. coli	NC_001910.1	Plasmid	Orlek_et_al.
E. coli	NC_001911.1	Plasmid	Orlek_et_al.
E. coli	NC_002056.1	Plasmid	Orlek_et_al.
E. coli	NC_002070.1	Plasmid	Orlek_et_al.
E. coli	NC_002090.1	Plasmid	Orlek_et_al.
E. coli	NC_002119.1	Plasmid	Orlek_et_al.
E. coli	NC_002120.1	Plasmid	Orlek_et_al.
E. coli	NC_002122.1	Plasmid	Orlek_et_al.
E. coli	NC_002127.1	Plasmid	Orlek_et_al.
E. coli	NC_002128.1	Plasmid	Orlek_et_al.
E. coli	NC_002134.1	Plasmid	Orlek_et_al.
E. coli	NC_002142.1	Plasmid	Orlek_et_al.
E. coli	NC_002144.1	Plasmid	Orlek_et_al.
E. coli	NC_002145.1	Plasmid	Orlek_et_al.
E. coli	NC_002252.1	Plasmid	Orlek_et_al.
E. coli	NC_002253.1	Plasmid	Orlek_et_al.
E. coli	NC_002305.1	Plasmid	Orlek_et_al.
E. coli	NC_002483.1	Plasmid	Orlek_et_al.
E. coli	NC_002487.1	Plasmid	Orlek_et_al.
E. coli	NC_002497.1	Plasmid	Orlek_et_al.
E. coli	NC_002498.1	Plasmid	Orlek_et_al.
E. coli	NC_002523.4	Plasmid	Orlek_et_al.
E. coli	NC_002525.1	Plasmid	Orlek_et_al.
E. coli	NC_002610.1	Plasmid	Orlek_et_al.
E. coli	NC_002632.1	Plasmid	Orlek_et_al.
E. coli	NC_002638.1	Plasmid	Orlek_et_al.
E. coli	NC_002698.1	Plasmid	Orlek_et_al.
E. coli	NC_002773.1	Plasmid	Orlek_et_al.
E. coli	NC_002809.1	Plasmid	Orlek_et_al.
E. coli	NC_003079.1	Plasmid	Orlek_et_al.
E. coli	NC_003114.1	Plasmid	Orlek_et_al.
E. coli	NC_003131.1	Plasmid	Orlek_et_al.
E. coli	NC_003132.1	Plasmid	Orlek_et_al.
E. coli	NC_003134.1	Plasmid	Orlek_et_al.
E. coli	NC_003277.1	Plasmid	Orlek_et_al.
E. coli	NC_003292.1	Plasmid	Orlek_et_al.
E. coli	NC_003384.1	Plasmid	Orlek_et_al.
E. coli	NC_003385.1	Plasmid	Orlek_et_al.
E. coli	NC_003425.1	Plasmid	Orlek_et_al.
E. coli	NC_003455.1	Plasmid	Orlek_et_al.
E. coli	NC_003456.1	Plasmid	Orlek_et_al.
E. coli	NC_003457.1	Plasmid	Orlek_et_al.
E. coli	NC_003486.1	Plasmid	Orlek_et_al.
E. coli	NC_003789.1	Plasmid	Orlek_et_al.
E. coli	NC_003905.1	Plasmid	Orlek_et_al.
E. coli	NC_004429.1	Plasmid	Orlek_et_al.
E. coli	NC_004445.1	Plasmid	Orlek_et_al.
E. coli	NC_004446.1	Plasmid	Orlek_et_al.
E. coli	NC_004464.2	Plasmid	Orlek_et_al.
E. coli	NC_004555.1	Plasmid	Orlek_et_al.
E. coli	NC_004564.1	Plasmid	Orlek_et_al.
E. coli	NC_004834.1	Plasmid	Orlek_et_al.
E. coli	NC_004835.1	Plasmid	Orlek_et_al.
E. coli	NC_004836.1	Plasmid	Orlek_et_al.

E. coli	NC_004837.1	Plasmid	Orlek_et_al.
E. coli	NC_004838.1	Plasmid	Orlek_et_al.
E. coli	NC_004839.1	Plasmid	Orlek_et_al.
E. coli	NC_004843.1	Plasmid	Orlek_et_al.
E. coli	NC_004851.1	Plasmid	Orlek_et_al.
E. coli	NC_004936.1	Plasmid	Orlek_et_al.
E. coli	NC_004940.1	Plasmid	Orlek_et_al.
E. coli	NC_004989.1	Plasmid	Orlek_et_al.
E. coli	NC_004998.1	Plasmid	Orlek_et_al.
E. coli	NC_005002.1	Plasmid	Orlek_et_al.
E. coli	NC_005014.1	Plasmid	Orlek_et_al.
E. coli	NC_005015.1	Plasmid	Orlek_et_al.
E. coli	NC_005017.1	Plasmid	Orlek_et_al.
E. coli	NC_005018.1	Plasmid	Orlek_et_al.
E. coli	NC_005019.1	Plasmid	Orlek_et_al.
E. coli	NC_005211.1	Plasmid	Orlek_et_al.
E. coli	NC_005246.1	Plasmid	Orlek_et_al.
E. coli	NC_005247.1	Plasmid	Orlek_et_al.
E. coli	NC_005248.1	Plasmid	Orlek_et_al.
E. coli	NC_005249.1	Plasmid	Orlek_et_al.
E. coli	NC_005324.1	Plasmid	Orlek_et_al.
E. coli	NC_005327.1	Plasmid	Orlek_et_al.
E. coli	NC_005570.1	Plasmid	Orlek_et_al.
E. coli	NC_005706.1	Plasmid	Orlek_et_al.
E. coli	NC_005813.1	Plasmid	Orlek_et_al.
E. coli	NC_005814.1	Plasmid	Orlek_et_al.
E. coli	NC_005815.1	Plasmid	Orlek_et_al.
E. coli	NC_005816.1	Plasmid	Orlek_et_al.
E. coli	NC_005862.1	Plasmid	Orlek_et_al.
E. coli	NC_005910.1	Plasmid	Orlek_et_al.
E. coli	NC_005911.1	Plasmid	Orlek_et_al.
E. coli	NC_005923.1	Plasmid	Orlek_et_al.
E. coli	NC_005970.1	Plasmid	Orlek_et_al.
E. coli	NC_006153.2	Plasmid	Orlek_et_al.
E. coli	NC_006154.1	Plasmid	Orlek_et_al.
E. coli	NC_006323.1	Plasmid	Orlek_et_al.
E. coli	NC_006625.1	Plasmid	Orlek_et_al.
E. coli	NC_006671.1	Plasmid	Orlek_et_al.
E. coli	NC_006815.1	Plasmid	Orlek_et_al.
E. coli	NC_006816.1	Plasmid	Orlek_et_al.
E. coli	NC_006855.1	Plasmid	Orlek_et_al.
E. coli	NC_006856.1	Plasmid	Orlek_et_al.
E. coli	NC_006881.1	Plasmid	Orlek_et_al.
E. coli	NC_007182.1	Plasmid	Orlek_et_al.
E. coli	NC_007183.1	Plasmid	Orlek_et_al.
E. coli	NC_007184.1	Plasmid	Orlek_et_al.
E. coli	NC_007185.1	Plasmid	Orlek_et_al.
E. coli	NC_007186.1	Plasmid	Orlek_et_al.
E. coli	NC_007187.1	Plasmid	Orlek_et_al.
E. coli	NC_007208.1	Plasmid	Orlek_et_al.
E. coli	NC_007365.1	Plasmid	Orlek_et_al.
E. coli	NC_007385.1	Plasmid	Orlek_et_al.
E. coli	NC_007414.1	Plasmid	Orlek_et_al.
E. coli	NC_007607.1	Plasmid	Orlek_et_al.
E. coli	NC_007608.1	Plasmid	Orlek_et_al.
E. coli	NC_007635.1	Plasmid	Orlek_et_al.
E. coli	NC_007675.1	Plasmid	Orlek_et_al.
E. coli	NC_007682.3	Plasmid	Orlek_et_al.
E. coli	NC_007713.1	Plasmid	Orlek_et_al.
E. coli	NC_007714.1	Plasmid	Orlek_et_al.
E. coli	NC_007715.1	Plasmid	Orlek_et_al.
E. coli	NC_007941.1	Plasmid	Orlek_et_al.
E. coli	NC_008053.1	Plasmid	Orlek_et_al.
E. coli	NC_008118.1	Plasmid	Orlek_et_al.
E. coli	NC_008119.1	Plasmid	Orlek_et_al.
E. coli	NC_008120.1	Plasmid	Orlek_et_al.
E. coli	NC_008121.1	Plasmid	Orlek_et_al.
E. coli	NC_008122.1	Plasmid	Orlek_et_al.
E. coli	NC_008439.1	Plasmid	Orlek_et_al.
E. coli	NC_008444.1	Plasmid	Orlek_et_al.
E. coli	NC_008460.1	Plasmid	Orlek_et_al.
E. coli	NC_008486.1	Plasmid	Orlek_et_al.

E. coli	NC_008487.1	Plasmid	Orlek_et_al.
E. coli	NC_008488.1	Plasmid	Orlek_et_al.
E. coli	NC_008489.1	Plasmid	Orlek_et_al.
E. coli	NC_008490.1	Plasmid	Orlek_et_al.
E. coli	NC_008597.1	Plasmid	Orlek_et_al.
E. coli	NC_008791.1	Plasmid	Orlek_et_al.
E. coli	NC_009132.1	Plasmid	Orlek_et_al.
E. coli	NC_009133.1	Plasmid	Orlek_et_al.
E. coli	NC_009139.1	Plasmid	Orlek_et_al.
E. coli	NC_009140.1	Plasmid	Orlek_et_al.
E. coli	NC_009141.1	Plasmid	Orlek_et_al.
E. coli	NC_009344.1	Plasmid	Orlek_et_al.
E. coli	NC_009345.1	Plasmid	Orlek_et_al.
E. coli	NC_009346.1	Plasmid	Orlek_et_al.
E. coli	NC_009347.1	Plasmid	Orlek_et_al.
E. coli	NC_009377.1	Plasmid	Orlek_et_al.
E. coli	NC_009378.1	Plasmid	Orlek_et_al.
E. coli	NC_009425.1	Plasmid	Orlek_et_al.
E. coli	NC_009595.1	Plasmid	Orlek_et_al.
E. coli	NC_009596.1	Plasmid	Orlek_et_al.
E. coli	NC_009602.1	Plasmid	Orlek_et_al.
E. coli	NC_009649.1	Plasmid	Orlek_et_al.
E. coli	NC_009650.1	Plasmid	Orlek_et_al.
E. coli	NC_009651.1	Plasmid	Orlek_et_al.
E. coli	NC_009652.1	Plasmid	Orlek_et_al.
E. coli	NC_009653.1	Plasmid	Orlek_et_al.
E. coli	NC_009704.1	Plasmid	Orlek_et_al.
E. coli	NC_009705.1	Plasmid	Orlek_et_al.
E. coli	NC_009716.1	Plasmid	Orlek_et_al.
E. coli	NC_009779.1	Plasmid	Orlek_et_al.
E. coli	NC_009780.1	Plasmid	Orlek_et_al.
E. coli	NC_009781.1	Plasmid	Orlek_et_al.
E. coli	NC_009786.1	Plasmid	Orlek_et_al.
E. coli	NC_009787.1	Plasmid	Orlek_et_al.
E. coli	NC_009788.1	Plasmid	Orlek_et_al.
E. coli	NC_009789.1	Plasmid	Orlek_et_al.
E. coli	NC_009790.1	Plasmid	Orlek_et_al.
E. coli	NC_009791.1	Plasmid	Orlek_et_al.
E. coli	NC_009793.1	Plasmid	Orlek_et_al.
E. coli	NC_009794.1	Plasmid	Orlek_et_al.
E. coli	NC_009807.1	Plasmid	Orlek_et_al.
E. coli	NC_009829.1	Plasmid	Orlek_et_al.
E. coli	NC_009837.1	Plasmid	Orlek_et_al.
E. coli	NC_009838.1	Plasmid	Orlek_et_al.
E. coli	NC_009980.1	Plasmid	Orlek_et_al.
E. coli	NC_009981.1	Plasmid	Orlek_et_al.
E. coli	NC_009982.1	Plasmid	Orlek_et_al.
E. coli	NC_010064.1	Plasmid	Orlek_et_al.
E. coli	NC_010119.1	Plasmid	Orlek_et_al.
E. coli	NC_010157.1	Plasmid	Orlek_et_al.
E. coli	NC_010158.1	Plasmid	Orlek_et_al.
E. coli	NC_010257.1	Plasmid	Orlek_et_al.
E. coli	NC_010259.1	Plasmid	Orlek_et_al.
E. coli	NC_010261.1	Plasmid	Orlek_et_al.
E. coli	NC_010377.1	Plasmid	Orlek_et_al.
E. coli	NC_010378.1	Plasmid	Orlek_et_al.
E. coli	NC_010409.1	Plasmid	Orlek_et_al.
E. coli	NC_010421.1	Plasmid	Orlek_et_al.
E. coli	NC_010422.1	Plasmid	Orlek_et_al.
E. coli	NC_010485.1	Plasmid	Orlek_et_al.
E. coli	NC_010486.1	Plasmid	Orlek_et_al.
E. coli	NC_010487.1	Plasmid	Orlek_et_al.
E. coli	NC_010488.1	Plasmid	Orlek_et_al.
E. coli	NC_010499.1	Plasmid	Orlek_et_al.
E. coli	NC_010500.1	Plasmid	Orlek_et_al.
E. coli	NC_010555.1	Plasmid	Orlek_et_al.
E. coli	NC_010558.1	Plasmid	Orlek_et_al.
E. coli	NC_010635.1	Plasmid	Orlek_et_al.
E. coli	NC_010643.1	Plasmid	Orlek_et_al.
E. coli	NC_010656.1	Plasmid	Orlek_et_al.
E. coli	NC_010657.1	Plasmid	Orlek_et_al.
E. coli	NC_010659.1	Plasmid	Orlek_et_al.

E. coli	NC_010660.1	Plasmid	Orlek_et_al.
E. coli	NC_010672.1	Plasmid	Orlek_et_al.
E. coli	NC_010693.1	Plasmid	Orlek_et_al.
E. coli	NC_010695.1	Plasmid	Orlek_et_al.
E. coli	NC_010696.1	Plasmid	Orlek_et_al.
E. coli	NC_010697.1	Plasmid	Orlek_et_al.
E. coli	NC_010699.1	Plasmid	Orlek_et_al.
E. coli	NC_010716.1	Plasmid	Orlek_et_al.
E. coli	NC_010719.1	Plasmid	Orlek_et_al.
E. coli	NC_010720.1	Plasmid	Orlek_et_al.
E. coli	NC_010726.1	Plasmid	Orlek_et_al.
E. coli	NC_010796.1	Plasmid	Orlek_et_al.
E. coli	NC_010860.1	Plasmid	Orlek_et_al.
E. coli	NC_010862.1	Plasmid	Orlek_et_al.
E. coli	NC_010870.1	Plasmid	Orlek_et_al.
E. coli	NC_010883.1	Plasmid	Orlek_et_al.
E. coli	NC_010885.1	Plasmid	Orlek_et_al.
E. coli	NC_010886.1	Plasmid	Orlek_et_al.
E. coli	NC_010894.1	Plasmid	Orlek_et_al.
E. coli	NC_010896.1	Plasmid	Orlek_et_al.
E. coli	NC_010898.1	Plasmid	Orlek_et_al.
E. coli	NC_010904.1	Plasmid	Orlek_et_al.
E. coli	NC_010993.1	Plasmid	Orlek_et_al.
E. coli	NC_011076.1	Plasmid	Orlek_et_al.
E. coli	NC_011077.1	Plasmid	Orlek_et_al.
E. coli	NC_011078.1	Plasmid	Orlek_et_al.
E. coli	NC_011079.1	Plasmid	Orlek_et_al.
E. coli	NC_011081.1	Plasmid	Orlek_et_al.
E. coli	NC_011082.1	Plasmid	Orlek_et_al.
E. coli	NC_011092.1	Plasmid	Orlek_et_al.
E. coli	NC_011093.1	Plasmid	Orlek_et_al.
E. coli	NC_011148.1	Plasmid	Orlek_et_al.
E. coli	NC_011204.1	Plasmid	Orlek_et_al.
E. coli	NC_011214.1	Plasmid	Orlek_et_al.
E. coli	NC_011215.1	Plasmid	Orlek_et_al.
E. coli	NC_011227.1	Plasmid	Orlek_et_al.
E. coli	NC_011228.1	Plasmid	Orlek_et_al.
E. coli	NC_011266.1	Plasmid	Orlek_et_al.
E. coli	NC_011281.1	Plasmid	Orlek_et_al.
E. coli	NC_011282.1	Plasmid	Orlek_et_al.
E. coli	NC_011350.1	Plasmid	Orlek_et_al.
E. coli	NC_011351.1	Plasmid	Orlek_et_al.
E. coli	NC_011382.1	Plasmid	Orlek_et_al.
E. coli	NC_011383.1	Plasmid	Orlek_et_al.
E. coli	NC_011385.1	Plasmid	Orlek_et_al.
E. coli	NC_011404.1	Plasmid	Orlek_et_al.
E. coli	NC_011405.1	Plasmid	Orlek_et_al.
E. coli	NC_011406.1	Plasmid	Orlek_et_al.
E. coli	NC_011407.1	Plasmid	Orlek_et_al.
E. coli	NC_011408.1	Plasmid	Orlek_et_al.
E. coli	NC_011411.1	Plasmid	Orlek_et_al.
E. coli	NC_011413.1	Plasmid	Orlek_et_al.
E. coli	NC_011416.1	Plasmid	Orlek_et_al.
E. coli	NC_011418.1	Plasmid	Orlek_et_al.
E. coli	NC_011419.1	Plasmid	Orlek_et_al.
E. coli	NC_011422.1	Plasmid	Orlek_et_al.
E. coli	NC_011511.1	Plasmid	Orlek_et_al.
E. coli	NC_011512.1	Plasmid	Orlek_et_al.
E. coli	NC_011513.1	Plasmid	Orlek_et_al.
E. coli	NC_011514.1	Plasmid	Orlek_et_al.
E. coli	NC_011604.1	Plasmid	Orlek_et_al.
E. coli	NC_011617.1	Plasmid	Orlek_et_al.
E. coli	NC_011640.1	Plasmid	Orlek_et_al.
E. coli	NC_011641.1	Plasmid	Orlek_et_al.
E. coli	NC_011739.1	Plasmid	Orlek_et_al.
E. coli	NC_011743.1	Plasmid	Orlek_et_al.
E. coli	NC_011749.1	Plasmid	Orlek_et_al.
E. coli	NC_011752.1	Plasmid	Orlek_et_al.
E. coli	NC_011754.1	Plasmid	Orlek_et_al.
E. coli	NC_011767.1	Plasmid	Orlek_et_al.
E. coli	NC_011795.1	Plasmid	Orlek_et_al.
E. coli	NC_011799.1	Plasmid	Orlek_et_al.

E. coli	NC_011812.1	Plasmid	Orlek_et_al.
E. coli	NC_011878.1	Plasmid	Orlek_et_al.
E. coli	NC_011897.1	Plasmid	Orlek_et_al.
E. coli	NC_011917.1	Plasmid	Orlek_et_al.
E. coli	NC_011964.1	Plasmid	Orlek_et_al.
E. coli	NC_011977.1	Plasmid	Orlek_et_al.
E. coli	NC_011980.1	Plasmid	Orlek_et_al.
E. coli	NC_012006.1	Plasmid	Orlek_et_al.
E. coli	NC_012124.1	Plasmid	Orlek_et_al.
E. coli	NC_012208.1	Plasmid	Orlek_et_al.
E. coli	NC_012209.1	Plasmid	Orlek_et_al.
E. coli	NC_012487.1	Plasmid	Orlek_et_al.
E. coli	NC_012555.1	Plasmid	Orlek_et_al.
E. coli	NC_012556.1	Plasmid	Orlek_et_al.
E. coli	NC_012690.1	Plasmid	Orlek_et_al.
E. coli	NC_012692.1	Plasmid	Orlek_et_al.
E. coli	NC_012693.1	Plasmid	Orlek_et_al.
E. coli	NC_012752.1	Plasmid	Orlek_et_al.
E. coli	NC_012882.1	Plasmid	Orlek_et_al.
E. coli	NC_012886.1	Plasmid	Orlek_et_al.
E. coli	NC_012916.1	Plasmid	Orlek_et_al.
E. coli	NC_012944.1	Plasmid	Orlek_et_al.
E. coli	NC_012961.1	Plasmid	Orlek_et_al.
E. coli	NC_013010.1	Plasmid	Orlek_et_al.
E. coli	NC_013104.1	Plasmid	Orlek_et_al.
E. coli	NC_013120.1	Plasmid	Orlek_et_al.
E. coli	NC_013121.1	Plasmid	Orlek_et_al.
E. coli	NC_013122.1	Plasmid	Orlek_et_al.
E. coli	NC_013175.1	Plasmid	Orlek_et_al.
E. coli	NC_013263.1	Plasmid	Orlek_et_al.
E. coli	NC_013264.1	Plasmid	Orlek_et_al.
E. coli	NC_013265.1	Plasmid	Orlek_et_al.
E. coli	NC_013283.1	Plasmid	Orlek_et_al.
E. coli	NC_013284.1	Plasmid	Orlek_et_al.
E. coli	NC_013285.1	Plasmid	Orlek_et_al.
E. coli	NC_013354.1	Plasmid	Orlek_et_al.
E. coli	NC_013362.1	Plasmid	Orlek_et_al.
E. coli	NC_013363.1	Plasmid	Orlek_et_al.
E. coli	NC_013365.1	Plasmid	Orlek_et_al.
E. coli	NC_013366.1	Plasmid	Orlek_et_al.
E. coli	NC_013367.1	Plasmid	Orlek_et_al.
E. coli	NC_013368.1	Plasmid	Orlek_et_al.
E. coli	NC_013369.1	Plasmid	Orlek_et_al.
E. coli	NC_013370.1	Plasmid	Orlek_et_al.
E. coli	NC_013437.1	Plasmid	Orlek_et_al.
E. coli	NC_013503.1	Plasmid	Orlek_et_al.
E. coli	NC_013507.1	Plasmid	Orlek_et_al.
E. coli	NC_013509.1	Plasmid	Orlek_et_al.
E. coli	NC_013542.1	Plasmid	Orlek_et_al.
E. coli	NC_013549.1	Plasmid	Orlek_et_al.
E. coli	NC_013589.1	Plasmid	Orlek_et_al.
E. coli	NC_013652.1	Plasmid	Orlek_et_al.
E. coli	NC_013655.1	Plasmid	Orlek_et_al.
E. coli	NC_013717.1	Plasmid	Orlek_et_al.
E. coli	NC_013718.1	Plasmid	Orlek_et_al.
E. coli	NC_013719.1	Plasmid	Orlek_et_al.
E. coli	NC_013727.1	Plasmid	Orlek_et_al.
E. coli	NC_013728.1	Plasmid	Orlek_et_al.
E. coli	NC_013782.1	Plasmid	Orlek_et_al.
E. coli	NC_013942.1	Plasmid	Orlek_et_al.
E. coli	NC_013950.1	Plasmid	Orlek_et_al.
E. coli	NC_013951.1	Plasmid	Orlek_et_al.
E. coli	NC_013954.1	Plasmid	Orlek_et_al.
E. coli	NC_013957.1	Plasmid	Orlek_et_al.
E. coli	NC_013962.1	Plasmid	Orlek_et_al.
E. coli	NC_013972.1	Plasmid	Orlek_et_al.
E. coli	NC_013973.1	Plasmid	Orlek_et_al.
E. coli	NC_014003.1	Plasmid	Orlek_et_al.
E. coli	NC_014016.1	Plasmid	Orlek_et_al.
E. coli	NC_014017.1	Plasmid	Orlek_et_al.
E. coli	NC_014022.1	Plasmid	Orlek_et_al.
E. coli	NC_014027.1	Plasmid	Orlek_et_al.

E. coli	NC_014107.1	Plasmid	Orlek_et_al.
E. coli	NC_014108.1	Plasmid	Orlek_et_al.
E. coli	NC_014170.1	Plasmid	Orlek_et_al.
E. coli	NC_014208.1	Plasmid	Orlek_et_al.
E. coli	NC_014227.1	Plasmid	Orlek_et_al.
E. coli	NC_014231.1	Plasmid	Orlek_et_al.
E. coli	NC_014232.1	Plasmid	Orlek_et_al.
E. coli	NC_014233.1	Plasmid	Orlek_et_al.
E. coli	NC_014234.1	Plasmid	Orlek_et_al.
E. coli	NC_014235.1	Plasmid	Orlek_et_al.
E. coli	NC_014258.1	Plasmid	Orlek_et_al.
E. coli	NC_014304.1	Plasmid	Orlek_et_al.
E. coli	NC_014305.1	Plasmid	Orlek_et_al.
E. coli	NC_014312.1	Plasmid	Orlek_et_al.
E. coli	NC_014354.1	Plasmid	Orlek_et_al.
E. coli	NC_014356.1	Plasmid	Orlek_et_al.
E. coli	NC_014368.1	Plasmid	Orlek_et_al.
E. coli	NC_014382.1	Plasmid	Orlek_et_al.
E. coli	NC_014383.1	Plasmid	Orlek_et_al.
E. coli	NC_014384.1	Plasmid	Orlek_et_al.
E. coli	NC_014385.1	Plasmid	Orlek_et_al.
E. coli	NC_014476.2	Plasmid	Orlek_et_al.
E. coli	NC_014477.1	Plasmid	Orlek_et_al.
E. coli	NC_014478.1	Plasmid	Orlek_et_al.
E. coli	NC_014543.1	Plasmid	Orlek_et_al.
E. coli	NC_014561.1	Plasmid	Orlek_et_al.
E. coli	NC_014563.1	Plasmid	Orlek_et_al.
E. coli	NC_014615.1	Plasmid	Orlek_et_al.
E. coli	NC_014725.1	Plasmid	Orlek_et_al.
E. coli	NC_014838.1	Plasmid	Orlek_et_al.
E. coli	NC_014839.1	Plasmid	Orlek_et_al.
E. coli	NC_014840.1	Plasmid	Orlek_et_al.
E. coli	NC_014841.1	Plasmid	Orlek_et_al.
E. coli	NC_014842.1	Plasmid	Orlek_et_al.
E. coli	NC_014843.1	Plasmid	Orlek_et_al.
E. coli	NC_015054.1	Plasmid	Orlek_et_al.
E. coli	NC_015055.1	Plasmid	Orlek_et_al.
E. coli	NC_015056.1	Plasmid	Orlek_et_al.
E. coli	NC_015062.1	Plasmid	Orlek_et_al.
E. coli	NC_015063.1	Plasmid	Orlek_et_al.
E. coli	NC_015068.1	Plasmid	Orlek_et_al.
E. coli	NC_015154.1	Plasmid	Orlek_et_al.
E. coli	NC_015175.1	Plasmid	Orlek_et_al.
E. coli	NC_015392.1	Plasmid	Orlek_et_al.
E. coli	NC_015472.1	Plasmid	Orlek_et_al.
E. coli	NC_015475.1	Plasmid	Orlek_et_al.
E. coli	NC_015515.1	Plasmid	Orlek_et_al.
E. coli	NC_015570.1	Plasmid	Orlek_et_al.
E. coli	NC_015575.1	Plasmid	Orlek_et_al.
E. coli	NC_015599.1	Plasmid	Orlek_et_al.
E. coli	NC_015739.1	Plasmid	Orlek_et_al.
E. coli	NC_015872.1	Plasmid	Orlek_et_al.
E. coli	NC_015963.1	Plasmid	Orlek_et_al.
E. coli	NC_015965.1	Plasmid	Orlek_et_al.
E. coli	NC_015969.1	Plasmid	Orlek_et_al.
E. coli	NC_015972.1	Plasmid	Orlek_et_al.
E. coli	NC_016036.1	Plasmid	Orlek_et_al.
E. coli	NC_016039.1	Plasmid	Orlek_et_al.
E. coli	NC_016151.1	Plasmid	Orlek_et_al.
E. coli	NC_016515.1	Plasmid	Orlek_et_al.
E. coli	NC_016817.1	Plasmid	Orlek_et_al.
E. coli	NC_016819.1	Plasmid	Orlek_et_al.
E. coli	NC_016823.1	Plasmid	Orlek_et_al.
E. coli	NC_016824.1	Plasmid	Orlek_et_al.
E. coli	NC_016825.1	Plasmid	Orlek_et_al.
E. coli	NC_016833.1	Plasmid	Orlek_et_al.
E. coli	NC_016834.1	Plasmid	Orlek_et_al.
E. coli	NC_016835.1	Plasmid	Orlek_et_al.
E. coli	NC_016838.1	Plasmid	Orlek_et_al.
E. coli	NC_016839.1	Plasmid	Orlek_et_al.
E. coli	NC_016840.1	Plasmid	Orlek_et_al.
E. coli	NC_016841.1	Plasmid	Orlek_et_al.

E. coli	NC_016846.1	Plasmid	Orlek_et_al.
E. coli	NC_016847.1	Plasmid	Orlek_et_al.
E. coli	NC_016855.1	Plasmid	Orlek_et_al.
E. coli	NC_016861.1	Plasmid	Orlek_et_al.
E. coli	NC_016862.1	Plasmid	Orlek_et_al.
E. coli	NC_016864.1	Plasmid	Orlek_et_al.
E. coli	NC_016966.1	Plasmid	Orlek_et_al.
E. coli	NC_016974.1	Plasmid	Orlek_et_al.
E. coli	NC_016976.1	Plasmid	Orlek_et_al.
E. coli	NC_016979.1	Plasmid	Orlek_et_al.
E. coli	NC_016980.1	Plasmid	Orlek_et_al.
E. coli	NC_017054.1	Plasmid	Orlek_et_al.
E. coli	NC_017060.1	Plasmid	Orlek_et_al.
E. coli	NC_017153.1	Plasmid	Orlek_et_al.
E. coli	NC_017155.1	Plasmid	Orlek_et_al.
E. coli	NC_017156.1	Plasmid	Orlek_et_al.
E. coli	NC_017157.1	Plasmid	Orlek_et_al.
E. coli	NC_017158.1	Plasmid	Orlek_et_al.
E. coli	NC_017159.1	Plasmid	Orlek_et_al.
E. coli	NC_017169.1	Plasmid	Orlek_et_al.
E. coli	NC_017170.1	Plasmid	Orlek_et_al.
E. coli	NC_017257.1	Plasmid	Orlek_et_al.
E. coli	NC_017258.1	Plasmid	Orlek_et_al.
E. coli	NC_017260.1	Plasmid	Orlek_et_al.
E. coli	NC_017261.1	Plasmid	Orlek_et_al.
E. coli	NC_017263.1	Plasmid	Orlek_et_al.
E. coli	NC_017264.1	Plasmid	Orlek_et_al.
E. coli	NC_017266.1	Plasmid	Orlek_et_al.
E. coli	NC_017318.1	Plasmid	Orlek_et_al.
E. coli	NC_017319.1	Plasmid	Orlek_et_al.
E. coli	NC_017320.1	Plasmid	Orlek_et_al.
E. coli	NC_017321.1	Plasmid	Orlek_et_al.
E. coli	NC_017329.1	Plasmid	Orlek_et_al.
E. coli	NC_017330.1	Plasmid	Orlek_et_al.
E. coli	NC_017388.1	Plasmid	Orlek_et_al.
E. coli	NC_017389.1	Plasmid	Orlek_et_al.
E. coli	NC_017391.1	Plasmid	Orlek_et_al.
E. coli	NC_017392.1	Plasmid	Orlek_et_al.
E. coli	NC_017442.1	Plasmid	Orlek_et_al.
E. coli	NC_017444.1	Plasmid	Orlek_et_al.
E. coli	NC_017446.1	Plasmid	Orlek_et_al.
E. coli	NC_017533.1	Plasmid	Orlek_et_al.
E. coli	NC_017541.1	Plasmid	Orlek_et_al.
E. coli	NC_017553.1	Plasmid	Orlek_et_al.
E. coli	NC_017565.1	Plasmid	Orlek_et_al.
E. coli	NC_017624.1	Plasmid	Orlek_et_al.
E. coli	NC_017627.1	Plasmid	Orlek_et_al.
E. coli	NC_017629.1	Plasmid	Orlek_et_al.
E. coli	NC_017630.1	Plasmid	Orlek_et_al.
E. coli	NC_017637.1	Plasmid	Orlek_et_al.
E. coli	NC_017639.1	Plasmid	Orlek_et_al.
E. coli	NC_017640.1	Plasmid	Orlek_et_al.
E. coli	NC_017642.1	Plasmid	Orlek_et_al.
E. coli	NC_017643.1	Plasmid	Orlek_et_al.
E. coli	NC_017645.1	Plasmid	Orlek_et_al.
E. coli	NC_017647.1	Plasmid	Orlek_et_al.
E. coli	NC_017648.1	Plasmid	Orlek_et_al.
E. coli	NC_017649.1	Plasmid	Orlek_et_al.
E. coli	NC_017650.1	Plasmid	Orlek_et_al.
E. coli	NC_017653.1	Plasmid	Orlek_et_al.
E. coli	NC_017654.1	Plasmid	Orlek_et_al.
E. coli	NC_017655.1	Plasmid	Orlek_et_al.
E. coli	NC_017657.1	Plasmid	Orlek_et_al.
E. coli	NC_017658.1	Plasmid	Orlek_et_al.
E. coli	NC_017659.1	Plasmid	Orlek_et_al.
E. coli	NC_017661.1	Plasmid	Orlek_et_al.
E. coli	NC_017662.1	Plasmid	Orlek_et_al.
E. coli	NC_017665.1	Plasmid	Orlek_et_al.
E. coli	NC_017718.1	Plasmid	Orlek_et_al.
E. coli	NC_017719.1	Plasmid	Orlek_et_al.
E. coli	NC_017720.1	Plasmid	Orlek_et_al.
E. coli	NC_017722.1	Plasmid	Orlek_et_al.

E. coli	NC_017724.1	Plasmid	Orlek_et_al.
E. coli	NC_017903.1	Plasmid	Orlek_et_al.
E. coli	NC_017907.1	Plasmid	Orlek_et_al.
E. coli	NC_018107.1	Plasmid	Orlek_et_al.
E. coli	NC_018953.1	Plasmid	Orlek_et_al.
E. coli	NC_018954.1	Plasmid	Orlek_et_al.
E. coli	NC_018966.1	Plasmid	Orlek_et_al.
E. coli	NC_018977.1	Plasmid	Orlek_et_al.
E. coli	NC_018978.1	Plasmid	Orlek_et_al.
E. coli	NC_018985.1	Plasmid	Orlek_et_al.
E. coli	NC_018994.1	Plasmid	Orlek_et_al.
E. coli	NC_018995.1	Plasmid	Orlek_et_al.
E. coli	NC_018996.1	Plasmid	Orlek_et_al.
E. coli	NC_018997.1	Plasmid	Orlek_et_al.
E. coli	NC_018998.1	Plasmid	Orlek_et_al.
E. coli	NC_018999.1	Plasmid	Orlek_et_al.
E. coli	NC_019000.1	Plasmid	Orlek_et_al.
E. coli	NC_019001.1	Plasmid	Orlek_et_al.
E. coli	NC_019002.1	Plasmid	Orlek_et_al.
E. coli	NC_019013.1	Plasmid	Orlek_et_al.
E. coli	NC_019033.1	Plasmid	Orlek_et_al.
E. coli	NC_019037.1	Plasmid	Orlek_et_al.
E. coli	NC_019038.1	Plasmid	Orlek_et_al.
E. coli	NC_019039.1	Plasmid	Orlek_et_al.
E. coli	NC_019040.1	Plasmid	Orlek_et_al.
E. coli	NC_019043.1	Plasmid	Orlek_et_al.
E. coli	NC_019044.1	Plasmid	Orlek_et_al.
E. coli	NC_019045.2	Plasmid	Orlek_et_al.
E. coli	NC_019046.1	Plasmid	Orlek_et_al.
E. coli	NC_019047.1	Plasmid	Orlek_et_al.
E. coli	NC_019049.1	Plasmid	Orlek_et_al.
E. coli	NC_019050.1	Plasmid	Orlek_et_al.
E. coli	NC_019051.1	Plasmid	Orlek_et_al.
E. coli	NC_019052.1	Plasmid	Orlek_et_al.
E. coli	NC_019053.1	Plasmid	Orlek_et_al.
E. coli	NC_019054.1	Plasmid	Orlek_et_al.
E. coli	NC_019056.1	Plasmid	Orlek_et_al.
E. coli	NC_019057.1	Plasmid	Orlek_et_al.
E. coli	NC_019058.1	Plasmid	Orlek_et_al.
E. coli	NC_019059.1	Plasmid	Orlek_et_al.
E. coli	NC_019060.1	Plasmid	Orlek_et_al.
E. coli	NC_019061.1	Plasmid	Orlek_et_al.
E. coli	NC_019062.1	Plasmid	Orlek_et_al.
E. coli	NC_019063.1	Plasmid	Orlek_et_al.
E. coli	NC_019065.1	Plasmid	Orlek_et_al.
E. coli	NC_019066.1	Plasmid	Orlek_et_al.
E. coli	NC_019067.1	Plasmid	Orlek_et_al.
E. coli	NC_019068.1	Plasmid	Orlek_et_al.
E. coli	NC_019069.1	Plasmid	Orlek_et_al.
E. coli	NC_019070.1	Plasmid	Orlek_et_al.
E. coli	NC_019071.1	Plasmid	Orlek_et_al.
E. coli	NC_019072.1	Plasmid	Orlek_et_al.
E. coli	NC_019073.1	Plasmid	Orlek_et_al.
E. coli	NC_019074.1	Plasmid	Orlek_et_al.
E. coli	NC_019075.1	Plasmid	Orlek_et_al.
E. coli	NC_019076.1	Plasmid	Orlek_et_al.
E. coli	NC_019077.1	Plasmid	Orlek_et_al.
E. coli	NC_019078.1	Plasmid	Orlek_et_al.
E. coli	NC_019079.1	Plasmid	Orlek_et_al.
E. coli	NC_019080.1	Plasmid	Orlek_et_al.
E. coli	NC_019081.1	Plasmid	Orlek_et_al.
E. coli	NC_019082.1	Plasmid	Orlek_et_al.
E. coli	NC_019083.1	Plasmid	Orlek_et_al.
E. coli	NC_019084.1	Plasmid	Orlek_et_al.
E. coli	NC_019085.1	Plasmid	Orlek_et_al.
E. coli	NC_019086.1	Plasmid	Orlek_et_al.
E. coli	NC_019087.1	Plasmid	Orlek_et_al.
E. coli	NC_019088.1	Plasmid	Orlek_et_al.
E. coli	NC_019089.1	Plasmid	Orlek_et_al.
E. coli	NC_019090.1	Plasmid	Orlek_et_al.
E. coli	NC_019091.1	Plasmid	Orlek_et_al.
E. coli	NC_019092.1	Plasmid	Orlek_et_al.

E. coli	NC_019093.1	Plasmid	Orlek_et_al.
E. coli	NC_019094.1	Plasmid	Orlek_et_al.
E. coli	NC_019095.1	Plasmid	Orlek_et_al.
E. coli	NC_019096.1	Plasmid	Orlek_et_al.
E. coli	NC_019097.1	Plasmid	Orlek_et_al.
E. coli	NC_019098.1	Plasmid	Orlek_et_al.
E. coli	NC_019099.1	Plasmid	Orlek_et_al.
E. coli	NC_019101.1	Plasmid	Orlek_et_al.
E. coli	NC_019102.1	Plasmid	Orlek_et_al.
E. coli	NC_019103.1	Plasmid	Orlek_et_al.
E. coli	NC_019104.1	Plasmid	Orlek_et_al.
E. coli	NC_019105.1	Plasmid	Orlek_et_al.
E. coli	NC_019106.1	Plasmid	Orlek_et_al.
E. coli	NC_019107.1	Plasmid	Orlek_et_al.
E. coli	NC_019108.1	Plasmid	Orlek_et_al.
E. coli	NC_019109.1	Plasmid	Orlek_et_al.
E. coli	NC_019110.1	Plasmid	Orlek_et_al.
E. coli	NC_019111.1	Plasmid	Orlek_et_al.
E. coli	NC_019112.1	Plasmid	Orlek_et_al.
E. coli	NC_019113.1	Plasmid	Orlek_et_al.
E. coli	NC_019114.1	Plasmid	Orlek_et_al.
E. coli	NC_019115.1	Plasmid	Orlek_et_al.
E. coli	NC_019116.1	Plasmid	Orlek_et_al.
E. coli	NC_019117.1	Plasmid	Orlek_et_al.
E. coli	NC_019118.1	Plasmid	Orlek_et_al.
E. coli	NC_019120.1	Plasmid	Orlek_et_al.
E. coli	NC_019121.1	Plasmid	Orlek_et_al.
E. coli	NC_019122.1	Plasmid	Orlek_et_al.
E. coli	NC_019123.1	Plasmid	Orlek_et_al.
E. coli	NC_019124.1	Plasmid	Orlek_et_al.
E. coli	NC_019125.1	Plasmid	Orlek_et_al.
E. coli	NC_019126.1	Plasmid	Orlek_et_al.
E. coli	NC_019127.1	Plasmid	Orlek_et_al.
E. coli	NC_019128.1	Plasmid	Orlek_et_al.
E. coli	NC_019129.1	Plasmid	Orlek_et_al.
E. coli	NC_019130.1	Plasmid	Orlek_et_al.
E. coli	NC_019131.1	Plasmid	Orlek_et_al.
E. coli	NC_019132.1	Plasmid	Orlek_et_al.
E. coli	NC_019133.1	Plasmid	Orlek_et_al.
E. coli	NC_019134.1	Plasmid	Orlek_et_al.
E. coli	NC_019135.1	Plasmid	Orlek_et_al.
E. coli	NC_019136.1	Plasmid	Orlek_et_al.
E. coli	NC_019137.1	Plasmid	Orlek_et_al.
E. coli	NC_019151.1	Plasmid	Orlek_et_al.
E. coli	NC_019152.1	Plasmid	Orlek_et_al.
E. coli	NC_019153.1	Plasmid	Orlek_et_al.
E. coli	NC_019154.1	Plasmid	Orlek_et_al.
E. coli	NC_019155.1	Plasmid	Orlek_et_al.
E. coli	NC_019156.1	Plasmid	Orlek_et_al.
E. coli	NC_019157.1	Plasmid	Orlek_et_al.
E. coli	NC_019158.1	Plasmid	Orlek_et_al.
E. coli	NC_019159.1	Plasmid	Orlek_et_al.
E. coli	NC_019160.1	Plasmid	Orlek_et_al.
E. coli	NC_019161.1	Plasmid	Orlek_et_al.
E. coli	NC_019162.1	Plasmid	Orlek_et_al.
E. coli	NC_019163.1	Plasmid	Orlek_et_al.
E. coli	NC_019165.1	Plasmid	Orlek_et_al.
E. coli	NC_019166.1	Plasmid	Orlek_et_al.
E. coli	NC_019187.1	Plasmid	Orlek_et_al.
E. coli	NC_019188.1	Plasmid	Orlek_et_al.
E. coli	NC_019197.1	Plasmid	Orlek_et_al.
E. coli	NC_019203.1	Plasmid	Orlek_et_al.
E. coli	NC_019204.1	Plasmid	Orlek_et_al.
E. coli	NC_019221.1	Plasmid	Orlek_et_al.
E. coli	NC_019222.1	Plasmid	Orlek_et_al.
E. coli	NC_019223.1	Plasmid	Orlek_et_al.
E. coli	NC_019224.1	Plasmid	Orlek_et_al.
E. coli	NC_019234.1	Plasmid	Orlek_et_al.
E. coli	NC_019242.1	Plasmid	Orlek_et_al.
E. coli	NC_019249.1	Plasmid	Orlek_et_al.
E. coli	NC_019250.1	Plasmid	Orlek_et_al.
E. coli	NC_019251.1	Plasmid	Orlek_et_al.

E. coli	NC_019254.1	Plasmid	Orlek_et_al.
E. coli	NC_019256.1	Plasmid	Orlek_et_al.
E. coli	NC_019267.1	Plasmid	Orlek_et_al.
E. coli	NC_019269.1	Plasmid	Orlek_et_al.
E. coli	NC_019279.1	Plasmid	Orlek_et_al.
E. coli	NC_019286.1	Plasmid	Orlek_et_al.
E. coli	NC_019295.1	Plasmid	Orlek_et_al.
E. coli	NC_019296.1	Plasmid	Orlek_et_al.
E. coli	NC_019297.1	Plasmid	Orlek_et_al.
E. coli	NC_019298.1	Plasmid	Orlek_et_al.
E. coli	NC_019310.1	Plasmid	Orlek_et_al.
E. coli	NC_019335.1	Plasmid	Orlek_et_al.
E. coli	NC_019336.1	Plasmid	Orlek_et_al.
E. coli	NC_019342.1	Plasmid	Orlek_et_al.
E. coli	NC_019343.1	Plasmid	Orlek_et_al.
E. coli	NC_019344.1	Plasmid	Orlek_et_al.
E. coli	NC_019346.1	Plasmid	Orlek_et_al.
E. coli	NC_019357.1	Plasmid	Orlek_et_al.
E. coli	NC_019360.1	Plasmid	Orlek_et_al.
E. coli	NC_019368.1	Plasmid	Orlek_et_al.
E. coli	NC_019373.1	Plasmid	Orlek_et_al.
E. coli	NC_019374.1	Plasmid	Orlek_et_al.
E. coli	NC_019375.1	Plasmid	Orlek_et_al.
E. coli	NC_019384.1	Plasmid	Orlek_et_al.
E. coli	NC_019389.1	Plasmid	Orlek_et_al.
E. coli	NC_019390.1	Plasmid	Orlek_et_al.
E. coli	NC_019424.1	Plasmid	Orlek_et_al.
E. coli	NC_019888.1	Plasmid	Orlek_et_al.
E. coli	NC_019889.1	Plasmid	Orlek_et_al.
E. coli	NC_019899.1	Plasmid	Orlek_et_al.
E. coli	NC_019982.1	Plasmid	Orlek_et_al.
E. coli	NC_019983.1	Plasmid	Orlek_et_al.
E. coli	NC_019984.1	Plasmid	Orlek_et_al.
E. coli	NC_019986.1	Plasmid	Orlek_et_al.
E. coli	NC_019987.1	Plasmid	Orlek_et_al.
E. coli	NC_019988.1	Plasmid	Orlek_et_al.
E. coli	NC_019989.1	Plasmid	Orlek_et_al.
E. coli	NC_019990.1	Plasmid	Orlek_et_al.
E. coli	NC_019991.1	Plasmid	Orlek_et_al.
E. coli	NC_020086.1	Plasmid	Orlek_et_al.
E. coli	NC_020087.1	Plasmid	Orlek_et_al.
E. coli	NC_020088.1	Plasmid	Orlek_et_al.
E. coli	NC_020122.1	Plasmid	Orlek_et_al.
E. coli	NC_020123.1	Plasmid	Orlek_et_al.
E. coli	NC_020131.1	Plasmid	Orlek_et_al.
E. coli	NC_020132.1	Plasmid	Orlek_et_al.
E. coli	NC_020180.1	Plasmid	Orlek_et_al.
E. coli	NC_020212.1	Plasmid	Orlek_et_al.
E. coli	NC_020251.1	Plasmid	Orlek_et_al.
E. coli	NC_020261.1	Plasmid	Orlek_et_al.
E. coli	NC_020262.1	Plasmid	Orlek_et_al.
E. coli	NC_020263.1	Plasmid	Orlek_et_al.
E. coli	NC_020270.1	Plasmid	Orlek_et_al.
E. coli	NC_020271.1	Plasmid	Orlek_et_al.
E. coli	NC_020278.2	Plasmid	Orlek_et_al.
E. coli	NC_020279.1	Plasmid	Orlek_et_al.
E. coli	NC_020280.1	Plasmid	Orlek_et_al.
E. coli	NC_020281.1	Plasmid	Orlek_et_al.
E. coli	NC_020282.1	Plasmid	Orlek_et_al.
E. coli	NC_020295.1	Plasmid	Orlek_et_al.
E. coli	NC_020306.1	Plasmid	Orlek_et_al.
E. coli	NC_020308.1	Plasmid	Orlek_et_al.
E. coli	NC_020412.1	Plasmid	Orlek_et_al.
E. coli	NC_020413.1	Plasmid	Orlek_et_al.
E. coli	NC_020552.1	Plasmid	Orlek_et_al.
E. coli	NC_020811.1	Plasmid	Orlek_et_al.
E. coli	NC_020893.1	Plasmid	Orlek_et_al.
E. coli	NC_020915.1	Plasmid	Orlek_et_al.
E. coli	NC_020916.1	Plasmid	Orlek_et_al.
E. coli	NC_020918.1	Plasmid	Orlek_et_al.
E. coli	NC_020919.1	Plasmid	Orlek_et_al.
E. coli	NC_020920.1	Plasmid	Orlek_et_al.

E. coli	NC_020921.1	Plasmid	Orlek_et_al.
E. coli	NC_020991.1	Plasmid	Orlek_et_al.
E. coli	NC_021078.1	Plasmid	Orlek_et_al.
E. coli	NC_021079.1	Plasmid	Orlek_et_al.
E. coli	NC_021087.1	Plasmid	Orlek_et_al.
E. coli	NC_021155.1	Plasmid	Orlek_et_al.
E. coli	NC_021156.1	Plasmid	Orlek_et_al.
E. coli	NC_021157.1	Plasmid	Orlek_et_al.
E. coli	NC_021180.1	Plasmid	Orlek_et_al.
E. coli	NC_021198.1	Plasmid	Orlek_et_al.
E. coli	NC_021199.1	Plasmid	Orlek_et_al.
E. coli	NC_021231.1	Plasmid	Orlek_et_al.
E. coli	NC_021238.1	Plasmid	Orlek_et_al.
E. coli	NC_021293.1	Plasmid	Orlek_et_al.
E. coli	NC_021488.1	Plasmid	Orlek_et_al.
E. coli	NC_021492.1	Plasmid	Orlek_et_al.
E. coli	NC_021501.1	Plasmid	Orlek_et_al.
E. coli	NC_021502.1	Plasmid	Orlek_et_al.
E. coli	NC_021511.1	Plasmid	Orlek_et_al.
E. coli	NC_021522.1	Plasmid	Orlek_et_al.
E. coli	NC_021523.1	Plasmid	Orlek_et_al.
E. coli	NC_021524.1	Plasmid	Orlek_et_al.
E. coli	NC_021570.1	Plasmid	Orlek_et_al.
E. coli	NC_021576.1	Plasmid	Orlek_et_al.
E. coli	NC_021594.1	Plasmid	Orlek_et_al.
E. coli	NC_021622.1	Plasmid	Orlek_et_al.
E. coli	NC_021654.1	Plasmid	Orlek_et_al.
E. coli	NC_021655.1	Plasmid	Orlek_et_al.
E. coli	NC_021656.1	Plasmid	Orlek_et_al.
E. coli	NC_021660.2	Plasmid	Orlek_et_al.
E. coli	NC_021664.2	Plasmid	Orlek_et_al.
E. coli	NC_021666.1	Plasmid	Orlek_et_al.
E. coli	NC_021667.1	Plasmid	Orlek_et_al.
E. coli	NC_021742.1	Plasmid	Orlek_et_al.
E. coli	NC_021811.1	Plasmid	Orlek_et_al.
E. coli	NC_021813.2	Plasmid	Orlek_et_al.
E. coli	NC_021815.1	Plasmid	Orlek_et_al.
E. coli	NC_021816.1	Plasmid	Orlek_et_al.
E. coli	NC_021817.1	Plasmid	Orlek_et_al.
E. coli	NC_021819.1	Plasmid	Orlek_et_al.
E. coli	NC_021835.1	Plasmid	Orlek_et_al.
E. coli	NC_021836.1	Plasmid	Orlek_et_al.
E. coli	NC_021841.1	Plasmid	Orlek_et_al.
E. coli	NC_021842.2	Plasmid	Orlek_et_al.
E. coli	NC_021843.1	Plasmid	Orlek_et_al.
E. coli	NC_021845.1	Plasmid	Orlek_et_al.
E. coli	NC_021869.1	Plasmid	Orlek_et_al.
E. coli	NC_021871.1	Plasmid	Orlek_et_al.
E. coli	NC_021922.1	Plasmid	Orlek_et_al.
E. coli	NC_021927.1	Plasmid	Orlek_et_al.
E. coli	NC_022078.1	Plasmid	Orlek_et_al.
E. coli	NC_022083.1	Plasmid	Orlek_et_al.
E. coli	NC_022105.1	Plasmid	Orlek_et_al.
E. coli	NC_022197.1	Plasmid	Orlek_et_al.
E. coli	NC_022267.1	Plasmid	Orlek_et_al.
E. coli	NC_022333.1	Plasmid	Orlek_et_al.
E. coli	NC_022371.1	Plasmid	Orlek_et_al.
E. coli	NC_022372.1	Plasmid	Orlek_et_al.
E. coli	NC_022374.1	Plasmid	Orlek_et_al.
E. coli	NC_022375.1	Plasmid	Orlek_et_al.
E. coli	NC_022376.1	Plasmid	Orlek_et_al.
E. coli	NC_022377.1	Plasmid	Orlek_et_al.
E. coli	NC_022520.1	Plasmid	Orlek_et_al.
E. coli	NC_022522.2	Plasmid	Orlek_et_al.
E. coli	NC_022533.1	Plasmid	Orlek_et_al.
E. coli	NC_022534.1	Plasmid	Orlek_et_al.
E. coli	NC_022570.1	Plasmid	Orlek_et_al.
E. coli	NC_022585.1	Plasmid	Orlek_et_al.
E. coli	NC_022589.1	Plasmid	Orlek_et_al.
E. coli	NC_022609.1	Plasmid	Orlek_et_al.
E. coli	NC_022652.1	Plasmid	Orlek_et_al.
E. coli	NC_022740.1	Plasmid	Orlek_et_al.

E. coli	NC_022741.1	Plasmid	Orlek_et_al.
E. coli	NC_022742.1	Plasmid	Orlek_et_al.
E. coli	NC_022743.1	Plasmid	Orlek_et_al.
E. coli	NC_022885.1	Plasmid	Orlek_et_al.
E. coli	NC_022963.1	Plasmid	Orlek_et_al.
E. coli	NC_022992.1	Plasmid	Orlek_et_al.
E. coli	NC_022996.1	Plasmid	Orlek_et_al.
E. coli	NC_023024.1	Plasmid	Orlek_et_al.
E. coli	NC_023025.1	Plasmid	Orlek_et_al.
E. coli	NC_023027.1	Plasmid	Orlek_et_al.
E. coli	NC_023056.1	Plasmid	Orlek_et_al.
E. coli	NC_023071.1	Plasmid	Orlek_et_al.
E. coli	NC_023072.1	Plasmid	Orlek_et_al.
E. coli	NC_023275.1	Plasmid	Orlek_et_al.
E. coli	NC_023276.1	Plasmid	Orlek_et_al.
E. coli	NC_023277.2	Plasmid	Orlek_et_al.
E. coli	NC_023289.2	Plasmid	Orlek_et_al.
E. coli	NC_023290.1	Plasmid	Orlek_et_al.
E. coli	NC_023314.1	Plasmid	Orlek_et_al.
E. coli	NC_023315.1	Plasmid	Orlek_et_al.
E. coli	NC_023317.1	Plasmid	Orlek_et_al.
E. coli	NC_023318.1	Plasmid	Orlek_et_al.
E. coli	NC_023319.1	Plasmid	Orlek_et_al.
E. coli	NC_023320.1	Plasmid	Orlek_et_al.
E. coli	NC_023321.1	Plasmid	Orlek_et_al.
E. coli	NC_023323.1	Plasmid	Orlek_et_al.
E. coli	NC_023324.1	Plasmid	Orlek_et_al.
E. coli	NC_023325.1	Plasmid	Orlek_et_al.
E. coli	NC_023326.1	Plasmid	Orlek_et_al.
E. coli	NC_023327.1	Plasmid	Orlek_et_al.
E. coli	NC_023328.1	Plasmid	Orlek_et_al.
E. coli	NC_023329.1	Plasmid	Orlek_et_al.
E. coli	NC_023330.1	Plasmid	Orlek_et_al.
E. coli	NC_023331.1	Plasmid	Orlek_et_al.
E. coli	NC_023332.1	Plasmid	Orlek_et_al.
E. coli	NC_023333.1	Plasmid	Orlek_et_al.
E. coli	NC_023334.1	Plasmid	Orlek_et_al.
E. coli	NC_023898.1	Plasmid	Orlek_et_al.
E. coli	NC_023899.1	Plasmid	Orlek_et_al.
E. coli	NC_023900.1	Plasmid	Orlek_et_al.
E. coli	NC_023901.1	Plasmid	Orlek_et_al.
E. coli	NC_023903.1	Plasmid	Orlek_et_al.
E. coli	NC_023904.1	Plasmid	Orlek_et_al.
E. coli	NC_023905.1	Plasmid	Orlek_et_al.
E. coli	NC_023906.1	Plasmid	Orlek_et_al.
E. coli	NC_023907.1	Plasmid	Orlek_et_al.
E. coli	NC_023908.1	Plasmid	Orlek_et_al.
E. coli	NC_023909.1	Plasmid	Orlek_et_al.
E. coli	NC_023910.1	Plasmid	Orlek_et_al.
E. coli	NC_023911.1	Plasmid	Orlek_et_al.
E. coli	NC_023912.1	Plasmid	Orlek_et_al.
E. coli	NC_023914.1	Plasmid	Orlek_et_al.
E. coli	NC_023915.1	Plasmid	Orlek_et_al.
E. coli	NC_023916.1	Plasmid	Orlek_et_al.
E. coli	NC_024954.1	Plasmid	Orlek_et_al.
E. coli	NC_024955.2	Plasmid	Orlek_et_al.
E. coli	NC_024956.1	Plasmid	Orlek_et_al.
E. coli	NC_024960.1	Plasmid	Orlek_et_al.
E. coli	NC_024961.1	Plasmid	Orlek_et_al.
E. coli	NC_024962.1	Plasmid	Orlek_et_al.
E. coli	NC_024967.1	Plasmid	Orlek_et_al.
E. coli	NC_024968.1	Plasmid	Orlek_et_al.
E. coli	NC_024969.1	Plasmid	Orlek_et_al.
E. coli	NC_024974.1	Plasmid	Orlek_et_al.
E. coli	NC_024975.1	Plasmid	Orlek_et_al.
E. coli	NC_024976.1	Plasmid	Orlek_et_al.
E. coli	NC_024977.1	Plasmid	Orlek_et_al.
E. coli	NC_024978.1	Plasmid	Orlek_et_al.
E. coli	NC_024979.1	Plasmid	Orlek_et_al.
E. coli	NC_024980.1	Plasmid	Orlek_et_al.
E. coli	NC_024981.1	Plasmid	Orlek_et_al.
E. coli	NC_024982.1	Plasmid	Orlek_et_al.

E. coli	NC_024983.1	Plasmid	Orlek_et_al.
E. coli	NC_024987.1	Plasmid	Orlek_et_al.
E. coli	NC_024992.1	Plasmid	Orlek_et_al.
E. coli	NC_024996.1	Plasmid	Orlek_et_al.
E. coli	NC_024997.1	Plasmid	Orlek_et_al.
E. coli	NC_025003.1	Plasmid	Orlek_et_al.
E. coli	NC_025013.1	Plasmid	Orlek_et_al.
E. coli	NC_025019.1	Plasmid	Orlek_et_al.
E. coli	NC_025021.1	Plasmid	Orlek_et_al.
E. coli	NC_025043.1	Plasmid	Orlek_et_al.
E. coli	NC_025044.1	Plasmid	Orlek_et_al.
E. coli	NC_025058.1	Plasmid	Orlek_et_al.
E. coli	NC_025105.1	Plasmid	Orlek_et_al.
E. coli	NC_025106.1	Plasmid	Orlek_et_al.
E. coli	NC_025130.1	Plasmid	Orlek_et_al.
E. coli	NC_025131.1	Plasmid	Orlek_et_al.
E. coli	NC_025134.1	Plasmid	Orlek_et_al.
E. coli	NC_025138.1	Plasmid	Orlek_et_al.
E. coli	NC_025139.1	Plasmid	Orlek_et_al.
E. coli	NC_025140.1	Plasmid	Orlek_et_al.
E. coli	NC_025141.1	Plasmid	Orlek_et_al.
E. coli	NC_025142.1	Plasmid	Orlek_et_al.
E. coli	NC_025143.1	Plasmid	Orlek_et_al.
E. coli	NC_025144.1	Plasmid	Orlek_et_al.
E. coli	NC_025145.1	Plasmid	Orlek_et_al.
E. coli	NC_025147.1	Plasmid	Orlek_et_al.
E. coli	NC_025148.1	Plasmid	Orlek_et_al.
E. coli	NC_025149.1	Plasmid	Orlek_et_al.
E. coli	NC_025150.1	Plasmid	Orlek_et_al.
E. coli	NC_025166.1	Plasmid	Orlek_et_al.
E. coli	NC_025167.1	Plasmid	Orlek_et_al.
E. coli	NC_025175.1	Plasmid	Orlek_et_al.
E. coli	NC_025176.1	Plasmid	Orlek_et_al.
E. coli	NC_025177.1	Plasmid	Orlek_et_al.
E. coli	NC_025178.1	Plasmid	Orlek_et_al.
E. coli	NC_025179.1	Plasmid	Orlek_et_al.
E. coli	NC_025180.1	Plasmid	Orlek_et_al.
E. coli	NC_025181.1	Plasmid	Orlek_et_al.
E. coli	NC_025182.1	Plasmid	Orlek_et_al.
E. coli	NC_025183.1	Plasmid	Orlek_et_al.
E. coli	NC_025184.1	Plasmid	Orlek_et_al.
E. coli	NC_025186.1	Plasmid	Orlek_et_al.
E. coli	NC_025187.1	Plasmid	Orlek_et_al.
E. coli	NC_025190.1	Plasmid	Orlek_et_al.
E. coli	NC_025198.1	Plasmid	Orlek_et_al.
E. coli	NC_028464.1	Plasmid	Orlek_et_al.
E. coli	NZ_AKKM01000049.1	Plasmid	Orlek_et_al.
E. coli	NZ_AP010873.1	Plasmid	Orlek_et_al.
E. coli	NZ_AP013064.1	Plasmid	Orlek_et_al.
E. coli	NZ_AP013065.1	Plasmid	Orlek_et_al.
E. coli	NZ_AP014566.1	Plasmid	Orlek_et_al.
E. coli	NZ_AP014952.1	Plasmid	Orlek_et_al.
E. coli	NZ_AP014953.1	Plasmid	Orlek_et_al.
E. coli	NZ_AP014954.1	Plasmid	Orlek_et_al.
E. coli	NZ_AP014955.1	Plasmid	Orlek_et_al.
E. coli	NZ_CM001146.1	Plasmid	Orlek_et_al.
E. coli	NZ_CM001850.1	Plasmid	Orlek_et_al.
E. coli	NZ_CM001851.1	Plasmid	Orlek_et_al.
E. coli	NZ_CM001853.1	Plasmid	Orlek_et_al.
E. coli	NZ_CM001854.1	Plasmid	Orlek_et_al.
E. coli	NZ_CM001855.1	Plasmid	Orlek_et_al.
E. coli	NZ_CM001856.1	Plasmid	Orlek_et_al.
E. coli	NZ_CM003247.1	Plasmid	Orlek_et_al.
E. coli	NZ_CM003276.1	Plasmid	Orlek_et_al.
E. coli	NZ_CM003365.1	Plasmid	Orlek_et_al.
E. coli	NZ_CM003366.1	Plasmid	Orlek_et_al.
E. coli	NZ_CM003367.1	Plasmid	Orlek_et_al.
E. coli	NZ_CM003368.1	Plasmid	Orlek_et_al.
E. coli	NZ_CM003470.1	Plasmid	Orlek_et_al.
E. coli	NZ_CM003471.1	Plasmid	Orlek_et_al.
E. coli	NZ_CM003649.1	Plasmid	Orlek_et_al.
E. coli	NZ_CP002698.1	Plasmid	Orlek_et_al.

E. coli	NZ_CP002700.1	Plasmid	Orlek_et_al.
E. coli	NZ_CP002702.1	Plasmid	Orlek_et_al.
E. coli	NZ_CP002704.1	Plasmid	Orlek_et_al.
E. coli	NZ_CP003425.1	Plasmid	Orlek_et_al.
E. coli	NZ_CP003994.1	Plasmid	Orlek_et_al.
E. coli	NZ_CP003995.1	Plasmid	Orlek_et_al.
E. coli	NZ_CP003996.1	Plasmid	Orlek_et_al.
E. coli	NZ_CP003997.1	Plasmid	Orlek_et_al.
E. coli	NZ_CP003998.1	Plasmid	Orlek_et_al.
E. coli	NZ_CP004000.1	Plasmid	Orlek_et_al.
E. coli	NZ_CP005931.1	Plasmid	Orlek_et_al.
E. coli	NZ_CP005932.1	Plasmid	Orlek_et_al.
E. coli	NZ_CP005994.1	Plasmid	Orlek_et_al.
E. coli	NZ_CP005999.1	Plasmid	Orlek_et_al.
E. coli	NZ_CP006000.1	Plasmid	Orlek_et_al.
E. coli	NZ_CP006001.1	Plasmid	Orlek_et_al.
E. coli	NZ_CP006002.1	Plasmid	Orlek_et_al.
E. coli	NZ_CP006028.1	Plasmid	Orlek_et_al.
E. coli	NZ_CP006029.1	Plasmid	Orlek_et_al.
E. coli	NZ_CP006263.1	Plasmid	Orlek_et_al.
E. coli	NZ_CP006264.1	Plasmid	Orlek_et_al.
E. coli	NZ_CP006570.1	Plasmid	Orlek_et_al.
E. coli	NZ_CP006633.1	Plasmid	Orlek_et_al.
E. coli	NZ_CP006634.1	Plasmid	Orlek_et_al.
E. coli	NZ_CP006635.1	Plasmid	Orlek_et_al.
E. coli	NZ_CP006637.1	Plasmid	Orlek_et_al.
E. coli	NZ_CP006638.1	Plasmid	Orlek_et_al.
E. coli	NZ_CP006639.1	Plasmid	Orlek_et_al.
E. coli	NZ_CP006640.1	Plasmid	Orlek_et_al.
E. coli	NZ_CP006641.1	Plasmid	Orlek_et_al.
E. coli	NZ_CP006642.1	Plasmid	Orlek_et_al.
E. coli	NZ_CP006660.1	Plasmid	Orlek_et_al.
E. coli	NZ_CP006661.1	Plasmid	Orlek_et_al.
E. coli	NZ_CP006662.2	Plasmid	Orlek_et_al.
E. coli	NZ_CP006663.1	Plasmid	Orlek_et_al.
E. coli	NZ_CP006665.1	Plasmid	Orlek_et_al.
E. coli	NZ_CP006666.1	Plasmid	Orlek_et_al.
E. coli	NZ_CP006746.1	Plasmid	Orlek_et_al.
E. coli	NZ_CP006747.1	Plasmid	Orlek_et_al.
E. coli	NZ_CP006749.1	Plasmid	Orlek_et_al.
E. coli	NZ_CP006750.1	Plasmid	Orlek_et_al.
E. coli	NZ_CP006752.1	Plasmid	Orlek_et_al.
E. coli	NZ_CP006753.1	Plasmid	Orlek_et_al.
E. coli	NZ_CP006756.1	Plasmid	Orlek_et_al.
E. coli	NZ_CP006757.1	Plasmid	Orlek_et_al.
E. coli	NZ_CP006760.1	Plasmid	Orlek_et_al.
E. coli	NZ_CP006761.1	Plasmid	Orlek_et_al.
E. coli	NZ_CP006779.1	Plasmid	Orlek_et_al.
E. coli	NZ_CP006780.1	Plasmid	Orlek_et_al.
E. coli	NZ_CP006790.1	Plasmid	Orlek_et_al.
E. coli	NZ_CP006791.1	Plasmid	Orlek_et_al.
E. coli	NZ_CP006793.1	Plasmid	Orlek_et_al.
E. coli	NZ_CP006795.1	Plasmid	Orlek_et_al.
E. coli	NZ_CP006796.1	Plasmid	Orlek_et_al.
E. coli	NZ_CP006797.1	Plasmid	Orlek_et_al.
E. coli	NZ_CP006799.1	Plasmid	Orlek_et_al.
E. coli	NZ_CP006800.1	Plasmid	Orlek_et_al.
E. coli	NZ_CP006801.1	Plasmid	Orlek_et_al.
E. coli	NZ_CP006807.1	Plasmid	Orlek_et_al.
E. coli	NZ_CP006808.1	Plasmid	Orlek_et_al.
E. coli	NZ_CP006919.1	Plasmid	Orlek_et_al.
E. coli	NZ_CP006921.1	Plasmid	Orlek_et_al.
E. coli	NZ_CP006922.1	Plasmid	Orlek_et_al.
E. coli	NZ_CP006924.1	Plasmid	Orlek_et_al.
E. coli	NZ_CP006925.1	Plasmid	Orlek_et_al.
E. coli	NZ_CP006926.1	Plasmid	Orlek_et_al.
E. coli	NZ_CP006927.1	Plasmid	Orlek_et_al.
E. coli	NZ_CP006928.1	Plasmid	Orlek_et_al.
E. coli	NZ_CP007038.1	Plasmid	Orlek_et_al.
E. coli	NZ_CP007134.1	Plasmid	Orlek_et_al.
E. coli	NZ_CP007135.1	Plasmid	Orlek_et_al.
E. coli	NZ_CP007137.1	Plasmid	Orlek_et_al.

E. coli	NZ_CP007150.1	Plasmid	Orlek_et_al.
E. coli	NZ_CP007231.1	Plasmid	Orlek_et_al.
E. coli	NZ_CP007488.1	Plasmid	Orlek_et_al.
E. coli	NZ_CP007489.1	Plasmid	Orlek_et_al.
E. coli	NZ_CP007508.1	Plasmid	Orlek_et_al.
E. coli	NZ_CP007529.1	Plasmid	Orlek_et_al.
E. coli	NZ_CP007558.1	Plasmid	Orlek_et_al.
E. coli	NZ_CP007582.1	Plasmid	Orlek_et_al.
E. coli	NZ_CP007599.1	Plasmid	Orlek_et_al.
E. coli	NZ_CP007732.1	Plasmid	Orlek_et_al.
E. coli	NZ_CP007733.1	Plasmid	Orlek_et_al.
E. coli	NZ_CP007734.1	Plasmid	Orlek_et_al.
E. coli	NZ_CP007735.1	Plasmid	Orlek_et_al.
E. coli	NZ_CP007736.1	Plasmid	Orlek_et_al.
E. coli	NZ_CP008715.1	Plasmid	Orlek_et_al.
E. coli	NZ_CP008716.1	Plasmid	Orlek_et_al.
E. coli	NZ_CP008717.1	Plasmid	Orlek_et_al.
E. coli	NZ_CP008718.1	Plasmid	Orlek_et_al.
E. coli	NZ_CP008719.1	Plasmid	Orlek_et_al.
E. coli	NZ_CP008720.1	Plasmid	Orlek_et_al.
E. coli	NZ_CP008721.1	Plasmid	Orlek_et_al.
E. coli	NZ_CP008745.1	Plasmid	Orlek_et_al.
E. coli	NZ_CP008789.1	Plasmid	Orlek_et_al.
E. coli	NZ_CP008790.1	Plasmid	Orlek_et_al.
E. coli	NZ_CP008791.1	Plasmid	Orlek_et_al.
E. coli	NZ_CP008798.1	Plasmid	Orlek_et_al.
E. coli	NZ_CP008799.1	Plasmid	Orlek_et_al.
E. coli	NZ_CP008800.1	Plasmid	Orlek_et_al.
E. coli	NZ_CP008806.1	Plasmid	Orlek_et_al.
E. coli	NZ_CP008807.1	Plasmid	Orlek_et_al.
E. coli	NZ_CP008824.1	Plasmid	Orlek_et_al.
E. coli	NZ_CP008825.1	Plasmid	Orlek_et_al.
E. coli	NZ_CP008826.1	Plasmid	Orlek_et_al.
E. coli	NZ_CP008828.1	Plasmid	Orlek_et_al.
E. coli	NZ_CP008829.1	Plasmid	Orlek_et_al.
E. coli	NZ_CP008842.1	Plasmid	Orlek_et_al.
E. coli	NZ_CP008843.1	Plasmid	Orlek_et_al.
E. coli	NZ_CP008844.1	Plasmid	Orlek_et_al.
E. coli	NZ_CP008845.1	Plasmid	Orlek_et_al.
E. coli	NZ_CP008898.1	Plasmid	Orlek_et_al.
E. coli	NZ_CP008899.1	Plasmid	Orlek_et_al.
E. coli	NZ_CP008900.1	Plasmid	Orlek_et_al.
E. coli	NZ_CP008906.1	Plasmid	Orlek_et_al.
E. coli	NZ_CP008907.1	Plasmid	Orlek_et_al.
E. coli	NZ_CP008919.1	Plasmid	Orlek_et_al.
E. coli	NZ_CP008927.1	Plasmid	Orlek_et_al.
E. coli	NZ_CP008930.1	Plasmid	Orlek_et_al.
E. coli	NZ_CP008931.1	Plasmid	Orlek_et_al.
E. coli	NZ_CP008932.1	Plasmid	Orlek_et_al.
E. coli	NZ_CP008933.1	Plasmid	Orlek_et_al.
E. coli	NZ_CP009103.1	Plasmid	Orlek_et_al.
E. coli	NZ_CP009105.1	Plasmid	Orlek_et_al.
E. coli	NZ_CP009107.1	Plasmid	Orlek_et_al.
E. coli	NZ_CP009115.1	Plasmid	Orlek_et_al.
E. coli	NZ_CP009116.1	Plasmid	Orlek_et_al.
E. coli	NZ_CP009126.1	Plasmid	Orlek_et_al.
E. coli	NZ_CP009167.1	Plasmid	Orlek_et_al.
E. coli	NZ_CP009168.1	Plasmid	Orlek_et_al.
E. coli	NZ_CP009169.1	Plasmid	Orlek_et_al.
E. coli	NZ_CP009254.1	Plasmid	Orlek_et_al.
E. coli	NZ_CP009255.1	Plasmid	Orlek_et_al.
E. coli	NZ_CP009275.1	Plasmid	Orlek_et_al.
E. coli	NZ_CP009276.1	Plasmid	Orlek_et_al.
E. coli	NZ_CP009363.1	Plasmid	Orlek_et_al.
E. coli	NZ_CP009366.1	Plasmid	Orlek_et_al.
E. coli	NZ_CP009409.2	Plasmid	Orlek_et_al.
E. coli	NZ_CP009457.1	Plasmid	Orlek_et_al.
E. coli	NZ_CP009489.1	Plasmid	Orlek_et_al.
E. coli	NZ_CP009490.1	Plasmid	Orlek_et_al.
E. coli	NZ_CP009491.1	Plasmid	Orlek_et_al.
E. coli	NZ_CP009560.1	Plasmid	Orlek_et_al.
E. coli	NZ_CP009562.1	Plasmid	Orlek_et_al.

E. coli	NZ_CP009563.1	Plasmid	Orlek_et_al.
E. coli	NZ_CP009564.1	Plasmid	Orlek_et_al.
E. coli	NZ_CP009566.1	Plasmid	Orlek_et_al.
E. coli	NZ_CP009567.1	Plasmid	Orlek_et_al.
E. coli	NZ_CP009568.1	Plasmid	Orlek_et_al.
E. coli	NZ_CP009570.1	Plasmid	Orlek_et_al.
E. coli	NZ_CP009579.1	Plasmid	Orlek_et_al.
E. coli	NZ_CP009581.1	Plasmid	Orlek_et_al.
E. coli	NZ_CP009582.1	Plasmid	Orlek_et_al.
E. coli	NZ_CP009701.1	Plasmid	Orlek_et_al.
E. coli	NZ_CP009702.1	Plasmid	Orlek_et_al.
E. coli	NZ_CP009703.1	Plasmid	Orlek_et_al.
E. coli	NZ_CP009710.1	Plasmid	Orlek_et_al.
E. coli	NZ_CP009711.1	Plasmid	Orlek_et_al.
E. coli	NZ_CP009713.1	Plasmid	Orlek_et_al.
E. coli	NZ_CP009714.1	Plasmid	Orlek_et_al.
E. coli	NZ_CP009721.1	Plasmid	Orlek_et_al.
E. coli	NZ_CP009722.1	Plasmid	Orlek_et_al.
E. coli	NZ_CP009724.1	Plasmid	Orlek_et_al.
E. coli	NZ_CP009758.1	Plasmid	Orlek_et_al.
E. coli	NZ_CP009767.1	Plasmid	Orlek_et_al.
E. coli	NZ_CP009772.1	Plasmid	Orlek_et_al.
E. coli	NZ_CP009773.1	Plasmid	Orlek_et_al.
E. coli	NZ_CP009774.1	Plasmid	Orlek_et_al.
E. coli	NZ_CP009776.1	Plasmid	Orlek_et_al.
E. coli	NZ_CP009777.1	Plasmid	Orlek_et_al.
E. coli	NZ_CP009778.1	Plasmid	Orlek_et_al.
E. coli	NZ_CP009779.1	Plasmid	Orlek_et_al.
E. coli	NZ_CP009783.1	Plasmid	Orlek_et_al.
E. coli	NZ_CP009784.1	Plasmid	Orlek_et_al.
E. coli	NZ_CP009834.1	Plasmid	Orlek_et_al.
E. coli	NZ_CP009835.1	Plasmid	Orlek_et_al.
E. coli	NZ_CP009837.1	Plasmid	Orlek_et_al.
E. coli	NZ_CP009839.1	Plasmid	Orlek_et_al.
E. coli	NZ_CP009841.1	Plasmid	Orlek_et_al.
E. coli	NZ_CP009842.1	Plasmid	Orlek_et_al.
E. coli	NZ_CP009843.1	Plasmid	Orlek_et_al.
E. coli	NZ_CP009851.1	Plasmid	Orlek_et_al.
E. coli	NZ_CP009852.1	Plasmid	Orlek_et_al.
E. coli	NZ_CP009853.1	Plasmid	Orlek_et_al.
E. coli	NZ_CP009855.1	Plasmid	Orlek_et_al.
E. coli	NZ_CP009856.1	Plasmid	Orlek_et_al.
E. coli	NZ_CP009857.1	Plasmid	Orlek_et_al.
E. coli	NZ_CP009858.1	Plasmid	Orlek_et_al.
E. coli	NZ_CP009860.1	Plasmid	Orlek_et_al.
E. coli	NZ_CP009861.1	Plasmid	Orlek_et_al.
E. coli	NZ_CP009862.1	Plasmid	Orlek_et_al.
E. coli	NZ_CP009864.1	Plasmid	Orlek_et_al.
E. coli	NZ_CP009865.1	Plasmid	Orlek_et_al.
E. coli	NZ_CP009867.1	Plasmid	Orlek_et_al.
E. coli	NZ_CP009868.1	Plasmid	Orlek_et_al.
E. coli	NZ_CP009869.1	Plasmid	Orlek_et_al.
E. coli	NZ_CP009870.1	Plasmid	Orlek_et_al.
E. coli	NZ_CP009871.1	Plasmid	Orlek_et_al.
E. coli	NZ_CP009873.1	Plasmid	Orlek_et_al.
E. coli	NZ_CP009874.1	Plasmid	Orlek_et_al.
E. coli	NZ_CP009875.1	Plasmid	Orlek_et_al.
E. coli	NZ_CP009877.1	Plasmid	Orlek_et_al.
E. coli	NZ_CP009878.1	Plasmid	Orlek_et_al.
E. coli	NZ_CP009879.1	Plasmid	Orlek_et_al.
E. coli	NZ_CP009881.1	Plasmid	Orlek_et_al.
E. coli	NZ_CP009882.1	Plasmid	Orlek_et_al.
E. coli	NZ_CP009883.1	Plasmid	Orlek_et_al.
E. coli	NZ_CP009884.1	Plasmid	Orlek_et_al.
E. coli	NZ_CP009903.1	Plasmid	Orlek_et_al.
E. coli	NZ_CP009904.1	Plasmid	Orlek_et_al.
E. coli	NZ_CP009905.1	Plasmid	Orlek_et_al.
E. coli	NZ_CP009934.1	Plasmid	Orlek_et_al.
E. coli	NZ_CP009936.1	Plasmid	Orlek_et_al.
E. coli	NZ_CP009937.1	Plasmid	Orlek_et_al.
E. coli	NZ_CP009971.1	Plasmid	Orlek_et_al.
E. coli	NZ_CP009972.1	Plasmid	Orlek_et_al.

E. coli	NZ_CP009988.1	Plasmid	Orlek_et_al.
E. coli	NZ_CP009989.1	Plasmid	Orlek_et_al.
E. coli	NZ_CP009990.1	Plasmid	Orlek_et_al.
E. coli	NZ_CP009993.1	Plasmid	Orlek_et_al.
E. coli	NZ_CP009994.1	Plasmid	Orlek_et_al.
E. coli	NZ_CP009995.1	Plasmid	Orlek_et_al.
E. coli	NZ_CP010020.1	Plasmid	Orlek_et_al.
E. coli	NZ_CP010021.1	Plasmid	Orlek_et_al.
E. coli	NZ_CP010022.1	Plasmid	Orlek_et_al.
E. coli	NZ_CP010068.1	Plasmid	Orlek_et_al.
E. coli	NZ_CP010069.1	Plasmid	Orlek_et_al.
E. coli	NZ_CP010246.1	Plasmid	Orlek_et_al.
E. coli	NZ_CP010294.1	Plasmid	Orlek_et_al.
E. coli	NZ_CP010305.1	Plasmid	Orlek_et_al.
E. coli	NZ_CP010316.1	Plasmid	Orlek_et_al.
E. coli	NZ_CP010317.1	Plasmid	Orlek_et_al.
E. coli	NZ_CP010318.1	Plasmid	Orlek_et_al.
E. coli	NZ_CP010325.1	Plasmid	Orlek_et_al.
E. coli	NZ_CP010326.1	Plasmid	Orlek_et_al.
E. coli	NZ_CP010360.2	Plasmid	Orlek_et_al.
E. coli	NZ_CP010362.1	Plasmid	Orlek_et_al.
E. coli	NZ_CP010363.1	Plasmid	Orlek_et_al.
E. coli	NZ_CP010364.1	Plasmid	Orlek_et_al.
E. coli	NZ_CP010365.1	Plasmid	Orlek_et_al.
E. coli	NZ_CP010366.2	Plasmid	Orlek_et_al.
E. coli	NZ_CP010367.1	Plasmid	Orlek_et_al.
E. coli	NZ_CP010372.1	Plasmid	Orlek_et_al.
E. coli	NZ_CP010373.2	Plasmid	Orlek_et_al.
E. coli	NZ_CP010374.1	Plasmid	Orlek_et_al.
E. coli	NZ_CP010375.2	Plasmid	Orlek_et_al.
E. coli	NZ_CP010378.1	Plasmid	Orlek_et_al.
E. coli	NZ_CP010379.1	Plasmid	Orlek_et_al.
E. coli	NZ_CP010380.1	Plasmid	Orlek_et_al.
E. coli	NZ_CP010381.1	Plasmid	Orlek_et_al.
E. coli	NZ_CP010382.1	Plasmid	Orlek_et_al.
E. coli	NZ_CP010383.2	Plasmid	Orlek_et_al.
E. coli	NZ_CP010385.1	Plasmid	Orlek_et_al.
E. coli	NZ_CP010386.1	Plasmid	Orlek_et_al.
E. coli	NZ_CP010387.1	Plasmid	Orlek_et_al.
E. coli	NZ_CP010388.1	Plasmid	Orlek_et_al.
E. coli	NZ_CP010389.1	Plasmid	Orlek_et_al.
E. coli	NZ_CP010390.1	Plasmid	Orlek_et_al.
E. coli	NZ_CP010391.1	Plasmid	Orlek_et_al.
E. coli	NZ_CP010393.1	Plasmid	Orlek_et_al.
E. coli	NZ_CP010394.1	Plasmid	Orlek_et_al.
E. coli	NZ_CP010395.1	Plasmid	Orlek_et_al.
E. coli	NZ_CP010396.1	Plasmid	Orlek_et_al.
E. coli	NZ_CP010830.1	Plasmid	Orlek_et_al.
E. coli	NZ_CP010831.1	Plasmid	Orlek_et_al.
E. coli	NZ_CP010832.1	Plasmid	Orlek_et_al.
E. coli	NZ_CP010877.1	Plasmid	Orlek_et_al.
E. coli	NZ_CP010878.1	Plasmid	Orlek_et_al.
E. coli	NZ_CP010879.1	Plasmid	Orlek_et_al.
E. coli	NZ_CP010880.1	Plasmid	Orlek_et_al.
E. coli	NZ_CP010881.1	Plasmid	Orlek_et_al.
E. coli	NZ_CP010882.1	Plasmid	Orlek_et_al.
E. coli	NZ_CP010908.1	Plasmid	Orlek_et_al.
E. coli	NZ_CP010909.1	Plasmid	Orlek_et_al.
E. coli	NZ_CP010910.1	Plasmid	Orlek_et_al.
E. coli	NZ_CP010911.1	Plasmid	Orlek_et_al.
E. coli	NZ_CP011019.1	Plasmid	Orlek_et_al.
E. coli	NZ_CP011048.1	Plasmid	Orlek_et_al.
E. coli	NZ_CP011049.1	Plasmid	Orlek_et_al.
E. coli	NZ_CP011050.1	Plasmid	Orlek_et_al.
E. coli	NZ_CP011062.1	Plasmid	Orlek_et_al.
E. coli	NZ_CP011063.1	Plasmid	Orlek_et_al.
E. coli	NZ_CP011064.1	Plasmid	Orlek_et_al.
E. coli	NZ_CP011065.1	Plasmid	Orlek_et_al.
E. coli	NZ_CP011066.1	Plasmid	Orlek_et_al.
E. coli	NZ_CP011067.1	Plasmid	Orlek_et_al.
E. coli	NZ_CP011133.1	Plasmid	Orlek_et_al.
E. coli	NZ_CP011135.1	Plasmid	Orlek_et_al.

E. coli	NZ_CP011136.1	Plasmid	Orlek_et_al.
E. coli	NZ_CP011137.1	Plasmid	Orlek_et_al.
E. coli	NZ_CP011138.1	Plasmid	Orlek_et_al.
E. coli	NZ_CP011139.1	Plasmid	Orlek_et_al.
E. coli	NZ_CP011140.1	Plasmid	Orlek_et_al.
E. coli	NZ_CP011141.1	Plasmid	Orlek_et_al.
E. coli	NZ_CP011142.1	Plasmid	Orlek_et_al.
E. coli	NZ_CP011143.1	Plasmid	Orlek_et_al.
E. coli	NZ_CP011287.1	Plasmid	Orlek_et_al.
E. coli	NZ_CP011290.1	Plasmid	Orlek_et_al.
E. coli	NZ_CP011291.1	Plasmid	Orlek_et_al.
E. coli	NZ_CP011294.1	Plasmid	Orlek_et_al.
E. coli	NZ_CP011314.1	Plasmid	Orlek_et_al.
E. coli	NZ_CP011315.1	Plasmid	Orlek_et_al.
E. coli	NZ_CP011316.1	Plasmid	Orlek_et_al.
E. coli	NZ_CP011332.1	Plasmid	Orlek_et_al.
E. coli	NZ_CP011333.1	Plasmid	Orlek_et_al.
E. coli	NZ_CP011334.1	Plasmid	Orlek_et_al.
E. coli	NZ_CP011335.1	Plasmid	Orlek_et_al.
E. coli	NZ_CP011336.1	Plasmid	Orlek_et_al.
E. coli	NZ_CP011337.1	Plasmid	Orlek_et_al.
E. coli	NZ_CP011338.1	Plasmid	Orlek_et_al.
E. coli	NZ_CP011395.1	Plasmid	Orlek_et_al.
E. coli	NZ_CP011417.1	Plasmid	Orlek_et_al.
E. coli	NZ_CP011418.1	Plasmid	Orlek_et_al.
E. coli	NZ_CP011429.1	Plasmid	Orlek_et_al.
E. coli	NZ_CP011430.1	Plasmid	Orlek_et_al.
E. coli	NZ_CP011431.1	Plasmid	Orlek_et_al.
E. coli	NZ_CP011432.1	Plasmid	Orlek_et_al.
E. coli	NZ_CP011433.1	Plasmid	Orlek_et_al.
E. coli	NZ_CP011434.1	Plasmid	Orlek_et_al.
E. coli	NZ_CP011435.1	Plasmid	Orlek_et_al.
E. coli	NZ_CP011493.1	Plasmid	Orlek_et_al.
E. coli	NZ_CP011496.1	Plasmid	Orlek_et_al.
E. coli	NZ_CP011540.1	Plasmid	Orlek_et_al.
E. coli	NZ_CP011569.1	Plasmid	Orlek_et_al.
E. coli	NZ_CP011571.1	Plasmid	Orlek_et_al.
E. coli	NZ_CP011573.1	Plasmid	Orlek_et_al.
E. coli	NZ_CP011575.1	Plasmid	Orlek_et_al.
E. coli	NZ_CP011576.1	Plasmid	Orlek_et_al.
E. coli	NZ_CP011577.1	Plasmid	Orlek_et_al.
E. coli	NZ_CP011579.1	Plasmid	Orlek_et_al.
E. coli	NZ_CP011580.1	Plasmid	Orlek_et_al.
E. coli	NZ_CP011582.1	Plasmid	Orlek_et_al.
E. coli	NZ_CP011583.1	Plasmid	Orlek_et_al.
E. coli	NZ_CP011585.1	Plasmid	Orlek_et_al.
E. coli	NZ_CP011586.1	Plasmid	Orlek_et_al.
E. coli	NZ_CP011587.1	Plasmid	Orlek_et_al.
E. coli	NZ_CP011588.1	Plasmid	Orlek_et_al.
E. coli	NZ_CP011589.1	Plasmid	Orlek_et_al.
E. coli	NZ_CP011590.1	Plasmid	Orlek_et_al.
E. coli	NZ_CP011592.1	Plasmid	Orlek_et_al.
E. coli	NZ_CP011593.1	Plasmid	Orlek_et_al.
E. coli	NZ_CP011594.1	Plasmid	Orlek_et_al.
E. coli	NZ_CP011595.1	Plasmid	Orlek_et_al.
E. coli	NZ_CP011596.1	Plasmid	Orlek_et_al.
E. coli	NZ_CP011598.1	Plasmid	Orlek_et_al.
E. coli	NZ_CP011599.1	Plasmid	Orlek_et_al.
E. coli	NZ_CP011600.1	Plasmid	Orlek_et_al.
E. coli	NZ_CP011601.1	Plasmid	Orlek_et_al.
E. coli	NZ_CP011603.1	Plasmid	Orlek_et_al.
E. coli	NZ_CP011604.1	Plasmid	Orlek_et_al.
E. coli	NZ_CP011605.1	Plasmid	Orlek_et_al.
E. coli	NZ_CP011606.1	Plasmid	Orlek_et_al.
E. coli	NZ_CP011607.1	Plasmid	Orlek_et_al.
E. coli	NZ_CP011608.1	Plasmid	Orlek_et_al.
E. coli	NZ_CP011609.1	Plasmid	Orlek_et_al.
E. coli	NZ_CP011610.1	Plasmid	Orlek_et_al.
E. coli	NZ_CP011611.1	Plasmid	Orlek_et_al.
E. coli	NZ_CP011613.1	Plasmid	Orlek_et_al.
E. coli	NZ_CP011614.1	Plasmid	Orlek_et_al.
E. coli	NZ_CP011616.1	Plasmid	Orlek_et_al.

E. coli	NZ_CP011617.1	Plasmid	Orlek_et_al.
E. coli	NZ_CP011620.1	Plasmid	Orlek_et_al.
E. coli	NZ_CP011622.1	Plasmid	Orlek_et_al.
E. coli	NZ_CP011623.1	Plasmid	Orlek_et_al.
E. coli	NZ_CP011625.1	Plasmid	Orlek_et_al.
E. coli	NZ_CP011626.1	Plasmid	Orlek_et_al.
E. coli	NZ_CP011627.1	Plasmid	Orlek_et_al.
E. coli	NZ_CP011628.1	Plasmid	Orlek_et_al.
E. coli	NZ_CP011630.1	Plasmid	Orlek_et_al.
E. coli	NZ_CP011631.1	Plasmid	Orlek_et_al.
E. coli	NZ_CP011632.1	Plasmid	Orlek_et_al.
E. coli	NZ_CP011633.1	Plasmid	Orlek_et_al.
E. coli	NZ_CP011634.1	Plasmid	Orlek_et_al.
E. coli	NZ_CP011635.1	Plasmid	Orlek_et_al.
E. coli	NZ_CP011637.1	Plasmid	Orlek_et_al.
E. coli	NZ_CP011638.1	Plasmid	Orlek_et_al.
E. coli	NZ_CP011639.1	Plasmid	Orlek_et_al.
E. coli	NZ_CP011640.1	Plasmid	Orlek_et_al.
E. coli	NZ_CP011641.1	Plasmid	Orlek_et_al.
E. coli	NZ_CP011643.1	Plasmid	Orlek_et_al.
E. coli	NZ_CP011644.1	Plasmid	Orlek_et_al.
E. coli	NZ_CP011645.1	Plasmid	Orlek_et_al.
E. coli	NZ_CP011646.1	Plasmid	Orlek_et_al.
E. coli	NZ_CP011649.1	Plasmid	Orlek_et_al.
E. coli	NZ_CP011651.1	Plasmid	Orlek_et_al.
E. coli	NZ_CP011652.1	Plasmid	Orlek_et_al.
E. coli	NZ_CP011653.1	Plasmid	Orlek_et_al.
E. coli	NZ_CP011654.1	Plasmid	Orlek_et_al.
E. coli	NZ_CP011655.1	Plasmid	Orlek_et_al.
E. coli	NZ_CP011656.1	Plasmid	Orlek_et_al.
E. coli	NZ_CP011864.1	Plasmid	Orlek_et_al.
E. coli	NZ_CP011865.1	Plasmid	Orlek_et_al.
E. coli	NZ_CP011866.1	Plasmid	Orlek_et_al.
E. coli	NZ_CP011867.1	Plasmid	Orlek_et_al.
E. coli	NZ_CP011943.1	Plasmid	Orlek_et_al.
E. coli	NZ_CP011944.1	Plasmid	Orlek_et_al.
E. coli	NZ_CP011945.1	Plasmid	Orlek_et_al.
E. coli	NZ_CP011977.1	Plasmid	Orlek_et_al.
E. coli	NZ_CP011978.1	Plasmid	Orlek_et_al.
E. coli	NZ_CP011979.1	Plasmid	Orlek_et_al.
E. coli	NZ_CP011981.1	Plasmid	Orlek_et_al.
E. coli	NZ_CP011982.1	Plasmid	Orlek_et_al.
E. coli	NZ_CP011983.1	Plasmid	Orlek_et_al.
E. coli	NZ_CP011984.1	Plasmid	Orlek_et_al.
E. coli	NZ_CP011986.1	Plasmid	Orlek_et_al.
E. coli	NZ_CP011987.1	Plasmid	Orlek_et_al.
E. coli	NZ_CP011988.1	Plasmid	Orlek_et_al.
E. coli	NZ_CP011990.1	Plasmid	Orlek_et_al.
E. coli	NZ_CP011991.1	Plasmid	Orlek_et_al.
E. coli	NZ_CP011993.1	Plasmid	Orlek_et_al.
E. coli	NZ_CP012039.1	Plasmid	Orlek_et_al.
E. coli	NZ_CP012138.1	Plasmid	Orlek_et_al.
E. coli	NZ_CP012139.1	Plasmid	Orlek_et_al.
E. coli	NZ_CP012141.1	Plasmid	Orlek_et_al.
E. coli	NZ_CP012142.1	Plasmid	Orlek_et_al.
E. coli	NZ_CP012143.1	Plasmid	Orlek_et_al.
E. coli	NZ_CP012163.1	Plasmid	Orlek_et_al.
E. coli	NZ_CP012164.1	Plasmid	Orlek_et_al.
E. coli	NZ_CP012166.1	Plasmid	Orlek_et_al.
E. coli	NZ_CP012168.1	Plasmid	Orlek_et_al.
E. coli	NZ_CP012169.1	Plasmid	Orlek_et_al.
E. coli	NZ_CP012170.1	Plasmid	Orlek_et_al.
E. coli	NZ_CP012254.1	Plasmid	Orlek_et_al.
E. coli	NZ_CP012255.1	Plasmid	Orlek_et_al.
E. coli	NZ_CP012256.1	Plasmid	Orlek_et_al.
E. coli	NZ_CP012258.1	Plasmid	Orlek_et_al.
E. coli	NZ_CP012265.1	Plasmid	Orlek_et_al.
E. coli	NZ_CP012267.1	Plasmid	Orlek_et_al.
E. coli	NZ_CP012345.1	Plasmid	Orlek_et_al.
E. coli	NZ_CP012348.1	Plasmid	Orlek_et_al.
E. coli	NZ_CP012397.1	Plasmid	Orlek_et_al.
E. coli	NZ_CP012488.1	Plasmid	Orlek_et_al.

E. coli	NZ_CP012489.1	Plasmid	Orlek_et_al.
E. coli	NZ_CP012490.1	Plasmid	Orlek_et_al.
E. coli	NZ_CP012498.1	Plasmid	Orlek_et_al.
E. coli	NZ_CP012499.1	Plasmid	Orlek_et_al.
E. coli	NZ_CP012500.1	Plasmid	Orlek_et_al.
E. coli	NZ_CP012501.1	Plasmid	Orlek_et_al.
E. coli	NZ_CP012626.1	Plasmid	Orlek_et_al.
E. coli	NZ_CP012627.1	Plasmid	Orlek_et_al.
E. coli	NZ_CP012628.1	Plasmid	Orlek_et_al.
E. coli	NZ_CP012629.1	Plasmid	Orlek_et_al.
E. coli	NZ_CP012630.1	Plasmid	Orlek_et_al.
E. coli	NZ_CP012632.1	Plasmid	Orlek_et_al.
E. coli	NZ_CP012634.1	Plasmid	Orlek_et_al.
E. coli	NZ_CP012636.1	Plasmid	Orlek_et_al.
E. coli	NZ_CP012637.1	Plasmid	Orlek_et_al.
E. coli	NZ_CP012638.1	Plasmid	Orlek_et_al.
E. coli	NZ_CP012682.1	Plasmid	Orlek_et_al.
E. coli	NZ_CP012683.1	Plasmid	Orlek_et_al.
E. coli	NZ_CP012684.1	Plasmid	Orlek_et_al.
E. coli	NZ_CP012686.1	Plasmid	Orlek_et_al.
E. coli	NZ_CP012732.1	Plasmid	Orlek_et_al.
E. coli	NZ_CP012733.1	Plasmid	Orlek_et_al.
E. coli	NZ_CP012734.1	Plasmid	Orlek_et_al.
E. coli	NZ_CP012736.1	Plasmid	Orlek_et_al.
E. coli	NZ_CP012754.1	Plasmid	Orlek_et_al.
E. coli	NZ_CP012755.1	Plasmid	Orlek_et_al.
E. coli	NZ_CP012803.1	Plasmid	Orlek_et_al.
E. coli	NZ_CP012834.1	Plasmid	Orlek_et_al.
E. coli	NZ_CP012835.1	Plasmid	Orlek_et_al.
E. coli	NZ_CP012884.1	Plasmid	Orlek_et_al.
E. coli	NZ_CP012923.1	Plasmid	Orlek_et_al.
E. coli	NZ_CP012926.1	Plasmid	Orlek_et_al.
E. coli	NZ_CP012928.1	Plasmid	Orlek_et_al.
E. coli	NZ_CP012929.1	Plasmid	Orlek_et_al.
E. coli	NZ_CP012931.1	Plasmid	Orlek_et_al.
E. coli	NZ_CP012932.1	Plasmid	Orlek_et_al.
E. coli	NZ_CP012933.1	Plasmid	Orlek_et_al.
E. coli	NZ_CP012934.1	Plasmid	Orlek_et_al.
E. coli	NZ_CP012935.1	Plasmid	Orlek_et_al.
E. coli	NZ_CP012936.1	Plasmid	Orlek_et_al.
E. coli	NZ_CP012988.3	Plasmid	Orlek_et_al.
E. coli	NZ_CP012989.1	Plasmid	Orlek_et_al.
E. coli	NZ_CP012990.1	Plasmid	Orlek_et_al.
E. coli	NZ_CP012993.2	Plasmid	Orlek_et_al.
E. coli	NZ_CP012994.1	Plasmid	Orlek_et_al.
E. coli	NZ_CP012995.1	Plasmid	Orlek_et_al.
E. coli	NZ_CP013028.1	Plasmid	Orlek_et_al.
E. coli	NZ_CP013030.1	Plasmid	Orlek_et_al.
E. coli	NZ_CP013047.1	Plasmid	Orlek_et_al.
E. coli	NZ_CP013098.1	Plasmid	Orlek_et_al.
E. coli	NZ_CP013221.1	Plasmid	Orlek_et_al.
E. coli	NZ_CP013223.1	Plasmid	Orlek_et_al.
E. coli	NZ_CP013224.1	Plasmid	Orlek_et_al.
E. coli	NZ_CP013225.1	Plasmid	Orlek_et_al.
E. coli	NZ_CP013321.1	Plasmid	Orlek_et_al.
E. coli	NZ_CP013323.1	Plasmid	Orlek_et_al.
E. coli	NZ_CP013324.1	Plasmid	Orlek_et_al.
E. coli	NZ_CP013325.1	Plasmid	Orlek_et_al.
E. coli	NZ_CP013326.1	Plasmid	Orlek_et_al.
E. coli	NZ_CP013339.1	Plasmid	Orlek_et_al.
E. coli	NZ_CP013340.1	Plasmid	Orlek_et_al.
E. coli	NZ_CP013712.1	Plasmid	Orlek_et_al.
E. coli	NZ_CP013713.1	Plasmid	Orlek_et_al.
E. coli	NZ_CP013832.1	Plasmid	Orlek_et_al.
E. coli	NZ_CP013833.1	Plasmid	Orlek_et_al.
E. coli	NZ_CP013834.1	Plasmid	Orlek_et_al.
E. coli	NZ_CP013914.1	Plasmid	Orlek_et_al.
E. coli	NZ_CP013915.1	Plasmid	Orlek_et_al.
E. coli	NZ_CP013941.1	Plasmid	Orlek_et_al.
E. coli	NZ_CP013942.1	Plasmid	Orlek_et_al.
E. coli	NZ_CP014005.1	Plasmid	Orlek_et_al.
E. coli	NZ_CP014006.1	Plasmid	Orlek_et_al.

E. coli	NZ_CP014009.1	Plasmid	Orlek_et_al.
E. coli	NZ_CP014011.1	Plasmid	Orlek_et_al.
E. coli	NZ_CP014050.1	Plasmid	Orlek_et_al.
E. coli	NZ_CP014096.1	Plasmid	Orlek_et_al.
E. coli	NZ_CP014098.1	Plasmid	Orlek_et_al.
E. coli	NZ_CP014100.1	Plasmid	Orlek_et_al.
E. coli	NZ_CP014101.1	Plasmid	Orlek_et_al.
E. coli	NZ_CP014126.1	Plasmid	Orlek_et_al.
E. coli	NZ_CP014127.1	Plasmid	Orlek_et_al.
E. coli	NZ_CP014128.1	Plasmid	Orlek_et_al.
E. coli	NZ_CP014198.2	Plasmid	Orlek_et_al.
E. coli	NZ_CP014199.1	Plasmid	Orlek_et_al.
E. coli	NZ_CP014200.1	Plasmid	Orlek_et_al.
E. coli	NZ_CP014208.1	Plasmid	Orlek_et_al.
E. coli	NZ_CP014271.1	Plasmid	Orlek_et_al.
E. coli	NZ_CP014273.1	Plasmid	Orlek_et_al.
E. coli	NZ_CP014305.2	Plasmid	Orlek_et_al.
E. coli	NZ_CP014357.1	Plasmid	Orlek_et_al.
E. coli	NZ_CP014359.1	Plasmid	Orlek_et_al.
E. coli	NZ_CP014489.1	Plasmid	Orlek_et_al.
E. coli	NZ_CP014490.1	Plasmid	Orlek_et_al.
E. coli	NZ_CP014491.1	Plasmid	Orlek_et_al.
E. coli	NZ_CP014493.1	Plasmid	Orlek_et_al.
E. coli	NZ_CP014494.1	Plasmid	Orlek_et_al.
E. coli	NZ_CP014496.1	Plasmid	Orlek_et_al.
E. coli	NZ_CP014498.1	Plasmid	Orlek_et_al.
E. coli	NZ_CP014523.1	Plasmid	Orlek_et_al.
E. coli	NZ_CP014524.1	Plasmid	Orlek_et_al.
E. coli	NZ_CP014537.1	Plasmid	Orlek_et_al.
E. coli	NZ_CP014577.1	Plasmid	Orlek_et_al.
E. coli	NZ_CP014622.1	Plasmid	Orlek_et_al.
E. coli	NZ_CP014648.1	Plasmid	Orlek_et_al.
E. coli	NZ_CP014649.1	Plasmid	Orlek_et_al.
E. coli	NZ_CP014650.1	Plasmid	Orlek_et_al.
E. coli	NZ_CP014658.1	Plasmid	Orlek_et_al.
E. coli	NZ_CP014660.1	Plasmid	Orlek_et_al.
E. coli	NZ_CP014662.1	Plasmid	Orlek_et_al.
E. coli	NZ_CP014697.1	Plasmid	Orlek_et_al.
E. coli	NZ_CP014698.1	Plasmid	Orlek_et_al.
E. coli	NZ_CP014707.1	Plasmid	Orlek_et_al.
E. coli	NZ_CP014756.1	Plasmid	Orlek_et_al.
E. coli	NZ_CP014757.1	Plasmid	Orlek_et_al.
E. coli	NZ_CP014758.1	Plasmid	Orlek_et_al.
E. coli	NZ_CP014763.1	Plasmid	Orlek_et_al.
E. coli	NZ_CP014764.1	Plasmid	Orlek_et_al.
E. coli	NZ_CP014765.1	Plasmid	Orlek_et_al.
E. coli	NZ_CP014966.1	Plasmid	Orlek_et_al.
E. coli	NZ_CP014968.1	Plasmid	Orlek_et_al.
E. coli	NZ_CP014970.1	Plasmid	Orlek_et_al.
E. coli	NZ_CP014972.1	Plasmid	Orlek_et_al.
E. coli	NZ_CP014973.1	Plasmid	Orlek_et_al.
E. coli	NZ_CP014974.1	Plasmid	Orlek_et_al.
E. coli	NZ_CP014976.1	Plasmid	Orlek_et_al.
E. coli	NZ_CP014978.1	Plasmid	Orlek_et_al.
E. coli	NZ_CP014980.1	Plasmid	Orlek_et_al.
E. coli	NZ_CP014995.1	Plasmid	Orlek_et_al.
E. coli	NZ_CP014997.1	Plasmid	Orlek_et_al.
E. coli	NZ_CP015021.1	Plasmid	Orlek_et_al.
E. coli	NZ_CP015022.1	Plasmid	Orlek_et_al.
E. coli	NZ_CP015026.1	Plasmid	Orlek_et_al.
E. coli	NZ_CP015070.1	Plasmid	Orlek_et_al.
E. coli	NZ_CP015071.1	Plasmid	Orlek_et_al.
E. coli	NZ_CP015072.1	Plasmid	Orlek_et_al.
E. coli	NZ_CP015073.1	Plasmid	Orlek_et_al.
E. coli	NZ_CP015075.2	Plasmid	Orlek_et_al.
E. coli	NZ_CP015077.1	Plasmid	Orlek_et_al.
E. coli	NZ_CP015078.1	Plasmid	Orlek_et_al.
E. coli	NZ_CP015119.1	Plasmid	Orlek_et_al.
E. coli	NZ_CP015131.1	Plasmid	Orlek_et_al.
E. coli	NZ_CP015132.1	Plasmid	Orlek_et_al.
E. coli	NZ_CP015133.1	Plasmid	Orlek_et_al.
E. coli	NZ_CP015139.1	Plasmid	Orlek_et_al.

E. coli	NZ_CP015140.1	Plasmid	Orlek_et_al.
E. coli	NZ_CP015141.1	Plasmid	Orlek_et_al.
E. coli	NZ_CP015142.1	Plasmid	Orlek_et_al.
E. coli	NZ_CP015143.1	Plasmid	Orlek_et_al.
E. coli	NZ_CP015158.1	Plasmid	Orlek_et_al.
E. coli	NZ_CP015239.1	Plasmid	Orlek_et_al.
E. coli	NZ_CP015242.1	Plasmid	Orlek_et_al.
E. coli	NZ_CP015380.1	Plasmid	Orlek_et_al.
E. coli	NZ_CP015501.1	Plasmid	Orlek_et_al.
E. coli	NZ_CP015502.1	Plasmid	Orlek_et_al.
E. coli	NZ_CP015503.1	Plasmid	Orlek_et_al.
E. coli	NZ_CP015504.1	Plasmid	Orlek_et_al.
E. coli	NZ_CP015505.1	Plasmid	Orlek_et_al.
E. coli	NZ_CP015725.1	Plasmid	Orlek_et_al.
E. coli	NZ_CP015754.1	Plasmid	Orlek_et_al.
E. coli	NZ_CP015755.1	Plasmid	Orlek_et_al.
E. coli	NZ_CP015816.1	Plasmid	Orlek_et_al.
E. coli	NZ_CP015823.1	Plasmid	Orlek_et_al.
E. coli	NZ_CP015824.1	Plasmid	Orlek_et_al.
E. coli	NZ_CP015825.1	Plasmid	Orlek_et_al.
E. coli	NZ_CP015833.1	Plasmid	Orlek_et_al.
E. coli	NZ_CP015844.1	Plasmid	Orlek_et_al.
E. coli	NZ_CP015845.1	Plasmid	Orlek_et_al.
E. coli	NZ_CP015856.1	Plasmid	Orlek_et_al.
E. coli	NZ_CP015913.1	Plasmid	Orlek_et_al.
E. coli	NZ_CP015914.1	Plasmid	Orlek_et_al.
E. coli	NZ_CP015915.1	Plasmid	Orlek_et_al.
E. coli	NZ_CP015916.1	Plasmid	Orlek_et_al.
E. coli	NZ_CP015917.1	Plasmid	Orlek_et_al.
E. coli	NZ_CP015991.1	Plasmid	Orlek_et_al.
E. coli	NZ_CP015996.1	Plasmid	Orlek_et_al.
E. coli	NZ_CP015997.1	Plasmid	Orlek_et_al.
E. coli	NZ_CP016035.1	Plasmid	Orlek_et_al.
E. coli	NZ_CP016036.1	Plasmid	Orlek_et_al.
E. coli	NZ_CP016038.1	Plasmid	Orlek_et_al.
E. coli	NZ_CP016039.1	Plasmid	Orlek_et_al.
E. coli	NZ_CP016040.1	Plasmid	Orlek_et_al.
E. coli	NZ_CP016041.1	Plasmid	Orlek_et_al.
E. coli	NZ_CP016042.1	Plasmid	Orlek_et_al.
E. coli	NZ_CP016160.1	Plasmid	Orlek_et_al.
E. coli	NZ_CP016161.1	Plasmid	Orlek_et_al.
E. coli	NZ_CP016274.1	Plasmid	Orlek_et_al.
E. coli	NZ_CP016275.1	Plasmid	Orlek_et_al.
E. coli	NZ_CP016276.1	Plasmid	Orlek_et_al.
E. coli	NZ_CP016498.1	Plasmid	Orlek_et_al.
E. coli	NZ_CP016508.1	Plasmid	Orlek_et_al.
E. coli	NZ_CP016516.1	Plasmid	Orlek_et_al.
E. coli	NZ_CP016518.1	Plasmid	Orlek_et_al.
E. coli	NZ_CP016519.1	Plasmid	Orlek_et_al.
E. coli	NZ_CP016520.1	Plasmid	Orlek_et_al.
E. coli	NZ_CP016522.1	Plasmid	Orlek_et_al.
E. coli	NZ_CP016526.1	Plasmid	Orlek_et_al.
E. coli	NZ_CP016527.1	Plasmid	Orlek_et_al.
E. coli	NZ_CP016528.1	Plasmid	Orlek_et_al.
E. coli	NZ_CP016529.1	Plasmid	Orlek_et_al.
E. coli	NZ_CP016532.1	Plasmid	Orlek_et_al.
E. coli	NZ_CP016533.1	Plasmid	Orlek_et_al.
E. coli	NZ_CP016547.1	Plasmid	Orlek_et_al.
E. coli	NZ_CP016548.1	Plasmid	Orlek_et_al.
E. coli	NZ_CP016549.1	Plasmid	Orlek_et_al.
E. coli	NZ_CP016550.1	Plasmid	Orlek_et_al.
E. coli	NZ_CP016551.1	Plasmid	Orlek_et_al.
E. coli	NZ_CP016566.1	Plasmid	Orlek_et_al.
E. coli	NZ_CP016567.1	Plasmid	Orlek_et_al.
E. coli	NZ_CP016568.1	Plasmid	Orlek_et_al.
E. coli	NZ_CP016572.1	Plasmid	Orlek_et_al.
E. coli	NZ_CP016575.1	Plasmid	Orlek_et_al.
E. coli	NZ_CP016582.1	Plasmid	Orlek_et_al.
E. coli	NZ_CP016583.1	Plasmid	Orlek_et_al.
E. coli	NZ_CP016584.1	Plasmid	Orlek_et_al.
E. coli	NZ_CP016585.1	Plasmid	Orlek_et_al.
E. coli	NZ_CP016587.1	Plasmid	Orlek_et_al.

E. coli	NZ_CP016763.1	Plasmid	Orlek_et_al.
E. coli	NZ_CP016764.1	Plasmid	Orlek_et_al.
E. coli	NZ_CP016765.1	Plasmid	Orlek_et_al.
E. coli	NZ_CP016810.1	Plasmid	Orlek_et_al.
E. coli	NZ_CP016812.1	Plasmid	Orlek_et_al.
E. coli	NZ_FO704549.1	Plasmid	Orlek_et_al.
E. coli	NZ_FO818638.1	Plasmid	Orlek_et_al.
E. coli	NZ_FO818639.1	Plasmid	Orlek_et_al.
E. coli	NZ_FO834904.1	Plasmid	Orlek_et_al.
E. coli	NZ_FO834905.1	Plasmid	Orlek_et_al.
E. coli	NZ_HG941719.1	Plasmid	Orlek_et_al.
E. coli	NZ_HG941720.1	Plasmid	Orlek_et_al.
E. coli	NZ_LN681228.1	Plasmid	Orlek_et_al.
E. coli	NZ_LN824134.1	Plasmid	Orlek_et_al.
E. coli	NZ_LN824135.1	Plasmid	Orlek_et_al.
E. coli	NZ_LN824137.1	Plasmid	Orlek_et_al.
E. coli	NZ_LN824138.1	Plasmid	Orlek_et_al.
E. coli	NZ_LN890519.1	Plasmid	Orlek_et_al.
E. coli	NZ_LN890521.1	Plasmid	Orlek_et_al.
E. coli	NZ_LN890526.1	Plasmid	Orlek_et_al.
E. coli	NZ_LN907828.1	Plasmid	Orlek_et_al.
E. coli	NZ_LN907829.1	Plasmid	Orlek_et_al.
E. coli	NZ_LN999012.1	Plasmid	Orlek_et_al.
E. coli	NZ_LN999834.1	Plasmid	Orlek_et_al.
E. coli	AB011549.2	Plasmid	CGE_PlasmidFinder
E. coli	AB040415.1	Plasmid	CGE_PlasmidFinder
E. coli	AB255435.1	Plasmid	CGE_PlasmidFinder
E. coli	AB366441.1	Plasmid	CGE_PlasmidFinder
E. coli	AB366442.1	Plasmid	CGE_PlasmidFinder
E. coli	AB576781.2	Plasmid	CGE_PlasmidFinder
E. coli	AB605179.1	Plasmid	CGE_PlasmidFinder
E. coli	AB616660.2	Plasmid	CGE_PlasmidFinder
E. coli	AE006471.2	Plasmid	CGE_PlasmidFinder
E. coli	AE017044.1	Plasmid	CGE_PlasmidFinder
E. coli	AE017045.1	Plasmid	CGE_PlasmidFinder
E. coli	AE017046.1	Plasmid	CGE_PlasmidFinder
E. coli	AF053945.1	Plasmid	CGE_PlasmidFinder
E. coli	AF053947.1	Plasmid	CGE_PlasmidFinder
E. coli	AF074611.1	Plasmid	CGE_PlasmidFinder
E. coli	AF074613.1	Plasmid	CGE_PlasmidFinder
E. coli	AF102990.1	Plasmid	CGE_PlasmidFinder
E. coli	AF135182.4	Plasmid	CGE_PlasmidFinder
E. coli	AF305615.1	Plasmid	CGE_PlasmidFinder
E. coli	AF336309.1	Plasmid	CGE_PlasmidFinder
E. coli	AF348706.1	Plasmid	CGE_PlasmidFinder
E. coli	AF386526.1	Plasmid	CGE_PlasmidFinder
E. coli	AF401292.1	Plasmid	CGE_PlasmidFinder
E. coli	AF497970.1	Plasmid	CGE_PlasmidFinder
E. coli	AF550415.2	Plasmid	CGE_PlasmidFinder
E. coli	AF550679.1	Plasmid	CGE_PlasmidFinder
E. coli	AGTD01000006.1	Plasmid	CGE_PlasmidFinder
E. coli	AJ634602.1	Plasmid	CGE_PlasmidFinder
E. coli	AL513384.1	Plasmid	CGE_PlasmidFinder
E. coli	AM286416.1	Plasmid	CGE_PlasmidFinder
E. coli	AM886293.1	Plasmid	CGE_PlasmidFinder
E. coli	AM901564.1	Plasmid	CGE_PlasmidFinder
E. coli	AM942760.1	Plasmid	CGE_PlasmidFinder
E. coli	AP002527.1	Plasmid	CGE_PlasmidFinder
E. coli	AP004237.1	Plasmid	CGE_PlasmidFinder
E. coli	AP005147.1	Plasmid	CGE_PlasmidFinder
E. coli	AP009242.1	Plasmid	CGE_PlasmidFinder
E. coli	AP009243.1	Plasmid	CGE_PlasmidFinder
E. coli	AP009245.1	Plasmid	CGE_PlasmidFinder
E. coli	AP009246.1	Plasmid	CGE_PlasmidFinder
E. coli	AP009379.1	Plasmid	CGE_PlasmidFinder
E. coli	AP010910.1	Plasmid	CGE_PlasmidFinder
E. coli	AP010954.1	Plasmid	CGE_PlasmidFinder
E. coli	AP010955.1	Plasmid	CGE_PlasmidFinder
E. coli	AP010959.1	Plasmid	CGE_PlasmidFinder
E. coli	AP010962.1	Plasmid	CGE_PlasmidFinder
E. coli	AP010963.1	Plasmid	CGE_PlasmidFinder
E. coli	AP011954.1	Plasmid	CGE_PlasmidFinder

E. coli	AP011958.1	Plasmid	CGE_PlasmidFinder
E. coli	AP012208.1	Plasmid	CGE_PlasmidFinder
E. coli	AY079200.1	Plasmid	CGE_PlasmidFinder
E. coli	AY150843.2	Plasmid	CGE_PlasmidFinder
E. coli	AY167049.1	Plasmid	CGE_PlasmidFinder
E. coli	AY214164.3	Plasmid	CGE_PlasmidFinder
E. coli	AY333434.1	Plasmid	CGE_PlasmidFinder
E. coli	AY360321.1	Plasmid	CGE_PlasmidFinder
E. coli	AY422214.1	Plasmid	CGE_PlasmidFinder
E. coli	AY458016.1	Plasmid	CGE_PlasmidFinder
E. coli	AY509003.1	Plasmid	CGE_PlasmidFinder
E. coli	AY509004.1	Plasmid	CGE_PlasmidFinder
E. coli	AY517905.1	Plasmid	CGE_PlasmidFinder
E. coli	AY522431.4	Plasmid	CGE_PlasmidFinder
E. coli	AY543071.1	Plasmid	CGE_PlasmidFinder
E. coli	AY545598.5	Plasmid	CGE_PlasmidFinder
E. coli	AY879342.1	Plasmid	CGE_PlasmidFinder
E. coli	AY913943.1	Plasmid	CGE_PlasmidFinder
E. coli	AY929248.1	Plasmid	CGE_PlasmidFinder
E. coli	BR000038.1	Plasmid	CGE_PlasmidFinder
E. coli	BX936400.1	Plasmid	CGE_PlasmidFinder
E. coli	CP000035.1	Plasmid	CGE_PlasmidFinder
E. coli	CP000037.1	Plasmid	CGE_PlasmidFinder
E. coli	CP000039.1	Plasmid	CGE_PlasmidFinder
E. coli	CP000244.1	Plasmid	CGE_PlasmidFinder
E. coli	CP000306.1	Plasmid	CGE_PlasmidFinder
E. coli	CP000309.1	Plasmid	CGE_PlasmidFinder
E. coli	CP000602.1	Plasmid	CGE_PlasmidFinder
E. coli	CP000603.1	Plasmid	CGE_PlasmidFinder
E. coli	CP000604.1	Plasmid	CGE_PlasmidFinder
E. coli	CP000648.1	Plasmid	CGE_PlasmidFinder
E. coli	CP000649.1	Plasmid	CGE_PlasmidFinder
E. coli	CP000650.1	Plasmid	CGE_PlasmidFinder
E. coli	CP000836.1	Plasmid	CGE_PlasmidFinder
E. coli	CP000858.1	Plasmid	CGE_PlasmidFinder
E. coli	CP000913.1	Plasmid	CGE_PlasmidFinder
E. coli	CP000965.1	Plasmid	CGE_PlasmidFinder
E. coli	CP000966.1	Plasmid	CGE_PlasmidFinder
E. coli	CP000973.1	Plasmid	CGE_PlasmidFinder
E. coli	CP001059.1	Plasmid	CGE_PlasmidFinder
E. coli	CP001064.1	Plasmid	CGE_PlasmidFinder
E. coli	CP001065.1	Plasmid	CGE_PlasmidFinder
E. coli	CP001122.1	Plasmid	CGE_PlasmidFinder
E. coli	CP001123.1	Plasmid	CGE_PlasmidFinder
E. coli	CP001162.1	Plasmid	CGE_PlasmidFinder
E. coli	CP001362.1	Plasmid	CGE_PlasmidFinder
E. coli	CP001369.1	Plasmid	CGE_PlasmidFinder
E. coli	CP001384.1	Plasmid	CGE_PlasmidFinder
E. coli	CP001386.1	Plasmid	CGE_PlasmidFinder
E. coli	CP001587.1	Plasmid	CGE_PlasmidFinder
E. coli	CP001591.1	Plasmid	CGE_PlasmidFinder
E. coli	CP001595.1	Plasmid	CGE_PlasmidFinder
E. coli	CP001847.1	Plasmid	CGE_PlasmidFinder
E. coli	CP001919.1	Plasmid	CGE_PlasmidFinder
E. coli	CP001926.1	Plasmid	CGE_PlasmidFinder
E. coli	CP001927.1	Plasmid	CGE_PlasmidFinder
E. coli	CP002089.1	Plasmid	CGE_PlasmidFinder
E. coli	CP002090.1	Plasmid	CGE_PlasmidFinder
E. coli	CP002168.1	Plasmid	CGE_PlasmidFinder
E. coli	CP002180.1	Plasmid	CGE_PlasmidFinder
E. coli	CP002187.1	Plasmid	CGE_PlasmidFinder
E. coli	CP002247.1	Plasmid	CGE_PlasmidFinder
E. coli	CP002474.1	Plasmid	CGE_PlasmidFinder
E. coli	CP002488.1	Plasmid	CGE_PlasmidFinder
E. coli	CP002615.1	Plasmid	CGE_PlasmidFinder
E. coli	CP002730.1	Plasmid	CGE_PlasmidFinder
E. coli	CP002732.1	Plasmid	CGE_PlasmidFinder
E. coli	CP002887.1	Plasmid	CGE_PlasmidFinder
E. coli	CP002957.1	Plasmid	CGE_PlasmidFinder
E. coli	CP002969.1	Plasmid	CGE_PlasmidFinder
E. coli	CP003027.1	Plasmid	CGE_PlasmidFinder
E. coli	CP003028.1	Plasmid	CGE_PlasmidFinder

E. coli	CP003035.1	Plasmid	CGE_PlasmidFinder
E. coli	CP003110.1	Plasmid	CGE_PlasmidFinder
E. coli	CP003111.1	Plasmid	CGE_PlasmidFinder
E. coli	CP003112.1	Plasmid	CGE_PlasmidFinder
E. coli	CP003113.1	Plasmid	CGE_PlasmidFinder
E. coli	CP003223.1	Plasmid	CGE_PlasmidFinder
E. coli	CP003224.1	Plasmid	CGE_PlasmidFinder
E. coli	CP003225.1	Plasmid	CGE_PlasmidFinder
E. coli	CP003387.1	Plasmid	CGE_PlasmidFinder
E. coli	CP003417.1	Plasmid	CGE_PlasmidFinder
E. coli	CP003684.1	Plasmid	CGE_PlasmidFinder
E. coli	CU638872.1	Plasmid	CGE_PlasmidFinder
E. coli	CU928144.1	Plasmid	CGE_PlasmidFinder
E. coli	CU928146.1	Plasmid	CGE_PlasmidFinder
E. coli	CU928147.1	Plasmid	CGE_PlasmidFinder
E. coli	CU928148.1	Plasmid	CGE_PlasmidFinder
E. coli	CU928149.1	Plasmid	CGE_PlasmidFinder
E. coli	CU928159.2	Plasmid	CGE_PlasmidFinder
E. coli	DQ017661.1	Plasmid	CGE_PlasmidFinder
E. coli	DQ115387.2	Plasmid	CGE_PlasmidFinder
E. coli	DQ115388.2	Plasmid	CGE_PlasmidFinder
E. coli	DQ298019.1	Plasmid	CGE_PlasmidFinder
E. coli	DQ311641.1	Plasmid	CGE_PlasmidFinder
E. coli	DQ364638.1	Plasmid	CGE_PlasmidFinder
E. coli	DQ381420.1	Plasmid	CGE_PlasmidFinder
E. coli	DQ388534.1	Plasmid	CGE_PlasmidFinder
E. coli	DQ390454.1	Plasmid	CGE_PlasmidFinder
E. coli	DQ390455.1	Plasmid	CGE_PlasmidFinder
E. coli	DQ401103.1	Plasmid	CGE_PlasmidFinder
E. coli	DQ449578.1	Plasmid	CGE_PlasmidFinder
E. coli	DQ659147.1	Plasmid	CGE_PlasmidFinder
E. coli	DQ916145.1	Plasmid	CGE_PlasmidFinder
E. coli	DQ916413.1	Plasmid	CGE_PlasmidFinder
E. coli	DQ995352.1	Plasmid	CGE_PlasmidFinder
E. coli	DQ995355.1	Plasmid	CGE_PlasmidFinder
E. coli	EF090911.1	Plasmid	CGE_PlasmidFinder
E. coli	EF219134.3	Plasmid	CGE_PlasmidFinder
E. coli	EF536825.1	Plasmid	CGE_PlasmidFinder
E. coli	EF633507.1	Plasmid	CGE_PlasmidFinder
E. coli	EU195449.1	Plasmid	CGE_PlasmidFinder
E. coli	EU219533.1	Plasmid	CGE_PlasmidFinder
E. coli	EU219534.1	Plasmid	CGE_PlasmidFinder
E. coli	EU330199.1	Plasmid	CGE_PlasmidFinder
E. coli	EU331425.1	Plasmid	CGE_PlasmidFinder
E. coli	EU370913.1	Plasmid	CGE_PlasmidFinder
E. coli	EU383016.1	Plasmid	CGE_PlasmidFinder
E. coli	EU880929.1	Plasmid	CGE_PlasmidFinder
E. coli	EU935738.1	Plasmid	CGE_PlasmidFinder
E. coli	EU935739.1	Plasmid	CGE_PlasmidFinder
E. coli	EU938349.1	Plasmid	CGE_PlasmidFinder
E. coli	EU999782.1	Plasmid	CGE_PlasmidFinder
E. coli	FJ223605.1	Plasmid	CGE_PlasmidFinder
E. coli	FJ223606.1	Plasmid	CGE_PlasmidFinder
E. coli	FJ223607.1	Plasmid	CGE_PlasmidFinder
E. coli	FJ386569.1	Plasmid	CGE_PlasmidFinder
E. coli	FJ449539.1	Plasmid	CGE_PlasmidFinder
E. coli	FJ494913.1	Plasmid	CGE_PlasmidFinder
E. coli	FJ621586.1	Plasmid	CGE_PlasmidFinder
E. coli	FJ621587.1	Plasmid	CGE_PlasmidFinder
E. coli	FJ621588.1	Plasmid	CGE_PlasmidFinder
E. coli	FJ628167.2	Plasmid	CGE_PlasmidFinder
E. coli	FJ666132.1	Plasmid	CGE_PlasmidFinder
E. coli	FJ696405.1	Plasmid	CGE_PlasmidFinder
E. coli	FJ705806.1	Plasmid	CGE_PlasmidFinder
E. coli	FJ876826.1	Plasmid	CGE_PlasmidFinder
E. coli	FJ876827.1	Plasmid	CGE_PlasmidFinder
E. coli	FJ914220.1	Plasmid	CGE_PlasmidFinder
E. coli	FM180569.1	Plasmid	CGE_PlasmidFinder
E. coli	FN428572.1	Plasmid	CGE_PlasmidFinder
E. coli	FN432031.1	Plasmid	CGE_PlasmidFinder
E. coli	FN543094.1	Plasmid	CGE_PlasmidFinder
E. coli	FN543095.1	Plasmid	CGE_PlasmidFinder

E. coli	FN543096.1	Plasmid	CGE_PlasmidFinder
E. coli	FN543503.1	Plasmid	CGE_PlasmidFinder
E. coli	FN543504.1	Plasmid	CGE_PlasmidFinder
E. coli	FN554767.1	Plasmid	CGE_PlasmidFinder
E. coli	FN594520.1	Plasmid	CGE_PlasmidFinder
E. coli	FN649417.1	Plasmid	CGE_PlasmidFinder
E. coli	FN649418.1	Plasmid	CGE_PlasmidFinder
E. coli	FN822745.1	Plasmid	CGE_PlasmidFinder
E. coli	FN822746.1	Plasmid	CGE_PlasmidFinder
E. coli	FN868832.1	Plasmid	CGE_PlasmidFinder
E. coli	FQ482074.1	Plasmid	CGE_PlasmidFinder
E. coli	FR687019.1	Plasmid	CGE_PlasmidFinder
E. coli	FR850039.1	Plasmid	CGE_PlasmidFinder
E. coli	FR851303.1	Plasmid	CGE_PlasmidFinder
E. coli	FR851304.1	Plasmid	CGE_PlasmidFinder
E. coli	FR851305.1	Plasmid	CGE_PlasmidFinder
E. coli	GQ149342.1	Plasmid	CGE_PlasmidFinder
E. coli	GQ149343.1	Plasmid	CGE_PlasmidFinder
E. coli	GQ149344.1	Plasmid	CGE_PlasmidFinder
E. coli	GQ149345.1	Plasmid	CGE_PlasmidFinder
E. coli	GQ149346.1	Plasmid	CGE_PlasmidFinder
E. coli	GQ149347.1	Plasmid	CGE_PlasmidFinder
E. coli	GQ149348.1	Plasmid	CGE_PlasmidFinder
E. coli	GQ259888.1	Plasmid	CGE_PlasmidFinder
E. coli	GQ374156.1	Plasmid	CGE_PlasmidFinder
E. coli	GQ374157.1	Plasmid	CGE_PlasmidFinder
E. coli	GQ379901.1	Plasmid	CGE_PlasmidFinder
E. coli	GQ398086.1	Plasmid	CGE_PlasmidFinder
E. coli	GQ412195.1	Plasmid	CGE_PlasmidFinder
E. coli	GU256641.1	Plasmid	CGE_PlasmidFinder
E. coli	GU363949.1	Plasmid	CGE_PlasmidFinder
E. coli	GU371926.1	Plasmid	CGE_PlasmidFinder
E. coli	GU371928.1	Plasmid	CGE_PlasmidFinder
E. coli	GU371929.1	Plasmid	CGE_PlasmidFinder
E. coli	GU585907.1	Plasmid	CGE_PlasmidFinder
E. coli	GU595196.1	Plasmid	CGE_PlasmidFinder
E. coli	GU949535.1	Plasmid	CGE_PlasmidFinder
E. coli	HE577112.1	Plasmid	CGE_PlasmidFinder
E. coli	HE578057.1	Plasmid	CGE_PlasmidFinder
E. coli	HE578058.1	Plasmid	CGE_PlasmidFinder
E. coli	HE603110.1	Plasmid	CGE_PlasmidFinder
E. coli	HE603111.1	Plasmid	CGE_PlasmidFinder
E. coli	HE613857.1	Plasmid	CGE_PlasmidFinder
E. coli	HE616529.1	Plasmid	CGE_PlasmidFinder
E. coli	HE652087.1	Plasmid	CGE_PlasmidFinder
E. coli	HE654726.1	Plasmid	CGE_PlasmidFinder
E. coli	HE663166.1	Plasmid	CGE_PlasmidFinder
E. coli	HM070380.1	Plasmid	CGE_PlasmidFinder
E. coli	HM126016.1	Plasmid	CGE_PlasmidFinder
E. coli	HM138194.1	Plasmid	CGE_PlasmidFinder
E. coli	HM138652.1	Plasmid	CGE_PlasmidFinder
E. coli	HM138653.1	Plasmid	CGE_PlasmidFinder
E. coli	HM231165.1	Plasmid	CGE_PlasmidFinder
E. coli	HM355591.2	Plasmid	CGE_PlasmidFinder
E. coli	HM807366.1	Plasmid	CGE_PlasmidFinder
E. coli	HM807367.1	Plasmid	CGE_PlasmidFinder
E. coli	HM807368.1	Plasmid	CGE_PlasmidFinder
E. coli	HQ023861.1	Plasmid	CGE_PlasmidFinder
E. coli	HQ023862.1	Plasmid	CGE_PlasmidFinder
E. coli	HQ023863.1	Plasmid	CGE_PlasmidFinder
E. coli	HQ328804.1	Plasmid	CGE_PlasmidFinder
E. coli	HQ404303.1	Plasmid	CGE_PlasmidFinder
E. coli	HQ451074.1	Plasmid	CGE_PlasmidFinder
E. coli	HQ589350.1	Plasmid	CGE_PlasmidFinder
E. coli	HQ834472.1	Plasmid	CGE_PlasmidFinder
E. coli	HQ834473.1	Plasmid	CGE_PlasmidFinder
E. coli	J01566.1	Plasmid	CGE_PlasmidFinder
E. coli	JF267651.1	Plasmid	CGE_PlasmidFinder
E. coli	JF267652.1	Plasmid	CGE_PlasmidFinder
E. coli	JF267653.1	Plasmid	CGE_PlasmidFinder
E. coli	JF274991.1	Plasmid	CGE_PlasmidFinder
E. coli	JF274992.1	Plasmid	CGE_PlasmidFinder

E. coli	JF436966.1	Plasmid	CGE_PlasmidFinder
E. coli	JF503991.1	Plasmid	CGE_PlasmidFinder
E. coli	JF714412.2	Plasmid	CGE_PlasmidFinder
E. coli	JF776874.1	Plasmid	CGE_PlasmidFinder
E. coli	JF779678.1	Plasmid	CGE_PlasmidFinder
E. coli	JF785549.1	Plasmid	CGE_PlasmidFinder
E. coli	JF785550.1	Plasmid	CGE_PlasmidFinder
E. coli	JF813187.1	Plasmid	CGE_PlasmidFinder
E. coli	JF813188.1	Plasmid	CGE_PlasmidFinder
E. coli	JF828150.1	Plasmid	CGE_PlasmidFinder
E. coli	JF927996.1	Plasmid	CGE_PlasmidFinder
E. coli	JF937655.1	Plasmid	CGE_PlasmidFinder
E. coli	JN087528.1	Plasmid	CGE_PlasmidFinder
E. coli	JN087529.1	Plasmid	CGE_PlasmidFinder
E. coli	JN157804.1	Plasmid	CGE_PlasmidFinder
E. coli	JN183060.1	Plasmid	CGE_PlasmidFinder
E. coli	JN183061.1	Plasmid	CGE_PlasmidFinder
E. coli	JN194214.1	Plasmid	CGE_PlasmidFinder
E. coli	JN205800.1	Plasmid	CGE_PlasmidFinder
E. coli	JN232517.1	Plasmid	CGE_PlasmidFinder
E. coli	JN233704.1	Plasmid	CGE_PlasmidFinder
E. coli	JN233705.2	Plasmid	CGE_PlasmidFinder
E. coli	JN247852.1	Plasmid	CGE_PlasmidFinder
E. coli	JN247853.1	Plasmid	CGE_PlasmidFinder
E. coli	JN253636.1	Plasmid	CGE_PlasmidFinder
E. coli	JN393220.1	Plasmid	CGE_PlasmidFinder
E. coli	JN566044.1	Plasmid	CGE_PlasmidFinder
E. coli	JN626286.1	Plasmid	CGE_PlasmidFinder
E. coli	JN687470.1	Plasmid	CGE_PlasmidFinder
E. coli	JN861072.1	Plasmid	CGE_PlasmidFinder
E. coli	JN885080.1	Plasmid	CGE_PlasmidFinder
E. coli	JN885081.1	Plasmid	CGE_PlasmidFinder
E. coli	JN887338.1	Plasmid	CGE_PlasmidFinder
E. coli	JN935897.1	Plasmid	CGE_PlasmidFinder
E. coli	JN935898.1	Plasmid	CGE_PlasmidFinder
E. coli	JN935899.1	Plasmid	CGE_PlasmidFinder
E. coli	JN983043.1	Plasmid	CGE_PlasmidFinder
E. coli	JN983044.1	Plasmid	CGE_PlasmidFinder
E. coli	JN983045.1	Plasmid	CGE_PlasmidFinder
E. coli	JN983046.1	Plasmid	CGE_PlasmidFinder
E. coli	JN983047.1	Plasmid	CGE_PlasmidFinder
E. coli	JN983048.1	Plasmid	CGE_PlasmidFinder
E. coli	JN995611.1	Plasmid	CGE_PlasmidFinder
E. coli	JQ269335.1	Plasmid	CGE_PlasmidFinder
E. coli	JQ269336.1	Plasmid	CGE_PlasmidFinder
E. coli	JQ349086.2	Plasmid	CGE_PlasmidFinder
E. coli	JQ418521.1	Plasmid	CGE_PlasmidFinder
E. coli	JQ418522.1	Plasmid	CGE_PlasmidFinder
E. coli	JQ418533.1	Plasmid	CGE_PlasmidFinder
E. coli	JQ418538.1	Plasmid	CGE_PlasmidFinder
E. coli	JQ418540.1	Plasmid	CGE_PlasmidFinder
E. coli	JQ418541.1	Plasmid	CGE_PlasmidFinder
E. coli	JQ418542.1	Plasmid	CGE_PlasmidFinder
E. coli	JQ609357.1	Plasmid	CGE_PlasmidFinder
E. coli	JQ776501.1	Plasmid	CGE_PlasmidFinder
E. coli	JQ776502.1	Plasmid	CGE_PlasmidFinder
E. coli	JQ776503.1	Plasmid	CGE_PlasmidFinder
E. coli	JQ776504.1	Plasmid	CGE_PlasmidFinder
E. coli	JQ776505.1	Plasmid	CGE_PlasmidFinder
E. coli	JQ776506.1	Plasmid	CGE_PlasmidFinder
E. coli	JQ776507.1	Plasmid	CGE_PlasmidFinder
E. coli	JQ776508.1	Plasmid	CGE_PlasmidFinder
E. coli	JQ776509.1	Plasmid	CGE_PlasmidFinder
E. coli	JQ824049.1	Plasmid	CGE_PlasmidFinder
E. coli	JQ837276.1	Plasmid	CGE_PlasmidFinder
E. coli	JX065630.1	Plasmid	CGE_PlasmidFinder
E. coli	JX065631.1	Plasmid	CGE_PlasmidFinder
E. coli	JX101693.1	Plasmid	CGE_PlasmidFinder
E. coli	JX104759.1	Plasmid	CGE_PlasmidFinder
E. coli	JX104760.1	Plasmid	CGE_PlasmidFinder
E. coli	JX258654.1	Plasmid	CGE_PlasmidFinder
E. coli	JX258655.1	Plasmid	CGE_PlasmidFinder

E. coli	JX258656.1	Plasmid	CGE_PlasmidFinder
E. coli	JX461340.1	Plasmid	CGE_PlasmidFinder
E. coli	JX514065.1	Plasmid	CGE_PlasmidFinder
E. coli	JX514066.1	Plasmid	CGE_PlasmidFinder
E. coli	NC_001848.1	Plasmid	CGE_PlasmidFinder
E. coli	NC_002142.1	Plasmid	CGE_PlasmidFinder
E. coli	NC_002144.1	Plasmid	CGE_PlasmidFinder
E. coli	NC_002487.1	Plasmid	CGE_PlasmidFinder
E. coli	NC_002610.1	Plasmid	CGE_PlasmidFinder
E. coli	NC_002773.1	Plasmid	CGE_PlasmidFinder
E. coli	NC_002809.1	Plasmid	CGE_PlasmidFinder
E. coli	NC_003079.1	Plasmid	CGE_PlasmidFinder
E. coli	NC_003132.1	Plasmid	CGE_PlasmidFinder
E. coli	NC_003134.1	Plasmid	CGE_PlasmidFinder
E. coli	NC_003455.1	Plasmid	CGE_PlasmidFinder
E. coli	NC_003486.1	Plasmid	CGE_PlasmidFinder
E. coli	NC_004837.1	Plasmid	CGE_PlasmidFinder
E. coli	NC_005015.1	Plasmid	CGE_PlasmidFinder
E. coli	NC_005018.1	Plasmid	CGE_PlasmidFinder
E. coli	NC_005248.1	Plasmid	CGE_PlasmidFinder
E. coli	NC_005570.1	Plasmid	CGE_PlasmidFinder
E. coli	NC_005862.1	Plasmid	CGE_PlasmidFinder
E. coli	NC_005923.1	Plasmid	CGE_PlasmidFinder
E. coli	NC_005970.1	Plasmid	CGE_PlasmidFinder
E. coli	NC_006815.1	Plasmid	CGE_PlasmidFinder
E. coli	NC_007635.1	Plasmid	CGE_PlasmidFinder
E. coli	NC_008119.1	Plasmid	CGE_PlasmidFinder
E. coli	NC_008121.1	Plasmid	CGE_PlasmidFinder
E. coli	NC_008444.1	Plasmid	CGE_PlasmidFinder
E. coli	NC_008488.1	Plasmid	CGE_PlasmidFinder
E. coli	NC_008489.1	Plasmid	CGE_PlasmidFinder
E. coli	NC_009344.1	Plasmid	CGE_PlasmidFinder
E. coli	NC_009345.1	Plasmid	CGE_PlasmidFinder
E. coli	NC_009346.1	Plasmid	CGE_PlasmidFinder
E. coli	NC_009347.1	Plasmid	CGE_PlasmidFinder
E. coli	NC_009425.1	Plasmid	CGE_PlasmidFinder
E. coli	NC_009652.1	Plasmid	CGE_PlasmidFinder
E. coli	NC_009653.1	Plasmid	CGE_PlasmidFinder
E. coli	NC_009704.1	Plasmid	CGE_PlasmidFinder
E. coli	NC_009705.1	Plasmid	CGE_PlasmidFinder
E. coli	NC_009716.1	Plasmid	CGE_PlasmidFinder
E. coli	NC_009779.1	Plasmid	CGE_PlasmidFinder
E. coli	NC_009781.1	Plasmid	CGE_PlasmidFinder
E. coli	NC_009786.1	Plasmid	CGE_PlasmidFinder
E. coli	NC_009787.1	Plasmid	CGE_PlasmidFinder
E. coli	NC_009790.1	Plasmid	CGE_PlasmidFinder
E. coli	NC_009807.1	Plasmid	CGE_PlasmidFinder
E. coli	NC_009829.1	Plasmid	CGE_PlasmidFinder
E. coli	NC_010158.1	Plasmid	CGE_PlasmidFinder
E. coli	NC_010261.1	Plasmid	CGE_PlasmidFinder
E. coli	NC_010377.1	Plasmid	CGE_PlasmidFinder
E. coli	NC_010485.1	Plasmid	CGE_PlasmidFinder
E. coli	NC_010487.1	Plasmid	CGE_PlasmidFinder
E. coli	NC_010488.1	Plasmid	CGE_PlasmidFinder
E. coli	NC_010656.1	Plasmid	CGE_PlasmidFinder
E. coli	NC_010659.1	Plasmid	CGE_PlasmidFinder
E. coli	NC_010672.1	Plasmid	CGE_PlasmidFinder
E. coli	NC_010796.1	Plasmid	CGE_PlasmidFinder
E. coli	NC_010894.1	Plasmid	CGE_PlasmidFinder
E. coli	NC_010898.1	Plasmid	CGE_PlasmidFinder
E. coli	NC_010904.1	Plasmid	CGE_PlasmidFinder
E. coli	NC_010993.1	Plasmid	CGE_PlasmidFinder
E. coli	NC_011079.1	Plasmid	CGE_PlasmidFinder
E. coli	NC_011093.1	Plasmid	CGE_PlasmidFinder
E. coli	NC_011148.1	Plasmid	CGE_PlasmidFinder
E. coli	NC_011204.1	Plasmid	CGE_PlasmidFinder
E. coli	NC_011214.1	Plasmid	CGE_PlasmidFinder
E. coli	NC_011215.1	Plasmid	CGE_PlasmidFinder
E. coli	NC_011227.1	Plasmid	CGE_PlasmidFinder
E. coli	NC_011228.1	Plasmid	CGE_PlasmidFinder
E. coli	NC_011350.1	Plasmid	CGE_PlasmidFinder
E. coli	NC_011351.1	Plasmid	CGE_PlasmidFinder

E. coli	NC_011406.1	Plasmid	CGE_PlasmidFinder
E. coli	NC_011407.1	Plasmid	CGE_PlasmidFinder
E. coli	NC_011418.1	Plasmid	CGE_PlasmidFinder
E. coli	NC_011640.1	Plasmid	CGE_PlasmidFinder
E. coli	NC_011759.1	Plasmid	CGE_PlasmidFinder
E. coli	NC_011795.1	Plasmid	CGE_PlasmidFinder
E. coli	NC_011799.1	Plasmid	CGE_PlasmidFinder
E. coli	NC_011897.1	Plasmid	CGE_PlasmidFinder
E. coli	NC_011964.1	Plasmid	CGE_PlasmidFinder
E. coli	NC_011977.1	Plasmid	CGE_PlasmidFinder
E. coli	NC_012006.1	Plasmid	CGE_PlasmidFinder
E. coli	NC_012882.1	Plasmid	CGE_PlasmidFinder
E. coli	NC_013363.1	Plasmid	CGE_PlasmidFinder
E. coli	NC_013367.1	Plasmid	CGE_PlasmidFinder
E. coli	NC_013719.1	Plasmid	CGE_PlasmidFinder
E. coli	NC_014003.1	Plasmid	CGE_PlasmidFinder
E. coli	NC_014027.1	Plasmid	CGE_PlasmidFinder
E. coli	NC_014356.1	Plasmid	CGE_PlasmidFinder
E. coli	NC_014543.1	Plasmid	CGE_PlasmidFinder
E. coli	NC_015054.1	Plasmid	CGE_PlasmidFinder
E. coli	NC_015068.1	Plasmid	CGE_PlasmidFinder
E. coli	NC_015154.1	Plasmid	CGE_PlasmidFinder
E. coli	NC_015392.1	Plasmid	CGE_PlasmidFinder
E. coli	NC_015570.1	Plasmid	CGE_PlasmidFinder
E. coli	NC_015575.1	Plasmid	CGE_PlasmidFinder
E. coli	NC_015972.1	Plasmid	CGE_PlasmidFinder
E. coli	NC_016823.1	Plasmid	CGE_PlasmidFinder
E. coli	NC_016824.1	Plasmid	CGE_PlasmidFinder
E. coli	NC_016834.1	Plasmid	CGE_PlasmidFinder
E. coli	NC_016840.1	Plasmid	CGE_PlasmidFinder
E. coli	NC_016841.1	Plasmid	CGE_PlasmidFinder
E. coli	NC_016847.1	Plasmid	CGE_PlasmidFinder
E. coli	NC_016859.1	Plasmid	CGE_PlasmidFinder
E. coli	NC_016862.1	Plasmid	CGE_PlasmidFinder
E. coli	NC_016976.1	Plasmid	CGE_PlasmidFinder
E. coli	NC_016979.1	Plasmid	CGE_PlasmidFinder
E. coli	NC_017097.1	Plasmid	CGE_PlasmidFinder
E. coli	NC_017156.1	Plasmid	CGE_PlasmidFinder
E. coli	NC_017159.1	Plasmid	CGE_PlasmidFinder
E. coli	NC_017320.1	Plasmid	CGE_PlasmidFinder
E. coli	NC_017321.1	Plasmid	CGE_PlasmidFinder
E. coli	NC_017330.1	Plasmid	CGE_PlasmidFinder
E. coli	NC_017629.1	Plasmid	CGE_PlasmidFinder
E. coli	NC_017648.1	Plasmid	CGE_PlasmidFinder
E. coli	NC_017649.1	Plasmid	CGE_PlasmidFinder
E. coli	NC_017655.1	Plasmid	CGE_PlasmidFinder
E. coli	NC_017659.1	Plasmid	CGE_PlasmidFinder
E. coli	NC_017723.1	Plasmid	CGE_PlasmidFinder
E. coli	U67194.4	Plasmid	CGE_PlasmidFinder
E. coli	X01654.1	Plasmid	CGE_PlasmidFinder
E. coli	LT985253	Plasmid	ReplicolScope
E. coli	LT985262	Plasmid	ReplicolScope
E. coli	NC_014615	Plasmid	ReplicolScope
E. coli	LO017737	Plasmid	ReplicolScope
E. coli	LO017736	Plasmid	ReplicolScope
E. coli	LO017738	Plasmid	ReplicolScope
E. coli	LT985215	Plasmid	ReplicolScope
E. coli	LT985214	Plasmid	ReplicolScope
E. coli	LT985213	Plasmid	ReplicolScope
E. coli	LT985217	Plasmid	ReplicolScope
E. coli	LT985216	Plasmid	ReplicolScope
E. coli	LT985218	Plasmid	ReplicolScope
E. coli	LT985220	Plasmid	ReplicolScope
E. coli	LT985219	Plasmid	ReplicolScope
E. coli	LT985221	Plasmid	ReplicolScope
E. coli	LT985222	Plasmid	ReplicolScope
E. coli	LT985223	Plasmid	ReplicolScope
E. coli	LT985226	Plasmid	ReplicolScope
E. coli	LT985227	Plasmid	ReplicolScope
E. coli	LT985228	Plasmid	ReplicolScope
E. coli	LT985238	Plasmid	ReplicolScope
E. coli	LT985239	Plasmid	ReplicolScope

E. coli	LT985225	Plasmid	ReplicolScope
E. coli	LT985224	Plasmid	ReplicolScope
E. coli	LT985240	Plasmid	ReplicolScope
E. coli	LT985234	Plasmid	ReplicolScope
E. coli	LT985236	Plasmid	ReplicolScope
E. coli	LT985237	Plasmid	ReplicolScope
E. coli	LT985235	Plasmid	ReplicolScope
E. coli	LT985232	Plasmid	ReplicolScope
E. coli	LT985233	Plasmid	ReplicolScope
E. coli	LT985229	Plasmid	ReplicolScope
E. coli	LT985230	Plasmid	ReplicolScope
E. coli	LT985231	Plasmid	ReplicolScope
E. coli	LT985250	Plasmid	ReplicolScope
E. coli	LT985244	Plasmid	ReplicolScope
E. coli	LT985241	Plasmid	ReplicolScope
E. coli	LT985243	Plasmid	ReplicolScope
E. coli	LT985245	Plasmid	ReplicolScope
E. coli	LT985242	Plasmid	ReplicolScope
E. coli	LT985259	Plasmid	ReplicolScope
E. coli	LT985251	Plasmid	ReplicolScope
E. coli	LT985249	Plasmid	ReplicolScope
E. coli	LT985248	Plasmid	ReplicolScope
E. coli	LT985247	Plasmid	ReplicolScope
E. coli	LT985246	Plasmid	ReplicolScope
E. coli	LT985256	Plasmid	ReplicolScope
E. coli	LT985255	Plasmid	ReplicolScope
E. coli	LT985254	Plasmid	ReplicolScope
E. coli	LT985257	Plasmid	ReplicolScope
E. coli	LT985261	Plasmid	ReplicolScope
E. coli	LT985252	Plasmid	ReplicolScope
E. coli	LT985264	Plasmid	ReplicolScope
E. coli	LT985266	Plasmid	ReplicolScope
E. coli	LT985260	Plasmid	ReplicolScope
E. coli	LT985263	Plasmid	ReplicolScope
E. coli	LT985258	Plasmid	ReplicolScope
E. coli	LT985322	Plasmid	ReplicolScope
E. coli	LT985270	Plasmid	ReplicolScope
E. coli	LT985265	Plasmid	ReplicolScope
E. coli	LT985268	Plasmid	ReplicolScope
E. coli	LT985271	Plasmid	ReplicolScope
E. coli	LT985279	Plasmid	ReplicolScope
E. coli	LT985267	Plasmid	ReplicolScope
E. coli	LT985272	Plasmid	ReplicolScope
E. coli	LT985269	Plasmid	ReplicolScope
E. coli	LT985294	Plasmid	ReplicolScope
E. coli	LT985295	Plasmid	ReplicolScope
E. coli	LT985277	Plasmid	ReplicolScope
E. coli	LT985274	Plasmid	ReplicolScope
E. coli	LT985276	Plasmid	ReplicolScope
E. coli	LT985278	Plasmid	ReplicolScope
E. coli	LT985287	Plasmid	ReplicolScope
E. coli	LT985275	Plasmid	ReplicolScope
E. coli	LT985281	Plasmid	ReplicolScope
E. coli	LT985273	Plasmid	ReplicolScope
E. coli	LT985288	Plasmid	ReplicolScope
E. coli	LT985283	Plasmid	ReplicolScope
E. coli	LT985282	Plasmid	ReplicolScope
E. coli	LT985280	Plasmid	ReplicolScope
E. coli	LT985286	Plasmid	ReplicolScope
E. coli	LT985284	Plasmid	ReplicolScope
E. coli	LT985285	Plasmid	ReplicolScope
E. coli	LT985297	Plasmid	ReplicolScope
E. coli	LT985296	Plasmid	ReplicolScope
E. coli	LT985293	Plasmid	ReplicolScope
E. coli	LT985289	Plasmid	ReplicolScope
E. coli	LT985317	Plasmid	ReplicolScope
E. coli	LT985302	Plasmid	ReplicolScope
E. coli	LT985303	Plasmid	ReplicolScope
E. coli	LT985290	Plasmid	ReplicolScope
E. coli	LT985291	Plasmid	ReplicolScope
E. coli	LT985292	Plasmid	ReplicolScope
E. coli	LT985305	Plasmid	ReplicolScope

E. coli	LT985321	Plasmid	ReplicolScope
E. coli	LT985320	Plasmid	ReplicolScope
E. coli	LT985307	Plasmid	ReplicolScope
E. coli	LT985298	Plasmid	ReplicolScope
E. coli	LT985306	Plasmid	ReplicolScope
E. coli	LT985304	Plasmid	ReplicolScope
E. coli	LT985300	Plasmid	ReplicolScope
E. coli	LT985301	Plasmid	ReplicolScope
E. coli	LT985299	Plasmid	ReplicolScope
E. coli	LT985314	Plasmid	ReplicolScope
E. coli	LT985313	Plasmid	ReplicolScope
E. coli	LT985312	Plasmid	ReplicolScope
E. coli	LT985309	Plasmid	ReplicolScope
E. coli	LT985310	Plasmid	ReplicolScope
E. coli	LT985311	Plasmid	ReplicolScope
E. coli	LT985308	Plasmid	ReplicolScope
E. coli	LT985318	Plasmid	ReplicolScope
E. coli	LT985319	Plasmid	ReplicolScope
E. coli	LT985316	Plasmid	ReplicolScope
E. coli	LT985315	Plasmid	ReplicolScope
Klebsiella sp.	AP006725.1	Chromosome	NCBI_complete_genomes
Klebsiella sp.	AP014950.1	Chromosome	NCBI_complete_genomes
Klebsiella sp.	AP014951.1	Chromosome	NCBI_complete_genomes
Klebsiella sp.	CP000647.1	Chromosome	NCBI_complete_genomes
Klebsiella sp.	CP000964.1	Chromosome	NCBI_complete_genomes
Klebsiella sp.	CP001891.1	Chromosome	NCBI_complete_genomes
Klebsiella sp.	CP002910.1	Chromosome	NCBI_complete_genomes
Klebsiella sp.	CP003200.1	Chromosome	NCBI_complete_genomes
Klebsiella sp.	CP003218.1	Chromosome	NCBI_complete_genomes
Klebsiella sp.	CP003683.1	Chromosome	NCBI_complete_genomes
Klebsiella sp.	CP003785.1	Chromosome	NCBI_complete_genomes
Klebsiella sp.	CP003999.1	Chromosome	NCBI_complete_genomes
Klebsiella sp.	CP004887.1	Chromosome	NCBI_complete_genomes
Klebsiella sp.	CP006648.1	Chromosome	NCBI_complete_genomes
Klebsiella sp.	CP006656.1	Chromosome	NCBI_complete_genomes
Klebsiella sp.	CP006659.2	Chromosome	NCBI_complete_genomes
Klebsiella sp.	CP006722.1	Chromosome	NCBI_complete_genomes
Klebsiella sp.	CP006738.1	Chromosome	NCBI_complete_genomes
Klebsiella sp.	CP006798.1	Chromosome	NCBI_complete_genomes
Klebsiella sp.	CP006918.1	Chromosome	NCBI_complete_genomes
Klebsiella sp.	CP006923.1	Chromosome	NCBI_complete_genomes
Klebsiella sp.	CP007727.1	Chromosome	NCBI_complete_genomes
Klebsiella sp.	CP007731.1	Chromosome	NCBI_complete_genomes
Klebsiella sp.	CP008700.1	Chromosome	NCBI_complete_genomes
Klebsiella sp.	CP008788.1	Chromosome	NCBI_complete_genomes
Klebsiella sp.	CP008797.1	Chromosome	NCBI_complete_genomes
Klebsiella sp.	CP008827.1	Chromosome	NCBI_complete_genomes
Klebsiella sp.	CP008831.1	Chromosome	NCBI_complete_genomes
Klebsiella sp.	CP008841.1	Chromosome	NCBI_complete_genomes
Klebsiella sp.	CP008929.1	Chromosome	NCBI_complete_genomes
Klebsiella sp.	CP009114.1	Chromosome	NCBI_complete_genomes
Klebsiella sp.	CP009208.1	Chromosome	NCBI_complete_genomes
Klebsiella sp.	CP009274.2	Chromosome	NCBI_complete_genomes
Klebsiella sp.	CP009461.1	Chromosome	NCBI_complete_genomes
Klebsiella sp.	CP009771.1	Chromosome	NCBI_complete_genomes
Klebsiella sp.	CP009775.1	Chromosome	NCBI_complete_genomes
Klebsiella sp.	CP009863.1	Chromosome	NCBI_complete_genomes
Klebsiella sp.	CP009872.1	Chromosome	NCBI_complete_genomes
Klebsiella sp.	CP009876.1	Chromosome	NCBI_complete_genomes
Klebsiella sp.	CP010361.1	Chromosome	NCBI_complete_genomes
Klebsiella sp.	CP010392.1	Chromosome	NCBI_complete_genomes
Klebsiella sp.	CP010523.2	Chromosome	NCBI_complete_genomes
Klebsiella sp.	CP011313.1	Chromosome	NCBI_complete_genomes
Klebsiella sp.	CP011421.1	Chromosome	NCBI_complete_genomes
Klebsiella sp.	CP011539.1	Chromosome	NCBI_complete_genomes
Klebsiella sp.	CP011574.1	Chromosome	NCBI_complete_genomes
Klebsiella sp.	CP011578.1	Chromosome	NCBI_complete_genomes
Klebsiella sp.	CP011597.1	Chromosome	NCBI_complete_genomes
Klebsiella sp.	CP011618.1	Chromosome	NCBI_complete_genomes
Klebsiella sp.	CP011624.1	Chromosome	NCBI_complete_genomes
Klebsiella sp.	CP011636.1	Chromosome	NCBI_complete_genomes
Klebsiella sp.	CP011647.1	Chromosome	NCBI_complete_genomes

Klebsiella sp.	CP011976.1	Chromosome	NCBI_complete_genomes
Klebsiella sp.	CP011980.1	Chromosome	NCBI_complete_genomes
Klebsiella sp.	CP011985.1	Chromosome	NCBI_complete_genomes
Klebsiella sp.	CP011989.1	Chromosome	NCBI_complete_genomes
Klebsiella sp.	CP012043.1	Chromosome	NCBI_complete_genomes
Klebsiella sp.	CP012252.1	Chromosome	NCBI_complete_genomes
Klebsiella sp.	CP012300.1	Chromosome	NCBI_complete_genomes
Klebsiella sp.	CP012426.1	Chromosome	NCBI_complete_genomes
Klebsiella sp.	CP012560.1	Chromosome	NCBI_complete_genomes
Klebsiella sp.	CP012561.1	Chromosome	NCBI_complete_genomes
Klebsiella sp.	CP012568.1	Chromosome	NCBI_complete_genomes
Klebsiella sp.	CP012743.1	Chromosome	NCBI_complete_genomes
Klebsiella sp.	CP012744.1	Chromosome	NCBI_complete_genomes
Klebsiella sp.	CP012745.1	Chromosome	NCBI_complete_genomes
Klebsiella sp.	CP012753.1	Chromosome	NCBI_complete_genomes
Klebsiella sp.	CP012883.1	Chromosome	NCBI_complete_genomes
Klebsiella sp.	CP012987.1	Chromosome	NCBI_complete_genomes
Klebsiella sp.	CP012992.1	Chromosome	NCBI_complete_genomes
Klebsiella sp.	CP013322.1	Chromosome	NCBI_complete_genomes
Klebsiella sp.	CP013711.1	Chromosome	NCBI_complete_genomes
Klebsiella sp.	CP014004.1	Chromosome	NCBI_complete_genomes
Klebsiella sp.	CP014008.1	Chromosome	NCBI_complete_genomes
Klebsiella sp.	CP014010.1	Chromosome	NCBI_complete_genomes
Klebsiella sp.	CP014029.2	Chromosome	NCBI_complete_genomes
Klebsiella sp.	CP014071.1	Chromosome	NCBI_complete_genomes
Klebsiella sp.	CP014123.1	Chromosome	NCBI_complete_genomes
Klebsiella sp.	CP014154.1	Chromosome	NCBI_complete_genomes
Klebsiella sp.	CP014155.1	Chromosome	NCBI_complete_genomes
Klebsiella sp.	CP014156.1	Chromosome	NCBI_complete_genomes
Klebsiella sp.	CP014294.1	Chromosome	NCBI_complete_genomes
Klebsiella sp.	CP014647.1	Chromosome	NCBI_complete_genomes
Klebsiella sp.	CP014696.2	Chromosome	NCBI_complete_genomes
Klebsiella sp.	CP014748.2	Chromosome	NCBI_complete_genomes
Klebsiella sp.	CP014755.1	Chromosome	NCBI_complete_genomes
Klebsiella sp.	CP014762.1	Chromosome	NCBI_complete_genomes
Klebsiella sp.	CP015025.1	Chromosome	NCBI_complete_genomes
Klebsiella sp.	CP015120.1	Chromosome	NCBI_complete_genomes
Klebsiella sp.	CP015130.1	Chromosome	NCBI_complete_genomes
Klebsiella sp.	CP015134.1	Chromosome	NCBI_complete_genomes
Klebsiella sp.	CP015382.1	Chromosome	NCBI_complete_genomes
Klebsiella sp.	CP015385.1	Chromosome	NCBI_complete_genomes
Klebsiella sp.	CP015392.1	Chromosome	NCBI_complete_genomes
Klebsiella sp.	CP015500.1	Chromosome	NCBI_complete_genomes
Klebsiella sp.	CP015753.1	Chromosome	NCBI_complete_genomes
Klebsiella sp.	CP015822.1	Chromosome	NCBI_complete_genomes
Klebsiella sp.	CP015990.1	Chromosome	NCBI_complete_genomes
Klebsiella sp.	CP016159.1	Chromosome	NCBI_complete_genomes
Klebsiella sp.	CP016811.1	Chromosome	NCBI_complete_genomes
Klebsiella sp.	CP016813.1	Chromosome	NCBI_complete_genomes
Klebsiella sp.	CP016814.1	Chromosome	NCBI_complete_genomes
Klebsiella sp.	CP016923.1	Chromosome	NCBI_complete_genomes
Klebsiella sp.	CP016926.1	Chromosome	NCBI_complete_genomes
Klebsiella sp.	CP017284.1	Chromosome	NCBI_complete_genomes
Klebsiella sp.	CP017289.1	Chromosome	NCBI_complete_genomes
Klebsiella sp.	CP017385.1	Chromosome	NCBI_complete_genomes
Klebsiella sp.	CP017450.1	Chromosome	NCBI_complete_genomes
Klebsiella sp.	CP017849.1	Chromosome	NCBI_complete_genomes
Klebsiella sp.	CP017928.1	Chromosome	NCBI_complete_genomes
Klebsiella sp.	CP017934.1	Chromosome	NCBI_complete_genomes
Klebsiella sp.	CP017985.1	Chromosome	NCBI_complete_genomes
Klebsiella sp.	CP017994.1	Chromosome	NCBI_complete_genomes
Klebsiella sp.	CP018056.1	Chromosome	NCBI_complete_genomes
Klebsiella sp.	CP018140.1	Chromosome	NCBI_complete_genomes
Klebsiella sp.	CP018306.1	Chromosome	NCBI_complete_genomes
Klebsiella sp.	CP018337.1	Chromosome	NCBI_complete_genomes
Klebsiella sp.	CP018352.1	Chromosome	NCBI_complete_genomes
Klebsiella sp.	CP018356.1	Chromosome	NCBI_complete_genomes
Klebsiella sp.	CP018362.1	Chromosome	NCBI_complete_genomes
Klebsiella sp.	CP018364.1	Chromosome	NCBI_complete_genomes
Klebsiella sp.	CP018427.1	Chromosome	NCBI_complete_genomes
Klebsiella sp.	CP018428.1	Chromosome	NCBI_complete_genomes
Klebsiella sp.	CP018437.1	Chromosome	NCBI_complete_genomes

Klebsiella sp.	CP018438.1	Chromosome	NCBI_complete_genomes
Klebsiella sp.	CP018447.1	Chromosome	NCBI_complete_genomes
Klebsiella sp.	CP018450.1	Chromosome	NCBI_complete_genomes
Klebsiella sp.	CP018454.1	Chromosome	NCBI_complete_genomes
Klebsiella sp.	CP018458.1	Chromosome	NCBI_complete_genomes
Klebsiella sp.	CP018671.1	Chromosome	NCBI_complete_genomes
Klebsiella sp.	CP018676.1	Chromosome	NCBI_complete_genomes
Klebsiella sp.	CP018686.1	Chromosome	NCBI_complete_genomes
Klebsiella sp.	CP018692.1	Chromosome	NCBI_complete_genomes
Klebsiella sp.	CP018695.1	Chromosome	NCBI_complete_genomes
Klebsiella sp.	CP018701.1	Chromosome	NCBI_complete_genomes
Klebsiella sp.	CP018707.1	Chromosome	NCBI_complete_genomes
Klebsiella sp.	CP018713.1	Chromosome	NCBI_complete_genomes
Klebsiella sp.	CP018719.1	Chromosome	NCBI_complete_genomes
Klebsiella sp.	CP018735.1	Chromosome	NCBI_complete_genomes
Klebsiella sp.	CP018816.1	Chromosome	NCBI_complete_genomes
Klebsiella sp.	CP018883.1	Chromosome	NCBI_complete_genomes
Klebsiella sp.	CP018885.1	Chromosome	NCBI_complete_genomes
Klebsiella sp.	CP019047.1	Chromosome	NCBI_complete_genomes
Klebsiella sp.	CP019077.1	Chromosome	NCBI_complete_genomes
Klebsiella sp.	CP019079.1	Chromosome	NCBI_complete_genomes
Klebsiella sp.	CP019160.1	Chromosome	NCBI_complete_genomes
Klebsiella sp.	CP019219.1	Chromosome	NCBI_complete_genomes
Klebsiella sp.	CP019772.1	Chromosome	NCBI_complete_genomes
Klebsiella sp.	CP020061.1	Chromosome	NCBI_complete_genomes
Klebsiella sp.	CP020067.1	Chromosome	NCBI_complete_genomes
Klebsiella sp.	CP020071.1	Chromosome	NCBI_complete_genomes
Klebsiella sp.	CP020108.1	Chromosome	NCBI_complete_genomes
Klebsiella sp.	CP020358.1	Chromosome	NCBI_complete_genomes
Klebsiella sp.	CP020657.1	Chromosome	NCBI_complete_genomes
Klebsiella sp.	CP020837.1	Chromosome	NCBI_complete_genomes
Klebsiella sp.	CP020841.1	Chromosome	NCBI_complete_genomes
Klebsiella sp.	CP020847.1	Chromosome	NCBI_complete_genomes
Klebsiella sp.	CP020853.1	Chromosome	NCBI_complete_genomes
Klebsiella sp.	CP020901.1	Chromosome	NCBI_complete_genomes
Klebsiella sp.	CP021165.1	Chromosome	NCBI_complete_genomes
Klebsiella sp.	CP021539.1	Chromosome	NCBI_complete_genomes
Klebsiella sp.	CP021549.1	Chromosome	NCBI_complete_genomes
Klebsiella sp.	CP021685.1	Chromosome	NCBI_complete_genomes
Klebsiella sp.	CP021696.1	Chromosome	NCBI_complete_genomes
Klebsiella sp.	CP021708.1	Chromosome	NCBI_complete_genomes
Klebsiella sp.	CP021718.1	Chromosome	NCBI_complete_genomes
Klebsiella sp.	CP021740.1	Chromosome	NCBI_complete_genomes
Klebsiella sp.	CP021751.1	Chromosome	NCBI_complete_genomes
Klebsiella sp.	CP021757.1	Chromosome	NCBI_complete_genomes
Klebsiella sp.	CP021833.1	Chromosome	NCBI_complete_genomes
Klebsiella sp.	CP021859.1	Chromosome	NCBI_complete_genomes
Klebsiella sp.	CP021939.1	Chromosome	NCBI_complete_genomes
Klebsiella sp.	CP021944.1	Chromosome	NCBI_complete_genomes
Klebsiella sp.	CP021950.1	Chromosome	NCBI_complete_genomes
Klebsiella sp.	CP021955.1	Chromosome	NCBI_complete_genomes
Klebsiella sp.	CP021960.1	Chromosome	NCBI_complete_genomes
Klebsiella sp.	CP022023.1	Chromosome	NCBI_complete_genomes
Klebsiella sp.	CP022127.1	Chromosome	NCBI_complete_genomes
Klebsiella sp.	CP022143.1	Chromosome	NCBI_complete_genomes
Klebsiella sp.	CP022348.1	Chromosome	NCBI_complete_genomes
Klebsiella sp.	CP022573.1	Chromosome	NCBI_complete_genomes
Klebsiella sp.	CP022611.1	Chromosome	NCBI_complete_genomes
Klebsiella sp.	CP022691.1	Chromosome	NCBI_complete_genomes
Klebsiella sp.	CP022823.1	Chromosome	NCBI_complete_genomes
Klebsiella sp.	CP022882.1	Chromosome	NCBI_complete_genomes
Klebsiella sp.	CP022997.1	Chromosome	NCBI_complete_genomes
Klebsiella sp.	CP023134.2	Chromosome	NCBI_complete_genomes
Klebsiella sp.	CP023185.1	Chromosome	NCBI_complete_genomes
Klebsiella sp.	CP023249.1	Chromosome	NCBI_complete_genomes
Klebsiella sp.	CP023441.1	Chromosome	NCBI_complete_genomes
Klebsiella sp.	CP023478.1	Chromosome	NCBI_complete_genomes
Klebsiella sp.	CP023487.1	Chromosome	NCBI_complete_genomes
Klebsiella sp.	CP023502.1	Chromosome	NCBI_complete_genomes
Klebsiella sp.	CP023553.1	Chromosome	NCBI_complete_genomes
Klebsiella sp.	CP023722.1	Chromosome	NCBI_complete_genomes
Klebsiella sp.	CP023907.1	Chromosome	NCBI_complete_genomes

Klebsiella sp.	CP023913.1	Chromosome	NCBI_complete_genomes
Klebsiella sp.	CP023919.1	Chromosome	NCBI_complete_genomes
Klebsiella sp.	CP023925.1	Chromosome	NCBI_complete_genomes
Klebsiella sp.	CP023933.1	Chromosome	NCBI_complete_genomes
Klebsiella sp.	CP023941.1	Chromosome	NCBI_complete_genomes
Klebsiella sp.	CP023946.1	Chromosome	NCBI_complete_genomes
Klebsiella sp.	CP023949.1	Chromosome	NCBI_complete_genomes
Klebsiella sp.	CP023963.1	Chromosome	NCBI_complete_genomes
Klebsiella sp.	CP024038.1	Chromosome	NCBI_complete_genomes
Klebsiella sp.	CP024191.1	Chromosome	NCBI_complete_genomes
Klebsiella sp.	CP024429.1	Chromosome	NCBI_complete_genomes
Klebsiella sp.	CP024458.1	Chromosome	NCBI_complete_genomes
Klebsiella sp.	CP024482.1	Chromosome	NCBI_complete_genomes
Klebsiella sp.	CP024489.1	Chromosome	NCBI_complete_genomes
Klebsiella sp.	CP024496.1	Chromosome	NCBI_complete_genomes
Klebsiella sp.	CP024499.1	Chromosome	NCBI_complete_genomes
Klebsiella sp.	CP024504.1	Chromosome	NCBI_complete_genomes
Klebsiella sp.	CP024515.1	Chromosome	NCBI_complete_genomes
Klebsiella sp.	CP024521.1	Chromosome	NCBI_complete_genomes
Klebsiella sp.	CP024528.1	Chromosome	NCBI_complete_genomes
Klebsiella sp.	CP024535.1	Chromosome	NCBI_complete_genomes
Klebsiella sp.	CP024542.1	Chromosome	NCBI_complete_genomes
Klebsiella sp.	CP024545.1	Chromosome	NCBI_complete_genomes
Klebsiella sp.	CP024548.1	Chromosome	NCBI_complete_genomes
Klebsiella sp.	CP024549.1	Chromosome	NCBI_complete_genomes
Klebsiella sp.	CP024556.1	Chromosome	NCBI_complete_genomes
Klebsiella sp.	CP024563.1	Chromosome	NCBI_complete_genomes
Klebsiella sp.	CP024570.1	Chromosome	NCBI_complete_genomes
Klebsiella sp.	CP024834.1	Chromosome	NCBI_complete_genomes
Klebsiella sp.	CP024838.1	Chromosome	NCBI_complete_genomes
Klebsiella sp.	CP024874.1	Chromosome	NCBI_complete_genomes
Klebsiella sp.	CP024880.1	Chromosome	NCBI_complete_genomes
Klebsiella sp.	CP024883.1	Chromosome	NCBI_complete_genomes
Klebsiella sp.	CP024885.1	Chromosome	NCBI_complete_genomes
Klebsiella sp.	CP024916.1	Chromosome	NCBI_complete_genomes
Klebsiella sp.	CP025005.1	Chromosome	NCBI_complete_genomes
Klebsiella sp.	CP025008.1	Chromosome	NCBI_complete_genomes
Klebsiella sp.	CP025037.1	Chromosome	NCBI_complete_genomes
Klebsiella sp.	CP025080.1	Chromosome	NCBI_complete_genomes
Klebsiella sp.	CP025087.1	Chromosome	NCBI_complete_genomes
Klebsiella sp.	CP025088.1	Chromosome	NCBI_complete_genomes
Klebsiella sp.	CP025089.1	Chromosome	NCBI_complete_genomes
Klebsiella sp.	CP025090.1	Chromosome	NCBI_complete_genomes
Klebsiella sp.	CP025091.1	Chromosome	NCBI_complete_genomes
Klebsiella sp.	CP025092.1	Chromosome	NCBI_complete_genomes
Klebsiella sp.	CP025093.1	Chromosome	NCBI_complete_genomes
Klebsiella sp.	CP025140.1	Chromosome	NCBI_complete_genomes
Klebsiella sp.	CP025143.1	Chromosome	NCBI_complete_genomes
Klebsiella sp.	CP025146.1	Chromosome	NCBI_complete_genomes
Klebsiella sp.	CP025211.1	Chromosome	NCBI_complete_genomes
Klebsiella sp.	CP025456.1	Chromosome	NCBI_complete_genomes
Klebsiella sp.	CP025461.1	Chromosome	NCBI_complete_genomes
Klebsiella sp.	CP025466.1	Chromosome	NCBI_complete_genomes
Klebsiella sp.	CP025515.1	Chromosome	NCBI_complete_genomes
Klebsiella sp.	CP025541.1	Chromosome	NCBI_complete_genomes
Klebsiella sp.	CP025629.1	Chromosome	NCBI_complete_genomes
Klebsiella sp.	CP025630.1	Chromosome	NCBI_complete_genomes
Klebsiella sp.	CP025631.1	Chromosome	NCBI_complete_genomes
Klebsiella sp.	CP025633.1	Chromosome	NCBI_complete_genomes
Klebsiella sp.	CP025636.1	Chromosome	NCBI_complete_genomes
Klebsiella sp.	CP025639.1	Chromosome	NCBI_complete_genomes
Klebsiella sp.	CP025641.1	Chromosome	NCBI_complete_genomes
Klebsiella sp.	CP025816.1	Chromosome	NCBI_complete_genomes
Klebsiella sp.	CP025951.1	Chromosome	NCBI_complete_genomes
Klebsiella sp.	CP025963.2	Chromosome	NCBI_complete_genomes
Klebsiella sp.	CP026177.1	Chromosome	NCBI_complete_genomes
Klebsiella sp.	CP026178.1	Chromosome	NCBI_complete_genomes
Klebsiella sp.	CP026269.1	Chromosome	NCBI_complete_genomes
Klebsiella sp.	CP026275.1	Chromosome	NCBI_complete_genomes
Klebsiella sp.	CP026285.1	Chromosome	NCBI_complete_genomes
Klebsiella sp.	CP026392.1	Chromosome	NCBI_complete_genomes
Klebsiella sp.	CP026495.1	Chromosome	NCBI_complete_genomes

Klebsiella sp.	CP026585.2	Chromosome	NCBI_complete_genomes
Klebsiella sp.	CP026586.1	Chromosome	NCBI_complete_genomes
Klebsiella sp.	CP026715.1	Chromosome	NCBI_complete_genomes
Klebsiella sp.	CP026722.1	Chromosome	NCBI_complete_genomes
Klebsiella sp.	CP026751.1	Chromosome	NCBI_complete_genomes
Klebsiella sp.	CP026756.1	Chromosome	NCBI_complete_genomes
Klebsiella sp.	CP027064.2	Chromosome	NCBI_complete_genomes
Klebsiella sp.	CP027068.2	Chromosome	NCBI_complete_genomes
Klebsiella sp.	CP027146.1	Chromosome	NCBI_complete_genomes
Klebsiella sp.	CP027160.1	Chromosome	NCBI_complete_genomes
Klebsiella sp.	CP027189.1	Chromosome	NCBI_complete_genomes
Klebsiella sp.	CP027426.1	Chromosome	NCBI_complete_genomes
Klebsiella sp.	CP027602.1	Chromosome	NCBI_complete_genomes
Klebsiella sp.	CP027612.1	Chromosome	NCBI_complete_genomes
Klebsiella sp.	CP028176.1	Chromosome	NCBI_complete_genomes
Klebsiella sp.	CP028180.1	Chromosome	NCBI_complete_genomes
Klebsiella sp.	CP028391.1	Chromosome	NCBI_complete_genomes
Klebsiella sp.	CP028542.2	Chromosome	NCBI_complete_genomes
Klebsiella sp.	CP028548.1	Chromosome	NCBI_complete_genomes
Klebsiella sp.	CP028555.1	Chromosome	NCBI_complete_genomes
Klebsiella sp.	CP028583.1	Chromosome	NCBI_complete_genomes
Klebsiella sp.	CP028716.1	Chromosome	NCBI_complete_genomes
Klebsiella sp.	CP028783.1	Chromosome	NCBI_complete_genomes
Klebsiella sp.	CP028787.1	Chromosome	NCBI_complete_genomes
Klebsiella sp.	CP028793.1	Chromosome	NCBI_complete_genomes
Klebsiella sp.	CP028797.1	Chromosome	NCBI_complete_genomes
Klebsiella sp.	CP028806.1	Chromosome	NCBI_complete_genomes
Klebsiella sp.	CP028816.1	Chromosome	NCBI_complete_genomes
Klebsiella sp.	CP028915.1	Chromosome	NCBI_complete_genomes
Klebsiella sp.	CP028932.1	Chromosome	NCBI_complete_genomes
Klebsiella sp.	CP028951.1	Chromosome	NCBI_complete_genomes
Klebsiella sp.	CP028953.1	Chromosome	NCBI_complete_genomes
Klebsiella sp.	CP028990.1	Chromosome	NCBI_complete_genomes
Klebsiella sp.	CP028994.1	Chromosome	NCBI_complete_genomes
Klebsiella sp.	CP029099.1	Chromosome	NCBI_complete_genomes
Klebsiella sp.	CP029128.1	Chromosome	NCBI_complete_genomes
Klebsiella sp.	CP029137.1	Chromosome	NCBI_complete_genomes
Klebsiella sp.	CP029141.1	Chromosome	NCBI_complete_genomes
Klebsiella sp.	CP029216.1	Chromosome	NCBI_complete_genomes
Klebsiella sp.	CP029220.1	Chromosome	NCBI_complete_genomes
Klebsiella sp.	CP029226.1	Chromosome	NCBI_complete_genomes
Klebsiella sp.	CP029384.1	Chromosome	NCBI_complete_genomes
Klebsiella sp.	CP029388.1	Chromosome	NCBI_complete_genomes
Klebsiella sp.	CP029432.1	Chromosome	NCBI_complete_genomes
Klebsiella sp.	CP029437.1	Chromosome	NCBI_complete_genomes
Klebsiella sp.	CP029443.1	Chromosome	NCBI_complete_genomes
Klebsiella sp.	CP029582.1	Chromosome	NCBI_complete_genomes
Klebsiella sp.	CP029587.1	Chromosome	NCBI_complete_genomes
Klebsiella sp.	CP029590.1	Chromosome	NCBI_complete_genomes
Klebsiella sp.	CP029597.1	Chromosome	NCBI_complete_genomes
Klebsiella sp.	CP029722.1	Chromosome	NCBI_complete_genomes
Klebsiella sp.	CP029738.1	Chromosome	NCBI_complete_genomes
Klebsiella sp.	CP029770.1	Chromosome	NCBI_complete_genomes
Klebsiella sp.	FO203501.1	Chromosome	NCBI_complete_genomes
Klebsiella sp.	FO834906.1	Chromosome	NCBI_complete_genomes
Klebsiella sp.	LN824133.1	Chromosome	NCBI_complete_genomes
Klebsiella sp.	LS483483.1	Chromosome	NCBI_complete_genomes
Klebsiella sp.	LT216436.1	Chromosome	NCBI_complete_genomes
Klebsiella sp.	AP006726.1	Plasmid	NCBI_complete_genomes
Klebsiella sp.	AP014952.1	Plasmid	NCBI_complete_genomes
Klebsiella sp.	AP014953.1	Plasmid	NCBI_complete_genomes
Klebsiella sp.	AP014954.1	Plasmid	NCBI_complete_genomes
Klebsiella sp.	AP014955.1	Plasmid	NCBI_complete_genomes
Klebsiella sp.	CP000648.1	Plasmid	NCBI_complete_genomes
Klebsiella sp.	CP000649.1	Plasmid	NCBI_complete_genomes
Klebsiella sp.	CP000650.1	Plasmid	NCBI_complete_genomes
Klebsiella sp.	CP000651.1	Plasmid	NCBI_complete_genomes
Klebsiella sp.	CP000652.1	Plasmid	NCBI_complete_genomes
Klebsiella sp.	CP000965.1	Plasmid	NCBI_complete_genomes
Klebsiella sp.	CP000966.1	Plasmid	NCBI_complete_genomes
Klebsiella sp.	CP002911.1	Plasmid	NCBI_complete_genomes
Klebsiella sp.	CP003223.1	Plasmid	NCBI_complete_genomes

Klebsiella sp.	CP003224.1	Plasmid	NCBI_complete_genomes
Klebsiella sp.	CP003225.1	Plasmid	NCBI_complete_genomes
Klebsiella sp.	CP003226.1	Plasmid	NCBI_complete_genomes
Klebsiella sp.	CP003227.1	Plasmid	NCBI_complete_genomes
Klebsiella sp.	CP003228.1	Plasmid	NCBI_complete_genomes
Klebsiella sp.	CP003684.1	Plasmid	NCBI_complete_genomes
Klebsiella sp.	CP003994.1	Plasmid	NCBI_complete_genomes
Klebsiella sp.	CP003995.1	Plasmid	NCBI_complete_genomes
Klebsiella sp.	CP003996.1	Plasmid	NCBI_complete_genomes
Klebsiella sp.	CP003997.1	Plasmid	NCBI_complete_genomes
Klebsiella sp.	CP003998.1	Plasmid	NCBI_complete_genomes
Klebsiella sp.	CP004000.1	Plasmid	NCBI_complete_genomes
Klebsiella sp.	CP006657.1	Plasmid	NCBI_complete_genomes
Klebsiella sp.	CP006658.1	Plasmid	NCBI_complete_genomes
Klebsiella sp.	CP006660.1	Plasmid	NCBI_complete_genomes
Klebsiella sp.	CP006661.1	Plasmid	NCBI_complete_genomes
Klebsiella sp.	CP006662.2	Plasmid	NCBI_complete_genomes
Klebsiella sp.	CP006663.1	Plasmid	NCBI_complete_genomes
Klebsiella sp.	CP006799.1	Plasmid	NCBI_complete_genomes
Klebsiella sp.	CP006800.1	Plasmid	NCBI_complete_genomes
Klebsiella sp.	CP006801.1	Plasmid	NCBI_complete_genomes
Klebsiella sp.	CP006802.1	Plasmid	NCBI_complete_genomes
Klebsiella sp.	CP006919.1	Plasmid	NCBI_complete_genomes
Klebsiella sp.	CP006921.1	Plasmid	NCBI_complete_genomes
Klebsiella sp.	CP006922.1	Plasmid	NCBI_complete_genomes
Klebsiella sp.	CP006924.1	Plasmid	NCBI_complete_genomes
Klebsiella sp.	CP006925.1	Plasmid	NCBI_complete_genomes
Klebsiella sp.	CP006926.1	Plasmid	NCBI_complete_genomes
Klebsiella sp.	CP006927.1	Plasmid	NCBI_complete_genomes
Klebsiella sp.	CP006928.1	Plasmid	NCBI_complete_genomes
Klebsiella sp.	CP007728.1	Plasmid	NCBI_complete_genomes
Klebsiella sp.	CP007729.1	Plasmid	NCBI_complete_genomes
Klebsiella sp.	CP007730.1	Plasmid	NCBI_complete_genomes
Klebsiella sp.	CP007732.1	Plasmid	NCBI_complete_genomes
Klebsiella sp.	CP007733.1	Plasmid	NCBI_complete_genomes
Klebsiella sp.	CP007734.1	Plasmid	NCBI_complete_genomes
Klebsiella sp.	CP007735.1	Plasmid	NCBI_complete_genomes
Klebsiella sp.	CP007736.1	Plasmid	NCBI_complete_genomes
Klebsiella sp.	CP008701.1	Plasmid	NCBI_complete_genomes
Klebsiella sp.	CP008789.1	Plasmid	NCBI_complete_genomes
Klebsiella sp.	CP008790.1	Plasmid	NCBI_complete_genomes
Klebsiella sp.	CP008791.1	Plasmid	NCBI_complete_genomes
Klebsiella sp.	CP008798.1	Plasmid	NCBI_complete_genomes
Klebsiella sp.	CP008799.1	Plasmid	NCBI_complete_genomes
Klebsiella sp.	CP008800.1	Plasmid	NCBI_complete_genomes
Klebsiella sp.	CP008828.1	Plasmid	NCBI_complete_genomes
Klebsiella sp.	CP008829.1	Plasmid	NCBI_complete_genomes
Klebsiella sp.	CP008830.1	Plasmid	NCBI_complete_genomes
Klebsiella sp.	CP008832.1	Plasmid	NCBI_complete_genomes
Klebsiella sp.	CP008833.1	Plasmid	NCBI_complete_genomes
Klebsiella sp.	CP008842.1	Plasmid	NCBI_complete_genomes
Klebsiella sp.	CP008843.1	Plasmid	NCBI_complete_genomes
Klebsiella sp.	CP008844.1	Plasmid	NCBI_complete_genomes
Klebsiella sp.	CP008845.1	Plasmid	NCBI_complete_genomes
Klebsiella sp.	CP008930.1	Plasmid	NCBI_complete_genomes
Klebsiella sp.	CP008931.1	Plasmid	NCBI_complete_genomes
Klebsiella sp.	CP008932.1	Plasmid	NCBI_complete_genomes
Klebsiella sp.	CP008933.1	Plasmid	NCBI_complete_genomes
Klebsiella sp.	CP009115.1	Plasmid	NCBI_complete_genomes
Klebsiella sp.	CP009116.1	Plasmid	NCBI_complete_genomes
Klebsiella sp.	CP009275.1	Plasmid	NCBI_complete_genomes
Klebsiella sp.	CP009276.1	Plasmid	NCBI_complete_genomes
Klebsiella sp.	CP009772.1	Plasmid	NCBI_complete_genomes
Klebsiella sp.	CP009773.1	Plasmid	NCBI_complete_genomes
Klebsiella sp.	CP009774.1	Plasmid	NCBI_complete_genomes
Klebsiella sp.	CP009776.1	Plasmid	NCBI_complete_genomes
Klebsiella sp.	CP009777.1	Plasmid	NCBI_complete_genomes
Klebsiella sp.	CP009778.1	Plasmid	NCBI_complete_genomes
Klebsiella sp.	CP009864.1	Plasmid	NCBI_complete_genomes
Klebsiella sp.	CP009865.1	Plasmid	NCBI_complete_genomes
Klebsiella sp.	CP009873.1	Plasmid	NCBI_complete_genomes
Klebsiella sp.	CP009874.1	Plasmid	NCBI_complete_genomes

Klebsiella sp.	CP009875.1	Plasmid	NCBI_complete_genomes
Klebsiella sp.	CP009877.1	Plasmid	NCBI_complete_genomes
Klebsiella sp.	CP009878.1	Plasmid	NCBI_complete_genomes
Klebsiella sp.	CP009879.1	Plasmid	NCBI_complete_genomes
Klebsiella sp.	CP010393.1	Plasmid	NCBI_complete_genomes
Klebsiella sp.	CP010394.1	Plasmid	NCBI_complete_genomes
Klebsiella sp.	CP010395.1	Plasmid	NCBI_complete_genomes
Klebsiella sp.	CP010396.1	Plasmid	NCBI_complete_genomes
Klebsiella sp.	CP010574.1	Plasmid	NCBI_complete_genomes
Klebsiella sp.	CP010575.1	Plasmid	NCBI_complete_genomes
Klebsiella sp.	CP010576.1	Plasmid	NCBI_complete_genomes
Klebsiella sp.	CP011314.1	Plasmid	NCBI_complete_genomes
Klebsiella sp.	CP011315.1	Plasmid	NCBI_complete_genomes
Klebsiella sp.	CP011316.1	Plasmid	NCBI_complete_genomes
Klebsiella sp.	CP011540.1	Plasmid	NCBI_complete_genomes
Klebsiella sp.	CP011573.1	Plasmid	NCBI_complete_genomes
Klebsiella sp.	CP011575.1	Plasmid	NCBI_complete_genomes
Klebsiella sp.	CP011576.1	Plasmid	NCBI_complete_genomes
Klebsiella sp.	CP011577.1	Plasmid	NCBI_complete_genomes
Klebsiella sp.	CP011592.1	Plasmid	NCBI_complete_genomes
Klebsiella sp.	CP011593.1	Plasmid	NCBI_complete_genomes
Klebsiella sp.	CP011594.1	Plasmid	NCBI_complete_genomes
Klebsiella sp.	CP011595.1	Plasmid	NCBI_complete_genomes
Klebsiella sp.	CP011596.1	Plasmid	NCBI_complete_genomes
Klebsiella sp.	CP011613.1	Plasmid	NCBI_complete_genomes
Klebsiella sp.	CP011614.1	Plasmid	NCBI_complete_genomes
Klebsiella sp.	CP011615.1	Plasmid	NCBI_complete_genomes
Klebsiella sp.	CP011616.1	Plasmid	NCBI_complete_genomes
Klebsiella sp.	CP011617.1	Plasmid	NCBI_complete_genomes
Klebsiella sp.	CP011619.1	Plasmid	NCBI_complete_genomes
Klebsiella sp.	CP011620.1	Plasmid	NCBI_complete_genomes
Klebsiella sp.	CP011621.1	Plasmid	NCBI_complete_genomes
Klebsiella sp.	CP011622.1	Plasmid	NCBI_complete_genomes
Klebsiella sp.	CP011623.1	Plasmid	NCBI_complete_genomes
Klebsiella sp.	CP011625.1	Plasmid	NCBI_complete_genomes
Klebsiella sp.	CP011626.1	Plasmid	NCBI_complete_genomes
Klebsiella sp.	CP011627.1	Plasmid	NCBI_complete_genomes
Klebsiella sp.	CP011628.1	Plasmid	NCBI_complete_genomes
Klebsiella sp.	CP011629.1	Plasmid	NCBI_complete_genomes
Klebsiella sp.	CP011630.1	Plasmid	NCBI_complete_genomes
Klebsiella sp.	CP011631.1	Plasmid	NCBI_complete_genomes
Klebsiella sp.	CP011632.1	Plasmid	NCBI_complete_genomes
Klebsiella sp.	CP011633.1	Plasmid	NCBI_complete_genomes
Klebsiella sp.	CP011634.1	Plasmid	NCBI_complete_genomes
Klebsiella sp.	CP011635.1	Plasmid	NCBI_complete_genomes
Klebsiella sp.	CP011643.1	Plasmid	NCBI_complete_genomes
Klebsiella sp.	CP011644.1	Plasmid	NCBI_complete_genomes
Klebsiella sp.	CP011645.1	Plasmid	NCBI_complete_genomes
Klebsiella sp.	CP011646.1	Plasmid	NCBI_complete_genomes
Klebsiella sp.	CP011839.1	Plasmid	NCBI_complete_genomes
Klebsiella sp.	CP011977.1	Plasmid	NCBI_complete_genomes
Klebsiella sp.	CP011978.1	Plasmid	NCBI_complete_genomes
Klebsiella sp.	CP011979.1	Plasmid	NCBI_complete_genomes
Klebsiella sp.	CP011981.1	Plasmid	NCBI_complete_genomes
Klebsiella sp.	CP011982.1	Plasmid	NCBI_complete_genomes
Klebsiella sp.	CP011983.1	Plasmid	NCBI_complete_genomes
Klebsiella sp.	CP011984.1	Plasmid	NCBI_complete_genomes
Klebsiella sp.	CP011986.1	Plasmid	NCBI_complete_genomes
Klebsiella sp.	CP011987.1	Plasmid	NCBI_complete_genomes
Klebsiella sp.	CP011988.1	Plasmid	NCBI_complete_genomes
Klebsiella sp.	CP011990.1	Plasmid	NCBI_complete_genomes
Klebsiella sp.	CP011991.1	Plasmid	NCBI_complete_genomes
Klebsiella sp.	CP011992.1	Plasmid	NCBI_complete_genomes
Klebsiella sp.	CP011993.1	Plasmid	NCBI_complete_genomes
Klebsiella sp.	CP012427.1	Plasmid	NCBI_complete_genomes
Klebsiella sp.	CP012428.1	Plasmid	NCBI_complete_genomes
Klebsiella sp.	CP012429.1	Plasmid	NCBI_complete_genomes
Klebsiella sp.	CP012562.1	Plasmid	NCBI_complete_genomes
Klebsiella sp.	CP012563.1	Plasmid	NCBI_complete_genomes
Klebsiella sp.	CP012564.1	Plasmid	NCBI_complete_genomes
Klebsiella sp.	CP012565.1	Plasmid	NCBI_complete_genomes
Klebsiella sp.	CP012566.1	Plasmid	NCBI_complete_genomes

Klebsiella sp.	CP012567.1	Plasmid	NCBI_complete_genomes
Klebsiella sp.	CP012569.1	Plasmid	NCBI_complete_genomes
Klebsiella sp.	CP012570.1	Plasmid	NCBI_complete_genomes
Klebsiella sp.	CP012571.1	Plasmid	NCBI_complete_genomes
Klebsiella sp.	CP012572.1	Plasmid	NCBI_complete_genomes
Klebsiella sp.	CP012754.1	Plasmid	NCBI_complete_genomes
Klebsiella sp.	CP012755.1	Plasmid	NCBI_complete_genomes
Klebsiella sp.	CP012884.1	Plasmid	NCBI_complete_genomes
Klebsiella sp.	CP012988.3	Plasmid	NCBI_complete_genomes
Klebsiella sp.	CP012989.1	Plasmid	NCBI_complete_genomes
Klebsiella sp.	CP012990.1	Plasmid	NCBI_complete_genomes
Klebsiella sp.	CP012991.1	Plasmid	NCBI_complete_genomes
Klebsiella sp.	CP012993.2	Plasmid	NCBI_complete_genomes
Klebsiella sp.	CP012994.1	Plasmid	NCBI_complete_genomes
Klebsiella sp.	CP012995.1	Plasmid	NCBI_complete_genomes
Klebsiella sp.	CP013321.1	Plasmid	NCBI_complete_genomes
Klebsiella sp.	CP013323.1	Plasmid	NCBI_complete_genomes
Klebsiella sp.	CP013324.1	Plasmid	NCBI_complete_genomes
Klebsiella sp.	CP013325.1	Plasmid	NCBI_complete_genomes
Klebsiella sp.	CP013326.1	Plasmid	NCBI_complete_genomes
Klebsiella sp.	CP013712.1	Plasmid	NCBI_complete_genomes
Klebsiella sp.	CP013713.1	Plasmid	NCBI_complete_genomes
Klebsiella sp.	CP014005.1	Plasmid	NCBI_complete_genomes
Klebsiella sp.	CP014006.1	Plasmid	NCBI_complete_genomes
Klebsiella sp.	CP014009.1	Plasmid	NCBI_complete_genomes
Klebsiella sp.	CP014011.1	Plasmid	NCBI_complete_genomes
Klebsiella sp.	CP014072.1	Plasmid	NCBI_complete_genomes
Klebsiella sp.	CP014073.1	Plasmid	NCBI_complete_genomes
Klebsiella sp.	CP014121.1	Plasmid	NCBI_complete_genomes
Klebsiella sp.	CP014122.1	Plasmid	NCBI_complete_genomes
Klebsiella sp.	CP014124.1	Plasmid	NCBI_complete_genomes
Klebsiella sp.	CP014125.1	Plasmid	NCBI_complete_genomes
Klebsiella sp.	CP014295.1	Plasmid	NCBI_complete_genomes
Klebsiella sp.	CP014296.1	Plasmid	NCBI_complete_genomes
Klebsiella sp.	CP014297.1	Plasmid	NCBI_complete_genomes
Klebsiella sp.	CP014298.1	Plasmid	NCBI_complete_genomes
Klebsiella sp.	CP014299.1	Plasmid	NCBI_complete_genomes
Klebsiella sp.	CP014300.1	Plasmid	NCBI_complete_genomes
Klebsiella sp.	CP014305.2	Plasmid	NCBI_complete_genomes
Klebsiella sp.	CP014648.1	Plasmid	NCBI_complete_genomes
Klebsiella sp.	CP014649.1	Plasmid	NCBI_complete_genomes
Klebsiella sp.	CP014650.1	Plasmid	NCBI_complete_genomes
Klebsiella sp.	CP014697.2	Plasmid	NCBI_complete_genomes
Klebsiella sp.	CP014698.2	Plasmid	NCBI_complete_genomes
Klebsiella sp.	CP014747.1	Plasmid	NCBI_complete_genomes
Klebsiella sp.	CP014756.1	Plasmid	NCBI_complete_genomes
Klebsiella sp.	CP014757.1	Plasmid	NCBI_complete_genomes
Klebsiella sp.	CP014758.1	Plasmid	NCBI_complete_genomes
Klebsiella sp.	CP014763.1	Plasmid	NCBI_complete_genomes
Klebsiella sp.	CP014764.1	Plasmid	NCBI_complete_genomes
Klebsiella sp.	CP014765.1	Plasmid	NCBI_complete_genomes
Klebsiella sp.	CP015026.1	Plasmid	NCBI_complete_genomes
Klebsiella sp.	CP015131.1	Plasmid	NCBI_complete_genomes
Klebsiella sp.	CP015132.1	Plasmid	NCBI_complete_genomes
Klebsiella sp.	CP015133.1	Plasmid	NCBI_complete_genomes
Klebsiella sp.	CP015135.1	Plasmid	NCBI_complete_genomes
Klebsiella sp.	CP015383.1	Plasmid	NCBI_complete_genomes
Klebsiella sp.	CP015384.1	Plasmid	NCBI_complete_genomes
Klebsiella sp.	CP015386.1	Plasmid	NCBI_complete_genomes
Klebsiella sp.	CP015387.1	Plasmid	NCBI_complete_genomes
Klebsiella sp.	CP015388.1	Plasmid	NCBI_complete_genomes
Klebsiella sp.	CP015389.1	Plasmid	NCBI_complete_genomes
Klebsiella sp.	CP015390.1	Plasmid	NCBI_complete_genomes
Klebsiella sp.	CP015391.1	Plasmid	NCBI_complete_genomes
Klebsiella sp.	CP015393.1	Plasmid	NCBI_complete_genomes
Klebsiella sp.	CP015394.1	Plasmid	NCBI_complete_genomes
Klebsiella sp.	CP015395.1	Plasmid	NCBI_complete_genomes
Klebsiella sp.	CP015396.1	Plasmid	NCBI_complete_genomes
Klebsiella sp.	CP015397.1	Plasmid	NCBI_complete_genomes
Klebsiella sp.	CP015501.1	Plasmid	NCBI_complete_genomes
Klebsiella sp.	CP015502.1	Plasmid	NCBI_complete_genomes
Klebsiella sp.	CP015503.1	Plasmid	NCBI_complete_genomes

Klebsiella sp.	CP015504.1	Plasmid	NCBI_complete_genomes
Klebsiella sp.	CP015505.1	Plasmid	NCBI_complete_genomes
Klebsiella sp.	CP015754.1	Plasmid	NCBI_complete_genomes
Klebsiella sp.	CP015755.1	Plasmid	NCBI_complete_genomes
Klebsiella sp.	CP015823.1	Plasmid	NCBI_complete_genomes
Klebsiella sp.	CP015824.1	Plasmid	NCBI_complete_genomes
Klebsiella sp.	CP015825.1	Plasmid	NCBI_complete_genomes
Klebsiella sp.	CP015991.1	Plasmid	NCBI_complete_genomes
Klebsiella sp.	CP016160.1	Plasmid	NCBI_complete_genomes
Klebsiella sp.	CP016161.1	Plasmid	NCBI_complete_genomes
Klebsiella sp.	CP016810.1	Plasmid	NCBI_complete_genomes
Klebsiella sp.	CP016812.1	Plasmid	NCBI_complete_genomes
Klebsiella sp.	CP016815.1	Plasmid	NCBI_complete_genomes
Klebsiella sp.	CP016919.1	Plasmid	NCBI_complete_genomes
Klebsiella sp.	CP016920.1	Plasmid	NCBI_complete_genomes
Klebsiella sp.	CP016921.1	Plasmid	NCBI_complete_genomes
Klebsiella sp.	CP016922.1	Plasmid	NCBI_complete_genomes
Klebsiella sp.	CP016924.1	Plasmid	NCBI_complete_genomes
Klebsiella sp.	CP016925.1	Plasmid	NCBI_complete_genomes
Klebsiella sp.	CP016927.1	Plasmid	NCBI_complete_genomes
Klebsiella sp.	CP017281.1	Plasmid	NCBI_complete_genomes
Klebsiella sp.	CP017282.1	Plasmid	NCBI_complete_genomes
Klebsiella sp.	CP017283.1	Plasmid	NCBI_complete_genomes
Klebsiella sp.	CP017285.1	Plasmid	NCBI_complete_genomes
Klebsiella sp.	CP017286.1	Plasmid	NCBI_complete_genomes
Klebsiella sp.	CP017287.1	Plasmid	NCBI_complete_genomes
Klebsiella sp.	CP017288.1	Plasmid	NCBI_complete_genomes
Klebsiella sp.	CP017386.1	Plasmid	NCBI_complete_genomes
Klebsiella sp.	CP017387.1	Plasmid	NCBI_complete_genomes
Klebsiella sp.	CP017388.1	Plasmid	NCBI_complete_genomes
Klebsiella sp.	CP017451.1	Plasmid	NCBI_complete_genomes
Klebsiella sp.	CP017452.1	Plasmid	NCBI_complete_genomes
Klebsiella sp.	CP017850.1	Plasmid	NCBI_complete_genomes
Klebsiella sp.	CP017851.1	Plasmid	NCBI_complete_genomes
Klebsiella sp.	CP017852.1	Plasmid	NCBI_complete_genomes
Klebsiella sp.	CP017853.1	Plasmid	NCBI_complete_genomes
Klebsiella sp.	CP017929.1	Plasmid	NCBI_complete_genomes
Klebsiella sp.	CP017930.1	Plasmid	NCBI_complete_genomes
Klebsiella sp.	CP017931.1	Plasmid	NCBI_complete_genomes
Klebsiella sp.	CP017932.1	Plasmid	NCBI_complete_genomes
Klebsiella sp.	CP017933.1	Plasmid	NCBI_complete_genomes
Klebsiella sp.	CP017935.1	Plasmid	NCBI_complete_genomes
Klebsiella sp.	CP017936.1	Plasmid	NCBI_complete_genomes
Klebsiella sp.	CP017937.1	Plasmid	NCBI_complete_genomes
Klebsiella sp.	CP017986.1	Plasmid	NCBI_complete_genomes
Klebsiella sp.	CP017987.1	Plasmid	NCBI_complete_genomes
Klebsiella sp.	CP017988.1	Plasmid	NCBI_complete_genomes
Klebsiella sp.	CP017989.1	Plasmid	NCBI_complete_genomes
Klebsiella sp.	CP018313.1	Plasmid	NCBI_complete_genomes
Klebsiella sp.	CP018314.1	Plasmid	NCBI_complete_genomes
Klebsiella sp.	CP018315.1	Plasmid	NCBI_complete_genomes
Klebsiella sp.	CP018316.1	Plasmid	NCBI_complete_genomes
Klebsiella sp.	CP018317.1	Plasmid	NCBI_complete_genomes
Klebsiella sp.	CP018318.1	Plasmid	NCBI_complete_genomes
Klebsiella sp.	CP018338.1	Plasmid	NCBI_complete_genomes
Klebsiella sp.	CP018339.1	Plasmid	NCBI_complete_genomes
Klebsiella sp.	CP018340.1	Plasmid	NCBI_complete_genomes
Klebsiella sp.	CP018341.1	Plasmid	NCBI_complete_genomes
Klebsiella sp.	CP018342.1	Plasmid	NCBI_complete_genomes
Klebsiella sp.	CP018343.1	Plasmid	NCBI_complete_genomes
Klebsiella sp.	CP018348.1	Plasmid	NCBI_complete_genomes
Klebsiella sp.	CP018350.1	Plasmid	NCBI_complete_genomes
Klebsiella sp.	CP018351.1	Plasmid	NCBI_complete_genomes
Klebsiella sp.	CP018353.1	Plasmid	NCBI_complete_genomes
Klebsiella sp.	CP018354.1	Plasmid	NCBI_complete_genomes
Klebsiella sp.	CP018355.1	Plasmid	NCBI_complete_genomes
Klebsiella sp.	CP018357.1	Plasmid	NCBI_complete_genomes
Klebsiella sp.	CP018358.1	Plasmid	NCBI_complete_genomes
Klebsiella sp.	CP018359.1	Plasmid	NCBI_complete_genomes
Klebsiella sp.	CP018360.1	Plasmid	NCBI_complete_genomes
Klebsiella sp.	CP018361.1	Plasmid	NCBI_complete_genomes
Klebsiella sp.	CP018365.1	Plasmid	NCBI_complete_genomes

Klebsiella sp.	CP018366.1	Plasmid	NCBI_complete_genomes
Klebsiella sp.	CP018423.1	Plasmid	NCBI_complete_genomes
Klebsiella sp.	CP018424.1	Plasmid	NCBI_complete_genomes
Klebsiella sp.	CP018425.1	Plasmid	NCBI_complete_genomes
Klebsiella sp.	CP018426.1	Plasmid	NCBI_complete_genomes
Klebsiella sp.	CP018429.1	Plasmid	NCBI_complete_genomes
Klebsiella sp.	CP018430.1	Plasmid	NCBI_complete_genomes
Klebsiella sp.	CP018431.1	Plasmid	NCBI_complete_genomes
Klebsiella sp.	CP018432.1	Plasmid	NCBI_complete_genomes
Klebsiella sp.	CP018433.1	Plasmid	NCBI_complete_genomes
Klebsiella sp.	CP018434.1	Plasmid	NCBI_complete_genomes
Klebsiella sp.	CP018435.1	Plasmid	NCBI_complete_genomes
Klebsiella sp.	CP018436.1	Plasmid	NCBI_complete_genomes
Klebsiella sp.	CP018439.1	Plasmid	NCBI_complete_genomes
Klebsiella sp.	CP018440.1	Plasmid	NCBI_complete_genomes
Klebsiella sp.	CP018441.1	Plasmid	NCBI_complete_genomes
Klebsiella sp.	CP018442.1	Plasmid	NCBI_complete_genomes
Klebsiella sp.	CP018443.1	Plasmid	NCBI_complete_genomes
Klebsiella sp.	CP018444.1	Plasmid	NCBI_complete_genomes
Klebsiella sp.	CP018445.1	Plasmid	NCBI_complete_genomes
Klebsiella sp.	CP018446.1	Plasmid	NCBI_complete_genomes
Klebsiella sp.	CP018448.1	Plasmid	NCBI_complete_genomes
Klebsiella sp.	CP018449.1	Plasmid	NCBI_complete_genomes
Klebsiella sp.	CP018451.1	Plasmid	NCBI_complete_genomes
Klebsiella sp.	CP018452.1	Plasmid	NCBI_complete_genomes
Klebsiella sp.	CP018453.1	Plasmid	NCBI_complete_genomes
Klebsiella sp.	CP018455.1	Plasmid	NCBI_complete_genomes
Klebsiella sp.	CP018459.1	Plasmid	NCBI_complete_genomes
Klebsiella sp.	CP018460.1	Plasmid	NCBI_complete_genomes
Klebsiella sp.	CP018461.1	Plasmid	NCBI_complete_genomes
Klebsiella sp.	CP018462.1	Plasmid	NCBI_complete_genomes
Klebsiella sp.	CP018665.1	Plasmid	NCBI_complete_genomes
Klebsiella sp.	CP018666.1	Plasmid	NCBI_complete_genomes
Klebsiella sp.	CP018667.1	Plasmid	NCBI_complete_genomes
Klebsiella sp.	CP018668.1	Plasmid	NCBI_complete_genomes
Klebsiella sp.	CP018669.1	Plasmid	NCBI_complete_genomes
Klebsiella sp.	CP018670.1	Plasmid	NCBI_complete_genomes
Klebsiella sp.	CP018672.1	Plasmid	NCBI_complete_genomes
Klebsiella sp.	CP018674.1	Plasmid	NCBI_complete_genomes
Klebsiella sp.	CP018675.1	Plasmid	NCBI_complete_genomes
Klebsiella sp.	CP018687.1	Plasmid	NCBI_complete_genomes
Klebsiella sp.	CP018688.1	Plasmid	NCBI_complete_genomes
Klebsiella sp.	CP018689.1	Plasmid	NCBI_complete_genomes
Klebsiella sp.	CP018690.1	Plasmid	NCBI_complete_genomes
Klebsiella sp.	CP018691.1	Plasmid	NCBI_complete_genomes
Klebsiella sp.	CP018693.1	Plasmid	NCBI_complete_genomes
Klebsiella sp.	CP018694.1	Plasmid	NCBI_complete_genomes
Klebsiella sp.	CP018696.1	Plasmid	NCBI_complete_genomes
Klebsiella sp.	CP018697.1	Plasmid	NCBI_complete_genomes
Klebsiella sp.	CP018698.1	Plasmid	NCBI_complete_genomes
Klebsiella sp.	CP018699.1	Plasmid	NCBI_complete_genomes
Klebsiella sp.	CP018700.1	Plasmid	NCBI_complete_genomes
Klebsiella sp.	CP018702.1	Plasmid	NCBI_complete_genomes
Klebsiella sp.	CP018703.1	Plasmid	NCBI_complete_genomes
Klebsiella sp.	CP018704.1	Plasmid	NCBI_complete_genomes
Klebsiella sp.	CP018705.1	Plasmid	NCBI_complete_genomes
Klebsiella sp.	CP018706.1	Plasmid	NCBI_complete_genomes
Klebsiella sp.	CP018708.1	Plasmid	NCBI_complete_genomes
Klebsiella sp.	CP018709.1	Plasmid	NCBI_complete_genomes
Klebsiella sp.	CP018710.1	Plasmid	NCBI_complete_genomes
Klebsiella sp.	CP018711.1	Plasmid	NCBI_complete_genomes
Klebsiella sp.	CP018712.1	Plasmid	NCBI_complete_genomes
Klebsiella sp.	CP018714.1	Plasmid	NCBI_complete_genomes
Klebsiella sp.	CP018715.1	Plasmid	NCBI_complete_genomes
Klebsiella sp.	CP018716.1	Plasmid	NCBI_complete_genomes
Klebsiella sp.	CP018717.1	Plasmid	NCBI_complete_genomes
Klebsiella sp.	CP018718.1	Plasmid	NCBI_complete_genomes
Klebsiella sp.	CP018720.1	Plasmid	NCBI_complete_genomes
Klebsiella sp.	CP018721.1	Plasmid	NCBI_complete_genomes
Klebsiella sp.	CP018722.1	Plasmid	NCBI_complete_genomes
Klebsiella sp.	CP018723.1	Plasmid	NCBI_complete_genomes
Klebsiella sp.	CP018724.1	Plasmid	NCBI_complete_genomes

Klebsiella sp.	CP018736.1	Plasmid	NCBI_complete_genomes
Klebsiella sp.	CP018737.1	Plasmid	NCBI_complete_genomes
Klebsiella sp.	CP018738.1	Plasmid	NCBI_complete_genomes
Klebsiella sp.	CP018739.1	Plasmid	NCBI_complete_genomes
Klebsiella sp.	CP018740.1	Plasmid	NCBI_complete_genomes
Klebsiella sp.	CP018817.1	Plasmid	NCBI_complete_genomes
Klebsiella sp.	CP018818.1	Plasmid	NCBI_complete_genomes
Klebsiella sp.	CP018819.1	Plasmid	NCBI_complete_genomes
Klebsiella sp.	CP018884.1	Plasmid	NCBI_complete_genomes
Klebsiella sp.	CP018886.1	Plasmid	NCBI_complete_genomes
Klebsiella sp.	CP018887.1	Plasmid	NCBI_complete_genomes
Klebsiella sp.	CP019048.1	Plasmid	NCBI_complete_genomes
Klebsiella sp.	CP019049.1	Plasmid	NCBI_complete_genomes
Klebsiella sp.	CP019050.1	Plasmid	NCBI_complete_genomes
Klebsiella sp.	CP019078.1	Plasmid	NCBI_complete_genomes
Klebsiella sp.	CP019080.1	Plasmid	NCBI_complete_genomes
Klebsiella sp.	CP019161.1	Plasmid	NCBI_complete_genomes
Klebsiella sp.	CP019220.1	Plasmid	NCBI_complete_genomes
Klebsiella sp.	CP019773.1	Plasmid	NCBI_complete_genomes
Klebsiella sp.	CP019774.1	Plasmid	NCBI_complete_genomes
Klebsiella sp.	CP019775.1	Plasmid	NCBI_complete_genomes
Klebsiella sp.	CP019776.1	Plasmid	NCBI_complete_genomes
Klebsiella sp.	CP020062.1	Plasmid	NCBI_complete_genomes
Klebsiella sp.	CP020063.1	Plasmid	NCBI_complete_genomes
Klebsiella sp.	CP020064.1	Plasmid	NCBI_complete_genomes
Klebsiella sp.	CP020065.1	Plasmid	NCBI_complete_genomes
Klebsiella sp.	CP020066.1	Plasmid	NCBI_complete_genomes
Klebsiella sp.	CP020068.1	Plasmid	NCBI_complete_genomes
Klebsiella sp.	CP020069.1	Plasmid	NCBI_complete_genomes
Klebsiella sp.	CP020070.1	Plasmid	NCBI_complete_genomes
Klebsiella sp.	CP020072.1	Plasmid	NCBI_complete_genomes
Klebsiella sp.	CP020073.1	Plasmid	NCBI_complete_genomes
Klebsiella sp.	CP020074.1	Plasmid	NCBI_complete_genomes
Klebsiella sp.	CP020075.1	Plasmid	NCBI_complete_genomes
Klebsiella sp.	CP020109.1	Plasmid	NCBI_complete_genomes
Klebsiella sp.	CP020110.1	Plasmid	NCBI_complete_genomes
Klebsiella sp.	CP020111.1	Plasmid	NCBI_complete_genomes
Klebsiella sp.	CP020112.1	Plasmid	NCBI_complete_genomes
Klebsiella sp.	CP020359.1	Plasmid	NCBI_complete_genomes
Klebsiella sp.	CP020838.1	Plasmid	NCBI_complete_genomes
Klebsiella sp.	CP020839.1	Plasmid	NCBI_complete_genomes
Klebsiella sp.	CP020840.1	Plasmid	NCBI_complete_genomes
Klebsiella sp.	CP020842.1	Plasmid	NCBI_complete_genomes
Klebsiella sp.	CP020843.1	Plasmid	NCBI_complete_genomes
Klebsiella sp.	CP020844.1	Plasmid	NCBI_complete_genomes
Klebsiella sp.	CP020845.1	Plasmid	NCBI_complete_genomes
Klebsiella sp.	CP020846.1	Plasmid	NCBI_complete_genomes
Klebsiella sp.	CP020848.1	Plasmid	NCBI_complete_genomes
Klebsiella sp.	CP020849.1	Plasmid	NCBI_complete_genomes
Klebsiella sp.	CP020850.1	Plasmid	NCBI_complete_genomes
Klebsiella sp.	CP020851.1	Plasmid	NCBI_complete_genomes
Klebsiella sp.	CP020852.1	Plasmid	NCBI_complete_genomes
Klebsiella sp.	CP020854.1	Plasmid	NCBI_complete_genomes
Klebsiella sp.	CP020855.1	Plasmid	NCBI_complete_genomes
Klebsiella sp.	CP020856.1	Plasmid	NCBI_complete_genomes
Klebsiella sp.	CP020902.1	Plasmid	NCBI_complete_genomes
Klebsiella sp.	CP020903.1	Plasmid	NCBI_complete_genomes
Klebsiella sp.	CP020904.1	Plasmid	NCBI_complete_genomes
Klebsiella sp.	CP020905.1	Plasmid	NCBI_complete_genomes
Klebsiella sp.	CP021166.1	Plasmid	NCBI_complete_genomes
Klebsiella sp.	CP021540.1	Plasmid	NCBI_complete_genomes
Klebsiella sp.	CP021541.1	Plasmid	NCBI_complete_genomes
Klebsiella sp.	CP021542.1	Plasmid	NCBI_complete_genomes
Klebsiella sp.	CP021543.1	Plasmid	NCBI_complete_genomes
Klebsiella sp.	CP021544.1	Plasmid	NCBI_complete_genomes
Klebsiella sp.	CP021545.1	Plasmid	NCBI_complete_genomes
Klebsiella sp.	CP021546.1	Plasmid	NCBI_complete_genomes
Klebsiella sp.	CP021547.1	Plasmid	NCBI_complete_genomes
Klebsiella sp.	CP021548.1	Plasmid	NCBI_complete_genomes
Klebsiella sp.	CP021686.1	Plasmid	NCBI_complete_genomes
Klebsiella sp.	CP021687.1	Plasmid	NCBI_complete_genomes
Klebsiella sp.	CP021688.1	Plasmid	NCBI_complete_genomes

Klebsiella sp.	CP021697.1	Plasmid	NCBI_complete_genomes
Klebsiella sp.	CP021698.1	Plasmid	NCBI_complete_genomes
Klebsiella sp.	CP021699.1	Plasmid	NCBI_complete_genomes
Klebsiella sp.	CP021700.1	Plasmid	NCBI_complete_genomes
Klebsiella sp.	CP021709.1	Plasmid	NCBI_complete_genomes
Klebsiella sp.	CP021710.1	Plasmid	NCBI_complete_genomes
Klebsiella sp.	CP021711.1	Plasmid	NCBI_complete_genomes
Klebsiella sp.	CP021712.1	Plasmid	NCBI_complete_genomes
Klebsiella sp.	CP021713.1	Plasmid	NCBI_complete_genomes
Klebsiella sp.	CP021714.1	Plasmid	NCBI_complete_genomes
Klebsiella sp.	CP021715.1	Plasmid	NCBI_complete_genomes
Klebsiella sp.	CP021716.1	Plasmid	NCBI_complete_genomes
Klebsiella sp.	CP021717.1	Plasmid	NCBI_complete_genomes
Klebsiella sp.	CP021741.1	Plasmid	NCBI_complete_genomes
Klebsiella sp.	CP021742.1	Plasmid	NCBI_complete_genomes
Klebsiella sp.	CP021743.1	Plasmid	NCBI_complete_genomes
Klebsiella sp.	CP021752.1	Plasmid	NCBI_complete_genomes
Klebsiella sp.	CP021753.1	Plasmid	NCBI_complete_genomes
Klebsiella sp.	CP021754.1	Plasmid	NCBI_complete_genomes
Klebsiella sp.	CP021755.1	Plasmid	NCBI_complete_genomes
Klebsiella sp.	CP021756.1	Plasmid	NCBI_complete_genomes
Klebsiella sp.	CP021758.1	Plasmid	NCBI_complete_genomes
Klebsiella sp.	CP021759.1	Plasmid	NCBI_complete_genomes
Klebsiella sp.	CP021760.1	Plasmid	NCBI_complete_genomes
Klebsiella sp.	CP021761.1	Plasmid	NCBI_complete_genomes
Klebsiella sp.	CP021834.1	Plasmid	NCBI_complete_genomes
Klebsiella sp.	CP021835.1	Plasmid	NCBI_complete_genomes
Klebsiella sp.	CP021836.1	Plasmid	NCBI_complete_genomes
Klebsiella sp.	CP021837.1	Plasmid	NCBI_complete_genomes
Klebsiella sp.	CP021856.1	Plasmid	NCBI_complete_genomes
Klebsiella sp.	CP021857.1	Plasmid	NCBI_complete_genomes
Klebsiella sp.	CP021858.1	Plasmid	NCBI_complete_genomes
Klebsiella sp.	CP021860.1	Plasmid	NCBI_complete_genomes
Klebsiella sp.	CP021861.1	Plasmid	NCBI_complete_genomes
Klebsiella sp.	CP021940.1	Plasmid	NCBI_complete_genomes
Klebsiella sp.	CP021941.1	Plasmid	NCBI_complete_genomes
Klebsiella sp.	CP021942.1	Plasmid	NCBI_complete_genomes
Klebsiella sp.	CP021943.1	Plasmid	NCBI_complete_genomes
Klebsiella sp.	CP021945.1	Plasmid	NCBI_complete_genomes
Klebsiella sp.	CP021946.1	Plasmid	NCBI_complete_genomes
Klebsiella sp.	CP021947.1	Plasmid	NCBI_complete_genomes
Klebsiella sp.	CP021948.1	Plasmid	NCBI_complete_genomes
Klebsiella sp.	CP021949.1	Plasmid	NCBI_complete_genomes
Klebsiella sp.	CP021951.1	Plasmid	NCBI_complete_genomes
Klebsiella sp.	CP021952.1	Plasmid	NCBI_complete_genomes
Klebsiella sp.	CP021953.1	Plasmid	NCBI_complete_genomes
Klebsiella sp.	CP021954.1	Plasmid	NCBI_complete_genomes
Klebsiella sp.	CP021956.1	Plasmid	NCBI_complete_genomes
Klebsiella sp.	CP021957.1	Plasmid	NCBI_complete_genomes
Klebsiella sp.	CP021958.1	Plasmid	NCBI_complete_genomes
Klebsiella sp.	CP021959.1	Plasmid	NCBI_complete_genomes
Klebsiella sp.	CP021961.1	Plasmid	NCBI_complete_genomes
Klebsiella sp.	CP021962.1	Plasmid	NCBI_complete_genomes
Klebsiella sp.	CP022125.1	Plasmid	NCBI_complete_genomes
Klebsiella sp.	CP022126.1	Plasmid	NCBI_complete_genomes
Klebsiella sp.	CP022128.1	Plasmid	NCBI_complete_genomes
Klebsiella sp.	CP022144.1	Plasmid	NCBI_complete_genomes
Klebsiella sp.	CP022145.1	Plasmid	NCBI_complete_genomes
Klebsiella sp.	CP022146.1	Plasmid	NCBI_complete_genomes
Klebsiella sp.	CP022147.1	Plasmid	NCBI_complete_genomes
Klebsiella sp.	CP022349.1	Plasmid	NCBI_complete_genomes
Klebsiella sp.	CP022350.1	Plasmid	NCBI_complete_genomes
Klebsiella sp.	CP022351.1	Plasmid	NCBI_complete_genomes
Klebsiella sp.	CP022574.1	Plasmid	NCBI_complete_genomes
Klebsiella sp.	CP022575.1	Plasmid	NCBI_complete_genomes
Klebsiella sp.	CP022576.1	Plasmid	NCBI_complete_genomes
Klebsiella sp.	CP022612.1	Plasmid	NCBI_complete_genomes
Klebsiella sp.	CP022613.1	Plasmid	NCBI_complete_genomes
Klebsiella sp.	CP022692.1	Plasmid	NCBI_complete_genomes
Klebsiella sp.	CP022693.1	Plasmid	NCBI_complete_genomes
Klebsiella sp.	CP022694.1	Plasmid	NCBI_complete_genomes
Klebsiella sp.	CP022824.1	Plasmid	NCBI_complete_genomes

Klebsiella sp.	CP022825.1	Plasmid	NCBI_complete_genomes
Klebsiella sp.	CP022826.1	Plasmid	NCBI_complete_genomes
Klebsiella sp.	CP023135.2	Plasmid	NCBI_complete_genomes
Klebsiella sp.	CP023136.2	Plasmid	NCBI_complete_genomes
Klebsiella sp.	CP023137.2	Plasmid	NCBI_complete_genomes
Klebsiella sp.	CP023186.1	Plasmid	NCBI_complete_genomes
Klebsiella sp.	CP023187.1	Plasmid	NCBI_complete_genomes
Klebsiella sp.	CP023188.1	Plasmid	NCBI_complete_genomes
Klebsiella sp.	CP023250.1	Plasmid	NCBI_complete_genomes
Klebsiella sp.	CP023251.1	Plasmid	NCBI_complete_genomes
Klebsiella sp.	CP023252.1	Plasmid	NCBI_complete_genomes
Klebsiella sp.	CP023253.1	Plasmid	NCBI_complete_genomes
Klebsiella sp.	CP023442.1	Plasmid	NCBI_complete_genomes
Klebsiella sp.	CP023443.1	Plasmid	NCBI_complete_genomes
Klebsiella sp.	CP023444.1	Plasmid	NCBI_complete_genomes
Klebsiella sp.	CP023479.1	Plasmid	NCBI_complete_genomes
Klebsiella sp.	CP023480.1	Plasmid	NCBI_complete_genomes
Klebsiella sp.	CP023488.1	Plasmid	NCBI_complete_genomes
Klebsiella sp.	CP023489.1	Plasmid	NCBI_complete_genomes
Klebsiella sp.	CP023503.1	Plasmid	NCBI_complete_genomes
Klebsiella sp.	CP023554.1	Plasmid	NCBI_complete_genomes
Klebsiella sp.	CP023555.1	Plasmid	NCBI_complete_genomes
Klebsiella sp.	CP023556.1	Plasmid	NCBI_complete_genomes
Klebsiella sp.	CP023557.1	Plasmid	NCBI_complete_genomes
Klebsiella sp.	CP023723.1	Plasmid	NCBI_complete_genomes
Klebsiella sp.	CP023724.1	Plasmid	NCBI_complete_genomes
Klebsiella sp.	CP023725.1	Plasmid	NCBI_complete_genomes
Klebsiella sp.	CP023906.1	Plasmid	NCBI_complete_genomes
Klebsiella sp.	CP023908.1	Plasmid	NCBI_complete_genomes
Klebsiella sp.	CP023909.1	Plasmid	NCBI_complete_genomes
Klebsiella sp.	CP023910.1	Plasmid	NCBI_complete_genomes
Klebsiella sp.	CP023911.1	Plasmid	NCBI_complete_genomes
Klebsiella sp.	CP023912.1	Plasmid	NCBI_complete_genomes
Klebsiella sp.	CP023914.1	Plasmid	NCBI_complete_genomes
Klebsiella sp.	CP023915.1	Plasmid	NCBI_complete_genomes
Klebsiella sp.	CP023916.1	Plasmid	NCBI_complete_genomes
Klebsiella sp.	CP023917.1	Plasmid	NCBI_complete_genomes
Klebsiella sp.	CP023918.1	Plasmid	NCBI_complete_genomes
Klebsiella sp.	CP023920.1	Plasmid	NCBI_complete_genomes
Klebsiella sp.	CP023921.1	Plasmid	NCBI_complete_genomes
Klebsiella sp.	CP023922.1	Plasmid	NCBI_complete_genomes
Klebsiella sp.	CP023923.1	Plasmid	NCBI_complete_genomes
Klebsiella sp.	CP023924.1	Plasmid	NCBI_complete_genomes
Klebsiella sp.	CP023926.1	Plasmid	NCBI_complete_genomes
Klebsiella sp.	CP023927.1	Plasmid	NCBI_complete_genomes
Klebsiella sp.	CP023928.1	Plasmid	NCBI_complete_genomes
Klebsiella sp.	CP023929.1	Plasmid	NCBI_complete_genomes
Klebsiella sp.	CP023930.1	Plasmid	NCBI_complete_genomes
Klebsiella sp.	CP023931.1	Plasmid	NCBI_complete_genomes
Klebsiella sp.	CP023932.1	Plasmid	NCBI_complete_genomes
Klebsiella sp.	CP023934.1	Plasmid	NCBI_complete_genomes
Klebsiella sp.	CP023935.1	Plasmid	NCBI_complete_genomes
Klebsiella sp.	CP023936.1	Plasmid	NCBI_complete_genomes
Klebsiella sp.	CP023937.1	Plasmid	NCBI_complete_genomes
Klebsiella sp.	CP023938.1	Plasmid	NCBI_complete_genomes
Klebsiella sp.	CP023939.1	Plasmid	NCBI_complete_genomes
Klebsiella sp.	CP023940.1	Plasmid	NCBI_complete_genomes
Klebsiella sp.	CP023942.1	Plasmid	NCBI_complete_genomes
Klebsiella sp.	CP023943.1	Plasmid	NCBI_complete_genomes
Klebsiella sp.	CP023944.1	Plasmid	NCBI_complete_genomes
Klebsiella sp.	CP023945.1	Plasmid	NCBI_complete_genomes
Klebsiella sp.	CP023947.1	Plasmid	NCBI_complete_genomes
Klebsiella sp.	CP023948.1	Plasmid	NCBI_complete_genomes
Klebsiella sp.	CP023950.1	Plasmid	NCBI_complete_genomes
Klebsiella sp.	CP023951.1	Plasmid	NCBI_complete_genomes
Klebsiella sp.	CP023952.1	Plasmid	NCBI_complete_genomes
Klebsiella sp.	CP023953.1	Plasmid	NCBI_complete_genomes
Klebsiella sp.	CP023954.1	Plasmid	NCBI_complete_genomes
Klebsiella sp.	CP023955.1	Plasmid	NCBI_complete_genomes
Klebsiella sp.	CP024039.1	Plasmid	NCBI_complete_genomes
Klebsiella sp.	CP024040.1	Plasmid	NCBI_complete_genomes
Klebsiella sp.	CP024041.1	Plasmid	NCBI_complete_genomes

Klebsiella sp.	CP024042.1	Plasmid	NCBI_complete_genomes
Klebsiella sp.	CP024043.1	Plasmid	NCBI_complete_genomes
Klebsiella sp.	CP024044.1	Plasmid	NCBI_complete_genomes
Klebsiella sp.	CP024192.1	Plasmid	NCBI_complete_genomes
Klebsiella sp.	CP024193.1	Plasmid	NCBI_complete_genomes
Klebsiella sp.	CP024194.1	Plasmid	NCBI_complete_genomes
Klebsiella sp.	CP024195.1	Plasmid	NCBI_complete_genomes
Klebsiella sp.	CP024196.1	Plasmid	NCBI_complete_genomes
Klebsiella sp.	CP024197.1	Plasmid	NCBI_complete_genomes
Klebsiella sp.	CP024430.1	Plasmid	NCBI_complete_genomes
Klebsiella sp.	CP024431.1	Plasmid	NCBI_complete_genomes
Klebsiella sp.	CP024432.1	Plasmid	NCBI_complete_genomes
Klebsiella sp.	CP024433.1	Plasmid	NCBI_complete_genomes
Klebsiella sp.	CP024434.1	Plasmid	NCBI_complete_genomes
Klebsiella sp.	CP024435.1	Plasmid	NCBI_complete_genomes
Klebsiella sp.	CP024436.1	Plasmid	NCBI_complete_genomes
Klebsiella sp.	CP024459.1	Plasmid	NCBI_complete_genomes
Klebsiella sp.	CP024460.1	Plasmid	NCBI_complete_genomes
Klebsiella sp.	CP024461.1	Plasmid	NCBI_complete_genomes
Klebsiella sp.	CP024462.1	Plasmid	NCBI_complete_genomes
Klebsiella sp.	CP024463.1	Plasmid	NCBI_complete_genomes
Klebsiella sp.	CP024464.1	Plasmid	NCBI_complete_genomes
Klebsiella sp.	CP024465.1	Plasmid	NCBI_complete_genomes
Klebsiella sp.	CP024483.1	Plasmid	NCBI_complete_genomes
Klebsiella sp.	CP024484.1	Plasmid	NCBI_complete_genomes
Klebsiella sp.	CP024485.1	Plasmid	NCBI_complete_genomes
Klebsiella sp.	CP024486.1	Plasmid	NCBI_complete_genomes
Klebsiella sp.	CP024487.1	Plasmid	NCBI_complete_genomes
Klebsiella sp.	CP024488.1	Plasmid	NCBI_complete_genomes
Klebsiella sp.	CP024490.1	Plasmid	NCBI_complete_genomes
Klebsiella sp.	CP024491.1	Plasmid	NCBI_complete_genomes
Klebsiella sp.	CP024492.1	Plasmid	NCBI_complete_genomes
Klebsiella sp.	CP024493.1	Plasmid	NCBI_complete_genomes
Klebsiella sp.	CP024494.1	Plasmid	NCBI_complete_genomes
Klebsiella sp.	CP024495.1	Plasmid	NCBI_complete_genomes
Klebsiella sp.	CP024497.1	Plasmid	NCBI_complete_genomes
Klebsiella sp.	CP024498.1	Plasmid	NCBI_complete_genomes
Klebsiella sp.	CP024500.1	Plasmid	NCBI_complete_genomes
Klebsiella sp.	CP024501.1	Plasmid	NCBI_complete_genomes
Klebsiella sp.	CP024502.1	Plasmid	NCBI_complete_genomes
Klebsiella sp.	CP024503.1	Plasmid	NCBI_complete_genomes
Klebsiella sp.	CP024505.1	Plasmid	NCBI_complete_genomes
Klebsiella sp.	CP024506.1	Plasmid	NCBI_complete_genomes
Klebsiella sp.	CP024507.1	Plasmid	NCBI_complete_genomes
Klebsiella sp.	CP024508.1	Plasmid	NCBI_complete_genomes
Klebsiella sp.	CP024509.1	Plasmid	NCBI_complete_genomes
Klebsiella sp.	CP024510.1	Plasmid	NCBI_complete_genomes
Klebsiella sp.	CP024511.1	Plasmid	NCBI_complete_genomes
Klebsiella sp.	CP024512.1	Plasmid	NCBI_complete_genomes
Klebsiella sp.	CP024513.1	Plasmid	NCBI_complete_genomes
Klebsiella sp.	CP024514.1	Plasmid	NCBI_complete_genomes
Klebsiella sp.	CP024516.1	Plasmid	NCBI_complete_genomes
Klebsiella sp.	CP024517.1	Plasmid	NCBI_complete_genomes
Klebsiella sp.	CP024518.1	Plasmid	NCBI_complete_genomes
Klebsiella sp.	CP024519.1	Plasmid	NCBI_complete_genomes
Klebsiella sp.	CP024520.1	Plasmid	NCBI_complete_genomes
Klebsiella sp.	CP024522.1	Plasmid	NCBI_complete_genomes
Klebsiella sp.	CP024523.1	Plasmid	NCBI_complete_genomes
Klebsiella sp.	CP024524.1	Plasmid	NCBI_complete_genomes
Klebsiella sp.	CP024525.1	Plasmid	NCBI_complete_genomes
Klebsiella sp.	CP024526.1	Plasmid	NCBI_complete_genomes
Klebsiella sp.	CP024527.1	Plasmid	NCBI_complete_genomes
Klebsiella sp.	CP024529.1	Plasmid	NCBI_complete_genomes
Klebsiella sp.	CP024530.1	Plasmid	NCBI_complete_genomes
Klebsiella sp.	CP024531.1	Plasmid	NCBI_complete_genomes
Klebsiella sp.	CP024532.1	Plasmid	NCBI_complete_genomes
Klebsiella sp.	CP024533.1	Plasmid	NCBI_complete_genomes
Klebsiella sp.	CP024534.1	Plasmid	NCBI_complete_genomes
Klebsiella sp.	CP024536.1	Plasmid	NCBI_complete_genomes
Klebsiella sp.	CP024537.1	Plasmid	NCBI_complete_genomes
Klebsiella sp.	CP024538.1	Plasmid	NCBI_complete_genomes
Klebsiella sp.	CP024539.1	Plasmid	NCBI_complete_genomes

Klebsiella sp.	CP024540.1	Plasmid	NCBI_complete_genomes
Klebsiella sp.	CP024541.1	Plasmid	NCBI_complete_genomes
Klebsiella sp.	CP024543.1	Plasmid	NCBI_complete_genomes
Klebsiella sp.	CP024544.1	Plasmid	NCBI_complete_genomes
Klebsiella sp.	CP024546.1	Plasmid	NCBI_complete_genomes
Klebsiella sp.	CP024547.1	Plasmid	NCBI_complete_genomes
Klebsiella sp.	CP024550.1	Plasmid	NCBI_complete_genomes
Klebsiella sp.	CP024551.1	Plasmid	NCBI_complete_genomes
Klebsiella sp.	CP024552.1	Plasmid	NCBI_complete_genomes
Klebsiella sp.	CP024553.1	Plasmid	NCBI_complete_genomes
Klebsiella sp.	CP024554.1	Plasmid	NCBI_complete_genomes
Klebsiella sp.	CP024555.1	Plasmid	NCBI_complete_genomes
Klebsiella sp.	CP024557.1	Plasmid	NCBI_complete_genomes
Klebsiella sp.	CP024558.1	Plasmid	NCBI_complete_genomes
Klebsiella sp.	CP024559.1	Plasmid	NCBI_complete_genomes
Klebsiella sp.	CP024560.1	Plasmid	NCBI_complete_genomes
Klebsiella sp.	CP024561.1	Plasmid	NCBI_complete_genomes
Klebsiella sp.	CP024562.1	Plasmid	NCBI_complete_genomes
Klebsiella sp.	CP024564.1	Plasmid	NCBI_complete_genomes
Klebsiella sp.	CP024565.1	Plasmid	NCBI_complete_genomes
Klebsiella sp.	CP024566.1	Plasmid	NCBI_complete_genomes
Klebsiella sp.	CP024567.1	Plasmid	NCBI_complete_genomes
Klebsiella sp.	CP024568.1	Plasmid	NCBI_complete_genomes
Klebsiella sp.	CP024569.1	Plasmid	NCBI_complete_genomes
Klebsiella sp.	CP024571.1	Plasmid	NCBI_complete_genomes
Klebsiella sp.	CP024572.1	Plasmid	NCBI_complete_genomes
Klebsiella sp.	CP024573.1	Plasmid	NCBI_complete_genomes
Klebsiella sp.	CP024574.1	Plasmid	NCBI_complete_genomes
Klebsiella sp.	CP024575.1	Plasmid	NCBI_complete_genomes
Klebsiella sp.	CP024579.1	Plasmid	NCBI_complete_genomes
Klebsiella sp.	CP024580.1	Plasmid	NCBI_complete_genomes
Klebsiella sp.	CP024581.1	Plasmid	NCBI_complete_genomes
Klebsiella sp.	CP024835.1	Plasmid	NCBI_complete_genomes
Klebsiella sp.	CP024836.1	Plasmid	NCBI_complete_genomes
Klebsiella sp.	CP024837.1	Plasmid	NCBI_complete_genomes
Klebsiella sp.	CP024839.1	Plasmid	NCBI_complete_genomes
Klebsiella sp.	CP024840.1	Plasmid	NCBI_complete_genomes
Klebsiella sp.	CP024841.1	Plasmid	NCBI_complete_genomes
Klebsiella sp.	CP024875.1	Plasmid	NCBI_complete_genomes
Klebsiella sp.	CP024876.1	Plasmid	NCBI_complete_genomes
Klebsiella sp.	CP024877.1	Plasmid	NCBI_complete_genomes
Klebsiella sp.	CP024878.1	Plasmid	NCBI_complete_genomes
Klebsiella sp.	CP024879.1	Plasmid	NCBI_complete_genomes
Klebsiella sp.	CP024884.1	Plasmid	NCBI_complete_genomes
Klebsiella sp.	CP024917.1	Plasmid	NCBI_complete_genomes
Klebsiella sp.	CP024918.1	Plasmid	NCBI_complete_genomes
Klebsiella sp.	CP024919.1	Plasmid	NCBI_complete_genomes
Klebsiella sp.	CP025006.1	Plasmid	NCBI_complete_genomes
Klebsiella sp.	CP025007.1	Plasmid	NCBI_complete_genomes
Klebsiella sp.	CP025009.1	Plasmid	NCBI_complete_genomes
Klebsiella sp.	CP025010.1	Plasmid	NCBI_complete_genomes
Klebsiella sp.	CP025038.1	Plasmid	NCBI_complete_genomes
Klebsiella sp.	CP025039.1	Plasmid	NCBI_complete_genomes
Klebsiella sp.	CP025040.1	Plasmid	NCBI_complete_genomes
Klebsiella sp.	CP025041.1	Plasmid	NCBI_complete_genomes
Klebsiella sp.	CP025042.1	Plasmid	NCBI_complete_genomes
Klebsiella sp.	CP025081.1	Plasmid	NCBI_complete_genomes
Klebsiella sp.	CP025141.1	Plasmid	NCBI_complete_genomes
Klebsiella sp.	CP025142.1	Plasmid	NCBI_complete_genomes
Klebsiella sp.	CP025144.1	Plasmid	NCBI_complete_genomes
Klebsiella sp.	CP025145.1	Plasmid	NCBI_complete_genomes
Klebsiella sp.	CP025147.1	Plasmid	NCBI_complete_genomes
Klebsiella sp.	CP025148.1	Plasmid	NCBI_complete_genomes
Klebsiella sp.	CP025212.1	Plasmid	NCBI_complete_genomes
Klebsiella sp.	CP025213.1	Plasmid	NCBI_complete_genomes
Klebsiella sp.	CP025214.1	Plasmid	NCBI_complete_genomes
Klebsiella sp.	CP025215.1	Plasmid	NCBI_complete_genomes
Klebsiella sp.	CP025457.1	Plasmid	NCBI_complete_genomes
Klebsiella sp.	CP025458.1	Plasmid	NCBI_complete_genomes
Klebsiella sp.	CP025459.1	Plasmid	NCBI_complete_genomes
Klebsiella sp.	CP025460.1	Plasmid	NCBI_complete_genomes
Klebsiella sp.	CP025462.1	Plasmid	NCBI_complete_genomes

Klebsiella sp.	CP025463.1	Plasmid	NCBI_complete_genomes
Klebsiella sp.	CP025464.1	Plasmid	NCBI_complete_genomes
Klebsiella sp.	CP025465.1	Plasmid	NCBI_complete_genomes
Klebsiella sp.	CP025467.1	Plasmid	NCBI_complete_genomes
Klebsiella sp.	CP025468.1	Plasmid	NCBI_complete_genomes
Klebsiella sp.	CP025469.1	Plasmid	NCBI_complete_genomes
Klebsiella sp.	CP025470.1	Plasmid	NCBI_complete_genomes
Klebsiella sp.	CP025516.1	Plasmid	NCBI_complete_genomes
Klebsiella sp.	CP025517.1	Plasmid	NCBI_complete_genomes
Klebsiella sp.	CP025632.1	Plasmid	NCBI_complete_genomes
Klebsiella sp.	CP025634.1	Plasmid	NCBI_complete_genomes
Klebsiella sp.	CP025635.1	Plasmid	NCBI_complete_genomes
Klebsiella sp.	CP025637.1	Plasmid	NCBI_complete_genomes
Klebsiella sp.	CP025638.1	Plasmid	NCBI_complete_genomes
Klebsiella sp.	CP025640.1	Plasmid	NCBI_complete_genomes
Klebsiella sp.	CP025642.1	Plasmid	NCBI_complete_genomes
Klebsiella sp.	CP025817.1	Plasmid	NCBI_complete_genomes
Klebsiella sp.	CP025818.1	Plasmid	NCBI_complete_genomes
Klebsiella sp.	CP025952.1	Plasmid	NCBI_complete_genomes
Klebsiella sp.	CP025964.2	Plasmid	NCBI_complete_genomes
Klebsiella sp.	CP025965.2	Plasmid	NCBI_complete_genomes
Klebsiella sp.	CP025966.2	Plasmid	NCBI_complete_genomes
Klebsiella sp.	CP026172.1	Plasmid	NCBI_complete_genomes
Klebsiella sp.	CP026173.1	Plasmid	NCBI_complete_genomes
Klebsiella sp.	CP026174.1	Plasmid	NCBI_complete_genomes
Klebsiella sp.	CP026175.1	Plasmid	NCBI_complete_genomes
Klebsiella sp.	CP026176.1	Plasmid	NCBI_complete_genomes
Klebsiella sp.	CP026179.1	Plasmid	NCBI_complete_genomes
Klebsiella sp.	CP026180.1	Plasmid	NCBI_complete_genomes
Klebsiella sp.	CP026181.1	Plasmid	NCBI_complete_genomes
Klebsiella sp.	CP026182.1	Plasmid	NCBI_complete_genomes
Klebsiella sp.	CP026183.1	Plasmid	NCBI_complete_genomes
Klebsiella sp.	CP026184.1	Plasmid	NCBI_complete_genomes
Klebsiella sp.	CP026185.1	Plasmid	NCBI_complete_genomes
Klebsiella sp.	CP026186.1	Plasmid	NCBI_complete_genomes
Klebsiella sp.	CP026270.1	Plasmid	NCBI_complete_genomes
Klebsiella sp.	CP026271.1	Plasmid	NCBI_complete_genomes
Klebsiella sp.	CP026272.1	Plasmid	NCBI_complete_genomes
Klebsiella sp.	CP026273.1	Plasmid	NCBI_complete_genomes
Klebsiella sp.	CP026274.1	Plasmid	NCBI_complete_genomes
Klebsiella sp.	CP026276.1	Plasmid	NCBI_complete_genomes
Klebsiella sp.	CP026277.1	Plasmid	NCBI_complete_genomes
Klebsiella sp.	CP026278.1	Plasmid	NCBI_complete_genomes
Klebsiella sp.	CP026279.1	Plasmid	NCBI_complete_genomes
Klebsiella sp.	CP026280.1	Plasmid	NCBI_complete_genomes
Klebsiella sp.	CP026281.1	Plasmid	NCBI_complete_genomes
Klebsiella sp.	CP026282.1	Plasmid	NCBI_complete_genomes
Klebsiella sp.	CP026283.1	Plasmid	NCBI_complete_genomes
Klebsiella sp.	CP026284.1	Plasmid	NCBI_complete_genomes
Klebsiella sp.	CP026393.1	Plasmid	NCBI_complete_genomes
Klebsiella sp.	CP026394.1	Plasmid	NCBI_complete_genomes
Klebsiella sp.	CP026395.1	Plasmid	NCBI_complete_genomes
Klebsiella sp.	CP026396.1	Plasmid	NCBI_complete_genomes
Klebsiella sp.	CP026397.1	Plasmid	NCBI_complete_genomes
Klebsiella sp.	CP026398.1	Plasmid	NCBI_complete_genomes
Klebsiella sp.	CP026496.1	Plasmid	NCBI_complete_genomes
Klebsiella sp.	CP026497.1	Plasmid	NCBI_complete_genomes
Klebsiella sp.	CP026582.2	Plasmid	NCBI_complete_genomes
Klebsiella sp.	CP026583.2	Plasmid	NCBI_complete_genomes
Klebsiella sp.	CP026584.1	Plasmid	NCBI_complete_genomes
Klebsiella sp.	CP026587.1	Plasmid	NCBI_complete_genomes
Klebsiella sp.	CP026588.1	Plasmid	NCBI_complete_genomes
Klebsiella sp.	CP026589.1	Plasmid	NCBI_complete_genomes
Klebsiella sp.	CP026590.1	Plasmid	NCBI_complete_genomes
Klebsiella sp.	CP026716.1	Plasmid	NCBI_complete_genomes
Klebsiella sp.	CP026717.1	Plasmid	NCBI_complete_genomes
Klebsiella sp.	CP026718.1	Plasmid	NCBI_complete_genomes
Klebsiella sp.	CP026752.1	Plasmid	NCBI_complete_genomes
Klebsiella sp.	CP026753.1	Plasmid	NCBI_complete_genomes
Klebsiella sp.	CP026757.1	Plasmid	NCBI_complete_genomes
Klebsiella sp.	CP026758.1	Plasmid	NCBI_complete_genomes
Klebsiella sp.	CP026759.1	Plasmid	NCBI_complete_genomes

Klebsiella sp.	CP026760.1	Plasmid	NCBI_complete_genomes
Klebsiella sp.	CP027063.2	Plasmid	NCBI_complete_genomes
Klebsiella sp.	CP027065.2	Plasmid	NCBI_complete_genomes
Klebsiella sp.	CP027066.2	Plasmid	NCBI_complete_genomes
Klebsiella sp.	CP027067.1	Plasmid	NCBI_complete_genomes
Klebsiella sp.	CP027147.1	Plasmid	NCBI_complete_genomes
Klebsiella sp.	CP027148.1	Plasmid	NCBI_complete_genomes
Klebsiella sp.	CP027149.1	Plasmid	NCBI_complete_genomes
Klebsiella sp.	CP027150.1	Plasmid	NCBI_complete_genomes
Klebsiella sp.	CP027151.1	Plasmid	NCBI_complete_genomes
Klebsiella sp.	CP027152.1	Plasmid	NCBI_complete_genomes
Klebsiella sp.	CP027153.1	Plasmid	NCBI_complete_genomes
Klebsiella sp.	CP027154.1	Plasmid	NCBI_complete_genomes
Klebsiella sp.	CP027155.1	Plasmid	NCBI_complete_genomes
Klebsiella sp.	CP027156.1	Plasmid	NCBI_complete_genomes
Klebsiella sp.	CP027157.1	Plasmid	NCBI_complete_genomes
Klebsiella sp.	CP027158.1	Plasmid	NCBI_complete_genomes
Klebsiella sp.	CP027159.1	Plasmid	NCBI_complete_genomes
Klebsiella sp.	CP027161.1	Plasmid	NCBI_complete_genomes
Klebsiella sp.	CP027162.1	Plasmid	NCBI_complete_genomes
Klebsiella sp.	CP027163.1	Plasmid	NCBI_complete_genomes
Klebsiella sp.	CP027164.1	Plasmid	NCBI_complete_genomes
Klebsiella sp.	CP027425.1	Plasmid	NCBI_complete_genomes
Klebsiella sp.	CP027603.1	Plasmid	NCBI_complete_genomes
Klebsiella sp.	CP027613.1	Plasmid	NCBI_complete_genomes
Klebsiella sp.	CP027614.1	Plasmid	NCBI_complete_genomes
Klebsiella sp.	CP027615.1	Plasmid	NCBI_complete_genomes
Klebsiella sp.	CP027616.1	Plasmid	NCBI_complete_genomes
Klebsiella sp.	CP027617.1	Plasmid	NCBI_complete_genomes
Klebsiella sp.	CP028177.1	Plasmid	NCBI_complete_genomes
Klebsiella sp.	CP028178.1	Plasmid	NCBI_complete_genomes
Klebsiella sp.	CP028179.1	Plasmid	NCBI_complete_genomes
Klebsiella sp.	CP028181.1	Plasmid	NCBI_complete_genomes
Klebsiella sp.	CP028182.1	Plasmid	NCBI_complete_genomes
Klebsiella sp.	CP028389.2	Plasmid	NCBI_complete_genomes
Klebsiella sp.	CP028390.1	Plasmid	NCBI_complete_genomes
Klebsiella sp.	CP028539.2	Plasmid	NCBI_complete_genomes
Klebsiella sp.	CP028540.2	Plasmid	NCBI_complete_genomes
Klebsiella sp.	CP028541.2	Plasmid	NCBI_complete_genomes
Klebsiella sp.	CP028543.1	Plasmid	NCBI_complete_genomes
Klebsiella sp.	CP028544.1	Plasmid	NCBI_complete_genomes
Klebsiella sp.	CP028545.1	Plasmid	NCBI_complete_genomes
Klebsiella sp.	CP028546.1	Plasmid	NCBI_complete_genomes
Klebsiella sp.	CP028547.1	Plasmid	NCBI_complete_genomes
Klebsiella sp.	CP028549.1	Plasmid	NCBI_complete_genomes
Klebsiella sp.	CP028550.1	Plasmid	NCBI_complete_genomes
Klebsiella sp.	CP028551.1	Plasmid	NCBI_complete_genomes
Klebsiella sp.	CP028552.1	Plasmid	NCBI_complete_genomes
Klebsiella sp.	CP028553.1	Plasmid	NCBI_complete_genomes
Klebsiella sp.	CP028554.1	Plasmid	NCBI_complete_genomes
Klebsiella sp.	CP028579.1	Plasmid	NCBI_complete_genomes
Klebsiella sp.	CP028580.1	Plasmid	NCBI_complete_genomes
Klebsiella sp.	CP028581.1	Plasmid	NCBI_complete_genomes
Klebsiella sp.	CP028582.1	Plasmid	NCBI_complete_genomes
Klebsiella sp.	CP028717.1	Plasmid	NCBI_complete_genomes
Klebsiella sp.	CP028718.1	Plasmid	NCBI_complete_genomes
Klebsiella sp.	CP028779.1	Plasmid	NCBI_complete_genomes
Klebsiella sp.	CP028780.1	Plasmid	NCBI_complete_genomes
Klebsiella sp.	CP028781.1	Plasmid	NCBI_complete_genomes
Klebsiella sp.	CP028782.1	Plasmid	NCBI_complete_genomes
Klebsiella sp.	CP028784.1	Plasmid	NCBI_complete_genomes
Klebsiella sp.	CP028785.1	Plasmid	NCBI_complete_genomes
Klebsiella sp.	CP028786.1	Plasmid	NCBI_complete_genomes
Klebsiella sp.	CP028788.1	Plasmid	NCBI_complete_genomes
Klebsiella sp.	CP028789.1	Plasmid	NCBI_complete_genomes
Klebsiella sp.	CP028790.1	Plasmid	NCBI_complete_genomes
Klebsiella sp.	CP028791.1	Plasmid	NCBI_complete_genomes
Klebsiella sp.	CP028792.1	Plasmid	NCBI_complete_genomes
Klebsiella sp.	CP028794.1	Plasmid	NCBI_complete_genomes
Klebsiella sp.	CP028795.1	Plasmid	NCBI_complete_genomes
Klebsiella sp.	CP028796.1	Plasmid	NCBI_complete_genomes
Klebsiella sp.	CP028801.1	Plasmid	NCBI_complete_genomes

Klebsiella sp.	CP028802.1	Plasmid	NCBI_complete_genomes
Klebsiella sp.	CP028803.1	Plasmid	NCBI_complete_genomes
Klebsiella sp.	CP028804.1	Plasmid	NCBI_complete_genomes
Klebsiella sp.	CP028805.1	Plasmid	NCBI_complete_genomes
Klebsiella sp.	CP028817.1	Plasmid	NCBI_complete_genomes
Klebsiella sp.	CP028818.1	Plasmid	NCBI_complete_genomes
Klebsiella sp.	CP028819.1	Plasmid	NCBI_complete_genomes
Klebsiella sp.	CP028820.1	Plasmid	NCBI_complete_genomes
Klebsiella sp.	CP028821.1	Plasmid	NCBI_complete_genomes
Klebsiella sp.	CP028822.1	Plasmid	NCBI_complete_genomes
Klebsiella sp.	CP028823.1	Plasmid	NCBI_complete_genomes
Klebsiella sp.	CP028824.1	Plasmid	NCBI_complete_genomes
Klebsiella sp.	CP028825.1	Plasmid	NCBI_complete_genomes
Klebsiella sp.	CP028916.1	Plasmid	NCBI_complete_genomes
Klebsiella sp.	CP028929.1	Plasmid	NCBI_complete_genomes
Klebsiella sp.	CP028930.1	Plasmid	NCBI_complete_genomes
Klebsiella sp.	CP028931.1	Plasmid	NCBI_complete_genomes
Klebsiella sp.	CP028952.1	Plasmid	NCBI_complete_genomes
Klebsiella sp.	CP028954.1	Plasmid	NCBI_complete_genomes
Klebsiella sp.	CP028955.1	Plasmid	NCBI_complete_genomes
Klebsiella sp.	CP028991.1	Plasmid	NCBI_complete_genomes
Klebsiella sp.	CP028992.1	Plasmid	NCBI_complete_genomes
Klebsiella sp.	CP028993.1	Plasmid	NCBI_complete_genomes
Klebsiella sp.	CP028995.1	Plasmid	NCBI_complete_genomes
Klebsiella sp.	CP028996.1	Plasmid	NCBI_complete_genomes
Klebsiella sp.	CP028997.1	Plasmid	NCBI_complete_genomes
Klebsiella sp.	CP028998.1	Plasmid	NCBI_complete_genomes
Klebsiella sp.	CP028999.1	Plasmid	NCBI_complete_genomes
Klebsiella sp.	CP029000.1	Plasmid	NCBI_complete_genomes
Klebsiella sp.	CP029098.1	Plasmid	NCBI_complete_genomes
Klebsiella sp.	CP029100.1	Plasmid	NCBI_complete_genomes
Klebsiella sp.	CP029101.1	Plasmid	NCBI_complete_genomes
Klebsiella sp.	CP029102.1	Plasmid	NCBI_complete_genomes
Klebsiella sp.	CP029127.1	Plasmid	NCBI_complete_genomes
Klebsiella sp.	CP029135.1	Plasmid	NCBI_complete_genomes
Klebsiella sp.	CP029136.1	Plasmid	NCBI_complete_genomes
Klebsiella sp.	CP029138.1	Plasmid	NCBI_complete_genomes
Klebsiella sp.	CP029139.1	Plasmid	NCBI_complete_genomes
Klebsiella sp.	CP029140.1	Plasmid	NCBI_complete_genomes
Klebsiella sp.	CP029142.1	Plasmid	NCBI_complete_genomes
Klebsiella sp.	CP029143.1	Plasmid	NCBI_complete_genomes
Klebsiella sp.	CP029144.1	Plasmid	NCBI_complete_genomes
Klebsiella sp.	CP029217.1	Plasmid	NCBI_complete_genomes
Klebsiella sp.	CP029218.1	Plasmid	NCBI_complete_genomes
Klebsiella sp.	CP029219.1	Plasmid	NCBI_complete_genomes
Klebsiella sp.	CP029221.1	Plasmid	NCBI_complete_genomes
Klebsiella sp.	CP029222.1	Plasmid	NCBI_complete_genomes
Klebsiella sp.	CP029223.1	Plasmid	NCBI_complete_genomes
Klebsiella sp.	CP029224.1	Plasmid	NCBI_complete_genomes
Klebsiella sp.	CP029225.1	Plasmid	NCBI_complete_genomes
Klebsiella sp.	CP029227.1	Plasmid	NCBI_complete_genomes
Klebsiella sp.	CP029228.1	Plasmid	NCBI_complete_genomes
Klebsiella sp.	CP029229.1	Plasmid	NCBI_complete_genomes
Klebsiella sp.	CP029230.1	Plasmid	NCBI_complete_genomes
Klebsiella sp.	CP029379.1	Plasmid	NCBI_complete_genomes
Klebsiella sp.	CP029380.1	Plasmid	NCBI_complete_genomes
Klebsiella sp.	CP029381.1	Plasmid	NCBI_complete_genomes
Klebsiella sp.	CP029382.1	Plasmid	NCBI_complete_genomes
Klebsiella sp.	CP029383.1	Plasmid	NCBI_complete_genomes
Klebsiella sp.	CP029385.1	Plasmid	NCBI_complete_genomes
Klebsiella sp.	CP029386.1	Plasmid	NCBI_complete_genomes
Klebsiella sp.	CP029387.1	Plasmid	NCBI_complete_genomes
Klebsiella sp.	CP029428.1	Plasmid	NCBI_complete_genomes
Klebsiella sp.	CP029429.1	Plasmid	NCBI_complete_genomes
Klebsiella sp.	CP029430.1	Plasmid	NCBI_complete_genomes
Klebsiella sp.	CP029431.1	Plasmid	NCBI_complete_genomes
Klebsiella sp.	CP029433.1	Plasmid	NCBI_complete_genomes
Klebsiella sp.	CP029434.1	Plasmid	NCBI_complete_genomes
Klebsiella sp.	CP029435.1	Plasmid	NCBI_complete_genomes
Klebsiella sp.	CP029436.1	Plasmid	NCBI_complete_genomes
Klebsiella sp.	CP029438.1	Plasmid	NCBI_complete_genomes
Klebsiella sp.	CP029439.1	Plasmid	NCBI_complete_genomes

Klebsiella sp.	CP029440.1	Plasmid	NCBI_complete_genomes
Klebsiella sp.	CP029441.1	Plasmid	NCBI_complete_genomes
Klebsiella sp.	CP029442.1	Plasmid	NCBI_complete_genomes
Klebsiella sp.	CP029583.1	Plasmid	NCBI_complete_genomes
Klebsiella sp.	CP029584.1	Plasmid	NCBI_complete_genomes
Klebsiella sp.	CP029585.1	Plasmid	NCBI_complete_genomes
Klebsiella sp.	CP029586.1	Plasmid	NCBI_complete_genomes
Klebsiella sp.	CP029588.1	Plasmid	NCBI_complete_genomes
Klebsiella sp.	CP029589.1	Plasmid	NCBI_complete_genomes
Klebsiella sp.	CP029591.1	Plasmid	NCBI_complete_genomes
Klebsiella sp.	CP029592.1	Plasmid	NCBI_complete_genomes
Klebsiella sp.	CP029598.1	Plasmid	NCBI_complete_genomes
Klebsiella sp.	CP029599.1	Plasmid	NCBI_complete_genomes
Klebsiella sp.	CP029723.1	Plasmid	NCBI_complete_genomes
Klebsiella sp.	CP029724.1	Plasmid	NCBI_complete_genomes
Klebsiella sp.	CP029739.1	Plasmid	NCBI_complete_genomes
Klebsiella sp.	CP029740.1	Plasmid	NCBI_complete_genomes
Klebsiella sp.	CP029771.1	Plasmid	NCBI_complete_genomes
Klebsiella sp.	CP029779.1	Plasmid	NCBI_complete_genomes
Klebsiella sp.	FO203353.1	Plasmid	NCBI_complete_genomes
Klebsiella sp.	FO203354.1	Plasmid	NCBI_complete_genomes
Klebsiella sp.	FO203500.1	Plasmid	NCBI_complete_genomes
Klebsiella sp.	FO834904.1	Plasmid	NCBI_complete_genomes
Klebsiella sp.	FO834905.1	Plasmid	NCBI_complete_genomes
Klebsiella sp.	JQ314407.1	Plasmid	NCBI_complete_genomes
Klebsiella sp.	LN824134.1	Plasmid	NCBI_complete_genomes
Klebsiella sp.	LN824135.1	Plasmid	NCBI_complete_genomes
Klebsiella sp.	LN824136.1	Plasmid	NCBI_complete_genomes
Klebsiella sp.	LN824137.1	Plasmid	NCBI_complete_genomes
Klebsiella sp.	LN824138.1	Plasmid	NCBI_complete_genomes
Klebsiella sp.	LN824139.1	Plasmid	NCBI_complete_genomes
Klebsiella sp.	LT216437.1	Plasmid	NCBI_complete_genomes
Klebsiella sp.	LT216438.1	Plasmid	NCBI_complete_genomes
Klebsiella sp.	LT216439.1	Plasmid	NCBI_complete_genomes
Klebsiella sp.	LT216440.1	Plasmid	NCBI_complete_genomes
Klebsiella sp.	AB616660.2	Plasmid	Orlek_et_al.
Klebsiella sp.	AB715422.2	Plasmid	Orlek_et_al.
Klebsiella sp.	AE006471.2	Plasmid	Orlek_et_al.
Klebsiella sp.	AJGY01000060.1	Plasmid	Orlek_et_al.
Klebsiella sp.	AJGY01000090.1	Plasmid	Orlek_et_al.
Klebsiella sp.	AP014611.1	Plasmid	Orlek_et_al.
Klebsiella sp.	AP014654.2	Plasmid	Orlek_et_al.
Klebsiella sp.	AP014804.1	Plasmid	Orlek_et_al.
Klebsiella sp.	AP014805.1	Plasmid	Orlek_et_al.
Klebsiella sp.	AP014806.1	Plasmid	Orlek_et_al.
Klebsiella sp.	AP014807.1	Plasmid	Orlek_et_al.
Klebsiella sp.	AP014939.1	Plasmid	Orlek_et_al.
Klebsiella sp.	CM004293.1	Plasmid	Orlek_et_al.
Klebsiella sp.	CP002127.1	Plasmid	Orlek_et_al.
Klebsiella sp.	CP002128.1	Plasmid	Orlek_et_al.
Klebsiella sp.	CP006737.1	Plasmid	Orlek_et_al.
Klebsiella sp.	CP007449.1	Plasmid	Orlek_et_al.
Klebsiella sp.	CP007450.1	Plasmid	Orlek_et_al.
Klebsiella sp.	CP008701.1	Plasmid	Orlek_et_al.
Klebsiella sp.	CP009231.1	Plasmid	Orlek_et_al.
Klebsiella sp.	CP009232.1	Plasmid	Orlek_et_al.
Klebsiella sp.	CP009233.1	Plasmid	Orlek_et_al.
Klebsiella sp.	CP009410.2	Plasmid	Orlek_et_al.
Klebsiella sp.	CP009411.2	Plasmid	Orlek_et_al.
Klebsiella sp.	CP009412.2	Plasmid	Orlek_et_al.
Klebsiella sp.	CP009413.2	Plasmid	Orlek_et_al.
Klebsiella sp.	CP009414.2	Plasmid	Orlek_et_al.
Klebsiella sp.	CP010513.1	Plasmid	Orlek_et_al.
Klebsiella sp.	CP011658.1	Plasmid	Orlek_et_al.
Klebsiella sp.	CP011999.1	Plasmid	Orlek_et_al.
Klebsiella sp.	CP012000.1	Plasmid	Orlek_et_al.
Klebsiella sp.	CP012197.1	Plasmid	Orlek_et_al.
Klebsiella sp.	CP012198.1	Plasmid	Orlek_et_al.
Klebsiella sp.	CP012902.1	Plasmid	Orlek_et_al.
Klebsiella sp.	CP012903.1	Plasmid	Orlek_et_al.
Klebsiella sp.	CP012904.1	Plasmid	Orlek_et_al.
Klebsiella sp.	CP013215.1	Plasmid	Orlek_et_al.

Klebsiella sp.	CP013836.1	Plasmid	Orlek_et_al.
Klebsiella sp.	CP014776.1	Plasmid	Orlek_et_al.
Klebsiella sp.	CP014777.1	Plasmid	Orlek_et_al.
Klebsiella sp.	CP014778.1	Plasmid	Orlek_et_al.
Klebsiella sp.	CP014779.1	Plasmid	Orlek_et_al.
Klebsiella sp.	CP014962.1	Plasmid	Orlek_et_al.
Klebsiella sp.	CP015086.1	Plasmid	Orlek_et_al.
Klebsiella sp.	CP015977.1	Plasmid	Orlek_et_al.
Klebsiella sp.	CP016815.1	Plasmid	Orlek_et_al.
Klebsiella sp.	CP016864.1	Plasmid	Orlek_et_al.
Klebsiella sp.	CP016865.1	Plasmid	Orlek_et_al.
Klebsiella sp.	CP016866.1	Plasmid	Orlek_et_al.
Klebsiella sp.	CP016867.1	Plasmid	Orlek_et_al.
Klebsiella sp.	CP016890.1	Plasmid	Orlek_et_al.
Klebsiella sp.	CP016891.1	Plasmid	Orlek_et_al.
Klebsiella sp.	CP016892.1	Plasmid	Orlek_et_al.
Klebsiella sp.	CP016919.1	Plasmid	Orlek_et_al.
Klebsiella sp.	CP016920.1	Plasmid	Orlek_et_al.
Klebsiella sp.	CP016921.1	Plasmid	Orlek_et_al.
Klebsiella sp.	CP016922.1	Plasmid	Orlek_et_al.
Klebsiella sp.	CP016924.1	Plasmid	Orlek_et_al.
Klebsiella sp.	CP016925.1	Plasmid	Orlek_et_al.
Klebsiella sp.	CP016927.1	Plasmid	Orlek_et_al.
Klebsiella sp.	CP016932.1	Plasmid	Orlek_et_al.
Klebsiella sp.	CP016933.1	Plasmid	Orlek_et_al.
Klebsiella sp.	CP016934.1	Plasmid	Orlek_et_al.
Klebsiella sp.	CP016936.1	Plasmid	Orlek_et_al.
Klebsiella sp.	CP016938.1	Plasmid	Orlek_et_al.
Klebsiella sp.	CP016939.1	Plasmid	Orlek_et_al.
Klebsiella sp.	CP016941.1	Plasmid	Orlek_et_al.
Klebsiella sp.	CP016942.1	Plasmid	Orlek_et_al.
Klebsiella sp.	CP016943.1	Plasmid	Orlek_et_al.
Klebsiella sp.	CP016946.1	Plasmid	Orlek_et_al.
Klebsiella sp.	CP016947.1	Plasmid	Orlek_et_al.
Klebsiella sp.	CU928146.1	Plasmid	Orlek_et_al.
Klebsiella sp.	EF219134.3	Plasmid	Orlek_et_al.
Klebsiella sp.	EU418923.1	Plasmid	Orlek_et_al.
Klebsiella sp.	EU418926.1	Plasmid	Orlek_et_al.
Klebsiella sp.	EU418931.1	Plasmid	Orlek_et_al.
Klebsiella sp.	FN691998.1	Plasmid	Orlek_et_al.
Klebsiella sp.	HF560649.1	Plasmid	Orlek_et_al.
Klebsiella sp.	HF922624.1	Plasmid	Orlek_et_al.
Klebsiella sp.	HF969016.1	Plasmid	Orlek_et_al.
Klebsiella sp.	HG813238.1	Plasmid	Orlek_et_al.
Klebsiella sp.	HG813239.1	Plasmid	Orlek_et_al.
Klebsiella sp.	HG918041.1	Plasmid	Orlek_et_al.
Klebsiella sp.	HG969995.1	Plasmid	Orlek_et_al.
Klebsiella sp.	HG969996.1	Plasmid	Orlek_et_al.
Klebsiella sp.	HG969997.1	Plasmid	Orlek_et_al.
Klebsiella sp.	HG969998.1	Plasmid	Orlek_et_al.
Klebsiella sp.	HG969999.1	Plasmid	Orlek_et_al.
Klebsiella sp.	HG970000.1	Plasmid	Orlek_et_al.
Klebsiella sp.	HG970001.1	Plasmid	Orlek_et_al.
Klebsiella sp.	HM138194.1	Plasmid	Orlek_et_al.
Klebsiella sp.	JMMY01000002.1	Plasmid	Orlek_et_al.
Klebsiella sp.	JMSW01000002.1	Plasmid	Orlek_et_al.
Klebsiella sp.	JMSW01000004.1	Plasmid	Orlek_et_al.
Klebsiella sp.	JMSX01000002.1	Plasmid	Orlek_et_al.
Klebsiella sp.	JMSX01000003.1	Plasmid	Orlek_et_al.
Klebsiella sp.	JMSX01000004.1	Plasmid	Orlek_et_al.
Klebsiella sp.	JMSX01000005.1	Plasmid	Orlek_et_al.
Klebsiella sp.	JN194214.1	Plasmid	Orlek_et_al.
Klebsiella sp.	JN233704.1	Plasmid	Orlek_et_al.
Klebsiella sp.	JNBM01000002.1	Plasmid	Orlek_et_al.
Klebsiella sp.	JNBM01000003.1	Plasmid	Orlek_et_al.
Klebsiella sp.	JNBM01000005.1	Plasmid	Orlek_et_al.
Klebsiella sp.	JX065631.1	Plasmid	Orlek_et_al.
Klebsiella sp.	KC887916.2	Plasmid	Orlek_et_al.
Klebsiella sp.	KC887917.2	Plasmid	Orlek_et_al.
Klebsiella sp.	KC999035.4	Plasmid	Orlek_et_al.
Klebsiella sp.	KF601686.2	Plasmid	Orlek_et_al.
Klebsiella sp.	KF743817.1	Plasmid	Orlek_et_al.

Klebsiella sp.	KF992018.2	Plasmid	Orlek_et_al.
Klebsiella sp.	KJ020575.1	Plasmid	Orlek_et_al.
Klebsiella sp.	KJ020576.1	Plasmid	Orlek_et_al.
Klebsiella sp.	KJ158441.1	Plasmid	Orlek_et_al.
Klebsiella sp.	KJ187750.1	Plasmid	Orlek_et_al.
Klebsiella sp.	KJ187751.1	Plasmid	Orlek_et_al.
Klebsiella sp.	KJ187752.1	Plasmid	Orlek_et_al.
Klebsiella sp.	KJ190020.1	Plasmid	Orlek_et_al.
Klebsiella sp.	KJ201886.1	Plasmid	Orlek_et_al.
Klebsiella sp.	KJ201887.1	Plasmid	Orlek_et_al.
Klebsiella sp.	KJ406378.1	Plasmid	Orlek_et_al.
Klebsiella sp.	KJ440075.1	Plasmid	Orlek_et_al.
Klebsiella sp.	KJ440076.1	Plasmid	Orlek_et_al.
Klebsiella sp.	KJ460501.1	Plasmid	Orlek_et_al.
Klebsiella sp.	KJ541068.1	Plasmid	Orlek_et_al.
Klebsiella sp.	KJ541069.1	Plasmid	Orlek_et_al.
Klebsiella sp.	KJ541070.1	Plasmid	Orlek_et_al.
Klebsiella sp.	KJ541071.1	Plasmid	Orlek_et_al.
Klebsiella sp.	KJ563250.1	Plasmid	Orlek_et_al.
Klebsiella sp.	KJ577613.1	Plasmid	Orlek_et_al.
Klebsiella sp.	KJ588779.1	Plasmid	Orlek_et_al.
Klebsiella sp.	KJ653815.1	Plasmid	Orlek_et_al.
Klebsiella sp.	KJ721789.1	Plasmid	Orlek_et_al.
Klebsiella sp.	KJ721790.1	Plasmid	Orlek_et_al.
Klebsiella sp.	KJ721805.1	Plasmid	Orlek_et_al.
Klebsiella sp.	KJ802404.1	Plasmid	Orlek_et_al.
Klebsiella sp.	KJ802405.1	Plasmid	Orlek_et_al.
Klebsiella sp.	KJ812998.1	Plasmid	Orlek_et_al.
Klebsiella sp.	KJ866866.1	Plasmid	Orlek_et_al.
Klebsiella sp.	KJ958926.1	Plasmid	Orlek_et_al.
Klebsiella sp.	KJ958927.1	Plasmid	Orlek_et_al.
Klebsiella sp.	KM023153.1	Plasmid	Orlek_et_al.
Klebsiella sp.	KM052220.1	Plasmid	Orlek_et_al.
Klebsiella sp.	KM085449.1	Plasmid	Orlek_et_al.
Klebsiella sp.	KM085450.1	Plasmid	Orlek_et_al.
Klebsiella sp.	KM085451.1	Plasmid	Orlek_et_al.
Klebsiella sp.	KM085452.1	Plasmid	Orlek_et_al.
Klebsiella sp.	KM085453.1	Plasmid	Orlek_et_al.
Klebsiella sp.	KM107838.1	Plasmid	Orlek_et_al.
Klebsiella sp.	KM107839.1	Plasmid	Orlek_et_al.
Klebsiella sp.	KM107840.1	Plasmid	Orlek_et_al.
Klebsiella sp.	KM107841.1	Plasmid	Orlek_et_al.
Klebsiella sp.	KM107842.1	Plasmid	Orlek_et_al.
Klebsiella sp.	KM107843.1	Plasmid	Orlek_et_al.
Klebsiella sp.	KM107844.1	Plasmid	Orlek_et_al.
Klebsiella sp.	KM107845.1	Plasmid	Orlek_et_al.
Klebsiella sp.	KM107846.1	Plasmid	Orlek_et_al.
Klebsiella sp.	KM107847.1	Plasmid	Orlek_et_al.
Klebsiella sp.	KM107848.1	Plasmid	Orlek_et_al.
Klebsiella sp.	KM112087.1	Plasmid	Orlek_et_al.
Klebsiella sp.	KM198330.1	Plasmid	Orlek_et_al.
Klebsiella sp.	KM207012.2	Plasmid	Orlek_et_al.
Klebsiella sp.	KM212169.1	Plasmid	Orlek_et_al.
Klebsiella sp.	KM287568.1	Plasmid	Orlek_et_al.
Klebsiella sp.	KM373703.1	Plasmid	Orlek_et_al.
Klebsiella sp.	KM377238.1	Plasmid	Orlek_et_al.
Klebsiella sp.	KM377239.1	Plasmid	Orlek_et_al.
Klebsiella sp.	KM377240.1	Plasmid	Orlek_et_al.
Klebsiella sp.	KM396298.1	Plasmid	Orlek_et_al.
Klebsiella sp.	KM396299.1	Plasmid	Orlek_et_al.
Klebsiella sp.	KM396300.1	Plasmid	Orlek_et_al.
Klebsiella sp.	KM400601.1	Plasmid	Orlek_et_al.
Klebsiella sp.	KM406488.1	Plasmid	Orlek_et_al.
Klebsiella sp.	KM406489.1	Plasmid	Orlek_et_al.
Klebsiella sp.	KM406490.1	Plasmid	Orlek_et_al.
Klebsiella sp.	KM406491.1	Plasmid	Orlek_et_al.
Klebsiella sp.	KM409652.1	Plasmid	Orlek_et_al.
Klebsiella sp.	KM577619.1	Plasmid	Orlek_et_al.
Klebsiella sp.	KM580532.1	Plasmid	Orlek_et_al.
Klebsiella sp.	KM580533.1	Plasmid	Orlek_et_al.
Klebsiella sp.	KM660724.1	Plasmid	Orlek_et_al.
Klebsiella sp.	KM670336.1	Plasmid	Orlek_et_al.

Klebsiella sp.	KM877269.1	Plasmid	Orlek_et_al.
Klebsiella sp.	KM877517.1	Plasmid	Orlek_et_al.
Klebsiella sp.	KM977631.1	Plasmid	Orlek_et_al.
Klebsiella sp.	KP008371.1	Plasmid	Orlek_et_al.
Klebsiella sp.	KP017243.1	Plasmid	Orlek_et_al.
Klebsiella sp.	KP025948.1	Plasmid	Orlek_et_al.
Klebsiella sp.	KP056256.1	Plasmid	Orlek_et_al.
Klebsiella sp.	KP061858.1	Plasmid	Orlek_et_al.
Klebsiella sp.	KP091735.1	Plasmid	Orlek_et_al.
Klebsiella sp.	KP125892.1	Plasmid	Orlek_et_al.
Klebsiella sp.	KP125893.1	Plasmid	Orlek_et_al.
Klebsiella sp.	KP143090.1	Plasmid	Orlek_et_al.
Klebsiella sp.	KP198615.1	Plasmid	Orlek_et_al.
Klebsiella sp.	KP198616.1	Plasmid	Orlek_et_al.
Klebsiella sp.	KP205272.1	Plasmid	Orlek_et_al.
Klebsiella sp.	KP276584.1	Plasmid	Orlek_et_al.
Klebsiella sp.	KP294351.1	Plasmid	Orlek_et_al.
Klebsiella sp.	KP313759.1	Plasmid	Orlek_et_al.
Klebsiella sp.	KP324830.1	Plasmid	Orlek_et_al.
Klebsiella sp.	KP330456.1	Plasmid	Orlek_et_al.
Klebsiella sp.	KP345882.1	Plasmid	Orlek_et_al.
Klebsiella sp.	KP347127.1	Plasmid	Orlek_et_al.
Klebsiella sp.	KP398867.1	Plasmid	Orlek_et_al.
Klebsiella sp.	KP400525.1	Plasmid	Orlek_et_al.
Klebsiella sp.	KP453775.1	Plasmid	Orlek_et_al.
Klebsiella sp.	KP659188.1	Plasmid	Orlek_et_al.
Klebsiella sp.	KP689347.1	Plasmid	Orlek_et_al.
Klebsiella sp.	KP726894.1	Plasmid	Orlek_et_al.
Klebsiella sp.	KP742988.1	Plasmid	Orlek_et_al.
Klebsiella sp.	KP763470.1	Plasmid	Orlek_et_al.
Klebsiella sp.	KP765744.1	Plasmid	Orlek_et_al.
Klebsiella sp.	KP776609.1	Plasmid	Orlek_et_al.
Klebsiella sp.	KP789019.1	Plasmid	Orlek_et_al.
Klebsiella sp.	KP789020.1	Plasmid	Orlek_et_al.
Klebsiella sp.	KP792123.1	Plasmid	Orlek_et_al.
Klebsiella sp.	KP868646.1	Plasmid	Orlek_et_al.
Klebsiella sp.	KP868647.1	Plasmid	Orlek_et_al.
Klebsiella sp.	KP893385.1	Plasmid	Orlek_et_al.
Klebsiella sp.	KP899803.1	Plasmid	Orlek_et_al.
Klebsiella sp.	KP899804.1	Plasmid	Orlek_et_al.
Klebsiella sp.	KP899805.1	Plasmid	Orlek_et_al.
Klebsiella sp.	KP899806.1	Plasmid	Orlek_et_al.
Klebsiella sp.	KP900015.1	Plasmid	Orlek_et_al.
Klebsiella sp.	KP900016.1	Plasmid	Orlek_et_al.
Klebsiella sp.	KP942676.1	Plasmid	Orlek_et_al.
Klebsiella sp.	KP970685.1	Plasmid	Orlek_et_al.
Klebsiella sp.	KP975074.1	Plasmid	Orlek_et_al.
Klebsiella sp.	KP975075.1	Plasmid	Orlek_et_al.
Klebsiella sp.	KP975077.1	Plasmid	Orlek_et_al.
Klebsiella sp.	KP979588.1	Plasmid	Orlek_et_al.
Klebsiella sp.	KP979589.1	Plasmid	Orlek_et_al.
Klebsiella sp.	KP987215.1	Plasmid	Orlek_et_al.
Klebsiella sp.	KP987216.1	Plasmid	Orlek_et_al.
Klebsiella sp.	KP987217.1	Plasmid	Orlek_et_al.
Klebsiella sp.	KP987218.1	Plasmid	Orlek_et_al.
Klebsiella sp.	KR059864.1	Plasmid	Orlek_et_al.
Klebsiella sp.	KR078259.1	Plasmid	Orlek_et_al.
Klebsiella sp.	KR091911.1	Plasmid	Orlek_et_al.
Klebsiella sp.	KR091915.1	Plasmid	Orlek_et_al.
Klebsiella sp.	KR259130.1	Plasmid	Orlek_et_al.
Klebsiella sp.	KR259131.1	Plasmid	Orlek_et_al.
Klebsiella sp.	KR259132.1	Plasmid	Orlek_et_al.
Klebsiella sp.	KR259133.1	Plasmid	Orlek_et_al.
Klebsiella sp.	KR259134.1	Plasmid	Orlek_et_al.
Klebsiella sp.	KR351290.1	Plasmid	Orlek_et_al.
Klebsiella sp.	KR559888.1	Plasmid	Orlek_et_al.
Klebsiella sp.	KR559889.1	Plasmid	Orlek_et_al.
Klebsiella sp.	KR559890.1	Plasmid	Orlek_et_al.
Klebsiella sp.	KR653209.1	Plasmid	Orlek_et_al.
Klebsiella sp.	KR779901.1	Plasmid	Orlek_et_al.
Klebsiella sp.	KR822246.1	Plasmid	Orlek_et_al.
Klebsiella sp.	KR822247.1	Plasmid	Orlek_et_al.

Klebsiella sp.	KR827684.1	Plasmid	Orlek_et_al.
Klebsiella sp.	KR869777.1	Plasmid	Orlek_et_al.
Klebsiella sp.	KR905384.1	Plasmid	Orlek_et_al.
Klebsiella sp.	KR905385.1	Plasmid	Orlek_et_al.
Klebsiella sp.	KR905386.1	Plasmid	Orlek_et_al.
Klebsiella sp.	KR905387.1	Plasmid	Orlek_et_al.
Klebsiella sp.	KR905388.1	Plasmid	Orlek_et_al.
Klebsiella sp.	KR905389.1	Plasmid	Orlek_et_al.
Klebsiella sp.	KR905390.1	Plasmid	Orlek_et_al.
Klebsiella sp.	KT002541.1	Plasmid	Orlek_et_al.
Klebsiella sp.	KT005457.1	Plasmid	Orlek_et_al.
Klebsiella sp.	KT020860.1	Plasmid	Orlek_et_al.
Klebsiella sp.	KT070138.1	Plasmid	Orlek_et_al.
Klebsiella sp.	KT074362.1	Plasmid	Orlek_et_al.
Klebsiella sp.	KT148595.1	Plasmid	Orlek_et_al.
Klebsiella sp.	KT185451.1	Plasmid	Orlek_et_al.
Klebsiella sp.	KT203286.1	Plasmid	Orlek_et_al.
Klebsiella sp.	KT207463.1	Plasmid	Orlek_et_al.
Klebsiella sp.	KT225462.1	Plasmid	Orlek_et_al.
Klebsiella sp.	KT225520.1	Plasmid	Orlek_et_al.
Klebsiella sp.	KT282968.1	Plasmid	Orlek_et_al.
Klebsiella sp.	KT307967.1	Plasmid	Orlek_et_al.
Klebsiella sp.	KT317611.1	Plasmid	Orlek_et_al.
Klebsiella sp.	KT317612.1	Plasmid	Orlek_et_al.
Klebsiella sp.	KT317613.1	Plasmid	Orlek_et_al.
Klebsiella sp.	KT317614.1	Plasmid	Orlek_et_al.
Klebsiella sp.	KT334335.1	Plasmid	Orlek_et_al.
Klebsiella sp.	KT345945.1	Plasmid	Orlek_et_al.
Klebsiella sp.	KT345946.1	Plasmid	Orlek_et_al.
Klebsiella sp.	KT345947.1	Plasmid	Orlek_et_al.
Klebsiella sp.	KT347600.1	Plasmid	Orlek_et_al.
Klebsiella sp.	KT351734.1	Plasmid	Orlek_et_al.
Klebsiella sp.	KT351735.1	Plasmid	Orlek_et_al.
Klebsiella sp.	KT351736.1	Plasmid	Orlek_et_al.
Klebsiella sp.	KT351737.1	Plasmid	Orlek_et_al.
Klebsiella sp.	KT362706.1	Plasmid	Orlek_et_al.
Klebsiella sp.	KT693143.1	Plasmid	Orlek_et_al.
Klebsiella sp.	KT693144.1	Plasmid	Orlek_et_al.
Klebsiella sp.	KT693145.1	Plasmid	Orlek_et_al.
Klebsiella sp.	KT725788.1	Plasmid	Orlek_et_al.
Klebsiella sp.	KT725789.1	Plasmid	Orlek_et_al.
Klebsiella sp.	KT754160.1	Plasmid	Orlek_et_al.
Klebsiella sp.	KT754161.1	Plasmid	Orlek_et_al.
Klebsiella sp.	KT754162.1	Plasmid	Orlek_et_al.
Klebsiella sp.	KT754163.1	Plasmid	Orlek_et_al.
Klebsiella sp.	KT754164.1	Plasmid	Orlek_et_al.
Klebsiella sp.	KT754165.1	Plasmid	Orlek_et_al.
Klebsiella sp.	KT754166.1	Plasmid	Orlek_et_al.
Klebsiella sp.	KT754167.1	Plasmid	Orlek_et_al.
Klebsiella sp.	KT779550.1	Plasmid	Orlek_et_al.
Klebsiella sp.	KT818627.1	Plasmid	Orlek_et_al.
Klebsiella sp.	KT824791.1	Plasmid	Orlek_et_al.
Klebsiella sp.	KT868530.1	Plasmid	Orlek_et_al.
Klebsiella sp.	KT879914.1	Plasmid	Orlek_et_al.
Klebsiella sp.	KT896499.1	Plasmid	Orlek_et_al.
Klebsiella sp.	KT896500.1	Plasmid	Orlek_et_al.
Klebsiella sp.	KT896501.1	Plasmid	Orlek_et_al.
Klebsiella sp.	KT896502.1	Plasmid	Orlek_et_al.
Klebsiella sp.	KT896503.1	Plasmid	Orlek_et_al.
Klebsiella sp.	KT935445.1	Plasmid	Orlek_et_al.
Klebsiella sp.	KT935446.1	Plasmid	Orlek_et_al.
Klebsiella sp.	KT937280.1	Plasmid	Orlek_et_al.
Klebsiella sp.	KT937281.1	Plasmid	Orlek_et_al.
Klebsiella sp.	KT937282.1	Plasmid	Orlek_et_al.
Klebsiella sp.	KT950740.1	Plasmid	Orlek_et_al.
Klebsiella sp.	KT950741.1	Plasmid	Orlek_et_al.
Klebsiella sp.	KT982613.1	Plasmid	Orlek_et_al.
Klebsiella sp.	KT982614.1	Plasmid	Orlek_et_al.
Klebsiella sp.	KT982616.1	Plasmid	Orlek_et_al.
Klebsiella sp.	KT982617.1	Plasmid	Orlek_et_al.
Klebsiella sp.	KT982618.1	Plasmid	Orlek_et_al.
Klebsiella sp.	KT988018.1	Plasmid	Orlek_et_al.

Klebsiella sp.	KT988019.1	Plasmid	Orlek_et_al.
Klebsiella sp.	KT988020.1	Plasmid	Orlek_et_al.
Klebsiella sp.	KT988306.1	Plasmid	Orlek_et_al.
Klebsiella sp.	KT989598.1	Plasmid	Orlek_et_al.
Klebsiella sp.	KT989599.1	Plasmid	Orlek_et_al.
Klebsiella sp.	KT990220.1	Plasmid	Orlek_et_al.
Klebsiella sp.	KU051707.1	Plasmid	Orlek_et_al.
Klebsiella sp.	KU051708.1	Plasmid	Orlek_et_al.
Klebsiella sp.	KU051709.1	Plasmid	Orlek_et_al.
Klebsiella sp.	KU051710.1	Plasmid	Orlek_et_al.
Klebsiella sp.	KU130396.1	Plasmid	Orlek_et_al.
Klebsiella sp.	KU159085.1	Plasmid	Orlek_et_al.
Klebsiella sp.	KU159086.1	Plasmid	Orlek_et_al.
Klebsiella sp.	KU160530.1	Plasmid	Orlek_et_al.
Klebsiella sp.	KU166868.1	Plasmid	Orlek_et_al.
Klebsiella sp.	KU176944.1	Plasmid	Orlek_et_al.
Klebsiella sp.	KU254578.1	Plasmid	Orlek_et_al.
Klebsiella sp.	KU254579.1	Plasmid	Orlek_et_al.
Klebsiella sp.	KU254580.1	Plasmid	Orlek_et_al.
Klebsiella sp.	KU254581.1	Plasmid	Orlek_et_al.
Klebsiella sp.	KU288634.1	Plasmid	Orlek_et_al.
Klebsiella sp.	KU295131.1	Plasmid	Orlek_et_al.
Klebsiella sp.	KU295132.1	Plasmid	Orlek_et_al.
Klebsiella sp.	KU295133.1	Plasmid	Orlek_et_al.
Klebsiella sp.	KU295134.1	Plasmid	Orlek_et_al.
Klebsiella sp.	KU295135.1	Plasmid	Orlek_et_al.
Klebsiella sp.	KU295136.1	Plasmid	Orlek_et_al.
Klebsiella sp.	KU302800.1	Plasmid	Orlek_et_al.
Klebsiella sp.	KU302801.1	Plasmid	Orlek_et_al.
Klebsiella sp.	KU302802.1	Plasmid	Orlek_et_al.
Klebsiella sp.	KU302803.1	Plasmid	Orlek_et_al.
Klebsiella sp.	KU302804.1	Plasmid	Orlek_et_al.
Klebsiella sp.	KU302805.1	Plasmid	Orlek_et_al.
Klebsiella sp.	KU302806.1	Plasmid	Orlek_et_al.
Klebsiella sp.	KU302807.1	Plasmid	Orlek_et_al.
Klebsiella sp.	KU302808.1	Plasmid	Orlek_et_al.
Klebsiella sp.	KU302809.1	Plasmid	Orlek_et_al.
Klebsiella sp.	KU312044.1	Plasmid	Orlek_et_al.
Klebsiella sp.	KU314941.1	Plasmid	Orlek_et_al.
Klebsiella sp.	KU315015.1	Plasmid	Orlek_et_al.
Klebsiella sp.	KU318419.1	Plasmid	Orlek_et_al.
Klebsiella sp.	KU318420.1	Plasmid	Orlek_et_al.
Klebsiella sp.	KU318421.1	Plasmid	Orlek_et_al.
Klebsiella sp.	KU341381.1	Plasmid	Orlek_et_al.
Klebsiella sp.	KU353730.1	Plasmid	Orlek_et_al.
Klebsiella sp.	KU355873.1	Plasmid	Orlek_et_al.
Klebsiella sp.	KU355874.1	Plasmid	Orlek_et_al.
Klebsiella sp.	KU665641.1	Plasmid	Orlek_et_al.
Klebsiella sp.	KU665642.1	Plasmid	Orlek_et_al.
Klebsiella sp.	KU674895.1	Plasmid	Orlek_et_al.
Klebsiella sp.	KU726092.1	Plasmid	Orlek_et_al.
Klebsiella sp.	KU726616.1	Plasmid	Orlek_et_al.
Klebsiella sp.	KU743383.1	Plasmid	Orlek_et_al.
Klebsiella sp.	KU743384.1	Plasmid	Orlek_et_al.
Klebsiella sp.	KU761326.1	Plasmid	Orlek_et_al.
Klebsiella sp.	KU761327.1	Plasmid	Orlek_et_al.
Klebsiella sp.	KU761328.1	Plasmid	Orlek_et_al.
Klebsiella sp.	KU870627.1	Plasmid	Orlek_et_al.
Klebsiella sp.	KU886034.1	Plasmid	Orlek_et_al.
Klebsiella sp.	KU922754.1	Plasmid	Orlek_et_al.
Klebsiella sp.	KU932021.1	Plasmid	Orlek_et_al.
Klebsiella sp.	KU932022.1	Plasmid	Orlek_et_al.
Klebsiella sp.	KU932023.1	Plasmid	Orlek_et_al.
Klebsiella sp.	KU932024.1	Plasmid	Orlek_et_al.
Klebsiella sp.	KU932025.1	Plasmid	Orlek_et_al.
Klebsiella sp.	KU932026.1	Plasmid	Orlek_et_al.
Klebsiella sp.	KU932027.1	Plasmid	Orlek_et_al.
Klebsiella sp.	KU932028.1	Plasmid	Orlek_et_al.
Klebsiella sp.	KU932029.1	Plasmid	Orlek_et_al.
Klebsiella sp.	KU932030.1	Plasmid	Orlek_et_al.
Klebsiella sp.	KU932031.1	Plasmid	Orlek_et_al.
Klebsiella sp.	KU932032.1	Plasmid	Orlek_et_al.

Klebsiella sp.	KU932033.1	Plasmid	Orlek_et_al.
Klebsiella sp.	KU932034.1	Plasmid	Orlek_et_al.
Klebsiella sp.	KU934011.1	Plasmid	Orlek_et_al.
Klebsiella sp.	KU934209.1	Plasmid	Orlek_et_al.
Klebsiella sp.	KU963389.1	Plasmid	Orlek_et_al.
Klebsiella sp.	KU963390.1	Plasmid	Orlek_et_al.
Klebsiella sp.	KU980950.1	Plasmid	Orlek_et_al.
Klebsiella sp.	KU987452.1	Plasmid	Orlek_et_al.
Klebsiella sp.	KU987453.1	Plasmid	Orlek_et_al.
Klebsiella sp.	KU994859.1	Plasmid	Orlek_et_al.
Klebsiella sp.	KU997026.1	Plasmid	Orlek_et_al.
Klebsiella sp.	KX009507.1	Plasmid	Orlek_et_al.
Klebsiella sp.	KX013538.1	Plasmid	Orlek_et_al.
Klebsiella sp.	KX013539.1	Plasmid	Orlek_et_al.
Klebsiella sp.	KX013540.1	Plasmid	Orlek_et_al.
Klebsiella sp.	KX015668.1	Plasmid	Orlek_et_al.
Klebsiella sp.	KX032519.1	Plasmid	Orlek_et_al.
Klebsiella sp.	KX032520.1	Plasmid	Orlek_et_al.
Klebsiella sp.	KX034083.1	Plasmid	Orlek_et_al.
Klebsiella sp.	KX084392.1	Plasmid	Orlek_et_al.
Klebsiella sp.	KX084393.1	Plasmid	Orlek_et_al.
Klebsiella sp.	KX084394.1	Plasmid	Orlek_et_al.
Klebsiella sp.	KX118608.1	Plasmid	Orlek_et_al.
Klebsiella sp.	KX129782.1	Plasmid	Orlek_et_al.
Klebsiella sp.	KX129783.1	Plasmid	Orlek_et_al.
Klebsiella sp.	KX129784.1	Plasmid	Orlek_et_al.
Klebsiella sp.	KX156772.1	Plasmid	Orlek_et_al.
Klebsiella sp.	KX156773.1	Plasmid	Orlek_et_al.
Klebsiella sp.	KX230795.1	Plasmid	Orlek_et_al.
Klebsiella sp.	KX236178.1	Plasmid	Orlek_et_al.
Klebsiella sp.	KX236309.1	Plasmid	Orlek_et_al.
Klebsiella sp.	KX244760.1	Plasmid	Orlek_et_al.
Klebsiella sp.	KX276657.1	Plasmid	Orlek_et_al.
Klebsiella sp.	KX443694.1	Plasmid	Orlek_et_al.
Klebsiella sp.	KX447767.1	Plasmid	Orlek_et_al.
Klebsiella sp.	KX447768.1	Plasmid	Orlek_et_al.
Klebsiella sp.	KX528699.1	Plasmid	Orlek_et_al.
Klebsiella sp.	LC019731.1	Plasmid	Orlek_et_al.
Klebsiella sp.	LC055503.1	Plasmid	Orlek_et_al.
Klebsiella sp.	LC155908.1	Plasmid	Orlek_et_al.
Klebsiella sp.	LC155909.1	Plasmid	Orlek_et_al.
Klebsiella sp.	LK391770.1	Plasmid	Orlek_et_al.
Klebsiella sp.	LN610760.1	Plasmid	Orlek_et_al.
Klebsiella sp.	LN623683.2	Plasmid	Orlek_et_al.
Klebsiella sp.	LN624486.1	Plasmid	Orlek_et_al.
Klebsiella sp.	LN735558.1	Plasmid	Orlek_et_al.
Klebsiella sp.	LN735559.1	Plasmid	Orlek_et_al.
Klebsiella sp.	LN735560.1	Plasmid	Orlek_et_al.
Klebsiella sp.	LN735561.1	Plasmid	Orlek_et_al.
Klebsiella sp.	LN794247.1	Plasmid	Orlek_et_al.
Klebsiella sp.	LN794248.1	Plasmid	Orlek_et_al.
Klebsiella sp.	LN831046.1	Plasmid	Orlek_et_al.
Klebsiella sp.	LN854558.1	Plasmid	Orlek_et_al.
Klebsiella sp.	LN854559.1	Plasmid	Orlek_et_al.
Klebsiella sp.	LN854560.1	Plasmid	Orlek_et_al.
Klebsiella sp.	LN864819.1	Plasmid	Orlek_et_al.
Klebsiella sp.	LN864820.1	Plasmid	Orlek_et_al.
Klebsiella sp.	LN864821.1	Plasmid	Orlek_et_al.
Klebsiella sp.	LN890286.1	Plasmid	Orlek_et_al.
Klebsiella sp.	LN897474.2	Plasmid	Orlek_et_al.
Klebsiella sp.	LN897475.2	Plasmid	Orlek_et_al.
Klebsiella sp.	LO017736.1	Plasmid	Orlek_et_al.
Klebsiella sp.	LO017737.1	Plasmid	Orlek_et_al.
Klebsiella sp.	LO017738.1	Plasmid	Orlek_et_al.
Klebsiella sp.	LT009688.1	Plasmid	Orlek_et_al.
Klebsiella sp.	LT009689.1	Plasmid	Orlek_et_al.
Klebsiella sp.	LT174531.1	Plasmid	Orlek_et_al.
Klebsiella sp.	LT575491.1	Plasmid	Orlek_et_al.
Klebsiella sp.	LT575492.1	Plasmid	Orlek_et_al.
Klebsiella sp.	NC_001371.1	Plasmid	Orlek_et_al.
Klebsiella sp.	NC_001373.1	Plasmid	Orlek_et_al.
Klebsiella sp.	NC_001378.1	Plasmid	Orlek_et_al.

Klebsiella sp.	NC_001537.1	Plasmid	Orlek_et_al.
Klebsiella sp.	NC_001735.4	Plasmid	Orlek_et_al.
Klebsiella sp.	NC_001740.1	Plasmid	Orlek_et_al.
Klebsiella sp.	NC_001848.1	Plasmid	Orlek_et_al.
Klebsiella sp.	NC_001898.1	Plasmid	Orlek_et_al.
Klebsiella sp.	NC_001910.1	Plasmid	Orlek_et_al.
Klebsiella sp.	NC_001911.1	Plasmid	Orlek_et_al.
Klebsiella sp.	NC_002056.1	Plasmid	Orlek_et_al.
Klebsiella sp.	NC_002070.1	Plasmid	Orlek_et_al.
Klebsiella sp.	NC_002090.1	Plasmid	Orlek_et_al.
Klebsiella sp.	NC_002119.1	Plasmid	Orlek_et_al.
Klebsiella sp.	NC_002120.1	Plasmid	Orlek_et_al.
Klebsiella sp.	NC_002122.1	Plasmid	Orlek_et_al.
Klebsiella sp.	NC_002127.1	Plasmid	Orlek_et_al.
Klebsiella sp.	NC_002128.1	Plasmid	Orlek_et_al.
Klebsiella sp.	NC_002134.1	Plasmid	Orlek_et_al.
Klebsiella sp.	NC_002142.1	Plasmid	Orlek_et_al.
Klebsiella sp.	NC_002144.1	Plasmid	Orlek_et_al.
Klebsiella sp.	NC_002145.1	Plasmid	Orlek_et_al.
Klebsiella sp.	NC_002252.1	Plasmid	Orlek_et_al.
Klebsiella sp.	NC_002253.1	Plasmid	Orlek_et_al.
Klebsiella sp.	NC_002305.1	Plasmid	Orlek_et_al.
Klebsiella sp.	NC_002483.1	Plasmid	Orlek_et_al.
Klebsiella sp.	NC_002487.1	Plasmid	Orlek_et_al.
Klebsiella sp.	NC_002497.1	Plasmid	Orlek_et_al.
Klebsiella sp.	NC_002498.1	Plasmid	Orlek_et_al.
Klebsiella sp.	NC_002523.4	Plasmid	Orlek_et_al.
Klebsiella sp.	NC_002525.1	Plasmid	Orlek_et_al.
Klebsiella sp.	NC_002610.1	Plasmid	Orlek_et_al.
Klebsiella sp.	NC_002632.1	Plasmid	Orlek_et_al.
Klebsiella sp.	NC_002638.1	Plasmid	Orlek_et_al.
Klebsiella sp.	NC_002698.1	Plasmid	Orlek_et_al.
Klebsiella sp.	NC_002773.1	Plasmid	Orlek_et_al.
Klebsiella sp.	NC_002809.1	Plasmid	Orlek_et_al.
Klebsiella sp.	NC_003079.1	Plasmid	Orlek_et_al.
Klebsiella sp.	NC_003114.1	Plasmid	Orlek_et_al.
Klebsiella sp.	NC_003131.1	Plasmid	Orlek_et_al.
Klebsiella sp.	NC_003132.1	Plasmid	Orlek_et_al.
Klebsiella sp.	NC_003134.1	Plasmid	Orlek_et_al.
Klebsiella sp.	NC_003277.1	Plasmid	Orlek_et_al.
Klebsiella sp.	NC_003292.1	Plasmid	Orlek_et_al.
Klebsiella sp.	NC_003384.1	Plasmid	Orlek_et_al.
Klebsiella sp.	NC_003385.1	Plasmid	Orlek_et_al.
Klebsiella sp.	NC_003425.1	Plasmid	Orlek_et_al.
Klebsiella sp.	NC_003455.1	Plasmid	Orlek_et_al.
Klebsiella sp.	NC_003456.1	Plasmid	Orlek_et_al.
Klebsiella sp.	NC_003457.1	Plasmid	Orlek_et_al.
Klebsiella sp.	NC_003486.1	Plasmid	Orlek_et_al.
Klebsiella sp.	NC_003789.1	Plasmid	Orlek_et_al.
Klebsiella sp.	NC_003905.1	Plasmid	Orlek_et_al.
Klebsiella sp.	NC_004429.1	Plasmid	Orlek_et_al.
Klebsiella sp.	NC_004445.1	Plasmid	Orlek_et_al.
Klebsiella sp.	NC_004446.1	Plasmid	Orlek_et_al.
Klebsiella sp.	NC_004464.2	Plasmid	Orlek_et_al.
Klebsiella sp.	NC_004555.1	Plasmid	Orlek_et_al.
Klebsiella sp.	NC_004564.1	Plasmid	Orlek_et_al.
Klebsiella sp.	NC_004834.1	Plasmid	Orlek_et_al.
Klebsiella sp.	NC_004835.1	Plasmid	Orlek_et_al.
Klebsiella sp.	NC_004836.1	Plasmid	Orlek_et_al.
Klebsiella sp.	NC_004837.1	Plasmid	Orlek_et_al.
Klebsiella sp.	NC_004838.1	Plasmid	Orlek_et_al.
Klebsiella sp.	NC_004839.1	Plasmid	Orlek_et_al.
Klebsiella sp.	NC_004843.1	Plasmid	Orlek_et_al.
Klebsiella sp.	NC_004851.1	Plasmid	Orlek_et_al.
Klebsiella sp.	NC_004936.1	Plasmid	Orlek_et_al.
Klebsiella sp.	NC_004940.1	Plasmid	Orlek_et_al.
Klebsiella sp.	NC_004989.1	Plasmid	Orlek_et_al.
Klebsiella sp.	NC_004998.1	Plasmid	Orlek_et_al.
Klebsiella sp.	NC_005002.1	Plasmid	Orlek_et_al.
Klebsiella sp.	NC_005014.1	Plasmid	Orlek_et_al.
Klebsiella sp.	NC_005015.1	Plasmid	Orlek_et_al.
Klebsiella sp.	NC_005017.1	Plasmid	Orlek_et_al.

Klebsiella sp.	NC_005018.1	Plasmid	Orlek_et_al.
Klebsiella sp.	NC_005019.1	Plasmid	Orlek_et_al.
Klebsiella sp.	NC_005211.1	Plasmid	Orlek_et_al.
Klebsiella sp.	NC_005246.1	Plasmid	Orlek_et_al.
Klebsiella sp.	NC_005247.1	Plasmid	Orlek_et_al.
Klebsiella sp.	NC_005248.1	Plasmid	Orlek_et_al.
Klebsiella sp.	NC_005249.1	Plasmid	Orlek_et_al.
Klebsiella sp.	NC_005324.1	Plasmid	Orlek_et_al.
Klebsiella sp.	NC_005327.1	Plasmid	Orlek_et_al.
Klebsiella sp.	NC_005570.1	Plasmid	Orlek_et_al.
Klebsiella sp.	NC_005706.1	Plasmid	Orlek_et_al.
Klebsiella sp.	NC_005813.1	Plasmid	Orlek_et_al.
Klebsiella sp.	NC_005814.1	Plasmid	Orlek_et_al.
Klebsiella sp.	NC_005815.1	Plasmid	Orlek_et_al.
Klebsiella sp.	NC_005816.1	Plasmid	Orlek_et_al.
Klebsiella sp.	NC_005862.1	Plasmid	Orlek_et_al.
Klebsiella sp.	NC_005910.1	Plasmid	Orlek_et_al.
Klebsiella sp.	NC_005911.1	Plasmid	Orlek_et_al.
Klebsiella sp.	NC_005923.1	Plasmid	Orlek_et_al.
Klebsiella sp.	NC_005970.1	Plasmid	Orlek_et_al.
Klebsiella sp.	NC_006153.2	Plasmid	Orlek_et_al.
Klebsiella sp.	NC_006154.1	Plasmid	Orlek_et_al.
Klebsiella sp.	NC_006323.1	Plasmid	Orlek_et_al.
Klebsiella sp.	NC_006625.1	Plasmid	Orlek_et_al.
Klebsiella sp.	NC_006671.1	Plasmid	Orlek_et_al.
Klebsiella sp.	NC_006815.1	Plasmid	Orlek_et_al.
Klebsiella sp.	NC_006816.1	Plasmid	Orlek_et_al.
Klebsiella sp.	NC_006855.1	Plasmid	Orlek_et_al.
Klebsiella sp.	NC_006856.1	Plasmid	Orlek_et_al.
Klebsiella sp.	NC_006881.1	Plasmid	Orlek_et_al.
Klebsiella sp.	NC_007182.1	Plasmid	Orlek_et_al.
Klebsiella sp.	NC_007183.1	Plasmid	Orlek_et_al.
Klebsiella sp.	NC_007184.1	Plasmid	Orlek_et_al.
Klebsiella sp.	NC_007185.1	Plasmid	Orlek_et_al.
Klebsiella sp.	NC_007186.1	Plasmid	Orlek_et_al.
Klebsiella sp.	NC_007187.1	Plasmid	Orlek_et_al.
Klebsiella sp.	NC_007208.1	Plasmid	Orlek_et_al.
Klebsiella sp.	NC_007365.1	Plasmid	Orlek_et_al.
Klebsiella sp.	NC_007385.1	Plasmid	Orlek_et_al.
Klebsiella sp.	NC_007414.1	Plasmid	Orlek_et_al.
Klebsiella sp.	NC_007607.1	Plasmid	Orlek_et_al.
Klebsiella sp.	NC_007608.1	Plasmid	Orlek_et_al.
Klebsiella sp.	NC_007635.1	Plasmid	Orlek_et_al.
Klebsiella sp.	NC_007675.1	Plasmid	Orlek_et_al.
Klebsiella sp.	NC_007682.3	Plasmid	Orlek_et_al.
Klebsiella sp.	NC_007713.1	Plasmid	Orlek_et_al.
Klebsiella sp.	NC_007714.1	Plasmid	Orlek_et_al.
Klebsiella sp.	NC_007715.1	Plasmid	Orlek_et_al.
Klebsiella sp.	NC_007941.1	Plasmid	Orlek_et_al.
Klebsiella sp.	NC_008053.1	Plasmid	Orlek_et_al.
Klebsiella sp.	NC_008118.1	Plasmid	Orlek_et_al.
Klebsiella sp.	NC_008119.1	Plasmid	Orlek_et_al.
Klebsiella sp.	NC_008120.1	Plasmid	Orlek_et_al.
Klebsiella sp.	NC_008121.1	Plasmid	Orlek_et_al.
Klebsiella sp.	NC_008122.1	Plasmid	Orlek_et_al.
Klebsiella sp.	NC_008439.1	Plasmid	Orlek_et_al.
Klebsiella sp.	NC_008444.1	Plasmid	Orlek_et_al.
Klebsiella sp.	NC_008460.1	Plasmid	Orlek_et_al.
Klebsiella sp.	NC_008486.1	Plasmid	Orlek_et_al.
Klebsiella sp.	NC_008487.1	Plasmid	Orlek_et_al.
Klebsiella sp.	NC_008488.1	Plasmid	Orlek_et_al.
Klebsiella sp.	NC_008489.1	Plasmid	Orlek_et_al.
Klebsiella sp.	NC_008490.1	Plasmid	Orlek_et_al.
Klebsiella sp.	NC_008597.1	Plasmid	Orlek_et_al.
Klebsiella sp.	NC_008791.1	Plasmid	Orlek_et_al.
Klebsiella sp.	NC_009132.1	Plasmid	Orlek_et_al.
Klebsiella sp.	NC_009133.1	Plasmid	Orlek_et_al.
Klebsiella sp.	NC_009139.1	Plasmid	Orlek_et_al.
Klebsiella sp.	NC_009140.1	Plasmid	Orlek_et_al.
Klebsiella sp.	NC_009141.1	Plasmid	Orlek_et_al.
Klebsiella sp.	NC_009344.1	Plasmid	Orlek_et_al.
Klebsiella sp.	NC_009345.1	Plasmid	Orlek_et_al.

Klebsiella sp.	NC_009346.1	Plasmid	Orlek_et_al.
Klebsiella sp.	NC_009347.1	Plasmid	Orlek_et_al.
Klebsiella sp.	NC_009377.1	Plasmid	Orlek_et_al.
Klebsiella sp.	NC_009378.1	Plasmid	Orlek_et_al.
Klebsiella sp.	NC_009425.1	Plasmid	Orlek_et_al.
Klebsiella sp.	NC_009595.1	Plasmid	Orlek_et_al.
Klebsiella sp.	NC_009596.1	Plasmid	Orlek_et_al.
Klebsiella sp.	NC_009602.1	Plasmid	Orlek_et_al.
Klebsiella sp.	NC_009649.1	Plasmid	Orlek_et_al.
Klebsiella sp.	NC_009650.1	Plasmid	Orlek_et_al.
Klebsiella sp.	NC_009651.1	Plasmid	Orlek_et_al.
Klebsiella sp.	NC_009652.1	Plasmid	Orlek_et_al.
Klebsiella sp.	NC_009653.1	Plasmid	Orlek_et_al.
Klebsiella sp.	NC_009704.1	Plasmid	Orlek_et_al.
Klebsiella sp.	NC_009705.1	Plasmid	Orlek_et_al.
Klebsiella sp.	NC_009716.1	Plasmid	Orlek_et_al.
Klebsiella sp.	NC_009779.1	Plasmid	Orlek_et_al.
Klebsiella sp.	NC_009780.1	Plasmid	Orlek_et_al.
Klebsiella sp.	NC_009781.1	Plasmid	Orlek_et_al.
Klebsiella sp.	NC_009786.1	Plasmid	Orlek_et_al.
Klebsiella sp.	NC_009787.1	Plasmid	Orlek_et_al.
Klebsiella sp.	NC_009788.1	Plasmid	Orlek_et_al.
Klebsiella sp.	NC_009789.1	Plasmid	Orlek_et_al.
Klebsiella sp.	NC_009790.1	Plasmid	Orlek_et_al.
Klebsiella sp.	NC_009791.1	Plasmid	Orlek_et_al.
Klebsiella sp.	NC_009793.1	Plasmid	Orlek_et_al.
Klebsiella sp.	NC_009794.1	Plasmid	Orlek_et_al.
Klebsiella sp.	NC_009807.1	Plasmid	Orlek_et_al.
Klebsiella sp.	NC_009829.1	Plasmid	Orlek_et_al.
Klebsiella sp.	NC_009837.1	Plasmid	Orlek_et_al.
Klebsiella sp.	NC_009838.1	Plasmid	Orlek_et_al.
Klebsiella sp.	NC_009980.1	Plasmid	Orlek_et_al.
Klebsiella sp.	NC_009981.1	Plasmid	Orlek_et_al.
Klebsiella sp.	NC_009982.1	Plasmid	Orlek_et_al.
Klebsiella sp.	NC_010064.1	Plasmid	Orlek_et_al.
Klebsiella sp.	NC_010119.1	Plasmid	Orlek_et_al.
Klebsiella sp.	NC_010157.1	Plasmid	Orlek_et_al.
Klebsiella sp.	NC_010158.1	Plasmid	Orlek_et_al.
Klebsiella sp.	NC_010257.1	Plasmid	Orlek_et_al.
Klebsiella sp.	NC_010259.1	Plasmid	Orlek_et_al.
Klebsiella sp.	NC_010261.1	Plasmid	Orlek_et_al.
Klebsiella sp.	NC_010377.1	Plasmid	Orlek_et_al.
Klebsiella sp.	NC_010378.1	Plasmid	Orlek_et_al.
Klebsiella sp.	NC_010409.1	Plasmid	Orlek_et_al.
Klebsiella sp.	NC_010421.1	Plasmid	Orlek_et_al.
Klebsiella sp.	NC_010422.1	Plasmid	Orlek_et_al.
Klebsiella sp.	NC_010485.1	Plasmid	Orlek_et_al.
Klebsiella sp.	NC_010486.1	Plasmid	Orlek_et_al.
Klebsiella sp.	NC_010487.1	Plasmid	Orlek_et_al.
Klebsiella sp.	NC_010488.1	Plasmid	Orlek_et_al.
Klebsiella sp.	NC_010499.1	Plasmid	Orlek_et_al.
Klebsiella sp.	NC_010500.1	Plasmid	Orlek_et_al.
Klebsiella sp.	NC_010555.1	Plasmid	Orlek_et_al.
Klebsiella sp.	NC_010558.1	Plasmid	Orlek_et_al.
Klebsiella sp.	NC_010635.1	Plasmid	Orlek_et_al.
Klebsiella sp.	NC_010643.1	Plasmid	Orlek_et_al.
Klebsiella sp.	NC_010656.1	Plasmid	Orlek_et_al.
Klebsiella sp.	NC_010657.1	Plasmid	Orlek_et_al.
Klebsiella sp.	NC_010659.1	Plasmid	Orlek_et_al.
Klebsiella sp.	NC_010660.1	Plasmid	Orlek_et_al.
Klebsiella sp.	NC_010672.1	Plasmid	Orlek_et_al.
Klebsiella sp.	NC_010693.1	Plasmid	Orlek_et_al.
Klebsiella sp.	NC_010695.1	Plasmid	Orlek_et_al.
Klebsiella sp.	NC_010696.1	Plasmid	Orlek_et_al.
Klebsiella sp.	NC_010697.1	Plasmid	Orlek_et_al.
Klebsiella sp.	NC_010699.1	Plasmid	Orlek_et_al.
Klebsiella sp.	NC_010716.1	Plasmid	Orlek_et_al.
Klebsiella sp.	NC_010719.1	Plasmid	Orlek_et_al.
Klebsiella sp.	NC_010720.1	Plasmid	Orlek_et_al.
Klebsiella sp.	NC_010726.1	Plasmid	Orlek_et_al.
Klebsiella sp.	NC_010796.1	Plasmid	Orlek_et_al.
Klebsiella sp.	NC_010860.1	Plasmid	Orlek_et_al.

Klebsiella sp.	NC_010862.1	Plasmid	Orlek_et_al.
Klebsiella sp.	NC_010870.1	Plasmid	Orlek_et_al.
Klebsiella sp.	NC_010883.1	Plasmid	Orlek_et_al.
Klebsiella sp.	NC_010885.1	Plasmid	Orlek_et_al.
Klebsiella sp.	NC_010886.1	Plasmid	Orlek_et_al.
Klebsiella sp.	NC_010894.1	Plasmid	Orlek_et_al.
Klebsiella sp.	NC_010896.1	Plasmid	Orlek_et_al.
Klebsiella sp.	NC_010898.1	Plasmid	Orlek_et_al.
Klebsiella sp.	NC_010904.1	Plasmid	Orlek_et_al.
Klebsiella sp.	NC_010993.1	Plasmid	Orlek_et_al.
Klebsiella sp.	NC_011076.1	Plasmid	Orlek_et_al.
Klebsiella sp.	NC_011077.1	Plasmid	Orlek_et_al.
Klebsiella sp.	NC_011078.1	Plasmid	Orlek_et_al.
Klebsiella sp.	NC_011079.1	Plasmid	Orlek_et_al.
Klebsiella sp.	NC_011081.1	Plasmid	Orlek_et_al.
Klebsiella sp.	NC_011082.1	Plasmid	Orlek_et_al.
Klebsiella sp.	NC_011092.1	Plasmid	Orlek_et_al.
Klebsiella sp.	NC_011093.1	Plasmid	Orlek_et_al.
Klebsiella sp.	NC_011148.1	Plasmid	Orlek_et_al.
Klebsiella sp.	NC_011204.1	Plasmid	Orlek_et_al.
Klebsiella sp.	NC_011214.1	Plasmid	Orlek_et_al.
Klebsiella sp.	NC_011215.1	Plasmid	Orlek_et_al.
Klebsiella sp.	NC_011227.1	Plasmid	Orlek_et_al.
Klebsiella sp.	NC_011228.1	Plasmid	Orlek_et_al.
Klebsiella sp.	NC_011266.1	Plasmid	Orlek_et_al.
Klebsiella sp.	NC_011281.1	Plasmid	Orlek_et_al.
Klebsiella sp.	NC_011282.1	Plasmid	Orlek_et_al.
Klebsiella sp.	NC_011350.1	Plasmid	Orlek_et_al.
Klebsiella sp.	NC_011351.1	Plasmid	Orlek_et_al.
Klebsiella sp.	NC_011382.1	Plasmid	Orlek_et_al.
Klebsiella sp.	NC_011383.1	Plasmid	Orlek_et_al.
Klebsiella sp.	NC_011385.1	Plasmid	Orlek_et_al.
Klebsiella sp.	NC_011404.1	Plasmid	Orlek_et_al.
Klebsiella sp.	NC_011405.1	Plasmid	Orlek_et_al.
Klebsiella sp.	NC_011406.1	Plasmid	Orlek_et_al.
Klebsiella sp.	NC_011407.1	Plasmid	Orlek_et_al.
Klebsiella sp.	NC_011408.1	Plasmid	Orlek_et_al.
Klebsiella sp.	NC_011411.1	Plasmid	Orlek_et_al.
Klebsiella sp.	NC_011413.1	Plasmid	Orlek_et_al.
Klebsiella sp.	NC_011416.1	Plasmid	Orlek_et_al.
Klebsiella sp.	NC_011418.1	Plasmid	Orlek_et_al.
Klebsiella sp.	NC_011419.1	Plasmid	Orlek_et_al.
Klebsiella sp.	NC_011422.1	Plasmid	Orlek_et_al.
Klebsiella sp.	NC_011511.1	Plasmid	Orlek_et_al.
Klebsiella sp.	NC_011512.1	Plasmid	Orlek_et_al.
Klebsiella sp.	NC_011513.1	Plasmid	Orlek_et_al.
Klebsiella sp.	NC_011514.1	Plasmid	Orlek_et_al.
Klebsiella sp.	NC_011604.1	Plasmid	Orlek_et_al.
Klebsiella sp.	NC_011617.1	Plasmid	Orlek_et_al.
Klebsiella sp.	NC_011640.1	Plasmid	Orlek_et_al.
Klebsiella sp.	NC_011641.1	Plasmid	Orlek_et_al.
Klebsiella sp.	NC_011739.1	Plasmid	Orlek_et_al.
Klebsiella sp.	NC_011743.1	Plasmid	Orlek_et_al.
Klebsiella sp.	NC_011749.1	Plasmid	Orlek_et_al.
Klebsiella sp.	NC_011752.1	Plasmid	Orlek_et_al.
Klebsiella sp.	NC_011754.1	Plasmid	Orlek_et_al.
Klebsiella sp.	NC_011767.1	Plasmid	Orlek_et_al.
Klebsiella sp.	NC_011795.1	Plasmid	Orlek_et_al.
Klebsiella sp.	NC_011799.1	Plasmid	Orlek_et_al.
Klebsiella sp.	NC_011812.1	Plasmid	Orlek_et_al.
Klebsiella sp.	NC_011878.1	Plasmid	Orlek_et_al.
Klebsiella sp.	NC_011897.1	Plasmid	Orlek_et_al.
Klebsiella sp.	NC_011917.1	Plasmid	Orlek_et_al.
Klebsiella sp.	NC_011964.1	Plasmid	Orlek_et_al.
Klebsiella sp.	NC_011977.1	Plasmid	Orlek_et_al.
Klebsiella sp.	NC_011980.1	Plasmid	Orlek_et_al.
Klebsiella sp.	NC_012006.1	Plasmid	Orlek_et_al.
Klebsiella sp.	NC_012124.1	Plasmid	Orlek_et_al.
Klebsiella sp.	NC_012208.1	Plasmid	Orlek_et_al.
Klebsiella sp.	NC_012209.1	Plasmid	Orlek_et_al.
Klebsiella sp.	NC_012487.1	Plasmid	Orlek_et_al.
Klebsiella sp.	NC_012555.1	Plasmid	Orlek_et_al.

Klebsiella sp.	NC_012556.1	Plasmid	Orlek_et_al.
Klebsiella sp.	NC_012690.1	Plasmid	Orlek_et_al.
Klebsiella sp.	NC_012692.1	Plasmid	Orlek_et_al.
Klebsiella sp.	NC_012693.1	Plasmid	Orlek_et_al.
Klebsiella sp.	NC_012752.1	Plasmid	Orlek_et_al.
Klebsiella sp.	NC_012882.1	Plasmid	Orlek_et_al.
Klebsiella sp.	NC_012886.1	Plasmid	Orlek_et_al.
Klebsiella sp.	NC_012916.1	Plasmid	Orlek_et_al.
Klebsiella sp.	NC_012944.1	Plasmid	Orlek_et_al.
Klebsiella sp.	NC_012961.1	Plasmid	Orlek_et_al.
Klebsiella sp.	NC_013010.1	Plasmid	Orlek_et_al.
Klebsiella sp.	NC_013104.1	Plasmid	Orlek_et_al.
Klebsiella sp.	NC_013120.1	Plasmid	Orlek_et_al.
Klebsiella sp.	NC_013121.1	Plasmid	Orlek_et_al.
Klebsiella sp.	NC_013122.1	Plasmid	Orlek_et_al.
Klebsiella sp.	NC_013175.1	Plasmid	Orlek_et_al.
Klebsiella sp.	NC_013263.1	Plasmid	Orlek_et_al.
Klebsiella sp.	NC_013264.1	Plasmid	Orlek_et_al.
Klebsiella sp.	NC_013265.1	Plasmid	Orlek_et_al.
Klebsiella sp.	NC_013283.1	Plasmid	Orlek_et_al.
Klebsiella sp.	NC_013284.1	Plasmid	Orlek_et_al.
Klebsiella sp.	NC_013285.1	Plasmid	Orlek_et_al.
Klebsiella sp.	NC_013354.1	Plasmid	Orlek_et_al.
Klebsiella sp.	NC_013362.1	Plasmid	Orlek_et_al.
Klebsiella sp.	NC_013363.1	Plasmid	Orlek_et_al.
Klebsiella sp.	NC_013365.1	Plasmid	Orlek_et_al.
Klebsiella sp.	NC_013366.1	Plasmid	Orlek_et_al.
Klebsiella sp.	NC_013367.1	Plasmid	Orlek_et_al.
Klebsiella sp.	NC_013368.1	Plasmid	Orlek_et_al.
Klebsiella sp.	NC_013369.1	Plasmid	Orlek_et_al.
Klebsiella sp.	NC_013370.1	Plasmid	Orlek_et_al.
Klebsiella sp.	NC_013437.1	Plasmid	Orlek_et_al.
Klebsiella sp.	NC_013503.1	Plasmid	Orlek_et_al.
Klebsiella sp.	NC_013507.1	Plasmid	Orlek_et_al.
Klebsiella sp.	NC_013509.1	Plasmid	Orlek_et_al.
Klebsiella sp.	NC_013542.1	Plasmid	Orlek_et_al.
Klebsiella sp.	NC_013549.1	Plasmid	Orlek_et_al.
Klebsiella sp.	NC_013589.1	Plasmid	Orlek_et_al.
Klebsiella sp.	NC_013652.1	Plasmid	Orlek_et_al.
Klebsiella sp.	NC_013655.1	Plasmid	Orlek_et_al.
Klebsiella sp.	NC_013717.1	Plasmid	Orlek_et_al.
Klebsiella sp.	NC_013718.1	Plasmid	Orlek_et_al.
Klebsiella sp.	NC_013719.1	Plasmid	Orlek_et_al.
Klebsiella sp.	NC_013727.1	Plasmid	Orlek_et_al.
Klebsiella sp.	NC_013728.1	Plasmid	Orlek_et_al.
Klebsiella sp.	NC_013782.1	Plasmid	Orlek_et_al.
Klebsiella sp.	NC_013942.1	Plasmid	Orlek_et_al.
Klebsiella sp.	NC_013950.1	Plasmid	Orlek_et_al.
Klebsiella sp.	NC_013951.1	Plasmid	Orlek_et_al.
Klebsiella sp.	NC_013954.1	Plasmid	Orlek_et_al.
Klebsiella sp.	NC_013957.1	Plasmid	Orlek_et_al.
Klebsiella sp.	NC_013962.1	Plasmid	Orlek_et_al.
Klebsiella sp.	NC_013972.1	Plasmid	Orlek_et_al.
Klebsiella sp.	NC_013973.1	Plasmid	Orlek_et_al.
Klebsiella sp.	NC_014003.1	Plasmid	Orlek_et_al.
Klebsiella sp.	NC_014016.1	Plasmid	Orlek_et_al.
Klebsiella sp.	NC_014017.1	Plasmid	Orlek_et_al.
Klebsiella sp.	NC_014022.1	Plasmid	Orlek_et_al.
Klebsiella sp.	NC_014027.1	Plasmid	Orlek_et_al.
Klebsiella sp.	NC_014107.1	Plasmid	Orlek_et_al.
Klebsiella sp.	NC_014108.1	Plasmid	Orlek_et_al.
Klebsiella sp.	NC_014170.1	Plasmid	Orlek_et_al.
Klebsiella sp.	NC_014208.1	Plasmid	Orlek_et_al.
Klebsiella sp.	NC_014227.1	Plasmid	Orlek_et_al.
Klebsiella sp.	NC_014231.1	Plasmid	Orlek_et_al.
Klebsiella sp.	NC_014232.1	Plasmid	Orlek_et_al.
Klebsiella sp.	NC_014233.1	Plasmid	Orlek_et_al.
Klebsiella sp.	NC_014234.1	Plasmid	Orlek_et_al.
Klebsiella sp.	NC_014235.1	Plasmid	Orlek_et_al.
Klebsiella sp.	NC_014258.1	Plasmid	Orlek_et_al.
Klebsiella sp.	NC_014304.1	Plasmid	Orlek_et_al.
Klebsiella sp.	NC_014305.1	Plasmid	Orlek_et_al.

Klebsiella sp.	NC_014312.1	Plasmid	Orlek_et_al.
Klebsiella sp.	NC_014354.1	Plasmid	Orlek_et_al.
Klebsiella sp.	NC_014356.1	Plasmid	Orlek_et_al.
Klebsiella sp.	NC_014368.1	Plasmid	Orlek_et_al.
Klebsiella sp.	NC_014382.1	Plasmid	Orlek_et_al.
Klebsiella sp.	NC_014383.1	Plasmid	Orlek_et_al.
Klebsiella sp.	NC_014384.1	Plasmid	Orlek_et_al.
Klebsiella sp.	NC_014385.1	Plasmid	Orlek_et_al.
Klebsiella sp.	NC_014476.2	Plasmid	Orlek_et_al.
Klebsiella sp.	NC_014477.1	Plasmid	Orlek_et_al.
Klebsiella sp.	NC_014478.1	Plasmid	Orlek_et_al.
Klebsiella sp.	NC_014543.1	Plasmid	Orlek_et_al.
Klebsiella sp.	NC_014561.1	Plasmid	Orlek_et_al.
Klebsiella sp.	NC_014563.1	Plasmid	Orlek_et_al.
Klebsiella sp.	NC_014615.1	Plasmid	Orlek_et_al.
Klebsiella sp.	NC_014725.1	Plasmid	Orlek_et_al.
Klebsiella sp.	NC_014838.1	Plasmid	Orlek_et_al.
Klebsiella sp.	NC_014839.1	Plasmid	Orlek_et_al.
Klebsiella sp.	NC_014840.1	Plasmid	Orlek_et_al.
Klebsiella sp.	NC_014841.1	Plasmid	Orlek_et_al.
Klebsiella sp.	NC_014842.1	Plasmid	Orlek_et_al.
Klebsiella sp.	NC_014843.1	Plasmid	Orlek_et_al.
Klebsiella sp.	NC_015054.1	Plasmid	Orlek_et_al.
Klebsiella sp.	NC_015055.1	Plasmid	Orlek_et_al.
Klebsiella sp.	NC_015056.1	Plasmid	Orlek_et_al.
Klebsiella sp.	NC_015062.1	Plasmid	Orlek_et_al.
Klebsiella sp.	NC_015063.1	Plasmid	Orlek_et_al.
Klebsiella sp.	NC_015068.1	Plasmid	Orlek_et_al.
Klebsiella sp.	NC_015154.1	Plasmid	Orlek_et_al.
Klebsiella sp.	NC_015175.1	Plasmid	Orlek_et_al.
Klebsiella sp.	NC_015392.1	Plasmid	Orlek_et_al.
Klebsiella sp.	NC_015472.1	Plasmid	Orlek_et_al.
Klebsiella sp.	NC_015475.1	Plasmid	Orlek_et_al.
Klebsiella sp.	NC_015515.1	Plasmid	Orlek_et_al.
Klebsiella sp.	NC_015570.1	Plasmid	Orlek_et_al.
Klebsiella sp.	NC_015575.1	Plasmid	Orlek_et_al.
Klebsiella sp.	NC_015599.1	Plasmid	Orlek_et_al.
Klebsiella sp.	NC_015739.1	Plasmid	Orlek_et_al.
Klebsiella sp.	NC_015872.1	Plasmid	Orlek_et_al.
Klebsiella sp.	NC_015963.1	Plasmid	Orlek_et_al.
Klebsiella sp.	NC_015965.1	Plasmid	Orlek_et_al.
Klebsiella sp.	NC_015969.1	Plasmid	Orlek_et_al.
Klebsiella sp.	NC_015972.1	Plasmid	Orlek_et_al.
Klebsiella sp.	NC_016036.1	Plasmid	Orlek_et_al.
Klebsiella sp.	NC_016039.1	Plasmid	Orlek_et_al.
Klebsiella sp.	NC_016151.1	Plasmid	Orlek_et_al.
Klebsiella sp.	NC_016515.1	Plasmid	Orlek_et_al.
Klebsiella sp.	NC_016817.1	Plasmid	Orlek_et_al.
Klebsiella sp.	NC_016819.1	Plasmid	Orlek_et_al.
Klebsiella sp.	NC_016823.1	Plasmid	Orlek_et_al.
Klebsiella sp.	NC_016824.1	Plasmid	Orlek_et_al.
Klebsiella sp.	NC_016825.1	Plasmid	Orlek_et_al.
Klebsiella sp.	NC_016833.1	Plasmid	Orlek_et_al.
Klebsiella sp.	NC_016834.1	Plasmid	Orlek_et_al.
Klebsiella sp.	NC_016835.1	Plasmid	Orlek_et_al.
Klebsiella sp.	NC_016838.1	Plasmid	Orlek_et_al.
Klebsiella sp.	NC_016839.1	Plasmid	Orlek_et_al.
Klebsiella sp.	NC_016840.1	Plasmid	Orlek_et_al.
Klebsiella sp.	NC_016841.1	Plasmid	Orlek_et_al.
Klebsiella sp.	NC_016846.1	Plasmid	Orlek_et_al.
Klebsiella sp.	NC_016847.1	Plasmid	Orlek_et_al.
Klebsiella sp.	NC_016855.1	Plasmid	Orlek_et_al.
Klebsiella sp.	NC_016861.1	Plasmid	Orlek_et_al.
Klebsiella sp.	NC_016862.1	Plasmid	Orlek_et_al.
Klebsiella sp.	NC_016864.1	Plasmid	Orlek_et_al.
Klebsiella sp.	NC_016966.1	Plasmid	Orlek_et_al.
Klebsiella sp.	NC_016974.1	Plasmid	Orlek_et_al.
Klebsiella sp.	NC_016976.1	Plasmid	Orlek_et_al.
Klebsiella sp.	NC_016979.1	Plasmid	Orlek_et_al.
Klebsiella sp.	NC_016980.1	Plasmid	Orlek_et_al.
Klebsiella sp.	NC_017054.1	Plasmid	Orlek_et_al.
Klebsiella sp.	NC_017060.1	Plasmid	Orlek_et_al.

Klebsiella sp.	NC_017153.1	Plasmid	Orlek_et_al.
Klebsiella sp.	NC_017155.1	Plasmid	Orlek_et_al.
Klebsiella sp.	NC_017156.1	Plasmid	Orlek_et_al.
Klebsiella sp.	NC_017157.1	Plasmid	Orlek_et_al.
Klebsiella sp.	NC_017158.1	Plasmid	Orlek_et_al.
Klebsiella sp.	NC_017159.1	Plasmid	Orlek_et_al.
Klebsiella sp.	NC_017169.1	Plasmid	Orlek_et_al.
Klebsiella sp.	NC_017170.1	Plasmid	Orlek_et_al.
Klebsiella sp.	NC_017257.1	Plasmid	Orlek_et_al.
Klebsiella sp.	NC_017258.1	Plasmid	Orlek_et_al.
Klebsiella sp.	NC_017260.1	Plasmid	Orlek_et_al.
Klebsiella sp.	NC_017261.1	Plasmid	Orlek_et_al.
Klebsiella sp.	NC_017263.1	Plasmid	Orlek_et_al.
Klebsiella sp.	NC_017264.1	Plasmid	Orlek_et_al.
Klebsiella sp.	NC_017266.1	Plasmid	Orlek_et_al.
Klebsiella sp.	NC_017318.1	Plasmid	Orlek_et_al.
Klebsiella sp.	NC_017319.1	Plasmid	Orlek_et_al.
Klebsiella sp.	NC_017320.1	Plasmid	Orlek_et_al.
Klebsiella sp.	NC_017321.1	Plasmid	Orlek_et_al.
Klebsiella sp.	NC_017329.1	Plasmid	Orlek_et_al.
Klebsiella sp.	NC_017330.1	Plasmid	Orlek_et_al.
Klebsiella sp.	NC_017388.1	Plasmid	Orlek_et_al.
Klebsiella sp.	NC_017389.1	Plasmid	Orlek_et_al.
Klebsiella sp.	NC_017391.1	Plasmid	Orlek_et_al.
Klebsiella sp.	NC_017392.1	Plasmid	Orlek_et_al.
Klebsiella sp.	NC_017442.1	Plasmid	Orlek_et_al.
Klebsiella sp.	NC_017444.1	Plasmid	Orlek_et_al.
Klebsiella sp.	NC_017446.1	Plasmid	Orlek_et_al.
Klebsiella sp.	NC_017533.1	Plasmid	Orlek_et_al.
Klebsiella sp.	NC_017541.1	Plasmid	Orlek_et_al.
Klebsiella sp.	NC_017553.1	Plasmid	Orlek_et_al.
Klebsiella sp.	NC_017565.1	Plasmid	Orlek_et_al.
Klebsiella sp.	NC_017624.1	Plasmid	Orlek_et_al.
Klebsiella sp.	NC_017627.1	Plasmid	Orlek_et_al.
Klebsiella sp.	NC_017629.1	Plasmid	Orlek_et_al.
Klebsiella sp.	NC_017630.1	Plasmid	Orlek_et_al.
Klebsiella sp.	NC_017637.1	Plasmid	Orlek_et_al.
Klebsiella sp.	NC_017639.1	Plasmid	Orlek_et_al.
Klebsiella sp.	NC_017640.1	Plasmid	Orlek_et_al.
Klebsiella sp.	NC_017642.1	Plasmid	Orlek_et_al.
Klebsiella sp.	NC_017643.1	Plasmid	Orlek_et_al.
Klebsiella sp.	NC_017645.1	Plasmid	Orlek_et_al.
Klebsiella sp.	NC_017647.1	Plasmid	Orlek_et_al.
Klebsiella sp.	NC_017648.1	Plasmid	Orlek_et_al.
Klebsiella sp.	NC_017649.1	Plasmid	Orlek_et_al.
Klebsiella sp.	NC_017650.1	Plasmid	Orlek_et_al.
Klebsiella sp.	NC_017653.1	Plasmid	Orlek_et_al.
Klebsiella sp.	NC_017654.1	Plasmid	Orlek_et_al.
Klebsiella sp.	NC_017655.1	Plasmid	Orlek_et_al.
Klebsiella sp.	NC_017657.1	Plasmid	Orlek_et_al.
Klebsiella sp.	NC_017658.1	Plasmid	Orlek_et_al.
Klebsiella sp.	NC_017659.1	Plasmid	Orlek_et_al.
Klebsiella sp.	NC_017661.1	Plasmid	Orlek_et_al.
Klebsiella sp.	NC_017662.1	Plasmid	Orlek_et_al.
Klebsiella sp.	NC_017665.1	Plasmid	Orlek_et_al.
Klebsiella sp.	NC_017718.1	Plasmid	Orlek_et_al.
Klebsiella sp.	NC_017719.1	Plasmid	Orlek_et_al.
Klebsiella sp.	NC_017720.1	Plasmid	Orlek_et_al.
Klebsiella sp.	NC_017722.1	Plasmid	Orlek_et_al.
Klebsiella sp.	NC_017724.1	Plasmid	Orlek_et_al.
Klebsiella sp.	NC_017903.1	Plasmid	Orlek_et_al.
Klebsiella sp.	NC_017907.1	Plasmid	Orlek_et_al.
Klebsiella sp.	NC_018107.1	Plasmid	Orlek_et_al.
Klebsiella sp.	NC_018953.1	Plasmid	Orlek_et_al.
Klebsiella sp.	NC_018954.1	Plasmid	Orlek_et_al.
Klebsiella sp.	NC_018966.1	Plasmid	Orlek_et_al.
Klebsiella sp.	NC_018977.1	Plasmid	Orlek_et_al.
Klebsiella sp.	NC_018978.1	Plasmid	Orlek_et_al.
Klebsiella sp.	NC_018985.1	Plasmid	Orlek_et_al.
Klebsiella sp.	NC_018994.1	Plasmid	Orlek_et_al.
Klebsiella sp.	NC_018995.1	Plasmid	Orlek_et_al.
Klebsiella sp.	NC_018996.1	Plasmid	Orlek_et_al.

Klebsiella sp.	NC_018997.1	Plasmid	Orlek_et_al.
Klebsiella sp.	NC_018998.1	Plasmid	Orlek_et_al.
Klebsiella sp.	NC_018999.1	Plasmid	Orlek_et_al.
Klebsiella sp.	NC_019000.1	Plasmid	Orlek_et_al.
Klebsiella sp.	NC_019001.1	Plasmid	Orlek_et_al.
Klebsiella sp.	NC_019002.1	Plasmid	Orlek_et_al.
Klebsiella sp.	NC_019013.1	Plasmid	Orlek_et_al.
Klebsiella sp.	NC_019033.1	Plasmid	Orlek_et_al.
Klebsiella sp.	NC_019037.1	Plasmid	Orlek_et_al.
Klebsiella sp.	NC_019038.1	Plasmid	Orlek_et_al.
Klebsiella sp.	NC_019039.1	Plasmid	Orlek_et_al.
Klebsiella sp.	NC_019040.1	Plasmid	Orlek_et_al.
Klebsiella sp.	NC_019043.1	Plasmid	Orlek_et_al.
Klebsiella sp.	NC_019044.1	Plasmid	Orlek_et_al.
Klebsiella sp.	NC_019045.2	Plasmid	Orlek_et_al.
Klebsiella sp.	NC_019046.1	Plasmid	Orlek_et_al.
Klebsiella sp.	NC_019047.1	Plasmid	Orlek_et_al.
Klebsiella sp.	NC_019049.1	Plasmid	Orlek_et_al.
Klebsiella sp.	NC_019050.1	Plasmid	Orlek_et_al.
Klebsiella sp.	NC_019051.1	Plasmid	Orlek_et_al.
Klebsiella sp.	NC_019052.1	Plasmid	Orlek_et_al.
Klebsiella sp.	NC_019053.1	Plasmid	Orlek_et_al.
Klebsiella sp.	NC_019054.1	Plasmid	Orlek_et_al.
Klebsiella sp.	NC_019056.1	Plasmid	Orlek_et_al.
Klebsiella sp.	NC_019057.1	Plasmid	Orlek_et_al.
Klebsiella sp.	NC_019058.1	Plasmid	Orlek_et_al.
Klebsiella sp.	NC_019059.1	Plasmid	Orlek_et_al.
Klebsiella sp.	NC_019060.1	Plasmid	Orlek_et_al.
Klebsiella sp.	NC_019061.1	Plasmid	Orlek_et_al.
Klebsiella sp.	NC_019062.1	Plasmid	Orlek_et_al.
Klebsiella sp.	NC_019063.1	Plasmid	Orlek_et_al.
Klebsiella sp.	NC_019065.1	Plasmid	Orlek_et_al.
Klebsiella sp.	NC_019066.1	Plasmid	Orlek_et_al.
Klebsiella sp.	NC_019067.1	Plasmid	Orlek_et_al.
Klebsiella sp.	NC_019068.1	Plasmid	Orlek_et_al.
Klebsiella sp.	NC_019069.1	Plasmid	Orlek_et_al.
Klebsiella sp.	NC_019070.1	Plasmid	Orlek_et_al.
Klebsiella sp.	NC_019071.1	Plasmid	Orlek_et_al.
Klebsiella sp.	NC_019072.1	Plasmid	Orlek_et_al.
Klebsiella sp.	NC_019073.1	Plasmid	Orlek_et_al.
Klebsiella sp.	NC_019074.1	Plasmid	Orlek_et_al.
Klebsiella sp.	NC_019075.1	Plasmid	Orlek_et_al.
Klebsiella sp.	NC_019076.1	Plasmid	Orlek_et_al.
Klebsiella sp.	NC_019077.1	Plasmid	Orlek_et_al.
Klebsiella sp.	NC_019078.1	Plasmid	Orlek_et_al.
Klebsiella sp.	NC_019079.1	Plasmid	Orlek_et_al.
Klebsiella sp.	NC_019080.1	Plasmid	Orlek_et_al.
Klebsiella sp.	NC_019081.1	Plasmid	Orlek_et_al.
Klebsiella sp.	NC_019082.1	Plasmid	Orlek_et_al.
Klebsiella sp.	NC_019083.1	Plasmid	Orlek_et_al.
Klebsiella sp.	NC_019084.1	Plasmid	Orlek_et_al.
Klebsiella sp.	NC_019085.1	Plasmid	Orlek_et_al.
Klebsiella sp.	NC_019086.1	Plasmid	Orlek_et_al.
Klebsiella sp.	NC_019087.1	Plasmid	Orlek_et_al.
Klebsiella sp.	NC_019088.1	Plasmid	Orlek_et_al.
Klebsiella sp.	NC_019089.1	Plasmid	Orlek_et_al.
Klebsiella sp.	NC_019090.1	Plasmid	Orlek_et_al.
Klebsiella sp.	NC_019091.1	Plasmid	Orlek_et_al.
Klebsiella sp.	NC_019092.1	Plasmid	Orlek_et_al.
Klebsiella sp.	NC_019093.1	Plasmid	Orlek_et_al.
Klebsiella sp.	NC_019094.1	Plasmid	Orlek_et_al.
Klebsiella sp.	NC_019095.1	Plasmid	Orlek_et_al.
Klebsiella sp.	NC_019096.1	Plasmid	Orlek_et_al.
Klebsiella sp.	NC_019097.1	Plasmid	Orlek_et_al.
Klebsiella sp.	NC_019098.1	Plasmid	Orlek_et_al.
Klebsiella sp.	NC_019099.1	Plasmid	Orlek_et_al.
Klebsiella sp.	NC_019101.1	Plasmid	Orlek_et_al.
Klebsiella sp.	NC_019102.1	Plasmid	Orlek_et_al.
Klebsiella sp.	NC_019103.1	Plasmid	Orlek_et_al.
Klebsiella sp.	NC_019104.1	Plasmid	Orlek_et_al.
Klebsiella sp.	NC_019105.1	Plasmid	Orlek_et_al.
Klebsiella sp.	NC_019106.1	Plasmid	Orlek_et_al.

Klebsiella sp.	NC_019107.1	Plasmid	Orlek_et_al.
Klebsiella sp.	NC_019108.1	Plasmid	Orlek_et_al.
Klebsiella sp.	NC_019109.1	Plasmid	Orlek_et_al.
Klebsiella sp.	NC_019110.1	Plasmid	Orlek_et_al.
Klebsiella sp.	NC_019111.1	Plasmid	Orlek_et_al.
Klebsiella sp.	NC_019112.1	Plasmid	Orlek_et_al.
Klebsiella sp.	NC_019113.1	Plasmid	Orlek_et_al.
Klebsiella sp.	NC_019114.1	Plasmid	Orlek_et_al.
Klebsiella sp.	NC_019115.1	Plasmid	Orlek_et_al.
Klebsiella sp.	NC_019116.1	Plasmid	Orlek_et_al.
Klebsiella sp.	NC_019117.1	Plasmid	Orlek_et_al.
Klebsiella sp.	NC_019118.1	Plasmid	Orlek_et_al.
Klebsiella sp.	NC_019120.1	Plasmid	Orlek_et_al.
Klebsiella sp.	NC_019121.1	Plasmid	Orlek_et_al.
Klebsiella sp.	NC_019122.1	Plasmid	Orlek_et_al.
Klebsiella sp.	NC_019123.1	Plasmid	Orlek_et_al.
Klebsiella sp.	NC_019124.1	Plasmid	Orlek_et_al.
Klebsiella sp.	NC_019125.1	Plasmid	Orlek_et_al.
Klebsiella sp.	NC_019126.1	Plasmid	Orlek_et_al.
Klebsiella sp.	NC_019127.1	Plasmid	Orlek_et_al.
Klebsiella sp.	NC_019128.1	Plasmid	Orlek_et_al.
Klebsiella sp.	NC_019129.1	Plasmid	Orlek_et_al.
Klebsiella sp.	NC_019130.1	Plasmid	Orlek_et_al.
Klebsiella sp.	NC_019131.1	Plasmid	Orlek_et_al.
Klebsiella sp.	NC_019132.1	Plasmid	Orlek_et_al.
Klebsiella sp.	NC_019133.1	Plasmid	Orlek_et_al.
Klebsiella sp.	NC_019134.1	Plasmid	Orlek_et_al.
Klebsiella sp.	NC_019135.1	Plasmid	Orlek_et_al.
Klebsiella sp.	NC_019136.1	Plasmid	Orlek_et_al.
Klebsiella sp.	NC_019137.1	Plasmid	Orlek_et_al.
Klebsiella sp.	NC_019151.1	Plasmid	Orlek_et_al.
Klebsiella sp.	NC_019152.1	Plasmid	Orlek_et_al.
Klebsiella sp.	NC_019153.1	Plasmid	Orlek_et_al.
Klebsiella sp.	NC_019154.1	Plasmid	Orlek_et_al.
Klebsiella sp.	NC_019155.1	Plasmid	Orlek_et_al.
Klebsiella sp.	NC_019156.1	Plasmid	Orlek_et_al.
Klebsiella sp.	NC_019157.1	Plasmid	Orlek_et_al.
Klebsiella sp.	NC_019158.1	Plasmid	Orlek_et_al.
Klebsiella sp.	NC_019159.1	Plasmid	Orlek_et_al.
Klebsiella sp.	NC_019160.1	Plasmid	Orlek_et_al.
Klebsiella sp.	NC_019161.1	Plasmid	Orlek_et_al.
Klebsiella sp.	NC_019162.1	Plasmid	Orlek_et_al.
Klebsiella sp.	NC_019163.1	Plasmid	Orlek_et_al.
Klebsiella sp.	NC_019165.1	Plasmid	Orlek_et_al.
Klebsiella sp.	NC_019166.1	Plasmid	Orlek_et_al.
Klebsiella sp.	NC_019187.1	Plasmid	Orlek_et_al.
Klebsiella sp.	NC_019188.1	Plasmid	Orlek_et_al.
Klebsiella sp.	NC_019197.1	Plasmid	Orlek_et_al.
Klebsiella sp.	NC_019203.1	Plasmid	Orlek_et_al.
Klebsiella sp.	NC_019204.1	Plasmid	Orlek_et_al.
Klebsiella sp.	NC_019221.1	Plasmid	Orlek_et_al.
Klebsiella sp.	NC_019222.1	Plasmid	Orlek_et_al.
Klebsiella sp.	NC_019223.1	Plasmid	Orlek_et_al.
Klebsiella sp.	NC_019224.1	Plasmid	Orlek_et_al.
Klebsiella sp.	NC_019234.1	Plasmid	Orlek_et_al.
Klebsiella sp.	NC_019242.1	Plasmid	Orlek_et_al.
Klebsiella sp.	NC_019249.1	Plasmid	Orlek_et_al.
Klebsiella sp.	NC_019250.1	Plasmid	Orlek_et_al.
Klebsiella sp.	NC_019251.1	Plasmid	Orlek_et_al.
Klebsiella sp.	NC_019254.1	Plasmid	Orlek_et_al.
Klebsiella sp.	NC_019256.1	Plasmid	Orlek_et_al.
Klebsiella sp.	NC_019267.1	Plasmid	Orlek_et_al.
Klebsiella sp.	NC_019269.1	Plasmid	Orlek_et_al.
Klebsiella sp.	NC_019279.1	Plasmid	Orlek_et_al.
Klebsiella sp.	NC_019286.1	Plasmid	Orlek_et_al.
Klebsiella sp.	NC_019295.1	Plasmid	Orlek_et_al.
Klebsiella sp.	NC_019296.1	Plasmid	Orlek_et_al.
Klebsiella sp.	NC_019297.1	Plasmid	Orlek_et_al.
Klebsiella sp.	NC_019298.1	Plasmid	Orlek_et_al.
Klebsiella sp.	NC_019310.1	Plasmid	Orlek_et_al.
Klebsiella sp.	NC_019335.1	Plasmid	Orlek_et_al.
Klebsiella sp.	NC_019336.1	Plasmid	Orlek_et_al.

Klebsiella sp.	NC_019342.1	Plasmid	Orlek_et_al.
Klebsiella sp.	NC_019343.1	Plasmid	Orlek_et_al.
Klebsiella sp.	NC_019344.1	Plasmid	Orlek_et_al.
Klebsiella sp.	NC_019346.1	Plasmid	Orlek_et_al.
Klebsiella sp.	NC_019357.1	Plasmid	Orlek_et_al.
Klebsiella sp.	NC_019360.1	Plasmid	Orlek_et_al.
Klebsiella sp.	NC_019368.1	Plasmid	Orlek_et_al.
Klebsiella sp.	NC_019373.1	Plasmid	Orlek_et_al.
Klebsiella sp.	NC_019374.1	Plasmid	Orlek_et_al.
Klebsiella sp.	NC_019375.1	Plasmid	Orlek_et_al.
Klebsiella sp.	NC_019384.1	Plasmid	Orlek_et_al.
Klebsiella sp.	NC_019389.1	Plasmid	Orlek_et_al.
Klebsiella sp.	NC_019390.1	Plasmid	Orlek_et_al.
Klebsiella sp.	NC_019424.1	Plasmid	Orlek_et_al.
Klebsiella sp.	NC_019888.1	Plasmid	Orlek_et_al.
Klebsiella sp.	NC_019889.1	Plasmid	Orlek_et_al.
Klebsiella sp.	NC_019899.1	Plasmid	Orlek_et_al.
Klebsiella sp.	NC_019982.1	Plasmid	Orlek_et_al.
Klebsiella sp.	NC_019983.1	Plasmid	Orlek_et_al.
Klebsiella sp.	NC_019984.1	Plasmid	Orlek_et_al.
Klebsiella sp.	NC_019986.1	Plasmid	Orlek_et_al.
Klebsiella sp.	NC_019987.1	Plasmid	Orlek_et_al.
Klebsiella sp.	NC_019988.1	Plasmid	Orlek_et_al.
Klebsiella sp.	NC_019989.1	Plasmid	Orlek_et_al.
Klebsiella sp.	NC_019990.1	Plasmid	Orlek_et_al.
Klebsiella sp.	NC_019991.1	Plasmid	Orlek_et_al.
Klebsiella sp.	NC_020086.1	Plasmid	Orlek_et_al.
Klebsiella sp.	NC_020087.1	Plasmid	Orlek_et_al.
Klebsiella sp.	NC_020088.1	Plasmid	Orlek_et_al.
Klebsiella sp.	NC_020122.1	Plasmid	Orlek_et_al.
Klebsiella sp.	NC_020123.1	Plasmid	Orlek_et_al.
Klebsiella sp.	NC_020131.1	Plasmid	Orlek_et_al.
Klebsiella sp.	NC_020132.1	Plasmid	Orlek_et_al.
Klebsiella sp.	NC_020180.1	Plasmid	Orlek_et_al.
Klebsiella sp.	NC_020212.1	Plasmid	Orlek_et_al.
Klebsiella sp.	NC_020251.1	Plasmid	Orlek_et_al.
Klebsiella sp.	NC_020261.1	Plasmid	Orlek_et_al.
Klebsiella sp.	NC_020262.1	Plasmid	Orlek_et_al.
Klebsiella sp.	NC_020263.1	Plasmid	Orlek_et_al.
Klebsiella sp.	NC_020270.1	Plasmid	Orlek_et_al.
Klebsiella sp.	NC_020271.1	Plasmid	Orlek_et_al.
Klebsiella sp.	NC_020278.2	Plasmid	Orlek_et_al.
Klebsiella sp.	NC_020279.1	Plasmid	Orlek_et_al.
Klebsiella sp.	NC_020280.1	Plasmid	Orlek_et_al.
Klebsiella sp.	NC_020281.1	Plasmid	Orlek_et_al.
Klebsiella sp.	NC_020282.1	Plasmid	Orlek_et_al.
Klebsiella sp.	NC_020295.1	Plasmid	Orlek_et_al.
Klebsiella sp.	NC_020306.1	Plasmid	Orlek_et_al.
Klebsiella sp.	NC_020308.1	Plasmid	Orlek_et_al.
Klebsiella sp.	NC_020412.1	Plasmid	Orlek_et_al.
Klebsiella sp.	NC_020413.1	Plasmid	Orlek_et_al.
Klebsiella sp.	NC_020552.1	Plasmid	Orlek_et_al.
Klebsiella sp.	NC_020811.1	Plasmid	Orlek_et_al.
Klebsiella sp.	NC_020893.1	Plasmid	Orlek_et_al.
Klebsiella sp.	NC_020915.1	Plasmid	Orlek_et_al.
Klebsiella sp.	NC_020916.1	Plasmid	Orlek_et_al.
Klebsiella sp.	NC_020918.1	Plasmid	Orlek_et_al.
Klebsiella sp.	NC_020919.1	Plasmid	Orlek_et_al.
Klebsiella sp.	NC_020920.1	Plasmid	Orlek_et_al.
Klebsiella sp.	NC_020921.1	Plasmid	Orlek_et_al.
Klebsiella sp.	NC_020991.1	Plasmid	Orlek_et_al.
Klebsiella sp.	NC_021078.1	Plasmid	Orlek_et_al.
Klebsiella sp.	NC_021079.1	Plasmid	Orlek_et_al.
Klebsiella sp.	NC_021087.1	Plasmid	Orlek_et_al.
Klebsiella sp.	NC_021155.1	Plasmid	Orlek_et_al.
Klebsiella sp.	NC_021156.1	Plasmid	Orlek_et_al.
Klebsiella sp.	NC_021157.1	Plasmid	Orlek_et_al.
Klebsiella sp.	NC_021180.1	Plasmid	Orlek_et_al.
Klebsiella sp.	NC_021198.1	Plasmid	Orlek_et_al.
Klebsiella sp.	NC_021199.1	Plasmid	Orlek_et_al.
Klebsiella sp.	NC_021231.1	Plasmid	Orlek_et_al.
Klebsiella sp.	NC_021238.1	Plasmid	Orlek_et_al.

Klebsiella sp.	NC_021293.1	Plasmid	Orlek_et_al.
Klebsiella sp.	NC_021488.1	Plasmid	Orlek_et_al.
Klebsiella sp.	NC_021492.1	Plasmid	Orlek_et_al.
Klebsiella sp.	NC_021501.1	Plasmid	Orlek_et_al.
Klebsiella sp.	NC_021502.1	Plasmid	Orlek_et_al.
Klebsiella sp.	NC_021511.1	Plasmid	Orlek_et_al.
Klebsiella sp.	NC_021522.1	Plasmid	Orlek_et_al.
Klebsiella sp.	NC_021523.1	Plasmid	Orlek_et_al.
Klebsiella sp.	NC_021524.1	Plasmid	Orlek_et_al.
Klebsiella sp.	NC_021570.1	Plasmid	Orlek_et_al.
Klebsiella sp.	NC_021576.1	Plasmid	Orlek_et_al.
Klebsiella sp.	NC_021594.1	Plasmid	Orlek_et_al.
Klebsiella sp.	NC_021622.1	Plasmid	Orlek_et_al.
Klebsiella sp.	NC_021654.1	Plasmid	Orlek_et_al.
Klebsiella sp.	NC_021655.1	Plasmid	Orlek_et_al.
Klebsiella sp.	NC_021656.1	Plasmid	Orlek_et_al.
Klebsiella sp.	NC_021660.2	Plasmid	Orlek_et_al.
Klebsiella sp.	NC_021664.2	Plasmid	Orlek_et_al.
Klebsiella sp.	NC_021666.1	Plasmid	Orlek_et_al.
Klebsiella sp.	NC_021667.1	Plasmid	Orlek_et_al.
Klebsiella sp.	NC_021742.1	Plasmid	Orlek_et_al.
Klebsiella sp.	NC_021811.1	Plasmid	Orlek_et_al.
Klebsiella sp.	NC_021813.2	Plasmid	Orlek_et_al.
Klebsiella sp.	NC_021815.1	Plasmid	Orlek_et_al.
Klebsiella sp.	NC_021816.1	Plasmid	Orlek_et_al.
Klebsiella sp.	NC_021817.1	Plasmid	Orlek_et_al.
Klebsiella sp.	NC_021819.1	Plasmid	Orlek_et_al.
Klebsiella sp.	NC_021835.1	Plasmid	Orlek_et_al.
Klebsiella sp.	NC_021836.1	Plasmid	Orlek_et_al.
Klebsiella sp.	NC_021841.1	Plasmid	Orlek_et_al.
Klebsiella sp.	NC_021842.2	Plasmid	Orlek_et_al.
Klebsiella sp.	NC_021843.1	Plasmid	Orlek_et_al.
Klebsiella sp.	NC_021845.1	Plasmid	Orlek_et_al.
Klebsiella sp.	NC_021869.1	Plasmid	Orlek_et_al.
Klebsiella sp.	NC_021871.1	Plasmid	Orlek_et_al.
Klebsiella sp.	NC_021922.1	Plasmid	Orlek_et_al.
Klebsiella sp.	NC_021927.1	Plasmid	Orlek_et_al.
Klebsiella sp.	NC_022078.1	Plasmid	Orlek_et_al.
Klebsiella sp.	NC_022083.1	Plasmid	Orlek_et_al.
Klebsiella sp.	NC_022105.1	Plasmid	Orlek_et_al.
Klebsiella sp.	NC_022197.1	Plasmid	Orlek_et_al.
Klebsiella sp.	NC_022267.1	Plasmid	Orlek_et_al.
Klebsiella sp.	NC_022333.1	Plasmid	Orlek_et_al.
Klebsiella sp.	NC_022371.1	Plasmid	Orlek_et_al.
Klebsiella sp.	NC_022372.1	Plasmid	Orlek_et_al.
Klebsiella sp.	NC_022374.1	Plasmid	Orlek_et_al.
Klebsiella sp.	NC_022375.1	Plasmid	Orlek_et_al.
Klebsiella sp.	NC_022376.1	Plasmid	Orlek_et_al.
Klebsiella sp.	NC_022377.1	Plasmid	Orlek_et_al.
Klebsiella sp.	NC_022520.1	Plasmid	Orlek_et_al.
Klebsiella sp.	NC_022522.2	Plasmid	Orlek_et_al.
Klebsiella sp.	NC_022533.1	Plasmid	Orlek_et_al.
Klebsiella sp.	NC_022534.1	Plasmid	Orlek_et_al.
Klebsiella sp.	NC_022570.1	Plasmid	Orlek_et_al.
Klebsiella sp.	NC_022585.1	Plasmid	Orlek_et_al.
Klebsiella sp.	NC_022589.1	Plasmid	Orlek_et_al.
Klebsiella sp.	NC_022609.1	Plasmid	Orlek_et_al.
Klebsiella sp.	NC_022652.1	Plasmid	Orlek_et_al.
Klebsiella sp.	NC_022740.1	Plasmid	Orlek_et_al.
Klebsiella sp.	NC_022741.1	Plasmid	Orlek_et_al.
Klebsiella sp.	NC_022742.1	Plasmid	Orlek_et_al.
Klebsiella sp.	NC_022743.1	Plasmid	Orlek_et_al.
Klebsiella sp.	NC_022885.1	Plasmid	Orlek_et_al.
Klebsiella sp.	NC_022963.1	Plasmid	Orlek_et_al.
Klebsiella sp.	NC_022992.1	Plasmid	Orlek_et_al.
Klebsiella sp.	NC_022996.1	Plasmid	Orlek_et_al.
Klebsiella sp.	NC_023024.1	Plasmid	Orlek_et_al.
Klebsiella sp.	NC_023025.1	Plasmid	Orlek_et_al.
Klebsiella sp.	NC_023027.1	Plasmid	Orlek_et_al.
Klebsiella sp.	NC_023056.1	Plasmid	Orlek_et_al.
Klebsiella sp.	NC_023071.1	Plasmid	Orlek_et_al.
Klebsiella sp.	NC_023072.1	Plasmid	Orlek_et_al.

Klebsiella sp.	NC_023275.1	Plasmid	Orlek_et_al.
Klebsiella sp.	NC_023276.1	Plasmid	Orlek_et_al.
Klebsiella sp.	NC_023277.2	Plasmid	Orlek_et_al.
Klebsiella sp.	NC_023289.2	Plasmid	Orlek_et_al.
Klebsiella sp.	NC_023290.1	Plasmid	Orlek_et_al.
Klebsiella sp.	NC_023314.1	Plasmid	Orlek_et_al.
Klebsiella sp.	NC_023315.1	Plasmid	Orlek_et_al.
Klebsiella sp.	NC_023317.1	Plasmid	Orlek_et_al.
Klebsiella sp.	NC_023318.1	Plasmid	Orlek_et_al.
Klebsiella sp.	NC_023319.1	Plasmid	Orlek_et_al.
Klebsiella sp.	NC_023320.1	Plasmid	Orlek_et_al.
Klebsiella sp.	NC_023321.1	Plasmid	Orlek_et_al.
Klebsiella sp.	NC_023323.1	Plasmid	Orlek_et_al.
Klebsiella sp.	NC_023324.1	Plasmid	Orlek_et_al.
Klebsiella sp.	NC_023325.1	Plasmid	Orlek_et_al.
Klebsiella sp.	NC_023326.1	Plasmid	Orlek_et_al.
Klebsiella sp.	NC_023327.1	Plasmid	Orlek_et_al.
Klebsiella sp.	NC_023328.1	Plasmid	Orlek_et_al.
Klebsiella sp.	NC_023329.1	Plasmid	Orlek_et_al.
Klebsiella sp.	NC_023330.1	Plasmid	Orlek_et_al.
Klebsiella sp.	NC_023331.1	Plasmid	Orlek_et_al.
Klebsiella sp.	NC_023332.1	Plasmid	Orlek_et_al.
Klebsiella sp.	NC_023333.1	Plasmid	Orlek_et_al.
Klebsiella sp.	NC_023334.1	Plasmid	Orlek_et_al.
Klebsiella sp.	NC_023898.1	Plasmid	Orlek_et_al.
Klebsiella sp.	NC_023899.1	Plasmid	Orlek_et_al.
Klebsiella sp.	NC_023900.1	Plasmid	Orlek_et_al.
Klebsiella sp.	NC_023901.1	Plasmid	Orlek_et_al.
Klebsiella sp.	NC_023903.1	Plasmid	Orlek_et_al.
Klebsiella sp.	NC_023904.1	Plasmid	Orlek_et_al.
Klebsiella sp.	NC_023905.1	Plasmid	Orlek_et_al.
Klebsiella sp.	NC_023906.1	Plasmid	Orlek_et_al.
Klebsiella sp.	NC_023907.1	Plasmid	Orlek_et_al.
Klebsiella sp.	NC_023908.1	Plasmid	Orlek_et_al.
Klebsiella sp.	NC_023909.1	Plasmid	Orlek_et_al.
Klebsiella sp.	NC_023910.1	Plasmid	Orlek_et_al.
Klebsiella sp.	NC_023911.1	Plasmid	Orlek_et_al.
Klebsiella sp.	NC_023912.1	Plasmid	Orlek_et_al.
Klebsiella sp.	NC_023914.1	Plasmid	Orlek_et_al.
Klebsiella sp.	NC_023915.1	Plasmid	Orlek_et_al.
Klebsiella sp.	NC_023916.1	Plasmid	Orlek_et_al.
Klebsiella sp.	NC_024954.1	Plasmid	Orlek_et_al.
Klebsiella sp.	NC_024955.2	Plasmid	Orlek_et_al.
Klebsiella sp.	NC_024956.1	Plasmid	Orlek_et_al.
Klebsiella sp.	NC_024960.1	Plasmid	Orlek_et_al.
Klebsiella sp.	NC_024961.1	Plasmid	Orlek_et_al.
Klebsiella sp.	NC_024962.1	Plasmid	Orlek_et_al.
Klebsiella sp.	NC_024967.1	Plasmid	Orlek_et_al.
Klebsiella sp.	NC_024968.1	Plasmid	Orlek_et_al.
Klebsiella sp.	NC_024969.1	Plasmid	Orlek_et_al.
Klebsiella sp.	NC_024974.1	Plasmid	Orlek_et_al.
Klebsiella sp.	NC_024975.1	Plasmid	Orlek_et_al.
Klebsiella sp.	NC_024976.1	Plasmid	Orlek_et_al.
Klebsiella sp.	NC_024977.1	Plasmid	Orlek_et_al.
Klebsiella sp.	NC_024978.1	Plasmid	Orlek_et_al.
Klebsiella sp.	NC_024979.1	Plasmid	Orlek_et_al.
Klebsiella sp.	NC_024980.1	Plasmid	Orlek_et_al.
Klebsiella sp.	NC_024981.1	Plasmid	Orlek_et_al.
Klebsiella sp.	NC_024982.1	Plasmid	Orlek_et_al.
Klebsiella sp.	NC_024983.1	Plasmid	Orlek_et_al.
Klebsiella sp.	NC_024987.1	Plasmid	Orlek_et_al.
Klebsiella sp.	NC_024992.1	Plasmid	Orlek_et_al.
Klebsiella sp.	NC_024996.1	Plasmid	Orlek_et_al.
Klebsiella sp.	NC_024997.1	Plasmid	Orlek_et_al.
Klebsiella sp.	NC_025003.1	Plasmid	Orlek_et_al.
Klebsiella sp.	NC_025013.1	Plasmid	Orlek_et_al.
Klebsiella sp.	NC_025019.1	Plasmid	Orlek_et_al.
Klebsiella sp.	NC_025021.1	Plasmid	Orlek_et_al.
Klebsiella sp.	NC_025043.1	Plasmid	Orlek_et_al.
Klebsiella sp.	NC_025044.1	Plasmid	Orlek_et_al.
Klebsiella sp.	NC_025058.1	Plasmid	Orlek_et_al.
Klebsiella sp.	NC_025105.1	Plasmid	Orlek_et_al.

Klebsiella sp.	NC_025106.1	Plasmid	Orlek_et_al.
Klebsiella sp.	NC_025130.1	Plasmid	Orlek_et_al.
Klebsiella sp.	NC_025131.1	Plasmid	Orlek_et_al.
Klebsiella sp.	NC_025134.1	Plasmid	Orlek_et_al.
Klebsiella sp.	NC_025138.1	Plasmid	Orlek_et_al.
Klebsiella sp.	NC_025139.1	Plasmid	Orlek_et_al.
Klebsiella sp.	NC_025140.1	Plasmid	Orlek_et_al.
Klebsiella sp.	NC_025141.1	Plasmid	Orlek_et_al.
Klebsiella sp.	NC_025142.1	Plasmid	Orlek_et_al.
Klebsiella sp.	NC_025143.1	Plasmid	Orlek_et_al.
Klebsiella sp.	NC_025144.1	Plasmid	Orlek_et_al.
Klebsiella sp.	NC_025145.1	Plasmid	Orlek_et_al.
Klebsiella sp.	NC_025147.1	Plasmid	Orlek_et_al.
Klebsiella sp.	NC_025148.1	Plasmid	Orlek_et_al.
Klebsiella sp.	NC_025149.1	Plasmid	Orlek_et_al.
Klebsiella sp.	NC_025150.1	Plasmid	Orlek_et_al.
Klebsiella sp.	NC_025166.1	Plasmid	Orlek_et_al.
Klebsiella sp.	NC_025167.1	Plasmid	Orlek_et_al.
Klebsiella sp.	NC_025175.1	Plasmid	Orlek_et_al.
Klebsiella sp.	NC_025176.1	Plasmid	Orlek_et_al.
Klebsiella sp.	NC_025177.1	Plasmid	Orlek_et_al.
Klebsiella sp.	NC_025178.1	Plasmid	Orlek_et_al.
Klebsiella sp.	NC_025179.1	Plasmid	Orlek_et_al.
Klebsiella sp.	NC_025180.1	Plasmid	Orlek_et_al.
Klebsiella sp.	NC_025181.1	Plasmid	Orlek_et_al.
Klebsiella sp.	NC_025182.1	Plasmid	Orlek_et_al.
Klebsiella sp.	NC_025183.1	Plasmid	Orlek_et_al.
Klebsiella sp.	NC_025184.1	Plasmid	Orlek_et_al.
Klebsiella sp.	NC_025186.1	Plasmid	Orlek_et_al.
Klebsiella sp.	NC_025187.1	Plasmid	Orlek_et_al.
Klebsiella sp.	NC_025190.1	Plasmid	Orlek_et_al.
Klebsiella sp.	NC_025198.1	Plasmid	Orlek_et_al.
Klebsiella sp.	NC_028464.1	Plasmid	Orlek_et_al.
Klebsiella sp.	NZ_AKKM01000049.1	Plasmid	Orlek_et_al.
Klebsiella sp.	NZ_AP010873.1	Plasmid	Orlek_et_al.
Klebsiella sp.	NZ_AP013064.1	Plasmid	Orlek_et_al.
Klebsiella sp.	NZ_AP013065.1	Plasmid	Orlek_et_al.
Klebsiella sp.	NZ_AP014566.1	Plasmid	Orlek_et_al.
Klebsiella sp.	NZ_AP014952.1	Plasmid	Orlek_et_al.
Klebsiella sp.	NZ_AP014953.1	Plasmid	Orlek_et_al.
Klebsiella sp.	NZ_AP014954.1	Plasmid	Orlek_et_al.
Klebsiella sp.	NZ_AP014955.1	Plasmid	Orlek_et_al.
Klebsiella sp.	NZ_CM001146.1	Plasmid	Orlek_et_al.
Klebsiella sp.	NZ_CM001850.1	Plasmid	Orlek_et_al.
Klebsiella sp.	NZ_CM001851.1	Plasmid	Orlek_et_al.
Klebsiella sp.	NZ_CM001853.1	Plasmid	Orlek_et_al.
Klebsiella sp.	NZ_CM001854.1	Plasmid	Orlek_et_al.
Klebsiella sp.	NZ_CM001855.1	Plasmid	Orlek_et_al.
Klebsiella sp.	NZ_CM001856.1	Plasmid	Orlek_et_al.
Klebsiella sp.	NZ_CM003247.1	Plasmid	Orlek_et_al.
Klebsiella sp.	NZ_CM003276.1	Plasmid	Orlek_et_al.
Klebsiella sp.	NZ_CM003365.1	Plasmid	Orlek_et_al.
Klebsiella sp.	NZ_CM003366.1	Plasmid	Orlek_et_al.
Klebsiella sp.	NZ_CM003367.1	Plasmid	Orlek_et_al.
Klebsiella sp.	NZ_CM003368.1	Plasmid	Orlek_et_al.
Klebsiella sp.	NZ_CM003470.1	Plasmid	Orlek_et_al.
Klebsiella sp.	NZ_CM003471.1	Plasmid	Orlek_et_al.
Klebsiella sp.	NZ_CM003649.1	Plasmid	Orlek_et_al.
Klebsiella sp.	NZ_CP002698.1	Plasmid	Orlek_et_al.
Klebsiella sp.	NZ_CP002700.1	Plasmid	Orlek_et_al.
Klebsiella sp.	NZ_CP002702.1	Plasmid	Orlek_et_al.
Klebsiella sp.	NZ_CP002704.1	Plasmid	Orlek_et_al.
Klebsiella sp.	NZ_CP003425.1	Plasmid	Orlek_et_al.
Klebsiella sp.	NZ_CP003994.1	Plasmid	Orlek_et_al.
Klebsiella sp.	NZ_CP003995.1	Plasmid	Orlek_et_al.
Klebsiella sp.	NZ_CP003996.1	Plasmid	Orlek_et_al.
Klebsiella sp.	NZ_CP003997.1	Plasmid	Orlek_et_al.
Klebsiella sp.	NZ_CP003998.1	Plasmid	Orlek_et_al.
Klebsiella sp.	NZ_CP004000.1	Plasmid	Orlek_et_al.
Klebsiella sp.	NZ_CP005931.1	Plasmid	Orlek_et_al.
Klebsiella sp.	NZ_CP005932.1	Plasmid	Orlek_et_al.
Klebsiella sp.	NZ_CP005994.1	Plasmid	Orlek_et_al.

Klebsiella sp.	NZ_CP005999.1	Plasmid	Orlek_et_al.
Klebsiella sp.	NZ_CP006000.1	Plasmid	Orlek_et_al.
Klebsiella sp.	NZ_CP006001.1	Plasmid	Orlek_et_al.
Klebsiella sp.	NZ_CP006002.1	Plasmid	Orlek_et_al.
Klebsiella sp.	NZ_CP006028.1	Plasmid	Orlek_et_al.
Klebsiella sp.	NZ_CP006029.1	Plasmid	Orlek_et_al.
Klebsiella sp.	NZ_CP006263.1	Plasmid	Orlek_et_al.
Klebsiella sp.	NZ_CP006264.1	Plasmid	Orlek_et_al.
Klebsiella sp.	NZ_CP006570.1	Plasmid	Orlek_et_al.
Klebsiella sp.	NZ_CP006633.1	Plasmid	Orlek_et_al.
Klebsiella sp.	NZ_CP006634.1	Plasmid	Orlek_et_al.
Klebsiella sp.	NZ_CP006635.1	Plasmid	Orlek_et_al.
Klebsiella sp.	NZ_CP006637.1	Plasmid	Orlek_et_al.
Klebsiella sp.	NZ_CP006638.1	Plasmid	Orlek_et_al.
Klebsiella sp.	NZ_CP006639.1	Plasmid	Orlek_et_al.
Klebsiella sp.	NZ_CP006640.1	Plasmid	Orlek_et_al.
Klebsiella sp.	NZ_CP006641.1	Plasmid	Orlek_et_al.
Klebsiella sp.	NZ_CP006642.1	Plasmid	Orlek_et_al.
Klebsiella sp.	NZ_CP006660.1	Plasmid	Orlek_et_al.
Klebsiella sp.	NZ_CP006661.1	Plasmid	Orlek_et_al.
Klebsiella sp.	NZ_CP006662.2	Plasmid	Orlek_et_al.
Klebsiella sp.	NZ_CP006663.1	Plasmid	Orlek_et_al.
Klebsiella sp.	NZ_CP006665.1	Plasmid	Orlek_et_al.
Klebsiella sp.	NZ_CP006666.1	Plasmid	Orlek_et_al.
Klebsiella sp.	NZ_CP006746.1	Plasmid	Orlek_et_al.
Klebsiella sp.	NZ_CP006747.1	Plasmid	Orlek_et_al.
Klebsiella sp.	NZ_CP006749.1	Plasmid	Orlek_et_al.
Klebsiella sp.	NZ_CP006750.1	Plasmid	Orlek_et_al.
Klebsiella sp.	NZ_CP006752.1	Plasmid	Orlek_et_al.
Klebsiella sp.	NZ_CP006753.1	Plasmid	Orlek_et_al.
Klebsiella sp.	NZ_CP006756.1	Plasmid	Orlek_et_al.
Klebsiella sp.	NZ_CP006757.1	Plasmid	Orlek_et_al.
Klebsiella sp.	NZ_CP006760.1	Plasmid	Orlek_et_al.
Klebsiella sp.	NZ_CP006761.1	Plasmid	Orlek_et_al.
Klebsiella sp.	NZ_CP006779.1	Plasmid	Orlek_et_al.
Klebsiella sp.	NZ_CP006780.1	Plasmid	Orlek_et_al.
Klebsiella sp.	NZ_CP006790.1	Plasmid	Orlek_et_al.
Klebsiella sp.	NZ_CP006791.1	Plasmid	Orlek_et_al.
Klebsiella sp.	NZ_CP006793.1	Plasmid	Orlek_et_al.
Klebsiella sp.	NZ_CP006795.1	Plasmid	Orlek_et_al.
Klebsiella sp.	NZ_CP006796.1	Plasmid	Orlek_et_al.
Klebsiella sp.	NZ_CP006797.1	Plasmid	Orlek_et_al.
Klebsiella sp.	NZ_CP006799.1	Plasmid	Orlek_et_al.
Klebsiella sp.	NZ_CP006800.1	Plasmid	Orlek_et_al.
Klebsiella sp.	NZ_CP006801.1	Plasmid	Orlek_et_al.
Klebsiella sp.	NZ_CP006807.1	Plasmid	Orlek_et_al.
Klebsiella sp.	NZ_CP006808.1	Plasmid	Orlek_et_al.
Klebsiella sp.	NZ_CP006919.1	Plasmid	Orlek_et_al.
Klebsiella sp.	NZ_CP006921.1	Plasmid	Orlek_et_al.
Klebsiella sp.	NZ_CP006922.1	Plasmid	Orlek_et_al.
Klebsiella sp.	NZ_CP006924.1	Plasmid	Orlek_et_al.
Klebsiella sp.	NZ_CP006925.1	Plasmid	Orlek_et_al.
Klebsiella sp.	NZ_CP006926.1	Plasmid	Orlek_et_al.
Klebsiella sp.	NZ_CP006927.1	Plasmid	Orlek_et_al.
Klebsiella sp.	NZ_CP006928.1	Plasmid	Orlek_et_al.
Klebsiella sp.	NZ_CP007038.1	Plasmid	Orlek_et_al.
Klebsiella sp.	NZ_CP007134.1	Plasmid	Orlek_et_al.
Klebsiella sp.	NZ_CP007135.1	Plasmid	Orlek_et_al.
Klebsiella sp.	NZ_CP007137.1	Plasmid	Orlek_et_al.
Klebsiella sp.	NZ_CP007150.1	Plasmid	Orlek_et_al.
Klebsiella sp.	NZ_CP007231.1	Plasmid	Orlek_et_al.
Klebsiella sp.	NZ_CP007488.1	Plasmid	Orlek_et_al.
Klebsiella sp.	NZ_CP007489.1	Plasmid	Orlek_et_al.
Klebsiella sp.	NZ_CP007508.1	Plasmid	Orlek_et_al.
Klebsiella sp.	NZ_CP007529.1	Plasmid	Orlek_et_al.
Klebsiella sp.	NZ_CP007558.1	Plasmid	Orlek_et_al.
Klebsiella sp.	NZ_CP007582.1	Plasmid	Orlek_et_al.
Klebsiella sp.	NZ_CP007599.1	Plasmid	Orlek_et_al.
Klebsiella sp.	NZ_CP007732.1	Plasmid	Orlek_et_al.
Klebsiella sp.	NZ_CP007733.1	Plasmid	Orlek_et_al.
Klebsiella sp.	NZ_CP007734.1	Plasmid	Orlek_et_al.
Klebsiella sp.	NZ_CP007735.1	Plasmid	Orlek_et_al.

Klebsiella sp.	NZ_CP007736.1	Plasmid	Orlek_et_al.
Klebsiella sp.	NZ_CP008715.1	Plasmid	Orlek_et_al.
Klebsiella sp.	NZ_CP008716.1	Plasmid	Orlek_et_al.
Klebsiella sp.	NZ_CP008717.1	Plasmid	Orlek_et_al.
Klebsiella sp.	NZ_CP008718.1	Plasmid	Orlek_et_al.
Klebsiella sp.	NZ_CP008719.1	Plasmid	Orlek_et_al.
Klebsiella sp.	NZ_CP008720.1	Plasmid	Orlek_et_al.
Klebsiella sp.	NZ_CP008721.1	Plasmid	Orlek_et_al.
Klebsiella sp.	NZ_CP008745.1	Plasmid	Orlek_et_al.
Klebsiella sp.	NZ_CP008789.1	Plasmid	Orlek_et_al.
Klebsiella sp.	NZ_CP008790.1	Plasmid	Orlek_et_al.
Klebsiella sp.	NZ_CP008791.1	Plasmid	Orlek_et_al.
Klebsiella sp.	NZ_CP008798.1	Plasmid	Orlek_et_al.
Klebsiella sp.	NZ_CP008799.1	Plasmid	Orlek_et_al.
Klebsiella sp.	NZ_CP008800.1	Plasmid	Orlek_et_al.
Klebsiella sp.	NZ_CP008806.1	Plasmid	Orlek_et_al.
Klebsiella sp.	NZ_CP008807.1	Plasmid	Orlek_et_al.
Klebsiella sp.	NZ_CP008824.1	Plasmid	Orlek_et_al.
Klebsiella sp.	NZ_CP008825.1	Plasmid	Orlek_et_al.
Klebsiella sp.	NZ_CP008826.1	Plasmid	Orlek_et_al.
Klebsiella sp.	NZ_CP008828.1	Plasmid	Orlek_et_al.
Klebsiella sp.	NZ_CP008829.1	Plasmid	Orlek_et_al.
Klebsiella sp.	NZ_CP008842.1	Plasmid	Orlek_et_al.
Klebsiella sp.	NZ_CP008843.1	Plasmid	Orlek_et_al.
Klebsiella sp.	NZ_CP008844.1	Plasmid	Orlek_et_al.
Klebsiella sp.	NZ_CP008845.1	Plasmid	Orlek_et_al.
Klebsiella sp.	NZ_CP008898.1	Plasmid	Orlek_et_al.
Klebsiella sp.	NZ_CP008899.1	Plasmid	Orlek_et_al.
Klebsiella sp.	NZ_CP008900.1	Plasmid	Orlek_et_al.
Klebsiella sp.	NZ_CP008906.1	Plasmid	Orlek_et_al.
Klebsiella sp.	NZ_CP008907.1	Plasmid	Orlek_et_al.
Klebsiella sp.	NZ_CP008919.1	Plasmid	Orlek_et_al.
Klebsiella sp.	NZ_CP008927.1	Plasmid	Orlek_et_al.
Klebsiella sp.	NZ_CP008930.1	Plasmid	Orlek_et_al.
Klebsiella sp.	NZ_CP008931.1	Plasmid	Orlek_et_al.
Klebsiella sp.	NZ_CP008932.1	Plasmid	Orlek_et_al.
Klebsiella sp.	NZ_CP008933.1	Plasmid	Orlek_et_al.
Klebsiella sp.	NZ_CP009103.1	Plasmid	Orlek_et_al.
Klebsiella sp.	NZ_CP009105.1	Plasmid	Orlek_et_al.
Klebsiella sp.	NZ_CP009107.1	Plasmid	Orlek_et_al.
Klebsiella sp.	NZ_CP009115.1	Plasmid	Orlek_et_al.
Klebsiella sp.	NZ_CP009116.1	Plasmid	Orlek_et_al.
Klebsiella sp.	NZ_CP009126.1	Plasmid	Orlek_et_al.
Klebsiella sp.	NZ_CP009167.1	Plasmid	Orlek_et_al.
Klebsiella sp.	NZ_CP009168.1	Plasmid	Orlek_et_al.
Klebsiella sp.	NZ_CP009169.1	Plasmid	Orlek_et_al.
Klebsiella sp.	NZ_CP009254.1	Plasmid	Orlek_et_al.
Klebsiella sp.	NZ_CP009255.1	Plasmid	Orlek_et_al.
Klebsiella sp.	NZ_CP009275.1	Plasmid	Orlek_et_al.
Klebsiella sp.	NZ_CP009276.1	Plasmid	Orlek_et_al.
Klebsiella sp.	NZ_CP009363.1	Plasmid	Orlek_et_al.
Klebsiella sp.	NZ_CP009366.1	Plasmid	Orlek_et_al.
Klebsiella sp.	NZ_CP009409.2	Plasmid	Orlek_et_al.
Klebsiella sp.	NZ_CP009457.1	Plasmid	Orlek_et_al.
Klebsiella sp.	NZ_CP009489.1	Plasmid	Orlek_et_al.
Klebsiella sp.	NZ_CP009490.1	Plasmid	Orlek_et_al.
Klebsiella sp.	NZ_CP009491.1	Plasmid	Orlek_et_al.
Klebsiella sp.	NZ_CP009560.1	Plasmid	Orlek_et_al.
Klebsiella sp.	NZ_CP009562.1	Plasmid	Orlek_et_al.
Klebsiella sp.	NZ_CP009563.1	Plasmid	Orlek_et_al.
Klebsiella sp.	NZ_CP009564.1	Plasmid	Orlek_et_al.
Klebsiella sp.	NZ_CP009566.1	Plasmid	Orlek_et_al.
Klebsiella sp.	NZ_CP009567.1	Plasmid	Orlek_et_al.
Klebsiella sp.	NZ_CP009568.1	Plasmid	Orlek_et_al.
Klebsiella sp.	NZ_CP009570.1	Plasmid	Orlek_et_al.
Klebsiella sp.	NZ_CP009579.1	Plasmid	Orlek_et_al.
Klebsiella sp.	NZ_CP009581.1	Plasmid	Orlek_et_al.
Klebsiella sp.	NZ_CP009582.1	Plasmid	Orlek_et_al.
Klebsiella sp.	NZ_CP009701.1	Plasmid	Orlek_et_al.
Klebsiella sp.	NZ_CP009702.1	Plasmid	Orlek_et_al.
Klebsiella sp.	NZ_CP009703.1	Plasmid	Orlek_et_al.
Klebsiella sp.	NZ_CP009710.1	Plasmid	Orlek_et_al.

Klebsiella sp.	NZ_CP009711.1	Plasmid	Orlek_et_al.
Klebsiella sp.	NZ_CP009713.1	Plasmid	Orlek_et_al.
Klebsiella sp.	NZ_CP009714.1	Plasmid	Orlek_et_al.
Klebsiella sp.	NZ_CP009721.1	Plasmid	Orlek_et_al.
Klebsiella sp.	NZ_CP009722.1	Plasmid	Orlek_et_al.
Klebsiella sp.	NZ_CP009724.1	Plasmid	Orlek_et_al.
Klebsiella sp.	NZ_CP009758.1	Plasmid	Orlek_et_al.
Klebsiella sp.	NZ_CP009767.1	Plasmid	Orlek_et_al.
Klebsiella sp.	NZ_CP009772.1	Plasmid	Orlek_et_al.
Klebsiella sp.	NZ_CP009773.1	Plasmid	Orlek_et_al.
Klebsiella sp.	NZ_CP009774.1	Plasmid	Orlek_et_al.
Klebsiella sp.	NZ_CP009776.1	Plasmid	Orlek_et_al.
Klebsiella sp.	NZ_CP009777.1	Plasmid	Orlek_et_al.
Klebsiella sp.	NZ_CP009778.1	Plasmid	Orlek_et_al.
Klebsiella sp.	NZ_CP009779.1	Plasmid	Orlek_et_al.
Klebsiella sp.	NZ_CP009783.1	Plasmid	Orlek_et_al.
Klebsiella sp.	NZ_CP009784.1	Plasmid	Orlek_et_al.
Klebsiella sp.	NZ_CP009834.1	Plasmid	Orlek_et_al.
Klebsiella sp.	NZ_CP009835.1	Plasmid	Orlek_et_al.
Klebsiella sp.	NZ_CP009837.1	Plasmid	Orlek_et_al.
Klebsiella sp.	NZ_CP009839.1	Plasmid	Orlek_et_al.
Klebsiella sp.	NZ_CP009841.1	Plasmid	Orlek_et_al.
Klebsiella sp.	NZ_CP009842.1	Plasmid	Orlek_et_al.
Klebsiella sp.	NZ_CP009843.1	Plasmid	Orlek_et_al.
Klebsiella sp.	NZ_CP009851.1	Plasmid	Orlek_et_al.
Klebsiella sp.	NZ_CP009852.1	Plasmid	Orlek_et_al.
Klebsiella sp.	NZ_CP009853.1	Plasmid	Orlek_et_al.
Klebsiella sp.	NZ_CP009855.1	Plasmid	Orlek_et_al.
Klebsiella sp.	NZ_CP009856.1	Plasmid	Orlek_et_al.
Klebsiella sp.	NZ_CP009857.1	Plasmid	Orlek_et_al.
Klebsiella sp.	NZ_CP009858.1	Plasmid	Orlek_et_al.
Klebsiella sp.	NZ_CP009860.1	Plasmid	Orlek_et_al.
Klebsiella sp.	NZ_CP009861.1	Plasmid	Orlek_et_al.
Klebsiella sp.	NZ_CP009862.1	Plasmid	Orlek_et_al.
Klebsiella sp.	NZ_CP009864.1	Plasmid	Orlek_et_al.
Klebsiella sp.	NZ_CP009865.1	Plasmid	Orlek_et_al.
Klebsiella sp.	NZ_CP009867.1	Plasmid	Orlek_et_al.
Klebsiella sp.	NZ_CP009868.1	Plasmid	Orlek_et_al.
Klebsiella sp.	NZ_CP009869.1	Plasmid	Orlek_et_al.
Klebsiella sp.	NZ_CP009870.1	Plasmid	Orlek_et_al.
Klebsiella sp.	NZ_CP009871.1	Plasmid	Orlek_et_al.
Klebsiella sp.	NZ_CP009873.1	Plasmid	Orlek_et_al.
Klebsiella sp.	NZ_CP009874.1	Plasmid	Orlek_et_al.
Klebsiella sp.	NZ_CP009875.1	Plasmid	Orlek_et_al.
Klebsiella sp.	NZ_CP009877.1	Plasmid	Orlek_et_al.
Klebsiella sp.	NZ_CP009878.1	Plasmid	Orlek_et_al.
Klebsiella sp.	NZ_CP009879.1	Plasmid	Orlek_et_al.
Klebsiella sp.	NZ_CP009881.1	Plasmid	Orlek_et_al.
Klebsiella sp.	NZ_CP009882.1	Plasmid	Orlek_et_al.
Klebsiella sp.	NZ_CP009883.1	Plasmid	Orlek_et_al.
Klebsiella sp.	NZ_CP009884.1	Plasmid	Orlek_et_al.
Klebsiella sp.	NZ_CP009903.1	Plasmid	Orlek_et_al.
Klebsiella sp.	NZ_CP009904.1	Plasmid	Orlek_et_al.
Klebsiella sp.	NZ_CP009905.1	Plasmid	Orlek_et_al.
Klebsiella sp.	NZ_CP009934.1	Plasmid	Orlek_et_al.
Klebsiella sp.	NZ_CP009936.1	Plasmid	Orlek_et_al.
Klebsiella sp.	NZ_CP009937.1	Plasmid	Orlek_et_al.
Klebsiella sp.	NZ_CP009971.1	Plasmid	Orlek_et_al.
Klebsiella sp.	NZ_CP009972.1	Plasmid	Orlek_et_al.
Klebsiella sp.	NZ_CP009988.1	Plasmid	Orlek_et_al.
Klebsiella sp.	NZ_CP009989.1	Plasmid	Orlek_et_al.
Klebsiella sp.	NZ_CP009990.1	Plasmid	Orlek_et_al.
Klebsiella sp.	NZ_CP009993.1	Plasmid	Orlek_et_al.
Klebsiella sp.	NZ_CP009994.1	Plasmid	Orlek_et_al.
Klebsiella sp.	NZ_CP009995.1	Plasmid	Orlek_et_al.
Klebsiella sp.	NZ_CP010020.1	Plasmid	Orlek_et_al.
Klebsiella sp.	NZ_CP010021.1	Plasmid	Orlek_et_al.
Klebsiella sp.	NZ_CP010022.1	Plasmid	Orlek_et_al.
Klebsiella sp.	NZ_CP010068.1	Plasmid	Orlek_et_al.
Klebsiella sp.	NZ_CP010069.1	Plasmid	Orlek_et_al.
Klebsiella sp.	NZ_CP010246.1	Plasmid	Orlek_et_al.
Klebsiella sp.	NZ_CP010294.1	Plasmid	Orlek_et_al.

Klebsiella sp.	NZ_CP010305.1	Plasmid	Orlek_et_al.
Klebsiella sp.	NZ_CP010316.1	Plasmid	Orlek_et_al.
Klebsiella sp.	NZ_CP010317.1	Plasmid	Orlek_et_al.
Klebsiella sp.	NZ_CP010318.1	Plasmid	Orlek_et_al.
Klebsiella sp.	NZ_CP010325.1	Plasmid	Orlek_et_al.
Klebsiella sp.	NZ_CP010326.1	Plasmid	Orlek_et_al.
Klebsiella sp.	NZ_CP010360.2	Plasmid	Orlek_et_al.
Klebsiella sp.	NZ_CP010362.1	Plasmid	Orlek_et_al.
Klebsiella sp.	NZ_CP010363.1	Plasmid	Orlek_et_al.
Klebsiella sp.	NZ_CP010364.1	Plasmid	Orlek_et_al.
Klebsiella sp.	NZ_CP010365.1	Plasmid	Orlek_et_al.
Klebsiella sp.	NZ_CP010366.2	Plasmid	Orlek_et_al.
Klebsiella sp.	NZ_CP010367.1	Plasmid	Orlek_et_al.
Klebsiella sp.	NZ_CP010372.1	Plasmid	Orlek_et_al.
Klebsiella sp.	NZ_CP010373.2	Plasmid	Orlek_et_al.
Klebsiella sp.	NZ_CP010374.1	Plasmid	Orlek_et_al.
Klebsiella sp.	NZ_CP010375.2	Plasmid	Orlek_et_al.
Klebsiella sp.	NZ_CP010378.1	Plasmid	Orlek_et_al.
Klebsiella sp.	NZ_CP010379.1	Plasmid	Orlek_et_al.
Klebsiella sp.	NZ_CP010380.1	Plasmid	Orlek_et_al.
Klebsiella sp.	NZ_CP010381.1	Plasmid	Orlek_et_al.
Klebsiella sp.	NZ_CP010382.1	Plasmid	Orlek_et_al.
Klebsiella sp.	NZ_CP010383.2	Plasmid	Orlek_et_al.
Klebsiella sp.	NZ_CP010385.1	Plasmid	Orlek_et_al.
Klebsiella sp.	NZ_CP010386.1	Plasmid	Orlek_et_al.
Klebsiella sp.	NZ_CP010387.1	Plasmid	Orlek_et_al.
Klebsiella sp.	NZ_CP010388.1	Plasmid	Orlek_et_al.
Klebsiella sp.	NZ_CP010389.1	Plasmid	Orlek_et_al.
Klebsiella sp.	NZ_CP010390.1	Plasmid	Orlek_et_al.
Klebsiella sp.	NZ_CP010391.1	Plasmid	Orlek_et_al.
Klebsiella sp.	NZ_CP010393.1	Plasmid	Orlek_et_al.
Klebsiella sp.	NZ_CP010394.1	Plasmid	Orlek_et_al.
Klebsiella sp.	NZ_CP010395.1	Plasmid	Orlek_et_al.
Klebsiella sp.	NZ_CP010396.1	Plasmid	Orlek_et_al.
Klebsiella sp.	NZ_CP010830.1	Plasmid	Orlek_et_al.
Klebsiella sp.	NZ_CP010831.1	Plasmid	Orlek_et_al.
Klebsiella sp.	NZ_CP010832.1	Plasmid	Orlek_et_al.
Klebsiella sp.	NZ_CP010877.1	Plasmid	Orlek_et_al.
Klebsiella sp.	NZ_CP010878.1	Plasmid	Orlek_et_al.
Klebsiella sp.	NZ_CP010879.1	Plasmid	Orlek_et_al.
Klebsiella sp.	NZ_CP010880.1	Plasmid	Orlek_et_al.
Klebsiella sp.	NZ_CP010881.1	Plasmid	Orlek_et_al.
Klebsiella sp.	NZ_CP010882.1	Plasmid	Orlek_et_al.
Klebsiella sp.	NZ_CP010908.1	Plasmid	Orlek_et_al.
Klebsiella sp.	NZ_CP010909.1	Plasmid	Orlek_et_al.
Klebsiella sp.	NZ_CP010910.1	Plasmid	Orlek_et_al.
Klebsiella sp.	NZ_CP010911.1	Plasmid	Orlek_et_al.
Klebsiella sp.	NZ_CP011019.1	Plasmid	Orlek_et_al.
Klebsiella sp.	NZ_CP011048.1	Plasmid	Orlek_et_al.
Klebsiella sp.	NZ_CP011049.1	Plasmid	Orlek_et_al.
Klebsiella sp.	NZ_CP011050.1	Plasmid	Orlek_et_al.
Klebsiella sp.	NZ_CP011062.1	Plasmid	Orlek_et_al.
Klebsiella sp.	NZ_CP011063.1	Plasmid	Orlek_et_al.
Klebsiella sp.	NZ_CP011064.1	Plasmid	Orlek_et_al.
Klebsiella sp.	NZ_CP011065.1	Plasmid	Orlek_et_al.
Klebsiella sp.	NZ_CP011066.1	Plasmid	Orlek_et_al.
Klebsiella sp.	NZ_CP011067.1	Plasmid	Orlek_et_al.
Klebsiella sp.	NZ_CP011133.1	Plasmid	Orlek_et_al.
Klebsiella sp.	NZ_CP011135.1	Plasmid	Orlek_et_al.
Klebsiella sp.	NZ_CP011136.1	Plasmid	Orlek_et_al.
Klebsiella sp.	NZ_CP011137.1	Plasmid	Orlek_et_al.
Klebsiella sp.	NZ_CP011138.1	Plasmid	Orlek_et_al.
Klebsiella sp.	NZ_CP011139.1	Plasmid	Orlek_et_al.
Klebsiella sp.	NZ_CP011140.1	Plasmid	Orlek_et_al.
Klebsiella sp.	NZ_CP011141.1	Plasmid	Orlek_et_al.
Klebsiella sp.	NZ_CP011142.1	Plasmid	Orlek_et_al.
Klebsiella sp.	NZ_CP011143.1	Plasmid	Orlek_et_al.
Klebsiella sp.	NZ_CP011287.1	Plasmid	Orlek_et_al.
Klebsiella sp.	NZ_CP011290.1	Plasmid	Orlek_et_al.
Klebsiella sp.	NZ_CP011291.1	Plasmid	Orlek_et_al.
Klebsiella sp.	NZ_CP011294.1	Plasmid	Orlek_et_al.
Klebsiella sp.	NZ_CP011314.1	Plasmid	Orlek_et_al.

Klebsiella sp.	NZ_CP011315.1	Plasmid	Orlek_et_al.
Klebsiella sp.	NZ_CP011316.1	Plasmid	Orlek_et_al.
Klebsiella sp.	NZ_CP011332.1	Plasmid	Orlek_et_al.
Klebsiella sp.	NZ_CP011333.1	Plasmid	Orlek_et_al.
Klebsiella sp.	NZ_CP011334.1	Plasmid	Orlek_et_al.
Klebsiella sp.	NZ_CP011335.1	Plasmid	Orlek_et_al.
Klebsiella sp.	NZ_CP011336.1	Plasmid	Orlek_et_al.
Klebsiella sp.	NZ_CP011337.1	Plasmid	Orlek_et_al.
Klebsiella sp.	NZ_CP011338.1	Plasmid	Orlek_et_al.
Klebsiella sp.	NZ_CP011395.1	Plasmid	Orlek_et_al.
Klebsiella sp.	NZ_CP011417.1	Plasmid	Orlek_et_al.
Klebsiella sp.	NZ_CP011418.1	Plasmid	Orlek_et_al.
Klebsiella sp.	NZ_CP011429.1	Plasmid	Orlek_et_al.
Klebsiella sp.	NZ_CP011430.1	Plasmid	Orlek_et_al.
Klebsiella sp.	NZ_CP011431.1	Plasmid	Orlek_et_al.
Klebsiella sp.	NZ_CP011432.1	Plasmid	Orlek_et_al.
Klebsiella sp.	NZ_CP011433.1	Plasmid	Orlek_et_al.
Klebsiella sp.	NZ_CP011434.1	Plasmid	Orlek_et_al.
Klebsiella sp.	NZ_CP011435.1	Plasmid	Orlek_et_al.
Klebsiella sp.	NZ_CP011493.1	Plasmid	Orlek_et_al.
Klebsiella sp.	NZ_CP011496.1	Plasmid	Orlek_et_al.
Klebsiella sp.	NZ_CP011540.1	Plasmid	Orlek_et_al.
Klebsiella sp.	NZ_CP011569.1	Plasmid	Orlek_et_al.
Klebsiella sp.	NZ_CP011571.1	Plasmid	Orlek_et_al.
Klebsiella sp.	NZ_CP011573.1	Plasmid	Orlek_et_al.
Klebsiella sp.	NZ_CP011575.1	Plasmid	Orlek_et_al.
Klebsiella sp.	NZ_CP011576.1	Plasmid	Orlek_et_al.
Klebsiella sp.	NZ_CP011577.1	Plasmid	Orlek_et_al.
Klebsiella sp.	NZ_CP011579.1	Plasmid	Orlek_et_al.
Klebsiella sp.	NZ_CP011580.1	Plasmid	Orlek_et_al.
Klebsiella sp.	NZ_CP011582.1	Plasmid	Orlek_et_al.
Klebsiella sp.	NZ_CP011583.1	Plasmid	Orlek_et_al.
Klebsiella sp.	NZ_CP011585.1	Plasmid	Orlek_et_al.
Klebsiella sp.	NZ_CP011586.1	Plasmid	Orlek_et_al.
Klebsiella sp.	NZ_CP011587.1	Plasmid	Orlek_et_al.
Klebsiella sp.	NZ_CP011588.1	Plasmid	Orlek_et_al.
Klebsiella sp.	NZ_CP011589.1	Plasmid	Orlek_et_al.
Klebsiella sp.	NZ_CP011590.1	Plasmid	Orlek_et_al.
Klebsiella sp.	NZ_CP011592.1	Plasmid	Orlek_et_al.
Klebsiella sp.	NZ_CP011593.1	Plasmid	Orlek_et_al.
Klebsiella sp.	NZ_CP011594.1	Plasmid	Orlek_et_al.
Klebsiella sp.	NZ_CP011595.1	Plasmid	Orlek_et_al.
Klebsiella sp.	NZ_CP011596.1	Plasmid	Orlek_et_al.
Klebsiella sp.	NZ_CP011598.1	Plasmid	Orlek_et_al.
Klebsiella sp.	NZ_CP011599.1	Plasmid	Orlek_et_al.
Klebsiella sp.	NZ_CP011600.1	Plasmid	Orlek_et_al.
Klebsiella sp.	NZ_CP011601.1	Plasmid	Orlek_et_al.
Klebsiella sp.	NZ_CP011603.1	Plasmid	Orlek_et_al.
Klebsiella sp.	NZ_CP011604.1	Plasmid	Orlek_et_al.
Klebsiella sp.	NZ_CP011605.1	Plasmid	Orlek_et_al.
Klebsiella sp.	NZ_CP011606.1	Plasmid	Orlek_et_al.
Klebsiella sp.	NZ_CP011607.1	Plasmid	Orlek_et_al.
Klebsiella sp.	NZ_CP011608.1	Plasmid	Orlek_et_al.
Klebsiella sp.	NZ_CP011609.1	Plasmid	Orlek_et_al.
Klebsiella sp.	NZ_CP011610.1	Plasmid	Orlek_et_al.
Klebsiella sp.	NZ_CP011611.1	Plasmid	Orlek_et_al.
Klebsiella sp.	NZ_CP011613.1	Plasmid	Orlek_et_al.
Klebsiella sp.	NZ_CP011614.1	Plasmid	Orlek_et_al.
Klebsiella sp.	NZ_CP011616.1	Plasmid	Orlek_et_al.
Klebsiella sp.	NZ_CP011617.1	Plasmid	Orlek_et_al.
Klebsiella sp.	NZ_CP011620.1	Plasmid	Orlek_et_al.
Klebsiella sp.	NZ_CP011622.1	Plasmid	Orlek_et_al.
Klebsiella sp.	NZ_CP011623.1	Plasmid	Orlek_et_al.
Klebsiella sp.	NZ_CP011625.1	Plasmid	Orlek_et_al.
Klebsiella sp.	NZ_CP011626.1	Plasmid	Orlek_et_al.
Klebsiella sp.	NZ_CP011627.1	Plasmid	Orlek_et_al.
Klebsiella sp.	NZ_CP011628.1	Plasmid	Orlek_et_al.
Klebsiella sp.	NZ_CP011630.1	Plasmid	Orlek_et_al.
Klebsiella sp.	NZ_CP011631.1	Plasmid	Orlek_et_al.
Klebsiella sp.	NZ_CP011632.1	Plasmid	Orlek_et_al.
Klebsiella sp.	NZ_CP011633.1	Plasmid	Orlek_et_al.
Klebsiella sp.	NZ_CP011634.1	Plasmid	Orlek_et_al.

Klebsiella sp.	NZ_CP011635.1	Plasmid	Orlek_et_al.
Klebsiella sp.	NZ_CP011637.1	Plasmid	Orlek_et_al.
Klebsiella sp.	NZ_CP011638.1	Plasmid	Orlek_et_al.
Klebsiella sp.	NZ_CP011639.1	Plasmid	Orlek_et_al.
Klebsiella sp.	NZ_CP011640.1	Plasmid	Orlek_et_al.
Klebsiella sp.	NZ_CP011641.1	Plasmid	Orlek_et_al.
Klebsiella sp.	NZ_CP011643.1	Plasmid	Orlek_et_al.
Klebsiella sp.	NZ_CP011644.1	Plasmid	Orlek_et_al.
Klebsiella sp.	NZ_CP011645.1	Plasmid	Orlek_et_al.
Klebsiella sp.	NZ_CP011646.1	Plasmid	Orlek_et_al.
Klebsiella sp.	NZ_CP011649.1	Plasmid	Orlek_et_al.
Klebsiella sp.	NZ_CP011651.1	Plasmid	Orlek_et_al.
Klebsiella sp.	NZ_CP011652.1	Plasmid	Orlek_et_al.
Klebsiella sp.	NZ_CP011653.1	Plasmid	Orlek_et_al.
Klebsiella sp.	NZ_CP011654.1	Plasmid	Orlek_et_al.
Klebsiella sp.	NZ_CP011655.1	Plasmid	Orlek_et_al.
Klebsiella sp.	NZ_CP011656.1	Plasmid	Orlek_et_al.
Klebsiella sp.	NZ_CP011864.1	Plasmid	Orlek_et_al.
Klebsiella sp.	NZ_CP011865.1	Plasmid	Orlek_et_al.
Klebsiella sp.	NZ_CP011866.1	Plasmid	Orlek_et_al.
Klebsiella sp.	NZ_CP011867.1	Plasmid	Orlek_et_al.
Klebsiella sp.	NZ_CP011943.1	Plasmid	Orlek_et_al.
Klebsiella sp.	NZ_CP011944.1	Plasmid	Orlek_et_al.
Klebsiella sp.	NZ_CP011945.1	Plasmid	Orlek_et_al.
Klebsiella sp.	NZ_CP011977.1	Plasmid	Orlek_et_al.
Klebsiella sp.	NZ_CP011978.1	Plasmid	Orlek_et_al.
Klebsiella sp.	NZ_CP011979.1	Plasmid	Orlek_et_al.
Klebsiella sp.	NZ_CP011981.1	Plasmid	Orlek_et_al.
Klebsiella sp.	NZ_CP011982.1	Plasmid	Orlek_et_al.
Klebsiella sp.	NZ_CP011983.1	Plasmid	Orlek_et_al.
Klebsiella sp.	NZ_CP011984.1	Plasmid	Orlek_et_al.
Klebsiella sp.	NZ_CP011986.1	Plasmid	Orlek_et_al.
Klebsiella sp.	NZ_CP011987.1	Plasmid	Orlek_et_al.
Klebsiella sp.	NZ_CP011988.1	Plasmid	Orlek_et_al.
Klebsiella sp.	NZ_CP011990.1	Plasmid	Orlek_et_al.
Klebsiella sp.	NZ_CP011991.1	Plasmid	Orlek_et_al.
Klebsiella sp.	NZ_CP011993.1	Plasmid	Orlek_et_al.
Klebsiella sp.	NZ_CP012039.1	Plasmid	Orlek_et_al.
Klebsiella sp.	NZ_CP012138.1	Plasmid	Orlek_et_al.
Klebsiella sp.	NZ_CP012139.1	Plasmid	Orlek_et_al.
Klebsiella sp.	NZ_CP012141.1	Plasmid	Orlek_et_al.
Klebsiella sp.	NZ_CP012142.1	Plasmid	Orlek_et_al.
Klebsiella sp.	NZ_CP012143.1	Plasmid	Orlek_et_al.
Klebsiella sp.	NZ_CP012163.1	Plasmid	Orlek_et_al.
Klebsiella sp.	NZ_CP012164.1	Plasmid	Orlek_et_al.
Klebsiella sp.	NZ_CP012166.1	Plasmid	Orlek_et_al.
Klebsiella sp.	NZ_CP012168.1	Plasmid	Orlek_et_al.
Klebsiella sp.	NZ_CP012169.1	Plasmid	Orlek_et_al.
Klebsiella sp.	NZ_CP012170.1	Plasmid	Orlek_et_al.
Klebsiella sp.	NZ_CP012254.1	Plasmid	Orlek_et_al.
Klebsiella sp.	NZ_CP012255.1	Plasmid	Orlek_et_al.
Klebsiella sp.	NZ_CP012256.1	Plasmid	Orlek_et_al.
Klebsiella sp.	NZ_CP012258.1	Plasmid	Orlek_et_al.
Klebsiella sp.	NZ_CP012265.1	Plasmid	Orlek_et_al.
Klebsiella sp.	NZ_CP012267.1	Plasmid	Orlek_et_al.
Klebsiella sp.	NZ_CP012345.1	Plasmid	Orlek_et_al.
Klebsiella sp.	NZ_CP012348.1	Plasmid	Orlek_et_al.
Klebsiella sp.	NZ_CP012397.1	Plasmid	Orlek_et_al.
Klebsiella sp.	NZ_CP012488.1	Plasmid	Orlek_et_al.
Klebsiella sp.	NZ_CP012489.1	Plasmid	Orlek_et_al.
Klebsiella sp.	NZ_CP012490.1	Plasmid	Orlek_et_al.
Klebsiella sp.	NZ_CP012498.1	Plasmid	Orlek_et_al.
Klebsiella sp.	NZ_CP012499.1	Plasmid	Orlek_et_al.
Klebsiella sp.	NZ_CP012500.1	Plasmid	Orlek_et_al.
Klebsiella sp.	NZ_CP012501.1	Plasmid	Orlek_et_al.
Klebsiella sp.	NZ_CP012626.1	Plasmid	Orlek_et_al.
Klebsiella sp.	NZ_CP012627.1	Plasmid	Orlek_et_al.
Klebsiella sp.	NZ_CP012628.1	Plasmid	Orlek_et_al.
Klebsiella sp.	NZ_CP012629.1	Plasmid	Orlek_et_al.
Klebsiella sp.	NZ_CP012630.1	Plasmid	Orlek_et_al.
Klebsiella sp.	NZ_CP012632.1	Plasmid	Orlek_et_al.
Klebsiella sp.	NZ_CP012634.1	Plasmid	Orlek_et_al.

Klebsiella sp.	NZ_CP012636.1	Plasmid	Orlek_et_al.
Klebsiella sp.	NZ_CP012637.1	Plasmid	Orlek_et_al.
Klebsiella sp.	NZ_CP012638.1	Plasmid	Orlek_et_al.
Klebsiella sp.	NZ_CP012682.1	Plasmid	Orlek_et_al.
Klebsiella sp.	NZ_CP012683.1	Plasmid	Orlek_et_al.
Klebsiella sp.	NZ_CP012684.1	Plasmid	Orlek_et_al.
Klebsiella sp.	NZ_CP012686.1	Plasmid	Orlek_et_al.
Klebsiella sp.	NZ_CP012732.1	Plasmid	Orlek_et_al.
Klebsiella sp.	NZ_CP012733.1	Plasmid	Orlek_et_al.
Klebsiella sp.	NZ_CP012734.1	Plasmid	Orlek_et_al.
Klebsiella sp.	NZ_CP012736.1	Plasmid	Orlek_et_al.
Klebsiella sp.	NZ_CP012754.1	Plasmid	Orlek_et_al.
Klebsiella sp.	NZ_CP012755.1	Plasmid	Orlek_et_al.
Klebsiella sp.	NZ_CP012803.1	Plasmid	Orlek_et_al.
Klebsiella sp.	NZ_CP012834.1	Plasmid	Orlek_et_al.
Klebsiella sp.	NZ_CP012835.1	Plasmid	Orlek_et_al.
Klebsiella sp.	NZ_CP012884.1	Plasmid	Orlek_et_al.
Klebsiella sp.	NZ_CP012923.1	Plasmid	Orlek_et_al.
Klebsiella sp.	NZ_CP012926.1	Plasmid	Orlek_et_al.
Klebsiella sp.	NZ_CP012928.1	Plasmid	Orlek_et_al.
Klebsiella sp.	NZ_CP012929.1	Plasmid	Orlek_et_al.
Klebsiella sp.	NZ_CP012931.1	Plasmid	Orlek_et_al.
Klebsiella sp.	NZ_CP012932.1	Plasmid	Orlek_et_al.
Klebsiella sp.	NZ_CP012933.1	Plasmid	Orlek_et_al.
Klebsiella sp.	NZ_CP012934.1	Plasmid	Orlek_et_al.
Klebsiella sp.	NZ_CP012935.1	Plasmid	Orlek_et_al.
Klebsiella sp.	NZ_CP012936.1	Plasmid	Orlek_et_al.
Klebsiella sp.	NZ_CP012988.3	Plasmid	Orlek_et_al.
Klebsiella sp.	NZ_CP012989.1	Plasmid	Orlek_et_al.
Klebsiella sp.	NZ_CP012990.1	Plasmid	Orlek_et_al.
Klebsiella sp.	NZ_CP012993.2	Plasmid	Orlek_et_al.
Klebsiella sp.	NZ_CP012994.1	Plasmid	Orlek_et_al.
Klebsiella sp.	NZ_CP012995.1	Plasmid	Orlek_et_al.
Klebsiella sp.	NZ_CP013028.1	Plasmid	Orlek_et_al.
Klebsiella sp.	NZ_CP013030.1	Plasmid	Orlek_et_al.
Klebsiella sp.	NZ_CP013047.1	Plasmid	Orlek_et_al.
Klebsiella sp.	NZ_CP013098.1	Plasmid	Orlek_et_al.
Klebsiella sp.	NZ_CP013221.1	Plasmid	Orlek_et_al.
Klebsiella sp.	NZ_CP013223.1	Plasmid	Orlek_et_al.
Klebsiella sp.	NZ_CP013224.1	Plasmid	Orlek_et_al.
Klebsiella sp.	NZ_CP013225.1	Plasmid	Orlek_et_al.
Klebsiella sp.	NZ_CP013321.1	Plasmid	Orlek_et_al.
Klebsiella sp.	NZ_CP013323.1	Plasmid	Orlek_et_al.
Klebsiella sp.	NZ_CP013324.1	Plasmid	Orlek_et_al.
Klebsiella sp.	NZ_CP013325.1	Plasmid	Orlek_et_al.
Klebsiella sp.	NZ_CP013326.1	Plasmid	Orlek_et_al.
Klebsiella sp.	NZ_CP013339.1	Plasmid	Orlek_et_al.
Klebsiella sp.	NZ_CP013340.1	Plasmid	Orlek_et_al.
Klebsiella sp.	NZ_CP013712.1	Plasmid	Orlek_et_al.
Klebsiella sp.	NZ_CP013713.1	Plasmid	Orlek_et_al.
Klebsiella sp.	NZ_CP013832.1	Plasmid	Orlek_et_al.
Klebsiella sp.	NZ_CP013833.1	Plasmid	Orlek_et_al.
Klebsiella sp.	NZ_CP013834.1	Plasmid	Orlek_et_al.
Klebsiella sp.	NZ_CP013914.1	Plasmid	Orlek_et_al.
Klebsiella sp.	NZ_CP013915.1	Plasmid	Orlek_et_al.
Klebsiella sp.	NZ_CP013941.1	Plasmid	Orlek_et_al.
Klebsiella sp.	NZ_CP013942.1	Plasmid	Orlek_et_al.
Klebsiella sp.	NZ_CP014005.1	Plasmid	Orlek_et_al.
Klebsiella sp.	NZ_CP014006.1	Plasmid	Orlek_et_al.
Klebsiella sp.	NZ_CP014009.1	Plasmid	Orlek_et_al.
Klebsiella sp.	NZ_CP014011.1	Plasmid	Orlek_et_al.
Klebsiella sp.	NZ_CP014050.1	Plasmid	Orlek_et_al.
Klebsiella sp.	NZ_CP014096.1	Plasmid	Orlek_et_al.
Klebsiella sp.	NZ_CP014098.1	Plasmid	Orlek_et_al.
Klebsiella sp.	NZ_CP014100.1	Plasmid	Orlek_et_al.
Klebsiella sp.	NZ_CP014101.1	Plasmid	Orlek_et_al.
Klebsiella sp.	NZ_CP014126.1	Plasmid	Orlek_et_al.
Klebsiella sp.	NZ_CP014127.1	Plasmid	Orlek_et_al.
Klebsiella sp.	NZ_CP014128.1	Plasmid	Orlek_et_al.
Klebsiella sp.	NZ_CP014198.2	Plasmid	Orlek_et_al.
Klebsiella sp.	NZ_CP014199.1	Plasmid	Orlek_et_al.
Klebsiella sp.	NZ_CP014200.1	Plasmid	Orlek_et_al.

Klebsiella sp.	NZ_CP014208.1	Plasmid	Orlek_et_al.
Klebsiella sp.	NZ_CP014271.1	Plasmid	Orlek_et_al.
Klebsiella sp.	NZ_CP014273.1	Plasmid	Orlek_et_al.
Klebsiella sp.	NZ_CP014305.2	Plasmid	Orlek_et_al.
Klebsiella sp.	NZ_CP014357.1	Plasmid	Orlek_et_al.
Klebsiella sp.	NZ_CP014359.1	Plasmid	Orlek_et_al.
Klebsiella sp.	NZ_CP014489.1	Plasmid	Orlek_et_al.
Klebsiella sp.	NZ_CP014490.1	Plasmid	Orlek_et_al.
Klebsiella sp.	NZ_CP014491.1	Plasmid	Orlek_et_al.
Klebsiella sp.	NZ_CP014493.1	Plasmid	Orlek_et_al.
Klebsiella sp.	NZ_CP014494.1	Plasmid	Orlek_et_al.
Klebsiella sp.	NZ_CP014496.1	Plasmid	Orlek_et_al.
Klebsiella sp.	NZ_CP014498.1	Plasmid	Orlek_et_al.
Klebsiella sp.	NZ_CP014523.1	Plasmid	Orlek_et_al.
Klebsiella sp.	NZ_CP014524.1	Plasmid	Orlek_et_al.
Klebsiella sp.	NZ_CP014537.1	Plasmid	Orlek_et_al.
Klebsiella sp.	NZ_CP014577.1	Plasmid	Orlek_et_al.
Klebsiella sp.	NZ_CP014622.1	Plasmid	Orlek_et_al.
Klebsiella sp.	NZ_CP014648.1	Plasmid	Orlek_et_al.
Klebsiella sp.	NZ_CP014649.1	Plasmid	Orlek_et_al.
Klebsiella sp.	NZ_CP014650.1	Plasmid	Orlek_et_al.
Klebsiella sp.	NZ_CP014658.1	Plasmid	Orlek_et_al.
Klebsiella sp.	NZ_CP014660.1	Plasmid	Orlek_et_al.
Klebsiella sp.	NZ_CP014662.1	Plasmid	Orlek_et_al.
Klebsiella sp.	NZ_CP014697.1	Plasmid	Orlek_et_al.
Klebsiella sp.	NZ_CP014698.1	Plasmid	Orlek_et_al.
Klebsiella sp.	NZ_CP014707.1	Plasmid	Orlek_et_al.
Klebsiella sp.	NZ_CP014756.1	Plasmid	Orlek_et_al.
Klebsiella sp.	NZ_CP014757.1	Plasmid	Orlek_et_al.
Klebsiella sp.	NZ_CP014758.1	Plasmid	Orlek_et_al.
Klebsiella sp.	NZ_CP014763.1	Plasmid	Orlek_et_al.
Klebsiella sp.	NZ_CP014764.1	Plasmid	Orlek_et_al.
Klebsiella sp.	NZ_CP014765.1	Plasmid	Orlek_et_al.
Klebsiella sp.	NZ_CP014966.1	Plasmid	Orlek_et_al.
Klebsiella sp.	NZ_CP014968.1	Plasmid	Orlek_et_al.
Klebsiella sp.	NZ_CP014970.1	Plasmid	Orlek_et_al.
Klebsiella sp.	NZ_CP014972.1	Plasmid	Orlek_et_al.
Klebsiella sp.	NZ_CP014973.1	Plasmid	Orlek_et_al.
Klebsiella sp.	NZ_CP014974.1	Plasmid	Orlek_et_al.
Klebsiella sp.	NZ_CP014976.1	Plasmid	Orlek_et_al.
Klebsiella sp.	NZ_CP014978.1	Plasmid	Orlek_et_al.
Klebsiella sp.	NZ_CP014980.1	Plasmid	Orlek_et_al.
Klebsiella sp.	NZ_CP014995.1	Plasmid	Orlek_et_al.
Klebsiella sp.	NZ_CP014997.1	Plasmid	Orlek_et_al.
Klebsiella sp.	NZ_CP015021.1	Plasmid	Orlek_et_al.
Klebsiella sp.	NZ_CP015022.1	Plasmid	Orlek_et_al.
Klebsiella sp.	NZ_CP015026.1	Plasmid	Orlek_et_al.
Klebsiella sp.	NZ_CP015070.1	Plasmid	Orlek_et_al.
Klebsiella sp.	NZ_CP015071.1	Plasmid	Orlek_et_al.
Klebsiella sp.	NZ_CP015072.1	Plasmid	Orlek_et_al.
Klebsiella sp.	NZ_CP015073.1	Plasmid	Orlek_et_al.
Klebsiella sp.	NZ_CP015075.2	Plasmid	Orlek_et_al.
Klebsiella sp.	NZ_CP015077.1	Plasmid	Orlek_et_al.
Klebsiella sp.	NZ_CP015078.1	Plasmid	Orlek_et_al.
Klebsiella sp.	NZ_CP015119.1	Plasmid	Orlek_et_al.
Klebsiella sp.	NZ_CP015131.1	Plasmid	Orlek_et_al.
Klebsiella sp.	NZ_CP015132.1	Plasmid	Orlek_et_al.
Klebsiella sp.	NZ_CP015133.1	Plasmid	Orlek_et_al.
Klebsiella sp.	NZ_CP015139.1	Plasmid	Orlek_et_al.
Klebsiella sp.	NZ_CP015140.1	Plasmid	Orlek_et_al.
Klebsiella sp.	NZ_CP015141.1	Plasmid	Orlek_et_al.
Klebsiella sp.	NZ_CP015142.1	Plasmid	Orlek_et_al.
Klebsiella sp.	NZ_CP015143.1	Plasmid	Orlek_et_al.
Klebsiella sp.	NZ_CP015158.1	Plasmid	Orlek_et_al.
Klebsiella sp.	NZ_CP015239.1	Plasmid	Orlek_et_al.
Klebsiella sp.	NZ_CP015242.1	Plasmid	Orlek_et_al.
Klebsiella sp.	NZ_CP015380.1	Plasmid	Orlek_et_al.
Klebsiella sp.	NZ_CP015501.1	Plasmid	Orlek_et_al.
Klebsiella sp.	NZ_CP015502.1	Plasmid	Orlek_et_al.
Klebsiella sp.	NZ_CP015503.1	Plasmid	Orlek_et_al.
Klebsiella sp.	NZ_CP015504.1	Plasmid	Orlek_et_al.
Klebsiella sp.	NZ_CP015505.1	Plasmid	Orlek_et_al.

Klebsiella sp.	NZ_CP015725.1	Plasmid	Orlek_et_al.
Klebsiella sp.	NZ_CP015754.1	Plasmid	Orlek_et_al.
Klebsiella sp.	NZ_CP015755.1	Plasmid	Orlek_et_al.
Klebsiella sp.	NZ_CP015816.1	Plasmid	Orlek_et_al.
Klebsiella sp.	NZ_CP015823.1	Plasmid	Orlek_et_al.
Klebsiella sp.	NZ_CP015824.1	Plasmid	Orlek_et_al.
Klebsiella sp.	NZ_CP015825.1	Plasmid	Orlek_et_al.
Klebsiella sp.	NZ_CP015833.1	Plasmid	Orlek_et_al.
Klebsiella sp.	NZ_CP015844.1	Plasmid	Orlek_et_al.
Klebsiella sp.	NZ_CP015845.1	Plasmid	Orlek_et_al.
Klebsiella sp.	NZ_CP015856.1	Plasmid	Orlek_et_al.
Klebsiella sp.	NZ_CP015913.1	Plasmid	Orlek_et_al.
Klebsiella sp.	NZ_CP015914.1	Plasmid	Orlek_et_al.
Klebsiella sp.	NZ_CP015915.1	Plasmid	Orlek_et_al.
Klebsiella sp.	NZ_CP015916.1	Plasmid	Orlek_et_al.
Klebsiella sp.	NZ_CP015917.1	Plasmid	Orlek_et_al.
Klebsiella sp.	NZ_CP015991.1	Plasmid	Orlek_et_al.
Klebsiella sp.	NZ_CP015996.1	Plasmid	Orlek_et_al.
Klebsiella sp.	NZ_CP015997.1	Plasmid	Orlek_et_al.
Klebsiella sp.	NZ_CP016035.1	Plasmid	Orlek_et_al.
Klebsiella sp.	NZ_CP016036.1	Plasmid	Orlek_et_al.
Klebsiella sp.	NZ_CP016038.1	Plasmid	Orlek_et_al.
Klebsiella sp.	NZ_CP016039.1	Plasmid	Orlek_et_al.
Klebsiella sp.	NZ_CP016040.1	Plasmid	Orlek_et_al.
Klebsiella sp.	NZ_CP016041.1	Plasmid	Orlek_et_al.
Klebsiella sp.	NZ_CP016042.1	Plasmid	Orlek_et_al.
Klebsiella sp.	NZ_CP016160.1	Plasmid	Orlek_et_al.
Klebsiella sp.	NZ_CP016161.1	Plasmid	Orlek_et_al.
Klebsiella sp.	NZ_CP016274.1	Plasmid	Orlek_et_al.
Klebsiella sp.	NZ_CP016275.1	Plasmid	Orlek_et_al.
Klebsiella sp.	NZ_CP016276.1	Plasmid	Orlek_et_al.
Klebsiella sp.	NZ_CP016498.1	Plasmid	Orlek_et_al.
Klebsiella sp.	NZ_CP016508.1	Plasmid	Orlek_et_al.
Klebsiella sp.	NZ_CP016516.1	Plasmid	Orlek_et_al.
Klebsiella sp.	NZ_CP016518.1	Plasmid	Orlek_et_al.
Klebsiella sp.	NZ_CP016519.1	Plasmid	Orlek_et_al.
Klebsiella sp.	NZ_CP016520.1	Plasmid	Orlek_et_al.
Klebsiella sp.	NZ_CP016522.1	Plasmid	Orlek_et_al.
Klebsiella sp.	NZ_CP016526.1	Plasmid	Orlek_et_al.
Klebsiella sp.	NZ_CP016527.1	Plasmid	Orlek_et_al.
Klebsiella sp.	NZ_CP016528.1	Plasmid	Orlek_et_al.
Klebsiella sp.	NZ_CP016529.1	Plasmid	Orlek_et_al.
Klebsiella sp.	NZ_CP016532.1	Plasmid	Orlek_et_al.
Klebsiella sp.	NZ_CP016533.1	Plasmid	Orlek_et_al.
Klebsiella sp.	NZ_CP016547.1	Plasmid	Orlek_et_al.
Klebsiella sp.	NZ_CP016548.1	Plasmid	Orlek_et_al.
Klebsiella sp.	NZ_CP016549.1	Plasmid	Orlek_et_al.
Klebsiella sp.	NZ_CP016550.1	Plasmid	Orlek_et_al.
Klebsiella sp.	NZ_CP016551.1	Plasmid	Orlek_et_al.
Klebsiella sp.	NZ_CP016566.1	Plasmid	Orlek_et_al.
Klebsiella sp.	NZ_CP016567.1	Plasmid	Orlek_et_al.
Klebsiella sp.	NZ_CP016568.1	Plasmid	Orlek_et_al.
Klebsiella sp.	NZ_CP016572.1	Plasmid	Orlek_et_al.
Klebsiella sp.	NZ_CP016575.1	Plasmid	Orlek_et_al.
Klebsiella sp.	NZ_CP016582.1	Plasmid	Orlek_et_al.
Klebsiella sp.	NZ_CP016583.1	Plasmid	Orlek_et_al.
Klebsiella sp.	NZ_CP016584.1	Plasmid	Orlek_et_al.
Klebsiella sp.	NZ_CP016585.1	Plasmid	Orlek_et_al.
Klebsiella sp.	NZ_CP016587.1	Plasmid	Orlek_et_al.
Klebsiella sp.	NZ_CP016763.1	Plasmid	Orlek_et_al.
Klebsiella sp.	NZ_CP016764.1	Plasmid	Orlek_et_al.
Klebsiella sp.	NZ_CP016765.1	Plasmid	Orlek_et_al.
Klebsiella sp.	NZ_CP016810.1	Plasmid	Orlek_et_al.
Klebsiella sp.	NZ_CP016812.1	Plasmid	Orlek_et_al.
Klebsiella sp.	NZ_FO704549.1	Plasmid	Orlek_et_al.
Klebsiella sp.	NZ_FO818638.1	Plasmid	Orlek_et_al.
Klebsiella sp.	NZ_FO818639.1	Plasmid	Orlek_et_al.
Klebsiella sp.	NZ_FO834904.1	Plasmid	Orlek_et_al.
Klebsiella sp.	NZ_FO834905.1	Plasmid	Orlek_et_al.
Klebsiella sp.	NZ_HG941719.1	Plasmid	Orlek_et_al.
Klebsiella sp.	NZ_HG941720.1	Plasmid	Orlek_et_al.
Klebsiella sp.	NZ_LN681228.1	Plasmid	Orlek_et_al.

Klebsiella sp.	NZ_LN824134.1	Plasmid	Orlek_et_al.
Klebsiella sp.	NZ_LN824135.1	Plasmid	Orlek_et_al.
Klebsiella sp.	NZ_LN824137.1	Plasmid	Orlek_et_al.
Klebsiella sp.	NZ_LN824138.1	Plasmid	Orlek_et_al.
Klebsiella sp.	NZ_LN890519.1	Plasmid	Orlek_et_al.
Klebsiella sp.	NZ_LN890521.1	Plasmid	Orlek_et_al.
Klebsiella sp.	NZ_LN890526.1	Plasmid	Orlek_et_al.
Klebsiella sp.	NZ_LN907828.1	Plasmid	Orlek_et_al.
Klebsiella sp.	NZ_LN907829.1	Plasmid	Orlek_et_al.
Klebsiella sp.	NZ_LN999012.1	Plasmid	Orlek_et_al.
Klebsiella sp.	NZ_LN999834.1	Plasmid	Orlek_et_al.
Klebsiella sp.	AB011549.2	Plasmid	CGE_PlasmidFinder
Klebsiella sp.	AB040415.1	Plasmid	CGE_PlasmidFinder
Klebsiella sp.	AB255435.1	Plasmid	CGE_PlasmidFinder
Klebsiella sp.	AB366441.1	Plasmid	CGE_PlasmidFinder
Klebsiella sp.	AB366442.1	Plasmid	CGE_PlasmidFinder
Klebsiella sp.	AB576781.2	Plasmid	CGE_PlasmidFinder
Klebsiella sp.	AB605179.1	Plasmid	CGE_PlasmidFinder
Klebsiella sp.	AB616660.2	Plasmid	CGE_PlasmidFinder
Klebsiella sp.	AE006471.2	Plasmid	CGE_PlasmidFinder
Klebsiella sp.	AE017044.1	Plasmid	CGE_PlasmidFinder
Klebsiella sp.	AE017045.1	Plasmid	CGE_PlasmidFinder
Klebsiella sp.	AE017046.1	Plasmid	CGE_PlasmidFinder
Klebsiella sp.	AF053945.1	Plasmid	CGE_PlasmidFinder
Klebsiella sp.	AF053947.1	Plasmid	CGE_PlasmidFinder
Klebsiella sp.	AF074611.1	Plasmid	CGE_PlasmidFinder
Klebsiella sp.	AF074613.1	Plasmid	CGE_PlasmidFinder
Klebsiella sp.	AF102990.1	Plasmid	CGE_PlasmidFinder
Klebsiella sp.	AF135182.4	Plasmid	CGE_PlasmidFinder
Klebsiella sp.	AF305615.1	Plasmid	CGE_PlasmidFinder
Klebsiella sp.	AF336309.1	Plasmid	CGE_PlasmidFinder
Klebsiella sp.	AF348706.1	Plasmid	CGE_PlasmidFinder
Klebsiella sp.	AF386526.1	Plasmid	CGE_PlasmidFinder
Klebsiella sp.	AF401292.1	Plasmid	CGE_PlasmidFinder
Klebsiella sp.	AF497970.1	Plasmid	CGE_PlasmidFinder
Klebsiella sp.	AF550415.2	Plasmid	CGE_PlasmidFinder
Klebsiella sp.	AF550679.1	Plasmid	CGE_PlasmidFinder
Klebsiella sp.	AGTD01000006.1	Plasmid	CGE_PlasmidFinder
Klebsiella sp.	AJ634602.1	Plasmid	CGE_PlasmidFinder
Klebsiella sp.	AL513384.1	Plasmid	CGE_PlasmidFinder
Klebsiella sp.	AM286416.1	Plasmid	CGE_PlasmidFinder
Klebsiella sp.	AM886293.1	Plasmid	CGE_PlasmidFinder
Klebsiella sp.	AM901564.1	Plasmid	CGE_PlasmidFinder
Klebsiella sp.	AM942760.1	Plasmid	CGE_PlasmidFinder
Klebsiella sp.	AP002527.1	Plasmid	CGE_PlasmidFinder
Klebsiella sp.	AP004237.1	Plasmid	CGE_PlasmidFinder
Klebsiella sp.	AP005147.1	Plasmid	CGE_PlasmidFinder
Klebsiella sp.	AP009242.1	Plasmid	CGE_PlasmidFinder
Klebsiella sp.	AP009243.1	Plasmid	CGE_PlasmidFinder
Klebsiella sp.	AP009245.1	Plasmid	CGE_PlasmidFinder
Klebsiella sp.	AP009246.1	Plasmid	CGE_PlasmidFinder
Klebsiella sp.	AP009379.1	Plasmid	CGE_PlasmidFinder
Klebsiella sp.	AP010910.1	Plasmid	CGE_PlasmidFinder
Klebsiella sp.	AP010954.1	Plasmid	CGE_PlasmidFinder
Klebsiella sp.	AP010955.1	Plasmid	CGE_PlasmidFinder
Klebsiella sp.	AP010959.1	Plasmid	CGE_PlasmidFinder
Klebsiella sp.	AP010962.1	Plasmid	CGE_PlasmidFinder
Klebsiella sp.	AP010963.1	Plasmid	CGE_PlasmidFinder
Klebsiella sp.	AP011954.1	Plasmid	CGE_PlasmidFinder
Klebsiella sp.	AP011958.1	Plasmid	CGE_PlasmidFinder
Klebsiella sp.	AP012208.1	Plasmid	CGE_PlasmidFinder
Klebsiella sp.	AY079200.1	Plasmid	CGE_PlasmidFinder
Klebsiella sp.	AY150843.2	Plasmid	CGE_PlasmidFinder
Klebsiella sp.	AY167049.1	Plasmid	CGE_PlasmidFinder
Klebsiella sp.	AY214164.3	Plasmid	CGE_PlasmidFinder
Klebsiella sp.	AY333434.1	Plasmid	CGE_PlasmidFinder
Klebsiella sp.	AY360321.1	Plasmid	CGE_PlasmidFinder
Klebsiella sp.	AY422214.1	Plasmid	CGE_PlasmidFinder
Klebsiella sp.	AY458016.1	Plasmid	CGE_PlasmidFinder
Klebsiella sp.	AY509003.1	Plasmid	CGE_PlasmidFinder
Klebsiella sp.	AY509004.1	Plasmid	CGE_PlasmidFinder
Klebsiella sp.	AY517905.1	Plasmid	CGE_PlasmidFinder

Klebsiella sp.	AY522431.4	Plasmid	CGE_PlasmidFinder
Klebsiella sp.	AY543071.1	Plasmid	CGE_PlasmidFinder
Klebsiella sp.	AY545598.5	Plasmid	CGE_PlasmidFinder
Klebsiella sp.	AY879342.1	Plasmid	CGE_PlasmidFinder
Klebsiella sp.	AY913943.1	Plasmid	CGE_PlasmidFinder
Klebsiella sp.	AY929248.1	Plasmid	CGE_PlasmidFinder
Klebsiella sp.	BR000038.1	Plasmid	CGE_PlasmidFinder
Klebsiella sp.	BX936400.1	Plasmid	CGE_PlasmidFinder
Klebsiella sp.	CP000035.1	Plasmid	CGE_PlasmidFinder
Klebsiella sp.	CP000037.1	Plasmid	CGE_PlasmidFinder
Klebsiella sp.	CP000039.1	Plasmid	CGE_PlasmidFinder
Klebsiella sp.	CP000244.1	Plasmid	CGE_PlasmidFinder
Klebsiella sp.	CP000306.1	Plasmid	CGE_PlasmidFinder
Klebsiella sp.	CP000309.1	Plasmid	CGE_PlasmidFinder
Klebsiella sp.	CP000602.1	Plasmid	CGE_PlasmidFinder
Klebsiella sp.	CP000603.1	Plasmid	CGE_PlasmidFinder
Klebsiella sp.	CP000604.1	Plasmid	CGE_PlasmidFinder
Klebsiella sp.	CP000648.1	Plasmid	CGE_PlasmidFinder
Klebsiella sp.	CP000649.1	Plasmid	CGE_PlasmidFinder
Klebsiella sp.	CP000650.1	Plasmid	CGE_PlasmidFinder
Klebsiella sp.	CP000836.1	Plasmid	CGE_PlasmidFinder
Klebsiella sp.	CP000858.1	Plasmid	CGE_PlasmidFinder
Klebsiella sp.	CP000913.1	Plasmid	CGE_PlasmidFinder
Klebsiella sp.	CP000965.1	Plasmid	CGE_PlasmidFinder
Klebsiella sp.	CP000966.1	Plasmid	CGE_PlasmidFinder
Klebsiella sp.	CP000973.1	Plasmid	CGE_PlasmidFinder
Klebsiella sp.	CP001059.1	Plasmid	CGE_PlasmidFinder
Klebsiella sp.	CP001064.1	Plasmid	CGE_PlasmidFinder
Klebsiella sp.	CP001065.1	Plasmid	CGE_PlasmidFinder
Klebsiella sp.	CP001122.1	Plasmid	CGE_PlasmidFinder
Klebsiella sp.	CP001123.1	Plasmid	CGE_PlasmidFinder
Klebsiella sp.	CP001162.1	Plasmid	CGE_PlasmidFinder
Klebsiella sp.	CP001362.1	Plasmid	CGE_PlasmidFinder
Klebsiella sp.	CP001369.1	Plasmid	CGE_PlasmidFinder
Klebsiella sp.	CP001384.1	Plasmid	CGE_PlasmidFinder
Klebsiella sp.	CP001386.1	Plasmid	CGE_PlasmidFinder
Klebsiella sp.	CP001587.1	Plasmid	CGE_PlasmidFinder
Klebsiella sp.	CP001591.1	Plasmid	CGE_PlasmidFinder
Klebsiella sp.	CP001595.1	Plasmid	CGE_PlasmidFinder
Klebsiella sp.	CP001847.1	Plasmid	CGE_PlasmidFinder
Klebsiella sp.	CP001919.1	Plasmid	CGE_PlasmidFinder
Klebsiella sp.	CP001926.1	Plasmid	CGE_PlasmidFinder
Klebsiella sp.	CP001927.1	Plasmid	CGE_PlasmidFinder
Klebsiella sp.	CP002089.1	Plasmid	CGE_PlasmidFinder
Klebsiella sp.	CP002090.1	Plasmid	CGE_PlasmidFinder
Klebsiella sp.	CP002168.1	Plasmid	CGE_PlasmidFinder
Klebsiella sp.	CP002180.1	Plasmid	CGE_PlasmidFinder
Klebsiella sp.	CP002187.1	Plasmid	CGE_PlasmidFinder
Klebsiella sp.	CP002247.1	Plasmid	CGE_PlasmidFinder
Klebsiella sp.	CP002474.1	Plasmid	CGE_PlasmidFinder
Klebsiella sp.	CP002488.1	Plasmid	CGE_PlasmidFinder
Klebsiella sp.	CP002615.1	Plasmid	CGE_PlasmidFinder
Klebsiella sp.	CP002730.1	Plasmid	CGE_PlasmidFinder
Klebsiella sp.	CP002732.1	Plasmid	CGE_PlasmidFinder
Klebsiella sp.	CP002887.1	Plasmid	CGE_PlasmidFinder
Klebsiella sp.	CP002957.1	Plasmid	CGE_PlasmidFinder
Klebsiella sp.	CP002969.1	Plasmid	CGE_PlasmidFinder
Klebsiella sp.	CP003027.1	Plasmid	CGE_PlasmidFinder
Klebsiella sp.	CP003028.1	Plasmid	CGE_PlasmidFinder
Klebsiella sp.	CP003035.1	Plasmid	CGE_PlasmidFinder
Klebsiella sp.	CP003110.1	Plasmid	CGE_PlasmidFinder
Klebsiella sp.	CP003111.1	Plasmid	CGE_PlasmidFinder
Klebsiella sp.	CP003112.1	Plasmid	CGE_PlasmidFinder
Klebsiella sp.	CP003113.1	Plasmid	CGE_PlasmidFinder
Klebsiella sp.	CP003223.1	Plasmid	CGE_PlasmidFinder
Klebsiella sp.	CP003224.1	Plasmid	CGE_PlasmidFinder
Klebsiella sp.	CP003225.1	Plasmid	CGE_PlasmidFinder
Klebsiella sp.	CP003387.1	Plasmid	CGE_PlasmidFinder
Klebsiella sp.	CP003417.1	Plasmid	CGE_PlasmidFinder
Klebsiella sp.	CP003684.1	Plasmid	CGE_PlasmidFinder
Klebsiella sp.	CU638872.1	Plasmid	CGE_PlasmidFinder
Klebsiella sp.	CU928144.1	Plasmid	CGE_PlasmidFinder

Klebsiella sp.	CU928146.1	Plasmid	CGE_PlasmidFinder
Klebsiella sp.	CU928147.1	Plasmid	CGE_PlasmidFinder
Klebsiella sp.	CU928148.1	Plasmid	CGE_PlasmidFinder
Klebsiella sp.	CU928149.1	Plasmid	CGE_PlasmidFinder
Klebsiella sp.	CU928159.2	Plasmid	CGE_PlasmidFinder
Klebsiella sp.	DQ017661.1	Plasmid	CGE_PlasmidFinder
Klebsiella sp.	DQ115387.2	Plasmid	CGE_PlasmidFinder
Klebsiella sp.	DQ115388.2	Plasmid	CGE_PlasmidFinder
Klebsiella sp.	DQ298019.1	Plasmid	CGE_PlasmidFinder
Klebsiella sp.	DQ311641.1	Plasmid	CGE_PlasmidFinder
Klebsiella sp.	DQ364638.1	Plasmid	CGE_PlasmidFinder
Klebsiella sp.	DQ381420.1	Plasmid	CGE_PlasmidFinder
Klebsiella sp.	DQ388534.1	Plasmid	CGE_PlasmidFinder
Klebsiella sp.	DQ390454.1	Plasmid	CGE_PlasmidFinder
Klebsiella sp.	DQ390455.1	Plasmid	CGE_PlasmidFinder
Klebsiella sp.	DQ401103.1	Plasmid	CGE_PlasmidFinder
Klebsiella sp.	DQ449578.1	Plasmid	CGE_PlasmidFinder
Klebsiella sp.	DQ659147.1	Plasmid	CGE_PlasmidFinder
Klebsiella sp.	DQ916145.1	Plasmid	CGE_PlasmidFinder
Klebsiella sp.	DQ916413.1	Plasmid	CGE_PlasmidFinder
Klebsiella sp.	DQ995352.1	Plasmid	CGE_PlasmidFinder
Klebsiella sp.	DQ995355.1	Plasmid	CGE_PlasmidFinder
Klebsiella sp.	EF090911.1	Plasmid	CGE_PlasmidFinder
Klebsiella sp.	EF219134.3	Plasmid	CGE_PlasmidFinder
Klebsiella sp.	EF536825.1	Plasmid	CGE_PlasmidFinder
Klebsiella sp.	EF633507.1	Plasmid	CGE_PlasmidFinder
Klebsiella sp.	EU195449.1	Plasmid	CGE_PlasmidFinder
Klebsiella sp.	EU219533.1	Plasmid	CGE_PlasmidFinder
Klebsiella sp.	EU219534.1	Plasmid	CGE_PlasmidFinder
Klebsiella sp.	EU330199.1	Plasmid	CGE_PlasmidFinder
Klebsiella sp.	EU331425.1	Plasmid	CGE_PlasmidFinder
Klebsiella sp.	EU370913.1	Plasmid	CGE_PlasmidFinder
Klebsiella sp.	EU383016.1	Plasmid	CGE_PlasmidFinder
Klebsiella sp.	EU880929.1	Plasmid	CGE_PlasmidFinder
Klebsiella sp.	EU935738.1	Plasmid	CGE_PlasmidFinder
Klebsiella sp.	EU935739.1	Plasmid	CGE_PlasmidFinder
Klebsiella sp.	EU938349.1	Plasmid	CGE_PlasmidFinder
Klebsiella sp.	EU999782.1	Plasmid	CGE_PlasmidFinder
Klebsiella sp.	FJ223605.1	Plasmid	CGE_PlasmidFinder
Klebsiella sp.	FJ223606.1	Plasmid	CGE_PlasmidFinder
Klebsiella sp.	FJ223607.1	Plasmid	CGE_PlasmidFinder
Klebsiella sp.	FJ386569.1	Plasmid	CGE_PlasmidFinder
Klebsiella sp.	FJ449539.1	Plasmid	CGE_PlasmidFinder
Klebsiella sp.	FJ494913.1	Plasmid	CGE_PlasmidFinder
Klebsiella sp.	FJ621586.1	Plasmid	CGE_PlasmidFinder
Klebsiella sp.	FJ621587.1	Plasmid	CGE_PlasmidFinder
Klebsiella sp.	FJ621588.1	Plasmid	CGE_PlasmidFinder
Klebsiella sp.	FJ628167.2	Plasmid	CGE_PlasmidFinder
Klebsiella sp.	FJ666132.1	Plasmid	CGE_PlasmidFinder
Klebsiella sp.	FJ696405.1	Plasmid	CGE_PlasmidFinder
Klebsiella sp.	FJ705806.1	Plasmid	CGE_PlasmidFinder
Klebsiella sp.	FJ876826.1	Plasmid	CGE_PlasmidFinder
Klebsiella sp.	FJ876827.1	Plasmid	CGE_PlasmidFinder
Klebsiella sp.	FJ914220.1	Plasmid	CGE_PlasmidFinder
Klebsiella sp.	FM180569.1	Plasmid	CGE_PlasmidFinder
Klebsiella sp.	FN428572.1	Plasmid	CGE_PlasmidFinder
Klebsiella sp.	FN432031.1	Plasmid	CGE_PlasmidFinder
Klebsiella sp.	FN543094.1	Plasmid	CGE_PlasmidFinder
Klebsiella sp.	FN543095.1	Plasmid	CGE_PlasmidFinder
Klebsiella sp.	FN543096.1	Plasmid	CGE_PlasmidFinder
Klebsiella sp.	FN543503.1	Plasmid	CGE_PlasmidFinder
Klebsiella sp.	FN543504.1	Plasmid	CGE_PlasmidFinder
Klebsiella sp.	FN554767.1	Plasmid	CGE_PlasmidFinder
Klebsiella sp.	FN594520.1	Plasmid	CGE_PlasmidFinder
Klebsiella sp.	FN649417.1	Plasmid	CGE_PlasmidFinder
Klebsiella sp.	FN649418.1	Plasmid	CGE_PlasmidFinder
Klebsiella sp.	FN822745.1	Plasmid	CGE_PlasmidFinder
Klebsiella sp.	FN822746.1	Plasmid	CGE_PlasmidFinder
Klebsiella sp.	FN868832.1	Plasmid	CGE_PlasmidFinder
Klebsiella sp.	FQ482074.1	Plasmid	CGE_PlasmidFinder
Klebsiella sp.	FR687019.1	Plasmid	CGE_PlasmidFinder
Klebsiella sp.	FR850039.1	Plasmid	CGE_PlasmidFinder

Klebsiella sp.	FR851303.1	Plasmid	CGE_PlasmidFinder
Klebsiella sp.	FR851304.1	Plasmid	CGE_PlasmidFinder
Klebsiella sp.	FR851305.1	Plasmid	CGE_PlasmidFinder
Klebsiella sp.	GQ149342.1	Plasmid	CGE_PlasmidFinder
Klebsiella sp.	GQ149343.1	Plasmid	CGE_PlasmidFinder
Klebsiella sp.	GQ149344.1	Plasmid	CGE_PlasmidFinder
Klebsiella sp.	GQ149345.1	Plasmid	CGE_PlasmidFinder
Klebsiella sp.	GQ149346.1	Plasmid	CGE_PlasmidFinder
Klebsiella sp.	GQ149347.1	Plasmid	CGE_PlasmidFinder
Klebsiella sp.	GQ149348.1	Plasmid	CGE_PlasmidFinder
Klebsiella sp.	GQ259888.1	Plasmid	CGE_PlasmidFinder
Klebsiella sp.	GQ374156.1	Plasmid	CGE_PlasmidFinder
Klebsiella sp.	GQ374157.1	Plasmid	CGE_PlasmidFinder
Klebsiella sp.	GQ379901.1	Plasmid	CGE_PlasmidFinder
Klebsiella sp.	GQ398086.1	Plasmid	CGE_PlasmidFinder
Klebsiella sp.	GQ412195.1	Plasmid	CGE_PlasmidFinder
Klebsiella sp.	GU256641.1	Plasmid	CGE_PlasmidFinder
Klebsiella sp.	GU363949.1	Plasmid	CGE_PlasmidFinder
Klebsiella sp.	GU371926.1	Plasmid	CGE_PlasmidFinder
Klebsiella sp.	GU371928.1	Plasmid	CGE_PlasmidFinder
Klebsiella sp.	GU371929.1	Plasmid	CGE_PlasmidFinder
Klebsiella sp.	GU585907.1	Plasmid	CGE_PlasmidFinder
Klebsiella sp.	GU595196.1	Plasmid	CGE_PlasmidFinder
Klebsiella sp.	GU949535.1	Plasmid	CGE_PlasmidFinder
Klebsiella sp.	HE577112.1	Plasmid	CGE_PlasmidFinder
Klebsiella sp.	HE578057.1	Plasmid	CGE_PlasmidFinder
Klebsiella sp.	HE578058.1	Plasmid	CGE_PlasmidFinder
Klebsiella sp.	HE603110.1	Plasmid	CGE_PlasmidFinder
Klebsiella sp.	HE603111.1	Plasmid	CGE_PlasmidFinder
Klebsiella sp.	HE613857.1	Plasmid	CGE_PlasmidFinder
Klebsiella sp.	HE616529.1	Plasmid	CGE_PlasmidFinder
Klebsiella sp.	HE652087.1	Plasmid	CGE_PlasmidFinder
Klebsiella sp.	HE654726.1	Plasmid	CGE_PlasmidFinder
Klebsiella sp.	HE663166.1	Plasmid	CGE_PlasmidFinder
Klebsiella sp.	HM070380.1	Plasmid	CGE_PlasmidFinder
Klebsiella sp.	HM126016.1	Plasmid	CGE_PlasmidFinder
Klebsiella sp.	HM138194.1	Plasmid	CGE_PlasmidFinder
Klebsiella sp.	HM138652.1	Plasmid	CGE_PlasmidFinder
Klebsiella sp.	HM138653.1	Plasmid	CGE_PlasmidFinder
Klebsiella sp.	HM231165.1	Plasmid	CGE_PlasmidFinder
Klebsiella sp.	HM355591.2	Plasmid	CGE_PlasmidFinder
Klebsiella sp.	HM807366.1	Plasmid	CGE_PlasmidFinder
Klebsiella sp.	HM807367.1	Plasmid	CGE_PlasmidFinder
Klebsiella sp.	HM807368.1	Plasmid	CGE_PlasmidFinder
Klebsiella sp.	HQ023861.1	Plasmid	CGE_PlasmidFinder
Klebsiella sp.	HQ023862.1	Plasmid	CGE_PlasmidFinder
Klebsiella sp.	HQ023863.1	Plasmid	CGE_PlasmidFinder
Klebsiella sp.	HQ328804.1	Plasmid	CGE_PlasmidFinder
Klebsiella sp.	HQ404303.1	Plasmid	CGE_PlasmidFinder
Klebsiella sp.	HQ451074.1	Plasmid	CGE_PlasmidFinder
Klebsiella sp.	HQ589350.1	Plasmid	CGE_PlasmidFinder
Klebsiella sp.	HQ834472.1	Plasmid	CGE_PlasmidFinder
Klebsiella sp.	HQ834473.1	Plasmid	CGE_PlasmidFinder
Klebsiella sp.	J01566.1	Plasmid	CGE_PlasmidFinder
Klebsiella sp.	JF267651.1	Plasmid	CGE_PlasmidFinder
Klebsiella sp.	JF267652.1	Plasmid	CGE_PlasmidFinder
Klebsiella sp.	JF267653.1	Plasmid	CGE_PlasmidFinder
Klebsiella sp.	JF274991.1	Plasmid	CGE_PlasmidFinder
Klebsiella sp.	JF274992.1	Plasmid	CGE_PlasmidFinder
Klebsiella sp.	JF436966.1	Plasmid	CGE_PlasmidFinder
Klebsiella sp.	JF503991.1	Plasmid	CGE_PlasmidFinder
Klebsiella sp.	JF714412.2	Plasmid	CGE_PlasmidFinder
Klebsiella sp.	JF776874.1	Plasmid	CGE_PlasmidFinder
Klebsiella sp.	JF779678.1	Plasmid	CGE_PlasmidFinder
Klebsiella sp.	JF785549.1	Plasmid	CGE_PlasmidFinder
Klebsiella sp.	JF785550.1	Plasmid	CGE_PlasmidFinder
Klebsiella sp.	JF813187.1	Plasmid	CGE_PlasmidFinder
Klebsiella sp.	JF813188.1	Plasmid	CGE_PlasmidFinder
Klebsiella sp.	JF828150.1	Plasmid	CGE_PlasmidFinder
Klebsiella sp.	JF927996.1	Plasmid	CGE_PlasmidFinder
Klebsiella sp.	JF937655.1	Plasmid	CGE_PlasmidFinder
Klebsiella sp.	JN087528.1	Plasmid	CGE_PlasmidFinder

Klebsiella sp.	JN087529.1	Plasmid	CGE_PlasmidFinder
Klebsiella sp.	JN157804.1	Plasmid	CGE_PlasmidFinder
Klebsiella sp.	JN183060.1	Plasmid	CGE_PlasmidFinder
Klebsiella sp.	JN183061.1	Plasmid	CGE_PlasmidFinder
Klebsiella sp.	JN194214.1	Plasmid	CGE_PlasmidFinder
Klebsiella sp.	JN205800.1	Plasmid	CGE_PlasmidFinder
Klebsiella sp.	JN232517.1	Plasmid	CGE_PlasmidFinder
Klebsiella sp.	JN233704.1	Plasmid	CGE_PlasmidFinder
Klebsiella sp.	JN233705.2	Plasmid	CGE_PlasmidFinder
Klebsiella sp.	JN247852.1	Plasmid	CGE_PlasmidFinder
Klebsiella sp.	JN247853.1	Plasmid	CGE_PlasmidFinder
Klebsiella sp.	JN253636.1	Plasmid	CGE_PlasmidFinder
Klebsiella sp.	JN393220.1	Plasmid	CGE_PlasmidFinder
Klebsiella sp.	JN566044.1	Plasmid	CGE_PlasmidFinder
Klebsiella sp.	JN626286.1	Plasmid	CGE_PlasmidFinder
Klebsiella sp.	JN687470.1	Plasmid	CGE_PlasmidFinder
Klebsiella sp.	JN861072.1	Plasmid	CGE_PlasmidFinder
Klebsiella sp.	JN885080.1	Plasmid	CGE_PlasmidFinder
Klebsiella sp.	JN885081.1	Plasmid	CGE_PlasmidFinder
Klebsiella sp.	JN887338.1	Plasmid	CGE_PlasmidFinder
Klebsiella sp.	JN935897.1	Plasmid	CGE_PlasmidFinder
Klebsiella sp.	JN935898.1	Plasmid	CGE_PlasmidFinder
Klebsiella sp.	JN935899.1	Plasmid	CGE_PlasmidFinder
Klebsiella sp.	JN983043.1	Plasmid	CGE_PlasmidFinder
Klebsiella sp.	JN983044.1	Plasmid	CGE_PlasmidFinder
Klebsiella sp.	JN983045.1	Plasmid	CGE_PlasmidFinder
Klebsiella sp.	JN983046.1	Plasmid	CGE_PlasmidFinder
Klebsiella sp.	JN983047.1	Plasmid	CGE_PlasmidFinder
Klebsiella sp.	JN983048.1	Plasmid	CGE_PlasmidFinder
Klebsiella sp.	JN995611.1	Plasmid	CGE_PlasmidFinder
Klebsiella sp.	JQ269335.1	Plasmid	CGE_PlasmidFinder
Klebsiella sp.	JQ269336.1	Plasmid	CGE_PlasmidFinder
Klebsiella sp.	JQ349086.2	Plasmid	CGE_PlasmidFinder
Klebsiella sp.	JQ418521.1	Plasmid	CGE_PlasmidFinder
Klebsiella sp.	JQ418522.1	Plasmid	CGE_PlasmidFinder
Klebsiella sp.	JQ418533.1	Plasmid	CGE_PlasmidFinder
Klebsiella sp.	JQ418538.1	Plasmid	CGE_PlasmidFinder
Klebsiella sp.	JQ418540.1	Plasmid	CGE_PlasmidFinder
Klebsiella sp.	JQ418541.1	Plasmid	CGE_PlasmidFinder
Klebsiella sp.	JQ418542.1	Plasmid	CGE_PlasmidFinder
Klebsiella sp.	JQ609357.1	Plasmid	CGE_PlasmidFinder
Klebsiella sp.	JQ776501.1	Plasmid	CGE_PlasmidFinder
Klebsiella sp.	JQ776502.1	Plasmid	CGE_PlasmidFinder
Klebsiella sp.	JQ776503.1	Plasmid	CGE_PlasmidFinder
Klebsiella sp.	JQ776504.1	Plasmid	CGE_PlasmidFinder
Klebsiella sp.	JQ776505.1	Plasmid	CGE_PlasmidFinder
Klebsiella sp.	JQ776506.1	Plasmid	CGE_PlasmidFinder
Klebsiella sp.	JQ776507.1	Plasmid	CGE_PlasmidFinder
Klebsiella sp.	JQ776508.1	Plasmid	CGE_PlasmidFinder
Klebsiella sp.	JQ776509.1	Plasmid	CGE_PlasmidFinder
Klebsiella sp.	JQ824049.1	Plasmid	CGE_PlasmidFinder
Klebsiella sp.	JQ837276.1	Plasmid	CGE_PlasmidFinder
Klebsiella sp.	JX065630.1	Plasmid	CGE_PlasmidFinder
Klebsiella sp.	JX065631.1	Plasmid	CGE_PlasmidFinder
Klebsiella sp.	JX101693.1	Plasmid	CGE_PlasmidFinder
Klebsiella sp.	JX104759.1	Plasmid	CGE_PlasmidFinder
Klebsiella sp.	JX104760.1	Plasmid	CGE_PlasmidFinder
Klebsiella sp.	JX258654.1	Plasmid	CGE_PlasmidFinder
Klebsiella sp.	JX258655.1	Plasmid	CGE_PlasmidFinder
Klebsiella sp.	JX258656.1	Plasmid	CGE_PlasmidFinder
Klebsiella sp.	JX461340.1	Plasmid	CGE_PlasmidFinder
Klebsiella sp.	JX514065.1	Plasmid	CGE_PlasmidFinder
Klebsiella sp.	JX514066.1	Plasmid	CGE_PlasmidFinder
Klebsiella sp.	NC_001848.1	Plasmid	CGE_PlasmidFinder
Klebsiella sp.	NC_002142.1	Plasmid	CGE_PlasmidFinder
Klebsiella sp.	NC_002144.1	Plasmid	CGE_PlasmidFinder
Klebsiella sp.	NC_002487.1	Plasmid	CGE_PlasmidFinder
Klebsiella sp.	NC_002610.1	Plasmid	CGE_PlasmidFinder
Klebsiella sp.	NC_002773.1	Plasmid	CGE_PlasmidFinder
Klebsiella sp.	NC_002809.1	Plasmid	CGE_PlasmidFinder
Klebsiella sp.	NC_003079.1	Plasmid	CGE_PlasmidFinder
Klebsiella sp.	NC_003132.1	Plasmid	CGE_PlasmidFinder

Klebsiella sp.	NC_003134.1	Plasmid	CGE_PlasmidFinder
Klebsiella sp.	NC_003455.1	Plasmid	CGE_PlasmidFinder
Klebsiella sp.	NC_003486.1	Plasmid	CGE_PlasmidFinder
Klebsiella sp.	NC_004837.1	Plasmid	CGE_PlasmidFinder
Klebsiella sp.	NC_005015.1	Plasmid	CGE_PlasmidFinder
Klebsiella sp.	NC_005018.1	Plasmid	CGE_PlasmidFinder
Klebsiella sp.	NC_005248.1	Plasmid	CGE_PlasmidFinder
Klebsiella sp.	NC_005570.1	Plasmid	CGE_PlasmidFinder
Klebsiella sp.	NC_005862.1	Plasmid	CGE_PlasmidFinder
Klebsiella sp.	NC_005923.1	Plasmid	CGE_PlasmidFinder
Klebsiella sp.	NC_005970.1	Plasmid	CGE_PlasmidFinder
Klebsiella sp.	NC_006815.1	Plasmid	CGE_PlasmidFinder
Klebsiella sp.	NC_007635.1	Plasmid	CGE_PlasmidFinder
Klebsiella sp.	NC_008119.1	Plasmid	CGE_PlasmidFinder
Klebsiella sp.	NC_008121.1	Plasmid	CGE_PlasmidFinder
Klebsiella sp.	NC_008444.1	Plasmid	CGE_PlasmidFinder
Klebsiella sp.	NC_008488.1	Plasmid	CGE_PlasmidFinder
Klebsiella sp.	NC_008489.1	Plasmid	CGE_PlasmidFinder
Klebsiella sp.	NC_009344.1	Plasmid	CGE_PlasmidFinder
Klebsiella sp.	NC_009345.1	Plasmid	CGE_PlasmidFinder
Klebsiella sp.	NC_009346.1	Plasmid	CGE_PlasmidFinder
Klebsiella sp.	NC_009347.1	Plasmid	CGE_PlasmidFinder
Klebsiella sp.	NC_009425.1	Plasmid	CGE_PlasmidFinder
Klebsiella sp.	NC_009652.1	Plasmid	CGE_PlasmidFinder
Klebsiella sp.	NC_009653.1	Plasmid	CGE_PlasmidFinder
Klebsiella sp.	NC_009704.1	Plasmid	CGE_PlasmidFinder
Klebsiella sp.	NC_009705.1	Plasmid	CGE_PlasmidFinder
Klebsiella sp.	NC_009716.1	Plasmid	CGE_PlasmidFinder
Klebsiella sp.	NC_009779.1	Plasmid	CGE_PlasmidFinder
Klebsiella sp.	NC_009781.1	Plasmid	CGE_PlasmidFinder
Klebsiella sp.	NC_009786.1	Plasmid	CGE_PlasmidFinder
Klebsiella sp.	NC_009787.1	Plasmid	CGE_PlasmidFinder
Klebsiella sp.	NC_009790.1	Plasmid	CGE_PlasmidFinder
Klebsiella sp.	NC_009807.1	Plasmid	CGE_PlasmidFinder
Klebsiella sp.	NC_009829.1	Plasmid	CGE_PlasmidFinder
Klebsiella sp.	NC_010158.1	Plasmid	CGE_PlasmidFinder
Klebsiella sp.	NC_010261.1	Plasmid	CGE_PlasmidFinder
Klebsiella sp.	NC_010377.1	Plasmid	CGE_PlasmidFinder
Klebsiella sp.	NC_010485.1	Plasmid	CGE_PlasmidFinder
Klebsiella sp.	NC_010487.1	Plasmid	CGE_PlasmidFinder
Klebsiella sp.	NC_010488.1	Plasmid	CGE_PlasmidFinder
Klebsiella sp.	NC_010656.1	Plasmid	CGE_PlasmidFinder
Klebsiella sp.	NC_010659.1	Plasmid	CGE_PlasmidFinder
Klebsiella sp.	NC_010672.1	Plasmid	CGE_PlasmidFinder
Klebsiella sp.	NC_010796.1	Plasmid	CGE_PlasmidFinder
Klebsiella sp.	NC_010894.1	Plasmid	CGE_PlasmidFinder
Klebsiella sp.	NC_010898.1	Plasmid	CGE_PlasmidFinder
Klebsiella sp.	NC_010904.1	Plasmid	CGE_PlasmidFinder
Klebsiella sp.	NC_010993.1	Plasmid	CGE_PlasmidFinder
Klebsiella sp.	NC_011079.1	Plasmid	CGE_PlasmidFinder
Klebsiella sp.	NC_011093.1	Plasmid	CGE_PlasmidFinder
Klebsiella sp.	NC_011148.1	Plasmid	CGE_PlasmidFinder
Klebsiella sp.	NC_011204.1	Plasmid	CGE_PlasmidFinder
Klebsiella sp.	NC_011214.1	Plasmid	CGE_PlasmidFinder
Klebsiella sp.	NC_011215.1	Plasmid	CGE_PlasmidFinder
Klebsiella sp.	NC_011227.1	Plasmid	CGE_PlasmidFinder
Klebsiella sp.	NC_011228.1	Plasmid	CGE_PlasmidFinder
Klebsiella sp.	NC_011350.1	Plasmid	CGE_PlasmidFinder
Klebsiella sp.	NC_011351.1	Plasmid	CGE_PlasmidFinder
Klebsiella sp.	NC_011406.1	Plasmid	CGE_PlasmidFinder
Klebsiella sp.	NC_011407.1	Plasmid	CGE_PlasmidFinder
Klebsiella sp.	NC_011418.1	Plasmid	CGE_PlasmidFinder
Klebsiella sp.	NC_011640.1	Plasmid	CGE_PlasmidFinder
Klebsiella sp.	NC_011759.1	Plasmid	CGE_PlasmidFinder
Klebsiella sp.	NC_011795.1	Plasmid	CGE_PlasmidFinder
Klebsiella sp.	NC_011799.1	Plasmid	CGE_PlasmidFinder
Klebsiella sp.	NC_011897.1	Plasmid	CGE_PlasmidFinder
Klebsiella sp.	NC_011964.1	Plasmid	CGE_PlasmidFinder
Klebsiella sp.	NC_011977.1	Plasmid	CGE_PlasmidFinder
Klebsiella sp.	NC_012006.1	Plasmid	CGE_PlasmidFinder
Klebsiella sp.	NC_012882.1	Plasmid	CGE_PlasmidFinder
Klebsiella sp.	NC_013363.1	Plasmid	CGE_PlasmidFinder

Klebsiella sp.	NC_013367.1	Plasmid	CGE_PlasmidFinder
Klebsiella sp.	NC_013719.1	Plasmid	CGE_PlasmidFinder
Klebsiella sp.	NC_014003.1	Plasmid	CGE_PlasmidFinder
Klebsiella sp.	NC_014027.1	Plasmid	CGE_PlasmidFinder
Klebsiella sp.	NC_014356.1	Plasmid	CGE_PlasmidFinder
Klebsiella sp.	NC_014543.1	Plasmid	CGE_PlasmidFinder
Klebsiella sp.	NC_015054.1	Plasmid	CGE_PlasmidFinder
Klebsiella sp.	NC_015068.1	Plasmid	CGE_PlasmidFinder
Klebsiella sp.	NC_015154.1	Plasmid	CGE_PlasmidFinder
Klebsiella sp.	NC_015392.1	Plasmid	CGE_PlasmidFinder
Klebsiella sp.	NC_015570.1	Plasmid	CGE_PlasmidFinder
Klebsiella sp.	NC_015575.1	Plasmid	CGE_PlasmidFinder
Klebsiella sp.	NC_015972.1	Plasmid	CGE_PlasmidFinder
Klebsiella sp.	NC_016823.1	Plasmid	CGE_PlasmidFinder
Klebsiella sp.	NC_016824.1	Plasmid	CGE_PlasmidFinder
Klebsiella sp.	NC_016834.1	Plasmid	CGE_PlasmidFinder
Klebsiella sp.	NC_016840.1	Plasmid	CGE_PlasmidFinder
Klebsiella sp.	NC_016841.1	Plasmid	CGE_PlasmidFinder
Klebsiella sp.	NC_016847.1	Plasmid	CGE_PlasmidFinder
Klebsiella sp.	NC_016859.1	Plasmid	CGE_PlasmidFinder
Klebsiella sp.	NC_016862.1	Plasmid	CGE_PlasmidFinder
Klebsiella sp.	NC_016976.1	Plasmid	CGE_PlasmidFinder
Klebsiella sp.	NC_016979.1	Plasmid	CGE_PlasmidFinder
Klebsiella sp.	NC_017097.1	Plasmid	CGE_PlasmidFinder
Klebsiella sp.	NC_017156.1	Plasmid	CGE_PlasmidFinder
Klebsiella sp.	NC_017159.1	Plasmid	CGE_PlasmidFinder
Klebsiella sp.	NC_017320.1	Plasmid	CGE_PlasmidFinder
Klebsiella sp.	NC_017321.1	Plasmid	CGE_PlasmidFinder
Klebsiella sp.	NC_017330.1	Plasmid	CGE_PlasmidFinder
Klebsiella sp.	NC_017629.1	Plasmid	CGE_PlasmidFinder
Klebsiella sp.	NC_017648.1	Plasmid	CGE_PlasmidFinder
Klebsiella sp.	NC_017649.1	Plasmid	CGE_PlasmidFinder
Klebsiella sp.	NC_017655.1	Plasmid	CGE_PlasmidFinder
Klebsiella sp.	NC_017659.1	Plasmid	CGE_PlasmidFinder
Klebsiella sp.	NC_017723.1	Plasmid	CGE_PlasmidFinder
Klebsiella sp.	U67194.4	Plasmid	CGE_PlasmidFinder
Klebsiella sp.	X01654.1	Plasmid	CGE_PlasmidFinder
Klebsiella sp.	LT985253	Plasmid	ReplicolScope
Klebsiella sp.	LT985262	Plasmid	ReplicolScope
Klebsiella sp.	NC_014615	Plasmid	ReplicolScope
Klebsiella sp.	LO017737	Plasmid	ReplicolScope
Klebsiella sp.	LO017736	Plasmid	ReplicolScope
Klebsiella sp.	LO017738	Plasmid	ReplicolScope
Klebsiella sp.	LT985215	Plasmid	ReplicolScope
Klebsiella sp.	LT985214	Plasmid	ReplicolScope
Klebsiella sp.	LT985213	Plasmid	ReplicolScope
Klebsiella sp.	LT985217	Plasmid	ReplicolScope
Klebsiella sp.	LT985216	Plasmid	ReplicolScope
Klebsiella sp.	LT985218	Plasmid	ReplicolScope
Klebsiella sp.	LT985220	Plasmid	ReplicolScope
Klebsiella sp.	LT985219	Plasmid	ReplicolScope
Klebsiella sp.	LT985221	Plasmid	ReplicolScope
Klebsiella sp.	LT985222	Plasmid	ReplicolScope
Klebsiella sp.	LT985223	Plasmid	ReplicolScope
Klebsiella sp.	LT985226	Plasmid	ReplicolScope
Klebsiella sp.	LT985227	Plasmid	ReplicolScope
Klebsiella sp.	LT985228	Plasmid	ReplicolScope
Klebsiella sp.	LT985238	Plasmid	ReplicolScope
Klebsiella sp.	LT985239	Plasmid	ReplicolScope
Klebsiella sp.	LT985225	Plasmid	ReplicolScope
Klebsiella sp.	LT985224	Plasmid	ReplicolScope
Klebsiella sp.	LT985240	Plasmid	ReplicolScope
Klebsiella sp.	LT985234	Plasmid	ReplicolScope
Klebsiella sp.	LT985236	Plasmid	ReplicolScope
Klebsiella sp.	LT985237	Plasmid	ReplicolScope
Klebsiella sp.	LT985235	Plasmid	ReplicolScope
Klebsiella sp.	LT985232	Plasmid	ReplicolScope
Klebsiella sp.	LT985233	Plasmid	ReplicolScope
Klebsiella sp.	LT985229	Plasmid	ReplicolScope
Klebsiella sp.	LT985230	Plasmid	ReplicolScope
Klebsiella sp.	LT985231	Plasmid	ReplicolScope
Klebsiella sp.	LT985250	Plasmid	ReplicolScope

Klebsiella sp.	LT985244	Plasmid	ReplicolScope
Klebsiella sp.	LT985241	Plasmid	ReplicolScope
Klebsiella sp.	LT985243	Plasmid	ReplicolScope
Klebsiella sp.	LT985245	Plasmid	ReplicolScope
Klebsiella sp.	LT985242	Plasmid	ReplicolScope
Klebsiella sp.	LT985259	Plasmid	ReplicolScope
Klebsiella sp.	LT985251	Plasmid	ReplicolScope
Klebsiella sp.	LT985249	Plasmid	ReplicolScope
Klebsiella sp.	LT985248	Plasmid	ReplicolScope
Klebsiella sp.	LT985247	Plasmid	ReplicolScope
Klebsiella sp.	LT985246	Plasmid	ReplicolScope
Klebsiella sp.	LT985256	Plasmid	ReplicolScope
Klebsiella sp.	LT985255	Plasmid	ReplicolScope
Klebsiella sp.	LT985254	Plasmid	ReplicolScope
Klebsiella sp.	LT985257	Plasmid	ReplicolScope
Klebsiella sp.	LT985261	Plasmid	ReplicolScope
Klebsiella sp.	LT985252	Plasmid	ReplicolScope
Klebsiella sp.	LT985264	Plasmid	ReplicolScope
Klebsiella sp.	LT985266	Plasmid	ReplicolScope
Klebsiella sp.	LT985260	Plasmid	ReplicolScope
Klebsiella sp.	LT985263	Plasmid	ReplicolScope
Klebsiella sp.	LT985258	Plasmid	ReplicolScope
Klebsiella sp.	LT985322	Plasmid	ReplicolScope
Klebsiella sp.	LT985270	Plasmid	ReplicolScope
Klebsiella sp.	LT985265	Plasmid	ReplicolScope
Klebsiella sp.	LT985268	Plasmid	ReplicolScope
Klebsiella sp.	LT985271	Plasmid	ReplicolScope
Klebsiella sp.	LT985279	Plasmid	ReplicolScope
Klebsiella sp.	LT985267	Plasmid	ReplicolScope
Klebsiella sp.	LT985272	Plasmid	ReplicolScope
Klebsiella sp.	LT985269	Plasmid	ReplicolScope
Klebsiella sp.	LT985294	Plasmid	ReplicolScope
Klebsiella sp.	LT985295	Plasmid	ReplicolScope
Klebsiella sp.	LT985277	Plasmid	ReplicolScope
Klebsiella sp.	LT985274	Plasmid	ReplicolScope
Klebsiella sp.	LT985276	Plasmid	ReplicolScope
Klebsiella sp.	LT985278	Plasmid	ReplicolScope
Klebsiella sp.	LT985287	Plasmid	ReplicolScope
Klebsiella sp.	LT985275	Plasmid	ReplicolScope
Klebsiella sp.	LT985281	Plasmid	ReplicolScope
Klebsiella sp.	LT985273	Plasmid	ReplicolScope
Klebsiella sp.	LT985288	Plasmid	ReplicolScope
Klebsiella sp.	LT985283	Plasmid	ReplicolScope
Klebsiella sp.	LT985282	Plasmid	ReplicolScope
Klebsiella sp.	LT985280	Plasmid	ReplicolScope
Klebsiella sp.	LT985286	Plasmid	ReplicolScope
Klebsiella sp.	LT985284	Plasmid	ReplicolScope
Klebsiella sp.	LT985285	Plasmid	ReplicolScope
Klebsiella sp.	LT985297	Plasmid	ReplicolScope
Klebsiella sp.	LT985296	Plasmid	ReplicolScope
Klebsiella sp.	LT985293	Plasmid	ReplicolScope
Klebsiella sp.	LT985289	Plasmid	ReplicolScope
Klebsiella sp.	LT985317	Plasmid	ReplicolScope
Klebsiella sp.	LT985302	Plasmid	ReplicolScope
Klebsiella sp.	LT985303	Plasmid	ReplicolScope
Klebsiella sp.	LT985290	Plasmid	ReplicolScope
Klebsiella sp.	LT985291	Plasmid	ReplicolScope
Klebsiella sp.	LT985292	Plasmid	ReplicolScope
Klebsiella sp.	LT985305	Plasmid	ReplicolScope
Klebsiella sp.	LT985321	Plasmid	ReplicolScope
Klebsiella sp.	LT985320	Plasmid	ReplicolScope
Klebsiella sp.	LT985307	Plasmid	ReplicolScope
Klebsiella sp.	LT985298	Plasmid	ReplicolScope
Klebsiella sp.	LT985306	Plasmid	ReplicolScope
Klebsiella sp.	LT985304	Plasmid	ReplicolScope
Klebsiella sp.	LT985300	Plasmid	ReplicolScope
Klebsiella sp.	LT985301	Plasmid	ReplicolScope
Klebsiella sp.	LT985299	Plasmid	ReplicolScope
Klebsiella sp.	LT985314	Plasmid	ReplicolScope
Klebsiella sp.	LT985313	Plasmid	ReplicolScope
Klebsiella sp.	LT985312	Plasmid	ReplicolScope
Klebsiella sp.	LT985309	Plasmid	ReplicolScope

Klebsiella sp.	LT985310	Plasmid	ReplicolScope
Klebsiella sp.	LT985311	Plasmid	ReplicolScope
Klebsiella sp.	LT985308	Plasmid	ReplicolScope
Klebsiella sp.	LT985318	Plasmid	ReplicolScope
Klebsiella sp.	LT985319	Plasmid	ReplicolScope
Klebsiella sp.	LT985316	Plasmid	ReplicolScope
Klebsiella sp.	LT985315	Plasmid	ReplicolScope

Supplementary table S2. List of the sequences analysed with ECOPE and PlasmidSPAdes

SRA_accession	Assembly_accession	Chromosome_accession	Plasmid_1_accession	Plasmid_2_accession	Plasmid_3_accession	Plasmid_4_accession	Plasmid_5_accession	Plasmid_6_accession	Plasmid_7_accession	Plasmid_8_accession
SRR647664	GCA_000299255.1	NC_018650.1	NC_018651.1	NC_018654.1	NC_018652.1					
SRR647492	GCA_000299455.1	NC_018658.1	NC_018666.1	NC_018659.1	NC_018660.1					
SRR647666	GCA_000299475.1	NC_018661.1	NC_018662.1	NC_018663.1						
SRR2533563	GCA_000464955.2	NZ_CP023541.1	NZ_CP023542.1							
SRR1424625	GCA_000725305.1	NZ_CP008801.1								
SRR1509643	GCA_000732965.1	NZ_CP008957.1	NZ_CP008958.1							
SRR2889880	GCA_000743255.1	NZ_CP009072.1	NZ_CP009073.1	NZ_CP009074.1						
SRR3492223	GCA_000831565.1	NZ_CP010344.1	NZ_CP010345.1							
SRR3883475	GCA_000833145.1	NZ_CP010816.1								
ERR689069	GCA_000981485.1	NZ_LN832404.1								
SRR2052522	GCA_001039415.1	NZ_CP011938.1								
SRR2014554	GCA_001276585.2	NZ_CP011113.2								
SRR1570041	GCA_001420955.1	NZ_CP013025.1	NZ_CP013024.1	NZ_CP013026.1	NZ_CP013027.1					
SRR2970646	GCA_001469815.1	NZ_CP013658.1	NZ_CP013657.1							
SRR2914453	GCA_001651925.2	NZ_CP015846.1	NZ_CP015847.2							
SRR2914455	GCA_001651945.1	NZ_CP015842.1								
SRR3615375	GCA_001660585.1	NZ_CP016007.1	NZ_CP016008.1	NZ_CP016009.1						
SRR3465539	GCA_001663475.1	NZ_CP015159.1	NZ_CP015161.1	NZ_CP015160.1						
SRR3221963	GCA_001675145.1	NZ_CP014667.1	NZ_CP014668.1	NZ_CP014669.1						
SRR3191403	GCA_001695515.1	NZ_CP016625.1	NZ_CP016626.1							
SRR3195851	GCA_001753445.1	NZ_CP017434.1	NZ_CP017435.1							
SRR3195839	GCA_001753465.1	NZ_CP017436.1	NZ_CP017437.1							
SRR3195840	GCA_001753485.1	NZ_CP017444.1	NZ_CP017445.1							
SRR3195852	GCA_001753505.1	NZ_CP017438.1	NZ_CP017439.1							
SRR3195836	GCA_001753525.1	NZ_CP017446.1	NZ_CP017447.1							
SRR3195765	GCA_001753545.1	NZ_CP017440.1	NZ_CP017441.1							
SRR3195841	GCA_001753565.1	NZ_CP017442.1	NZ_CP017443.1							
SRR5177719	GCA_001999185.1	NZ_CP019629.1								
SRR3999069	GCA_002011945.1	NZ_CP018948.1	NZ_CP018947.1	NZ_CP018946.1	NZ_CP018944.1	NZ_CP018943.1	NZ_CP018942.1	NZ_CP018941.1	NZ_CP018940.1	NZ_CP018945.1
SRR3999062	GCA_002011965.1	NZ_CP018953.1	NZ_CP018952.1	NZ_CP018951.1	NZ_CP018950.1	NZ_CP018949.1				
SRR3999063	GCA_002011985.1	NZ_CP018957.1	NZ_CP018955.1	NZ_CP018954.1	NZ_CP018956.1					
SRR3999067	GCA_002012005.1	NZ_CP018965.1	NZ_CP018964.1	NZ_CP018963.1						
SRR3999060	GCA_002012025.1	NZ_CP018970.1	NZ_CP018969.1	NZ_CP018967.1	NZ_CP018966.1	NZ_CP018968.1				
SRR3999078	GCA_002012045.1	NZ_CP018976.1	NZ_CP018975.1	NZ_CP018973.1	NZ_CP018972.1	NZ_CP018971.1	NZ_CP018974.1			
SRR3999086	GCA_002012065.1	NZ_CP018979.1	NZ_CP018978.1	NZ_CP018977.1						
SRR3999096	GCA_002012085.1	NZ_CP018991.1	NZ_CP018990.1	NZ_CP018988.1	NZ_CP018987.1	NZ_CP018986.1	NZ_CP018985.1	NZ_CP018984.1	NZ_CP018989.1	
SRR3999064	GCA_002012105.1	NZ_CP018995.1	NZ_CP018994.1	NZ_CP018993.1	NZ_CP018992.1					
SRR3999079	GCA_002012125.1	NZ_CP019005.1	NZ_CP019002.1	NZ_CP019003.1	NZ_CP019004.1	NZ_CP019001.1				
SRR3999089	GCA_002012145.1	NZ_CP019008.1	NZ_CP019007.1	NZ_CP019006.1						
SRR3999061	GCA_002012165.1	NZ_CP019015.1	NZ_CP019013.1	NZ_CP019014.1						
SRR3999095	GCA_002012185.1	NZ_CP018962.1	NZ_CP018961.1	NZ_CP018960.1	NZ_CP018958.1	NZ_CP018959.1				
SRR3999101	GCA_002012205.1	NZ_CP018983.1	NZ_CP018982.1	NZ_CP018980.1	NZ_CP018981.1					
SRR3999098	GCA_002012225.1	NZ_CP019029.1	NZ_CP019028.1	NZ_CP019027.1	NZ_CP019025.1	NZ_CP019024.1	NZ_CP019023.1	NZ_CP019022.1	NZ_CP019021.1	NZ_CP019026.1
SRR3999087	GCA_002012245.1	NZ_CP019000.1	NZ_CP018998.1	NZ_CP018997.1	NZ_CP018996.1	NZ_CP018999.1				
SRR3999081	GCA_002012265.1	NZ_CP019012.1	NZ_CP019011.1	NZ_CP019009.1	NZ_CP019010.1					
SRR3999091	GCA_002012305.1	NZ_CP019020.1	NZ_CP019019.1	NZ_CP019018.1	NZ_CP019016.1	NZ_CP019017.1				
SRR5168379	GCA_002055635.1	NZ_CP020048.1	NZ_CP020049.1	NZ_CP020050.1	NZ_CP020051.1					
SRR5168215	GCA_002056065.1	NZ_CP020058.1	NZ_CP020059.1	NZ_CP020060.1						
SRR5168223	GCA_002056145.1	NZ_CP020055.1	NZ_CP020056.1	NZ_CP020057.1						
SRR5168233	GCA_002056635.1	NZ_CP020116.1	NZ_CP020117.1	NZ_CP020118.1	NZ_CP020119.1					
SRR5627612	GCA_002077195.1	NZ_CP017220.1	NZ_CP017221.1	NZ_CP017222.1	NZ_CP017223.1	NZ_CP017224.1	NZ_CP017225.1			
SRR3931165	GCA_002105735.1	NZ_CP020835.1	NZ_CP020836.1							
SRR5168500	GCA_002180055.1	NZ_CP021532.1	NZ_CP021533.1	NZ_CP021534.1						
SRR5168382	GCA_002180095.1	NZ_CP021535.1	NZ_CP021536.1	NZ_CP021537.1	NZ_CP021538.1					
SRR5168522	GCA_002180135.1	NZ_CP021683.1	NZ_CP021680.1	NZ_CP021681.1	NZ_CP021682.1	NZ_CP021684.1				
SRR5168210	GCA_002180195.1	NZ_CP021689.1	NZ_CP021690.1							
SRR5168506	GCA_002180215.1	NZ_CP021691.1	NZ_CP021692.1	NZ_CP021693.1						
SRR5168391	GCA_002180275.1	NZ_CP021722.1	NZ_CP021719.1	NZ_CP021720.1	NZ_CP021721.1					
SRR5168373	GCA_002192275.1	NZ_CP021732.1	NZ_CP021733.1	NZ_CP021734.1	NZ_CP021735.1					
SRR5168503	GCA_002192295.1	NZ_CP021736.1	NZ_CP021737.1	NZ_CP021738.1	NZ_CP021739.1					
SRR5168479	GCA_002201835.1	NZ_CP021879.1	NZ_CP021880.1	NZ_CP021881.1	NZ_CP021882.1					
SRR5167858	GCA_002202175.1	NZ_CP021935.1	NZ_CP021936.1	NZ_CP021937.1	NZ_CP021938.1					
SRR1714120	GCA_002249955.1	NZ_CP022689.1	NZ_CP022688.1							
SRR5688555	GCA_002278115.2	NZ_CP023142.1	NZ_CP023143.1	NZ_CP023144.1	NZ_CP023145.1					
DRR065948	GCA_002357875.1	NZ_AP017620.1	NZ_AP017621.1	NZ_AP017622.1						
DRR065947	GCA_002357895.1	NZ_AP017617.1	NZ_AP017618.1	NZ_AP017619.1						
DRR065946	GCA_002357915.1	NZ_AP017610.1	NZ_AP017611.1	NZ_AP017612.1	NZ_AP017613.1	NZ_AP017614.1	NZ_AP017615.1	NZ_AP017616.1		
SRR3290448	GCA_002741175.1	NZ_CP024228.1	NZ_CP024229.1	NZ_CP024230.1	NZ_CP024231.1					
SRR3341739	GCA_002741335.1	NZ_CP024260.1	NZ_CP024261.1	NZ_CP024262.1						
SRR5617250	GCA_002741535.1	NZ_CP024269.1	NZ_CP024270.1	NZ_CP024271.1	NZ_CP024272.1					

SRR3360246	GCA_002741555.1	NZ_CP024273.1	NZ_CP024274.1							
SRR3112258	GCA_002787215.1	NZ_CP024851.1	NZ_CP024852.1	NZ_CP024853.1	NZ_CP024854.1					
SRR3112265	GCA_002787315.1	NZ_CP024859.1	NZ_CP024860.1	NZ_CP024861.1						
SRR3112266	GCA_002788115.1	NZ_CP024862.1	NZ_CP024863.1	NZ_CP024864.1	NZ_CP024865.1					
SRR3112262	GCA_002788295.1	NZ_CP024855.1	NZ_CP024856.1	NZ_CP024857.1	NZ_CP024858.1					
SRR3112270	GCA_002796445.1	NZ_CP024889.1	NZ_CP024888.1							
SRR3112268	GCA_002796585.1	NZ_CP024886.1	NZ_CP024887.1							

Supplementary table S3. Detail count of contigs for each assignment type with PlaScope, Plasflow and cBar

	PlaScope predictions			Plasflow predictions			cBar predictions	
	Plasmid	Chromosome	Unclassified	Plasmid	Chromosome	Unclassified	Plasmid	Chromosome
True Positive	1123	-	-	1106	-	-	954	-
False Positive	52	-	-	2983	-	-	3644	-
True Negative	-	8938	224	-	4076	2155	-	5570
False Negative	-	20	153	-	22	168	-	342

Supplementary table S4. Results of Plasmidfinder and Resfinder analysis on PlaScope predictions (i.e. chromosome, plasmid and unclassified)

Isolates	Isolate_1	Isolate_2	Isolate_3	Isolate_4	Isolate_5	Isolate_6	Isolate_7	Isolate_8	Isolate_9	Isolate_10	Isolate_11	Isolate_12
**Resfinder results on chromosome predictions**	blaSHV-11, oqxA, oqxB, fosA	blaSHV-11, oqxA, oqxB, fosA	blaSHV-11, blaCTX-M-15, fosA, oqxA, oqxB,	blaSHV-11, blaCTX-M-15, fosA, oqxA, oqxB,	blaSHV-11, oqxA, oqxB, fosA	blaSHV-11, oqxA, oqxB, fosA	blaSHV-11, oqxA, oqxB, fosA	blaSHV-11, blaCTX-M-15, fosA, oqxA, oqxB,	blaSHV-11, blaCTX-M-15, fosA, oqxA, oqxB,	blaSHV-11, blaCTX-M-15, fosA, oqxA, oqxB,	blaSHV-11, blaCTX-M-15, fosA, oqxA, oqxB,	blaSHV-11, blaCTX-M-15, fosA, oqxA, oqxB,
**Plasmidfinder results on plasmid predictions**	H1B	H1B, Col(MG828)*	IncFIB, IncFII, IncN2, IncR, ColKP3, ColpVC, Col(MG828)	IncFIB, IncFII, IncN2, IncR, ColKP3, ColpVC, Col(MG828)	H1B, Col(MG828)*	H1B, Col(MG828)*, ColKP3*, ColpVC*	H1B	IncFIB, IncFII, IncN2, IncR, ColKP3, ColpVC	IncFIB, IncFII, IncN2, IncR, ColKP3, ColpVC	IncFIB, IncFII, IncN2, IncR, ColKP3, ColpVC, Col(MG828)	IncFIB, IncFII, IncN2, IncR, ColKP3, ColpVC, Col(MG828)	IncFIB, IncFII, IncN2, IncR, ColKP3, ColpVC, Col(MG828)
**ResFinder results on plasmid predictions**	-	-	blaOXA-181, blaTEM-1B, rmtf, dfrA12, aph(3'')-Ib, aph(6)Id, catA2	blaOXA-181, blaTEM-1B, aph(3'')-Ib, aph(6)Id, catA2	-	-	-	blaOXA-181, blaTEM-1B, aph(3'')-Ib, aph(6)Id, catA2	blaOXA-181, blaTEM-1B, rmtf, dfrA12, aph(3'')-Ib, aph(6)Id, catA2	blaOXA-181, blaTEM-1B, aph(3'')-Ib, aph(6)Id, catA2	blaOXA-181, blaTEM-1B, aph(3'')-Ib, aph(6)Id, catA2	blaOXA-181, blaTEM-1B, aph(3'')-Ib, aph(6)Id, catA2
**PlasmidFinder results on unclassified predictions**	ColpVC*	ColKP3*	-	-	ColpVC*	-	-	-	Col(MG828)	-	-	-
**Resfinder results on unclassified predictions**	-	-	QnrB1, mph(A), dfrA14, arr-2	QnrB1, mph(A), dfrA14, arr-2, dfrA12, rmtf_1	-	-	-	QnrB1, mph(A), dfrA14, arr-2, dfrA12, rmtf_1	QnrB1, mph(A), dfrA14, arr-2	QnrB1, mph(A), dfrA14, arr-2, dfrA12, rmtf_1	QnrB1, mph(A), dfrA14, arr-2, dfrA12, rmtf_1	QnrB1, mph(A), dfrA14, arr-2, dfrA12, rmtf_1

*These replicon sequences are not reported in the original publication
